# Supplementary material for: Iodine-catalyzed diazo activation to access radical reactivity
Source: Nat Commun. 2018 May 17;9:1972. doi: 10.1038/s41467-018-04331-4 (PMC5958049; doi:10.1038/s41467-018-04331-4)
Supplement: Supplementary file 2 — Supplementary Information [file 41467_2018_4331_MOESM2_ESM.pdf]

Supplementary Information

Iodine catalyzed diazo activation to access radical reactivity

Li et, al.

Supplementary Methods

General Information:

All reagents purchased from commercial sources were used as received. The silica gel for column chromatography was supplied as 300–400 meshes. The ^{1}H and ^{13}C NMR spectra were recorded on a Bruker AVANCE III spectrometer and are referenced to the residual solvent signals (7.26 ppm for ^{1}H and 77.0 ppm for ^{13}C in $CDCl_3$, 2.50 ppm for ^{1}H and 39.5 ppm for ^{13}C in d^6-DMSO). The HRMS spectra were recorded on a Bruker MicroTOF Q II spectrometer. EPR spectra were recorded on a Bruker A-300 spectrometer.

Synthesis of Ethyl Diiodoacetate

To a 50 mL Schlenk tubes with a stir bar was added ethyl diazoacetate **1a** (23 mg, 0.2 mmol) and 2 mL of $CDCl_3$. Then iodine (51 mg, 0.2 mmol) was added to the above solution. The reaction mixture was stirred under argon at room temperture for 0.5 h. The reaction was very clean by crude NMR. **Ethyl 2,2-diiodoacetate 1a'** *(the HNMR date is same with Lei's paper, see ref. 35)*: ^{1}H NMR (400 MHz, $CDCl_3$) δ 5.34 (s, 1 H), 4.28 (q, *J* = 7.1 Hz, 2 H), 1.31 (t, *J* = 7.1 Hz, 3 H). GC-MS: 339.7, 294.8, 266.8, 212.8, 184.8, 168.8, 156.8, 139.8, 126.9.

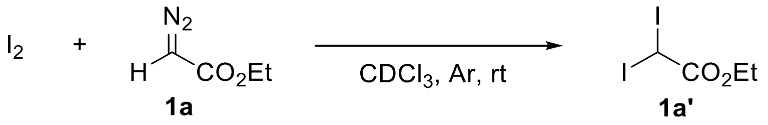

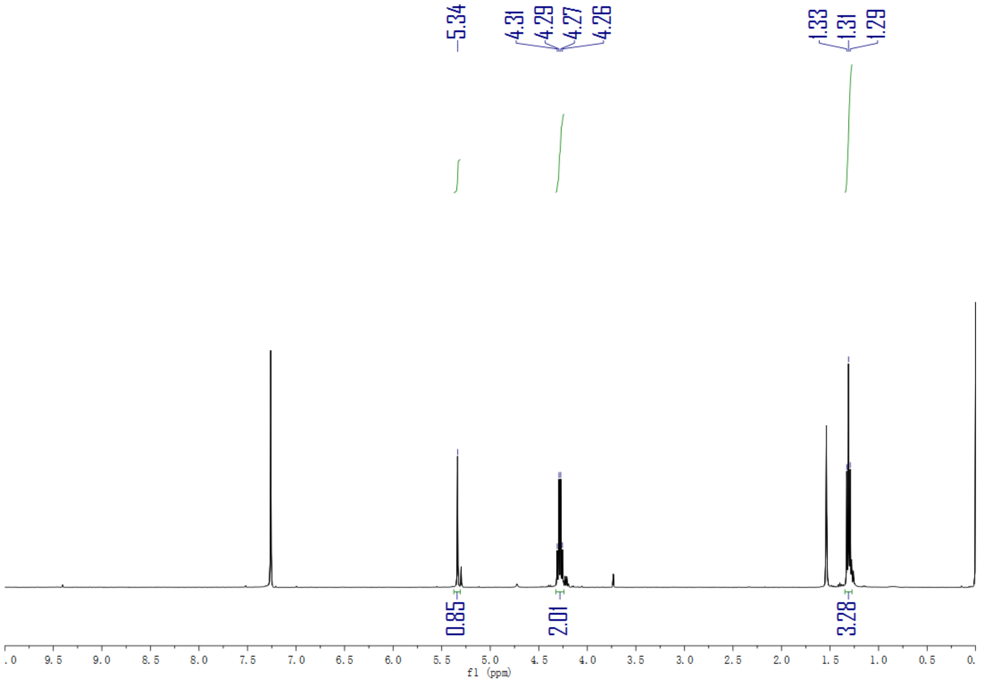

Supplementary Table 1. Optimization of Reaction under Photo-initiated Conditions

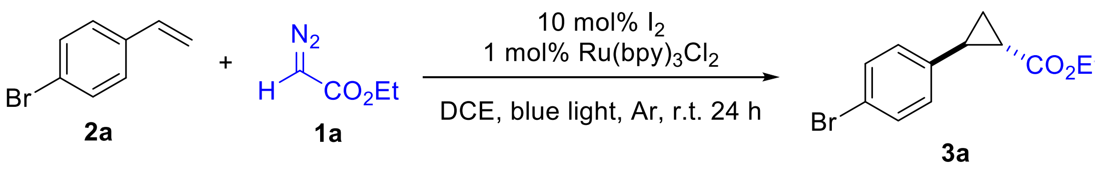

Alternation from above condition	**2a** conv.	**3a** yield
none	100%	96%
No I_2	0%	n.d.
No $Ru(bpy)_3Cl_2$	0%	n.d.
White light instead of blue light	95%	85%
In the dark	0%	n.d.
$Ir(dtbbpy)(bpy)_2PF_6$	75%	65%
Eosin Y instead of $Ru(bpy)_3Cl_2$	0%	n.d.
10 mol% **1a'** instead of I_2	100%	94%
10 mol% Br_2 instead of I_2	0%	n.d.
No I_2, 1 equiv. of **1a'** instead of **1a**	< 5%	trace
No I_2, 1 equiv. of **1a'** instead of **1a**, i-Pr_2EtN (1 equiv.), $Na_2S_2O_3$ (1 equiv.), MeCN instead of DCE (Suero's conditions, ref. 22)	55%	35%
Addition of 1 equiv. of TEMPO	0%	n.d.

Reaction conditions: 1 mol% photocatalyst and 10 mol% I_2 were added to a DCE solution (3 mL) of **2a** (0.3 mmol) and EDA **1a** (0.3 mmol), and reaction was kept under argon (degassed) at room temperature under blue light for 24 h. Conversion and yield were determined by ^{1}H NMR spectroscopy using 1,3,5-trimethoxybenzene as internal standard.

Radical Clock Experiment under Photo-initiated Conditions

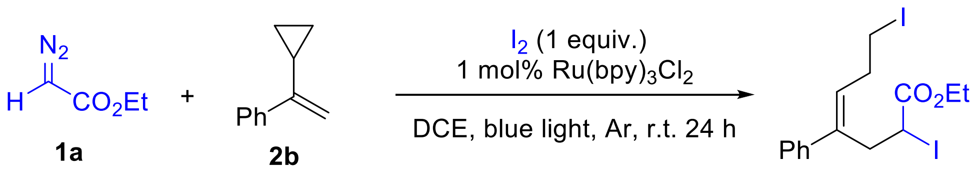

4, 75% (15:1)

To a 50 mL Schlenk tubes with a stir bar was added olefin **2b** (43 mg, 0.3 mmol), diazo compound **1a** (35 mg, 0.3 mmol) and 3 mL of DCE, then $Ru(bpy)_3Cl_2 \cdot 6H_2O$ (2 mg, 0.003 mmol) and I_2 (8 mg, 0.03 mmol) were added. The Schlenk tube was vacuumed and purged with argon three times before it was tightly screw-capped. The reaction mixture was stirred at room temperature under blue light for 24 h. The reaction solution was evaporated, and the residue was purified by column chromatography (PE/EA) to afford the desired product **4** in 75% yield (E/Z = 15:1). ***Ethyl-2,7-diiodo-4-phenylhept-4-enoate* 4** (E:Z = 15:1)**:** ^{1}H NMR (400 MHz, $CDCl_3$) δ 7.42–7.23 (m, 5 H), 5.68 (t, J = 7.2 Hz, 1 H), 4.20–4.03 (m, 3 H), 3.28–3.14 (m, 3 H), 2.91–2.75 (m, 1 H), 1.19 (t, J = 7.1 Hz, 3 H); ^{13}C NMR (100 MHz,

$CDCl_3$) δ 171.0, 140.7, 139.0, 131.0, 128.6, 127.6, 126.7, 61.8, 36.5, 32.4, 18.9, 13.7, 4.9. HRMS (ESI) calcd for $C_{15}H_{18}I_2NaO_2$ $[M+Na]^+$ 506.9288, found 506.9276.

Supplementary Table 2. Optimization of Reaction under Thermal-initiated Conditions

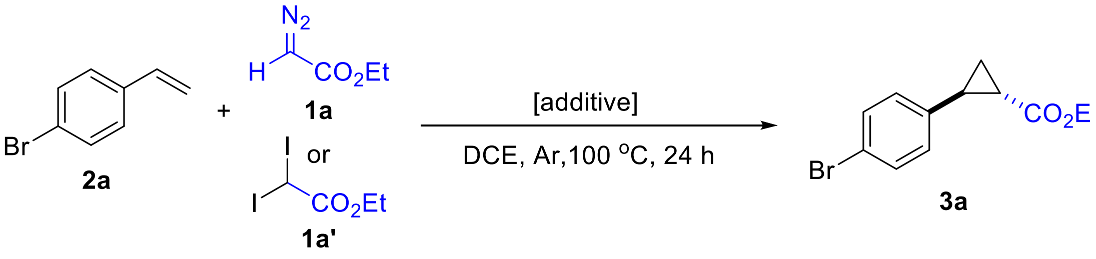

entry	reactant	additive	**2a** conv.	**3a** yield
1	**1a'**	None	0%	n.d.
2	**1a**	None	<10%	<5%
3	**1a**	10 mol% I_2	100%	98%
4	**1a**	1 equiv. of I_2	0%	n.d.
5	**1a**	10 mol% **1a'**	0%	n.d.
6	**1a**	10 mol% I_2 but 80 °C, 48 h	< 5%	< 5%
7	**1a**	Other catalysts including Br_2, KI, NIS	< 10%	< 5%

Reaction conditions: The DCE solution (3 mL) of **2a** (0.3 mmol), additive and EDA **1a** (0.3 mmol) or **1a'** (0.3 mmol) was kept under argon (degassed) at 100 °C for 24 h. Conversion and yield were determined by ^1H NMR spectroscopy using 1,3,5-trimethoxybenzene as internal standard.

Radical Clock Experiment under Thermal-initiated Conditions

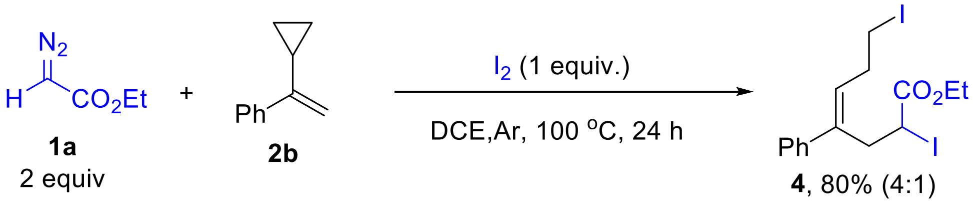

To a 50 mL Schlenk tubes with a stir bar was added olefin **1a** (43 mg, 0.3 mmol), diazo compound **1a** (35 mg, 0.3 mmol) and 3 mL of DCE, then I_2 (8 mg, 0.03 mmol) was added. The Schlenk tube was vacuumed and purged with argon three times before it was tightly screw-capped. The reaction mixture was stirred at 100 °C for 24 h, and cooled to room temperature. The reaction solution was evaporated, and the residue was purified by column chromatography (PE/EA) to afford the desired product **4** in 80% yield (E/Z = 4:1).

Radical Trapping Experiment under Thermal-initiated Conditions

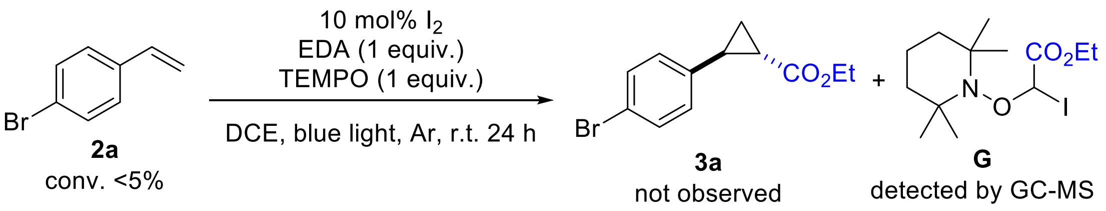

To a 50 mL Schlenk tubes with a stir bar was added olefin **2a** (55 mg, 0.3 mmol), diazo compound **1a** (35 mg, 0.3 mmol), TEMPO (47 mg, 0.3 mmol) and 3 mL of DCE, then I_2 (8 mg, 0.03 mmol) was added. The Schlenk tube was vacuumed and purged with argon three times before it was tightly screw-capped. The reaction mixture was stirred at 100 ^{o}C for 24 h, and cooled to room temperature. No **3a** was observed via the crude HNMR. And the key iodo-substituted alkyl radical intermediate was trapped by TEMPO to yield G (Figure 1). GC–MS [M+1]: 370.0, 342.0, 325.0, 297.0, 222.9.

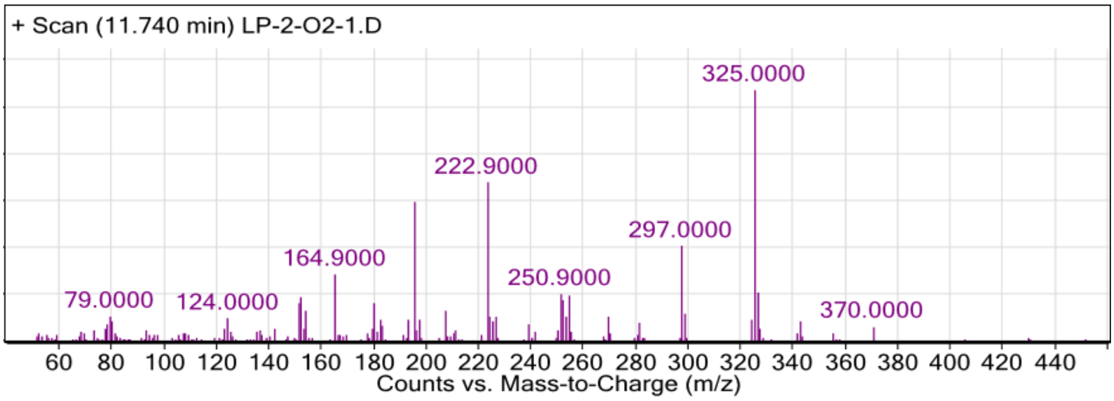

Supplementary Figure 1. The key radical intermediate G was trapped by TEMPO.

Control Experiment under Photo-initiated Conditions

Control experiment 1. Photocatalyst would deactivate in the presence of some alkenes, such as vinyl ferrocene, *p*-NO_2-styrene and enamide. Control experiments displayed that EDA would decompose slowly in the presence of I_2 and $Ru(bpy)_3Cl_2$. However, some alkenes (such as vinyl ferrocene, *p*-NO_2-styrene and enamide) added the above reaction, we don't observe decomposition of EDA (except the formation of diiodide **1a'**), which supported our hypothesis that these alkene substrates indeed deactivated the photo catalyst. Besides, the model reaction would be inhibited completely when these alkenes were added. And these results strongly indicated that photocatalyst would deactivate in the presence of these alkenes, see Supplementary Figure 2.

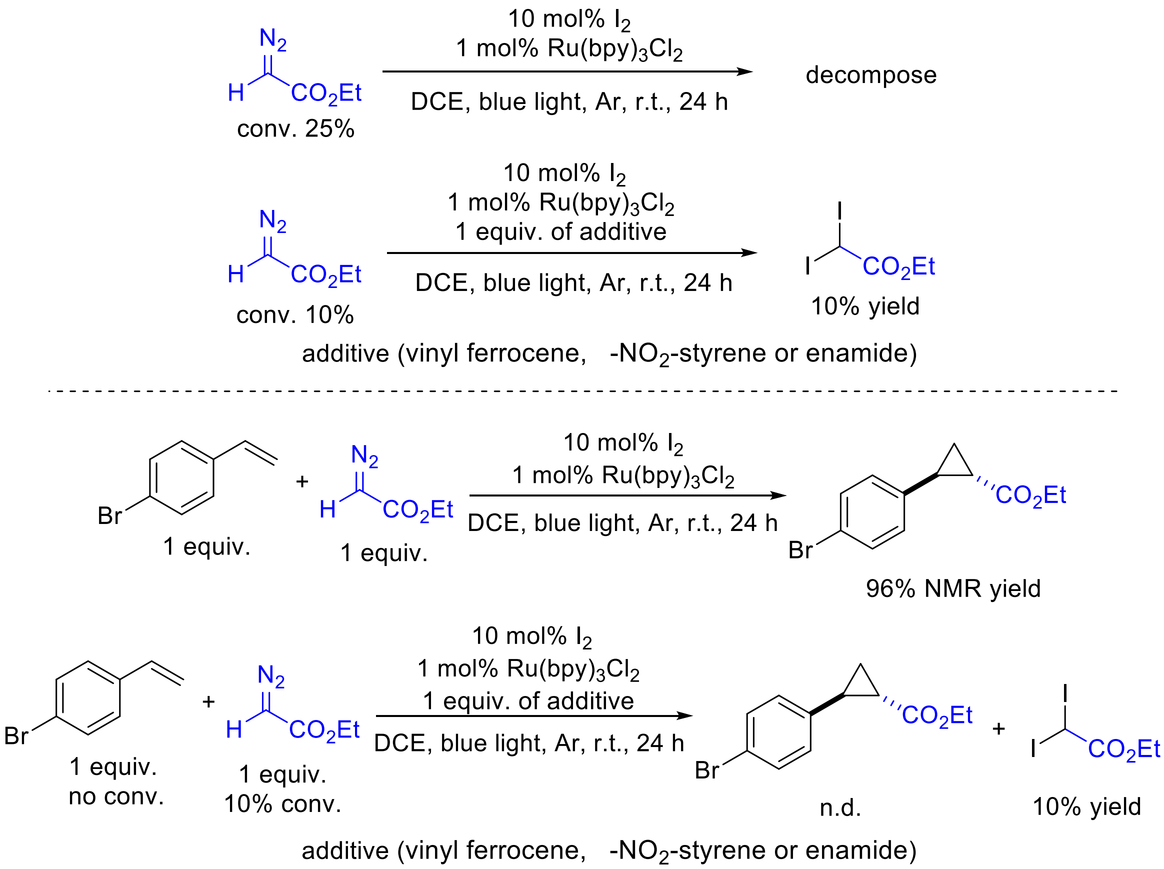

Supplementary Figure 2. Control experiment 1

Control experiment 2. Some alkenes would decompose in the presence of photocatalyst, such as vinyl ether and dienes. Significant alkene decomposition was observed owing to itself polymerization, see Supplementary Figure 3.

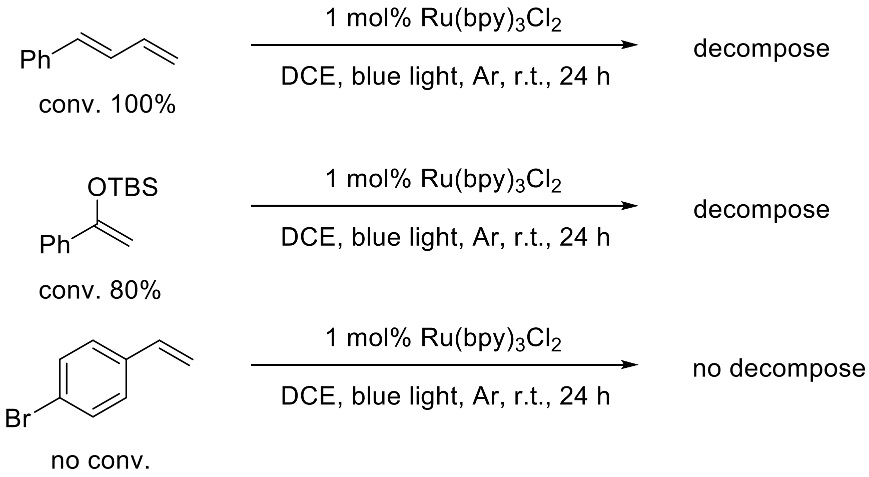

Supplementary Figure 3. Control experiment 2

Supplementary Table 3. Control Experiment for Olefin Epoxidation

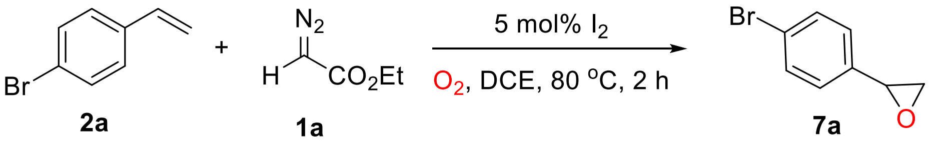

Alternation from above condition	**2a** conv.	**7a** yield
none	100%	96% (94%)
Open in the air	100%	70%
No I_2	< 5%	n.d.
0.2 equiv. of **1a** instead of 1 equiv.	20%	18%
60 °C for 12 h	< 5%	trace

Reaction conditions: 5 mol% I_2 were added to a DCE solution (3 mL) of **2a** (0.3 mmol) and EDA **1a** (0.3 mmol), and reaction was kept under O_2 (degassed) at 80 °C for 2 h. Conversion and yield were determined by ^{1}H NMR spectroscopy using 1,3,5-trimethoxybenzene as internal standard. Isolated yield in parentheses.

EPR Experiments

Catalytic amount of iodine and EDA under argon using MNP (2-methyl-2-nitrosopropane) as a spin trapping reagent. To a 50 mL Schlenk tubes with a stir bar was added EDA (23 mg, 0.2 mmol), MNP (3.5 mg, 0.04 mmol) and 2 mL of DCE. Then iodine (5 mg, 0.02 mmol) was added to the above solution. The Schlenk tube was vacuumed and purged with argon three times before it was tightly screw-capped. The mixture was stirred at 100 ºC for 1 h, and cooled to room temperature. Then 20 uL of this solution was taken out into a small tube and analyzed by EPR at room temperature (**Supplementary Figure 4**).

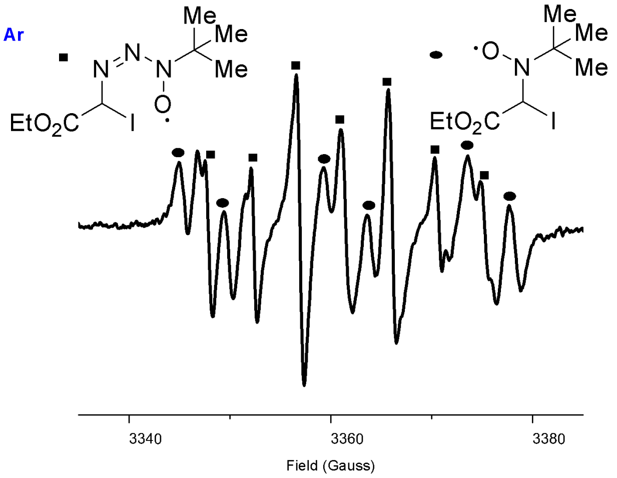

Supplementary Figure 4. EPR spectra from the reaction under argon.

Two radical signals were clearly observed. Data analysis suggested that the seven Lorentzian lines (g = 2.0066, *a* (^{14}N1) = 9.14 G, *a* (^{14}N2) = 4.56 G) displayed characteristic resonance of adduct from nitrogen radical and MNP. We believe that the nitrogen radical may be generated from iodine and EDA. The other six Lorentzian lines (g = 2.0063, *a* (^{14}N)

= 14.41 G, *a* (^{1}H) = 4.19 G) displayed a characteristic resonance of adduct from carbon radical and MNP, which we speculated was generated from denitrogenation product of the nitrogen radical.

Catalytic amount of iodine and EDA under oxygen using DMPO (5,5-dimethyl-1-pyrroline N-oxide) as a spin trapping reagent. To a 50 mL Schlenk tubes with a stir bar was added EDA (23 mg, 0.2 mmol), DMPO (4.5 mg, 0.04 mmol) and 2 mL of DCE. Then iodine (5 mg, 0.02 mmol) was added to the above solution. The Schlenk tube was vacuumed and purged with oxygen three times before it was tightly screw-capped. The mixture was stirred at 80 °C for 30 min, and cooled to room temperature. Then 20 uL of this solution was taken out into a small tube and analyzed by EPR at room temperature (**Supplementary Figure 5.**).

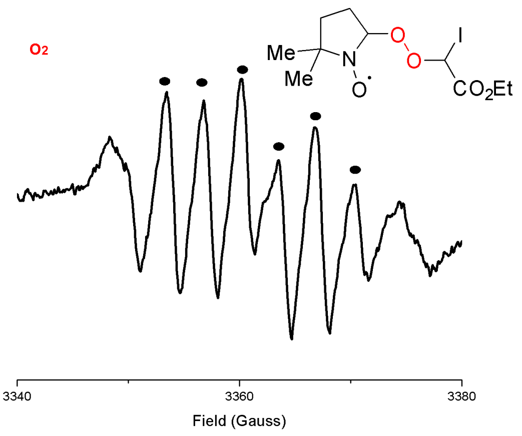

Supplementary Figure 5. EPR spectra from the reaction under oxygen using DMPO.

We observed clearly the peroxy radical signal with six Lorentzian lines. Other weak signals came form DMPO itself signal under oxygen. We speculated that the iodo-substituted alkyl radical would further react with oxygen to generate the peroxy radical which was easily captured by DMPO.

Experimental Procedure for Olefin Cyclopropanation

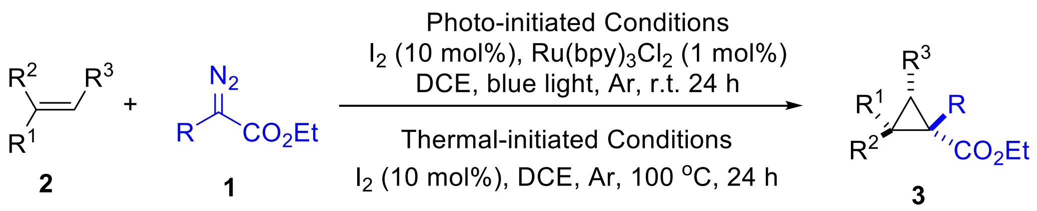

Photo-initiated Conditions: To a 50 mL Schlenk tubes with a stir bar was added olefin **2** (0.3 mmol), diazo compound **1a** (35 mg, 0.3 mmol) and 3 mL of DCE, then $Ru(bpy)_3Cl_2 \cdot 6H_2O$ (2 mg, 0.003 mmol) and I_2 (8 mg, 0.03 mmol) were added. The Schlenk tube was vacuumed and purged with argon three times before it was tightly screw-capped.

The reaction mixture was stirred at room temperature under blue light for 24 h. The reaction solution was evaporated, and the residue was purified by column chromatography (PE/EA) to afford the desired product **3a–3n**.

Thermal-initiated Conditions: To a 50 mL Schlenk tubes with a stir bar was added olefin **2** (0.3 mmol), diazo compound **1** (0.3 mmol) and 3 mL of DCE, then iodine (8 mg, 0.03 mmol) was added. The Schlenk tube was vacuumed and purged with argon three times before it was tightly screw-capped. The reaction mixture was stirred at 100 ºC for 24 h, and cooled to room temperature. The reaction solution was evaporated, and the residue was purified by column chromatography (PE/EA) to afford the desired product **3a–3x**.

The Experimental Date for Olefin Cyclopropanation

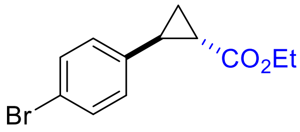

3a

Ethyl 2-(4-bromophenyl)cyclopropane-1-carboxylate **3a**: *E-isomers*: white solid, ^{1}H NMR (400 MHz, $CDCl_3$) δ 7.42–7.36 (m, 2 H), 6.99–6.93 (m, 2 H), 4.17 (q, *J* = 7.1 Hz, 2 H), 2.49–2.44 (m, 1 H), 1.87–1.85 (m, 1 H), 1.62–1.57 (m, 1 H), 1.30–1.24 (m, 4 H); ^{13}C NMR (100 MHz, $CDCl_3$) δ 173.0, 139.1, 131.5, 127.9, 120.1, 60.8, 25.5, 24.1, 16.9, 14.2. *Z-isomers*: colorless liquid, ^{1}H NMR (400 MHz, $CDCl_3$) δ 7.41–7.36 (m, 2 H), 7.14 (d, *J* = 8.3 Hz, 2 H), 3.90 (q, *J* = 7.0 Hz, 2 H), 2.50 (dd, *J* = 16.8, 8.6 Hz, 1H), 2.11–2.05 (m, 5.7 Hz, 1 H), 1.69–1.64 (m, 1 H), 1.36–1.31 (m, 1 H), 1.02 (t, *J* = 7.1 Hz, 3 H); ^{13}C NMR (100 MHz, $CDCl_3$) δ 170.7, 135.6, 131.0, 130.9, 120.5, 60.3, 24.9, 21.8, 14.1, 11.3. HRMS (ESI) calcd for $C_{12}H_{13}BrNaO_2$ $[M+Na]^+$ 290.9991, found 291.0000.

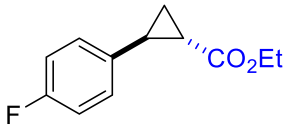

3b

Ethyl 2-(4-fluorophenyl)cyclopropane-1-carboxylate **3b**: the mixture of *E* and *Z*, colorless liquid, *E-isomers*: ^{1}H NMR (400 MHz, $CDCl_3$) δ 7.07–7.02 (m, 2 H), 6.97–6.91 (m, 2 H), 4.15 (q, *J* = 7.1 Hz, 2 H), 2.52–2.46 (m, 1 H), 1.89–1.78 (m, 1 H), 1.58–1.54 (m, 1 H), 1.31–1.24 (m, 4 H); ^{13}C NMR (100 MHz, $CDCl_3$) δ 173.2, 161.6 (d, *J* = 244.8 Hz), 135.7, 127.8 (d, *J* = 8.0 Hz, 2C), 115.2 (d, J = 21.5 Hz), 60.7, 25.4, 23.9, 16.8, 14.2. *Z-isomers*: ^{1}H NMR (400 MHz, $CDCl_3$) δ 7.22-7.19 (m, 2H), 6.97–6.90 (m, 2 H), 3.87 (q, *J* = 7.1 Hz, 2 H), 2.54–2.47 (m, 1 H), 2.07-2.01 (m, 1 H), 1.67–1.61 (m, 1 H), 1.34-1.31 (m, 1 H), 0.99 (t, *J* = 7.1 Hz, 3 H); ^{13}C NMR (100 MHz, $CDCl_3$) δ 170.8, 161.6 (d, *J* = 244.5 Hz), 132.2, 130.7 (d, *J* = 8.0 Hz), 114.6 (d, *J* = 21.3 Hz), 60.1, 24.6, 21.6, 14.0, 11.2. HRMS (ESI) calcd for $C_{12}H_{13}FNaO_2$ $[M+Na]^+$ 231.0792, found 237.0785.

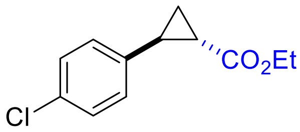

3c

***Ethyl 2-(4-chlorophenyl)cyclopropane-1-carboxylate* 3c**: *E-isomers*: white solid, ^{1}H NMR (400 MHz, $CDCl_3$) δ 7.24 (d, *J* = 8.4 Hz, 2 H), 7.03 (d, *J* = 8.4 Hz, 2 H), 4.17 (q, *J* = 7.1 Hz, 2 H), 2.54–2.42 (m, 1 H), 1.95–1.81 (m, 1 H), 1.65–1.53 (m, 1 H), 1.35–1.22 (m, 4 H); ^{13}C NMR (100 MHz, $CDCl_3$) δ 173.1, 138.6, 132.2, 128.5, 127.6, 60.8, 25.5, 24.2, 17.0, 14.2. *Z-isomers*: colorless liquid, ^{1}H NMR (400 MHz, $CDCl_3$) δ 7.25–7.13 (m, 4 H), 3.88 (q, *J* = 7.1 Hz, 2 H), 2.53-2.46 (m, 1 H), 2.08-2.02 (m, 1H), 1.67–1.62 (m, 1H), 1.34–1.27 (m, 1H), 1.00 (t, *J* = 7.1 Hz, 3 H); ^{13}C NMR (100 MHz, $CDCl_3$) δ 172.5, 170.8, 135.1, 130.6, 128.0, 60.3, 24.8, 21.8, 14.1, 11.3. HRMS (ESI) calcd for $C_{12}H_{14}ClO_2[M+H]^+$ 225.0677, found 225.0666.

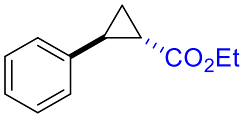

3d

***Ethyl 2-phenylcyclopropane-1-carboxylate* 3d**: *E-isomers*: white solid,^{1}H NMR (400 MHz, $CDCl_3$) δ 7.29–7.25 (m, 2 H), 7.21–7.19 (m, 1 H), 7.10–7.08 (m, 2 H), 4.17 (q, *J* = 7.1 Hz, 2 H), 2.54–2.49 (m, 1H), 1.92–1.88 (m, 1H), 1.62–1.58 (m, 1 H), 1.33–1.30 (m, 1 H), 1.27 (t, *J* = 7.1 Hz, 3 H); ^{13}C NMR (100 MHz, $CDCl_3$) δ 173.4, 140.1, 128.4, 126.4, 126.1, 60.7, 26.1, 24.1, 17.0, 14.2. HRMS (ESI) calcd for $C_{12}H_{15}O_2$ $[M+H]^+$ 191.1072, found 191.1071.

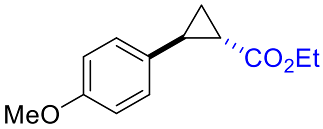

3e

***Ethyl 2-(4-methoxyphenyl)cyclopropane-1-carboxylate* 3e**: *E-isomers*: white solid, ^{1}H NMR (400 MHz, $CDCl_3$) δ 7.04 (d, *J* = 8.6 Hz, 2 H), 6.82 (d, *J* = 8.7 Hz, 2 H), 4.17 (q, *J* = 7.1 Hz, 2 H), 3.78 (s, 3 H), 2.51–2.46 (m, 1 H), 1.85–1.81 (m, 1 H), 1.58–1.53 (m, 1 H), 1.30–1.23 (m, 4 H); ^{13}C NMR (100 MHz, $CDCl_3$) δ 173.5, 158.3, 132.0, 127.3, 113.9, 60.6, 55.3, 25.6, 23.8, 16.7, 14.2. *Z-isomers*: colorless liquid, ^{1}H NMR (400 MHz, $CDCl_3$) δ 7.18 (d, *J* = 8.5 Hz, 2 H), 6.80 (d, *J* = 8.7 Hz, 2 H), 3.89 (q, *J* = 7.1 Hz, 2 H), 3.77 (s, 3 H), 2.52 (dd, *J* = 16.7, 8.6 Hz, 1 H), 2.06–2.00 (m, 1 H), 1.68–1.63 (m, 1 H), 1.32–1.27 (m, 1 H), 1.02 (t, *J* = 7.1 Hz, 3 H); ^{13}C NMR (100 MHz, $CDCl_3$) δ 171.1, 158.3 130.2, 128.5, 113.3, 60.1, 55.2, 24.8, 21.7, 14.1, 11.2. HRMS (ESI) calcd for $C_{13}H_{17}O_3$ $[M+H]^+$ 221.1172, found 221.1171.

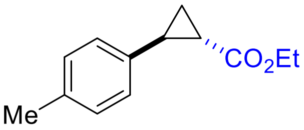

3f

***Ethyl 2-(p-tolyl)cyclopropane-1-carboxylate* 3f**: *E-isomers*: colorless liquid, ^{1}H NMR (400 MHz, $CDCl_3$) δ 7.09 (d, *J* = 7.9 Hz, 2 H), 7.00 (d, *J* = 8.0 Hz, 2 H), 4.17 (q, *J* = 7.1 Hz, 2 H), 2.54–2.43 (m, 1 H), 2.32 (s, 3 H), 1.88–1.84 (m, 1 H), 1.63–1.52 (m, 1 H), 1.33–1.23 (m, 4 H); ^{13}C NMR (100 MHz, $CDCl_3$) δ 173.5, 137.0, 136.1, 129.1, 126.1, 60.6, 25.9, 24.0, 21.0, 16.9, 14.3. *Z-isomers*: colorless liquid, ^{1}H NMR (400 MHz, $CDCl_3$) δ 7.15 (d, *J* = 8.0 Hz, 2 H), 7.06 (d, *J* = 8.0 Hz, 2 H), 3.89 (q, *J* = 7.1 Hz, 2 H), 2.55–2.53 (m, 1 H), 2.30 (s, 1 H), 2.05–2.04 (m, 1H), 1.70–1.65 (m, 1 H), 1.36–1.24 (m, 2 H), 1.01 (t, *J* = 7.1 Hz, 3 H); ^{13}C NMR (100 MHz, $CDCl_3$ δ 171.1, 136.1, 133.4, 129.1, 128.6, 60.2, 25.2, 21.7, 21.1, 14.1, 11.2. HRMS (ESI) calcd for $C_{13}H_{16}NaO_2$ $[M+Na]^+$ 227.1043, found 227.1038.

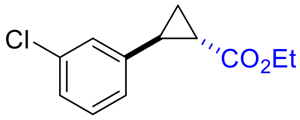

3g

***Ethyl 2-(3-chlorophenyl)cyclopropane-1-carboxylate* 3g*: E-isomers*:** colorless liquid, ^{1}H NMR (400 MHz, $CDCl_3$) δ 7.23–7.14 (m, 2 H), 7.08–7.05 (s, 1 H), 6.99–6.97 (m, 1 H), 4.17 (q, J = 7.1 Hz, 2 H), 2.56–2.37 (m, 1 H), 1.95–1.84 (m, 1 H), 1.64–1.52 (m, 1 H), 1.32–1.26 (m, 1 H), 1.28 (t, *J* = 7.1 Hz, 3 H); ^{13}C NMR (100 MHz, $CDCl_3$) δ 173.0, 142.2, 134.3, 129.6, 126.6, 126.3, 124.5, 60.8, 25.6, 24.2, 17.0, 14.2. *Z-isomers*: colorless liquid, ^{1}H NMR (400 MHz, $CDCl_3$) δ 7.32–7.29 (m, 1 H), 7.2–7.16 (m, 3 H), 3.95 (q, *J* = 7.1 Hz, 2 H), 2.61–2.55(m, 1 H), 2.22–2.09 (m, 1 H), 1.75–1.71 (m, 1 H), 1.41–1.37 (m, 1 H), 1.06 (t, *J* = 7.1 Hz, 3 H); ^{13}C NMR (100 MHz, $CDCl_3$) δ 170.7, 138.7, 133.6, 129.5, 129.1, 127.5, 126.8, 60.3, 24.9, 21.8, 14.0, 11.2. HRMS (ESI) calcd for $C_{12}H_{13}ClO_2$ $[M+H]^+$ 225.0604, found 225.0676.

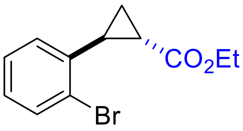

3h

***Ethyl 2-(2-bromophenyl)cyclopropane-1-carboxylate* 3h**: *E-isomers*: colorless liquid, ^{1}H NMR (400 MHz, $CDCl_3$) δ 7.59 (d, *J* = 7.9 Hz, 1 H), 7.29–7.24 (m, 1 H), 7.12 (t, *J* = 7.4 Hz, 1 H), 7.05 (d, *J* = 7.6 Hz, 1 H), 4.34–4.15 (m, 2 H), 2.76–2.71 (s, 1 H), 1.83–1.80 (m, 1 H), 1.69–1.63 (m, 1 H), 1.38–1.31 (m, 4 H); ^{13}C NMR (100 MHz, $CDCl_3$) δ 173.3, 139.0, 132.6, 128.2, 127.5, 127.3, 126.3, 60.7, 27.0, 23.1, 15.7, 14.3. *Z-isomers*: colorless liquid, ^{1}H NMR (400 MHz, $CDCl_3$) δ 7.47 (d, *J* = 7.9 Hz, 1 H), 7.25–7.22 (m, 2 H), 7.08–7.06 (m, 1 H), 3.88 (q, *J* = 7.4 Hz, 2 H), 2.51–2.49 (m, 1 H), 2.20–2.18 (m, 1 H), 1.71–1.61 (m, 1 H), 1.44–1.33 (m, 1 H), 0.98 (t, *J* = 7.1 Hz, 3 H); ^{13}C NMR (100 MHz, $CDCl_3$) δ 171.1, 136.5, 132.1, 131.1, 128.3, 126.8, 126.4, 60.3, 27.0, 21.7, 14.0, 12.4. HRMS (ESI) calcd for C12H13BrO2 [M+H]+ 269.0172, found 269.0171.

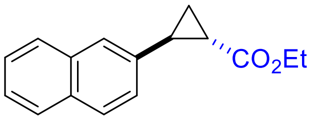

3i

***Ethyl 2-(naphthalen-2-yl)cyclopropane-1-carboxylate* 3i**: *E-isomers*: white solid, ^{1}H NMR (400 MHz, $CDCl_3$) δ 7.83–7.80 (m, 1 H), 7.78 (d, *J* = 8.2 Hz, 2 H), 7.59 (s, 1 H), 7.51–7.41 (m, 2 H), 7.22 (dd, *J* = 8.5, 1.6 Hz, 1 H), 4.23 (q, *J* = 7.1 Hz, 2 H), 2.77–2.66 (m, 1 H), 2.07–1.98 (m, 1 H), 1.77–1.64 (m, 1 H), 1.54–1.40 (m, 1 H), 1.32 (t, *J* = 7.1 Hz, 3 H); ^{13}C NMR (100 MHz, $CDCl_3$) δ 173.3, 137.5, 133.3, 132.2, 128.1, 127.6, 127.3, 126.2, 125.4, 124.7, 124.5, 60.7, 26.4, 24.1, 17.0, 14.2. *Z-isomers*: colorless liquid, ^{1}H NMR (400 MHz, $CDCl_3$) δ 7.81–7.71 (m, 4H), 7.48–7.38 (m, 3 H), 3.83 (q, *J* = 7.1 Hz, 2 H), 2.73 (dd, *J* = 16.9, 8.5 Hz, 1 H), 2.20–2.12 (m, 1 H), 1.88–1.83 (m, 1 H), 1.47–1.37 (m, 1 H), 0.91 (t, *J* = 7.1 Hz, 3 H); ^{13}C NMR (100 MHz, $CDCl_3$) δ 170.9, 134.1, 133.2, 132.4, 127.9, 127.63 (two peaks overlap), 127.55, 127.4, 125.8, 125.4, 60.2, 25.7, 22.0, 14.0, 11.3. HRMS (ESI) calcd for $C_{16}H_{17}O_2$ $[M+H]^+$ 241.1223, found 241.1223.

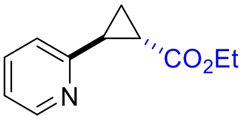

3j

***Ethyl 2-(pyridin-2-yl)cyclopropane-1-carboxylate* 3j**: *E-isomers*: colorless liquid, ^{1}H NMR (400 MHz, $CDCl_3$) δ 8.43 (d, *J* = 4.3 Hz, 1 H), 7.55 (td, *J* = 7.7, 1.6 Hz, 1 H), 7.21 (d, *J* = 7.8 Hz, 1 H), 7.07 (dd, *J* = 7.0, 5.2 Hz, 1 H), 4.16 (q, *J* = 7.1 Hz, 2 H), 2.63–2.49 (m, 1 H), 2.3–2.15 (m, 1 H), 1.64-1.56 (m, 1 H), 1.31–1.23 (m, 4 H); ^{13}C NMR

(100 MHz, $CDCl_3$) δ 173.4, 158.9, 149.4, 136.0, 122.5, 121.3, 60.7, 27.2, 24.3, 17.3, 14.2. *Z-isomers*: colorless liquid, 1H NMR (400 MHz, $CDCl_3$) δ 8.49 (d, *J* = 4.3 Hz, 1 H), 7.59 (td, *J* = 7.7, 1.6 Hz, 1 H), 7.27–7.23 (m, 1 H), 7.11 (dd, *J* = 7.0, 5.2 Hz, 1 H), 3.90 (q, *J* = 7.1 Hz, 2 H), 2.75–2.69 (m, 1 H), 2.21–2.09 (m, 1 H), 1.87–1.81 (m, 1 H), 1.44–1.39 (m, 1 H), 1.02 (t, *J* = 7.1 Hz, 3 H); ^{13}C NMR (100 MHz, $CDCl_3$) δ 171.1, 156.7, 148.9, 135.9, 123.6, 121.6, 60.31, 27.18, 21.7, 14.0, 11.6. HRMS (ESI) calcd for $C_{11}H_{14}NO_2[M+H]^+$ 192.1019, found 192.1016.

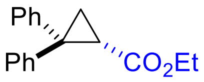

3k

***Ethyl 2,2-diphenylcyclopropane-1-carboxylate* 3k**: colorless liquid, ^{1}H NMR (400 MHz, $CDCl_3$) δ 7.35 (dd, *J* = 5.2, 3.3 Hz, 1H), 7.28–7.14 (m, 8 H), 3.98–3.84 (m, 1 H), 2.54 (dd, *J* = 8.1, 5.9 Hz, 1 H), 2.17 (dd, *J* = 5.8, 4.9 Hz, 1 H), 1.58 (dd, *J* = 8.1, 4.8 Hz, 1 H), 1.00 (t, *J* = 7.1 Hz, 3 H); ^{13}C NMR (100 MHz, $CDCl_3$) δ 170.6, 144.9, 140.2, 129.7, 128.4, 128.2, 127.6, 126.9, 126.5, 60.4, 39.8, 29.0, 20.1, 14.0; HRMS (ESI) calcd for $C_{18}H_{18}NaO_2$ $[M+Na]^+$ 289.1199, found 289.1209.

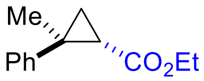

3l

***Ethyl 2-methyl-2-phenylcyclopropane-1-carboxylate* 3l**: *E-isomers*: colorless liquid, ^{1}H NMR (400 MHz, $CDCl_3$) δ 7.31–7.30 (m, 4 H), 7.23–7.20 (m, 1 H), 4.31–4.11 (m, 2 H), 1.99–1.95 (m, 1 H), 1.53 (s, 3 H), 1.46–1.39 (m, 1 H), 1.31 (t, *J* = 7.1 Hz, 3 H); ^{13}C NMR (100 MHz, $CDCl_3$) δ 172.2, 145.9, 128.4, 127.3, 126.4, 60.5, 30.6, 27.9, 20.8, 19.9, 14.4. *Z-isomers*: colorless liquid, ^{1}H NMR (400 MHz, $CDCl_3$) δ 7.25–7.24 (m, 4 H), 7.19–7.16 (m, 1 H), 3.83–3.78 (m, 2 H), 1.90–1.86 (m, 1H), 1.77–1.75 (m, 1 H), 1.44 (s, 3 H), 1.14–1.11 (m, 1 H), 0.92 (t, *J* = 7.1 Hz, 3 H); ^{13}C NMR (100 MHz, $CDCl_3$) δ 171.2, 141.9, 128.7, 128.1, 126.6, 60.0, 32.0, 28.5, 19.4, 13.9. HRMS (ESI) calcd for $C_{13}H_{17}O_2$ $[M+H]^+$ 205.1223, found 205.1216.

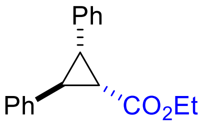

3m

***Diethyl 3-phenylcyclopropane-1,2-dicarboxylate* 3m**: colorless liquid, ^{1}H NMR (400 MHz, $CDCl_3$) δ 7.45–7.28 (m, 10 H), 4.03 (q, *J* = 7.1, 2 H), 3.32–3.29 (m, 1 H), 3.05–2.97 (m, 1 H), 2.52–2.44 (m, 1 H), 1.12 (t, *J* = 7.1 Hz, 3 H); ^{13}C NMR (100 MHz, $CDCl_3$) δ 169.9, 139.5, 136.1, 129.1, 128.6, 128.0, 126.9, 126.62, 126.58, 60.4, 34.4, 31.2, 29.2, 14.0. HRMS (ESI) calcd for $C_{18}H_{19}O_2$ $[M+H]^+$ 267.1385, found 267.1380.

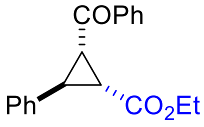

3n

***Ethyl 2-benzoyl-3-phenylcyclopropane-1-carboxylate* 3n**: *E-isomers*: white solid, ^{1}H NMR (400 MHz, $CDCl_3$) δ 8.08–8.03 (m, 2 H), 7.62–7.58 (m, 1 H), 7.52–7.45 (m, 2 H), 7.40–7.34 (m, 2 H), 7.32–7.23 (m, 3 H), 4.12 (q, *J* = 7.1 Hz, 2 H), 3.41–3.38 (m, 1H), 3.14–3.10 (m, 1H), 2.69–2.65 (m, 1 H), 1.17 (t, *J* = 7.1 Hz, 3 H); ^{13}C NMR (100 MHz, $CDCl_3$) δ 193.7, 169.1, 138.1, 136.9, 133.3, 128.7, 128.6, 128.4, 127.1, 126.6, 61.1, 35.1, 31.6, 29.7, 14.0. *Z-isomers*: colorless liquid, ^{1}H NMR (400 MHz, $CDCl_3$) δ 8.13–8.10 (m, 2 H), 7.65–7.58 (m, 1 H), 7.55–7.51 (m, 2 H), 7.34–7.25 (m, 5 H), 3.99 (q, *J* = 7.1, 2 H), 3.87–3.84 (m, 1 H), 3.28–3.24 (m, 1 H), 2.87–2.84 (m, 1 H), 1.07 (t, *J* = 7.1 Hz, 3 H); ^{13}C NMR (100 MHz, $CDCl_3$) δ 196.7, 168.7, 137.0, 134.8, 133.5, 128.9, 128.8, 128.3, 128.2, 127.3,

60.9, 35.0, 32.2, 29.6, 14.0. HRMS (ESI) calcd for $C_{19}H_{19}O_3$ $[M+H]^+$ 295.1329, found 295.1318.

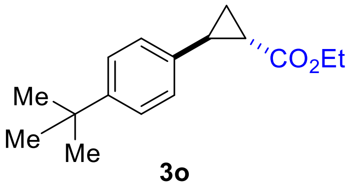

3o

***Ethyl 2-(4-(tert-butyl)phenyl)cyclopropane-1-carboxylate* 3o**: *E-isomers*: white solid, ^1H NMR (400 MHz, $CDCl_3$) δ 7.32 (d, *J* = 7.3 Hz, 2 H), 7.05 (d, *J* = 7.5 Hz, 2 H), 4.17 (q, *J* = 7.1 Hz, 2 H), 2.52-2.48 (m, 1 H), 1.90-1.87 (m, 1H), 1.64 – 1.58 (m, 1 H), 1.37–1.22 (m, 13 H); ^{13}C NMR (100 MHz, $CDCl_3$) δ 173.5, 149.4, 137.1, 125.8, 125.4, 60.6, 34.4, 31.3, 25.8, 24.1, 16.9, 14.3. *Z-isomers*: colorless liquid, ^1H NMR (400 MHz, $CDCl_3$) δ 7.34–7.28 (m, 2 H), 7.23 (d, *J* = 8.5 Hz, 2 H), 3.97–3.84 (m, 2 H), 2.61–2.55 (m, 1 H), 2.11–2.09 (m, 1 H), 1.74–1.72 (d, *J* = 7.4 Hz, 1H), 1.36–1.32 (m, 10 H), 0.96 (t, *J* = 7.1 Hz, 3 H); ^{13}C NMR (100 MHz, $CDCl_3$) δ 171.1, 149.4, 133.5, 128.9, 124.8, 60.1, 34.4, 31.3, 25.1, 21.8, 13.9, 11.1. HRMS (ESI) calcd for $C_{16}H_{23}O_2[M+H]^+$ 247.1699, found 247.1694.

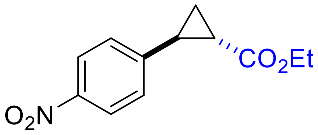

3p

***Ethyl 2-(4-nitrophenyl)cyclopropane-1-carboxylate* 3p**: the mixture of *E* and *Z*, colorless liquid, *E-isomers*: ^1H NMR (400 MHz, $CDCl_3$) δ 8.15-8.13 (m, 2 H), 7.22 (d, *J* = 8.7 Hz, 2 H), 4.19 (q, *J* = 7.1 Hz, 2 H), 2.63–2.59 (m, 1H), 2.02–1.98 (m, 1 H), 1.76–1.70 (m, 1 H), 1.40–1.35 (m, 1 H), 1.29 (t, *J* = 7.1 Hz, 3 H); ^{13}C NMR (100 MHz, $CDCl_3$) δ 172.4, 148.1, 146.5, 126.7, 123.7, 61.0, 25.6, 25.1, 17.8, 14.2. *Z-isomers*: ^1H NMR (400 MHz, $CDCl_3$) δ 8.14–8.12 (m, 2 H), 7.43 (d, *J* = 8.6 Hz, 2 H), 3.91 (q, *J* = 7.1 Hz, 2 H), 2.64–2.60 (m, 1 H), 2.25–2.15 (m, 1 H), 1.79–1.76 (m, 1 H), 1.49–1.43 (m, 1 H), 1.04 (t, *J* = 7.1 Hz, 3 H); ^{13}C NMR (100 MHz, $CDCl_3$) δ170.3, 146.7, 144.5, 130.1, 123.0, 60.5, 25.1, 22.4, 14.1, 11.8. HRMS (ESI) calcd for $C_{12}H_{14}NO_4[M+H]^+$ 236.0917, found 236.0905.

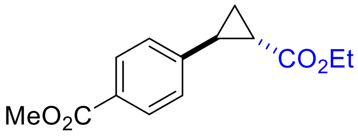

3q

***Methyl 2-(ethoxycarbonyl)cyclopropyl)benzoate* 3q**: *E-isomers*: colorless liquid, ^1H NMR (400 MHz, $CDCl_3$) *δ* 7.94 (d, *J* = 8.3 Hz, 2 H), 7.14 (d, *J* = 8.3 Hz, 2 H), 4.17 (q, *J* = 7.1 Hz, 2 H), 3.90 (s, 3 H), 2.63–2.42 (m, 1 H), 1.99–1.89 (m, 1 H), 1.69–1.62 (m, 1 H), 1.37–1.32 (m, 1 H), 1.28 (t, *J* = 7.1 Hz, 3 H); ^{13}C NMR (100 MHz, $CDCl_3$) δ 172.9, 166.8, 145.7, 129.8, 128.3, 126.0, 60.9, 52.0, 26.0, 24.7, 17.5, 14.2. *Z-isomers*: colorless liquid, ^1H NMR (400 MHz, $CDCl_3$) δ 7.93 (d, *J* = 8.3 Hz, 2 H), 7.32 (d, *J* = 8.2 Hz, 2 H), 3.88 (s, 3 H), 3.87 (q, *J* = 7.1 Hz, 2 H), 2.63–2.54 (m, 1 H), 2.19–2.06 (m, 1 H), 1.76–1.71 (m, 1 H), 1.39–1.32 (m, 1 H), 0.97 (t, *J* = 7.1 Hz, 3 H); ^{13}C NMR (100 MHz, $CDCl_3$) δ 170.5, 166.9, 142.0, 129.3, 129.1, 128.4, 60.3, 52.0, 25.3, 22.1, 14.0, 11.3. HRMS (ESI) calcd for $C_{14}H_{17}O_4[M+H]^+$ 249.1121, found 249.1125.

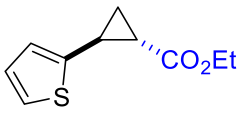

3r

Ethyl 2-(thiophen-2-yl)cyclopropane-1-carboxylate **3r**: *E-isomers*: colorless liquid, ^{1}H NMR (400 MHz, $CDCl_3$) δ 7.09 (d, *J* = 5.0 Hz, 1 H), 6.92–6.88 (m, 1 H), 6.84–6.80 (m, 1 H), 4.17 (q, *J* = 7.1 Hz, 2 H), 2.70–2.69 (m, 1 H), 2.01–1.88 (m, 1 H), 1.67–1.58 (m, 1 H), 1.36–1.18 (m, 4 H); ^{13}C NMR (100 MHz, $CDCl_3$) δ 172.8, 144.0, 126.9, 123.9, 123.1, 60.8, 25.0, 21.5, 17.9, 14.3. *Z-isomers*: colorless liquid, ^{1}H NMR (400 MHz, $CDCl_3$) δ 7.12 (d, *J* = 4.3 Hz, 1 H), 6.92–6.86 (m, 2 H), 3.96 (q, *J* = 7.1 Hz, 2 H), 2.63–2.57 (m, 1 H), 2.13-2.05 (m, 1 H), 1.73–1.69 (m, 1 H), 1.44–1.39 (m, 1 H), 1.07 (t, *J* = 7.1 Hz, 3 H); ^{13}C NMR (100 MHz, $CDCl_3$) δ 170.4, 140.3, 126.5, 126.3, 124.1, 60.41, 22.60, 19.6, 14.0, 13.0. HRMS (ESI) calcd for $C_{10}H_{13}O_2S$ $[M+H]^+$ 197.0636, found 197.0632.

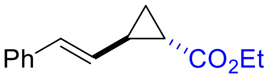

3s

Ethyl 2-((E)-styryl)cyclopropane-1-carboxylate **3s**: *E-isomers*: white solid, ^{1}H NMR (400 MHz, $CDCl_3$) δ 7.34–7.26 (m, 4 H), 7.24–7.18 (m, 1 H), 6.54 (d, *J* = 15.8 Hz, 1 H), 5.75 (dd, *J* = 15.8, 8.7 Hz, 1 H), 4.16 (q, *J* = 7.1 Hz, 2 H), 2.23–2.13 (m, 1 H), 1.80–1.71 (m, 1 H), 1.52–1.45 (m, 1 H), 1.28 (t, *J* = 7.1 Hz, 3 H), 1.14–1.06 (m, 1 H); ^{13}C NMR (100 MHz, $CDCl_3$) δ 173.3, 137.0, 130.3, 130.0, 128.5, 127.2, 125.8, 60.6, 25.5, 22.3, 15.9, 14.3. *Z-isomers*: colorless liquid, ^{1}H NMR (400 MHz, $CDCl_3$) δ 7.38–7.31 (m, 2 H), 7.29–7.26 (m, 2 H), 7.20–7.16 (m, 1 H), 6.58 (d, *J* = 15.9 Hz, 1 H), 6.23 (dd, *J* = 15.9, 9.3 Hz, 1 H), 4.21–4.08 (m, 2 H), 2.17–1.92 (m, 2 H), 1.45–1.29 (m, 2 H), 1.26 (t, *J* = 7.1 Hz, 3 H); ^{13}C NMR (100 MHz, CDCl3) δ 172.0, 137.3, 131.3, 128.5, 127.5, 127.0, 125.9, 60.6, 24.6, 21.4, 14.7, 14.3. HRMS (ESI) calcd for $C_{14}H_{16}NaO_2$ $[M+Na]^+$ 239.1043, found 229.1050.

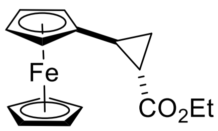

3t

Ethyl 2-(ferrocene)cyclopropane-1-carboxylate **3t**: *E-isomers*: ^{1}H NMR (400 MHz, $CDCl_3$) δ 4.22–4.01 (m, 11 H), 2.22–2.10 (m, 1 H), 1.76–1.73 (m, 1 H), 1.52–1.47 (m, 1 H), 1.28 (t, *J* = 7.1 Hz, 1H), 1.13–1.08 (m, 1 H); ^{13}C NMR (100 MHz, CDCl3) δ 173.5, 88.2, 69.2, 68.6, 67.4, 67.1, 66.63, 66.55, 60.6, 24.6, 22.2, 17.8, 14.3. *Z-isomers*: ^{1}H NMR (400 MHz, $CDCl_3$) δ 4.23–4.19 (m, 1 H), 4.15–4.11 (m, 5 H), 4.07–4.01 (m, 2 H), 3.98–3.92 (m, 2 H), 2.32–2.26 (m, 1 H), 1.94–1.88 (m, 1H), 1.43–1.39 (m, 1 H), 1.30–1.25 (m, 1H), 1.09 (t, *J* = 7.1 Hz, 2 H); ^{13}C NMR (100 MHz, $CDCl_3$) δ 171.2, 122.2, 68.93, 68.88, 68.6, 67.7, 66.8, 60.2, 23.1, 21.7, 14.2, 12.5. HRMS (ESI) calcd for $C_{16}H_{18}FeO_2$ $[M]^+$ 298.0656, found 298.0652.

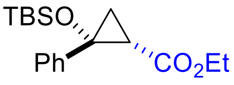

3u

Ethyl 2-((tert-butyldimethylsilyl)oxy)-2-phenylcyclopropane-1-carboxylate **3u**: Mixture of *two isomers*, colorless liquid, *E-isomers*: ^{1}H NMR (400 MHz, $CDCl_3$) δ 7.40–7.36 (m, 2 H), 7.31–7.21 (m, 3 H), 4.20–4.13 (m, 2 H), 1.92–1.88 (s, 1 H), 1.83–1.78 (m, 1 H), 1.72–1.67 (m, 1 H), 1.27 (t, *J* = 7.1 Hz, 3 H), 0.82 (s, 9 H), 0.04 (s, 3 H), -0.22 (s, 3 H); ^{13}C NMR (100 MHz, $CDCl_3$) δ 169.0, 142.5, 128.2, 127.9, 127.4, 63.5, 60.5, 30.9, 25.6, 18.4, 17.9, 14.4, -4.0. *Z-isomers*: ^{1}H NMR (400 MHz, $CDCl_3$) δ 7.40–7.36 (m, 2 H), 7.31–7.21 (m, 3 H), 3.82

(q, *J* = 7.1 Hz, 2 H), 2.26–2.19 (m, 1 H), 1.98–1.91 (m, 1H), 1.51–1.43 (m, 1 H), 0.92 (t, *J* = 7.1 Hz, 3 H), 0.78 (s, 9 H), -0.03 (s, 3 H), -0.28 (s, 3 H); ^{13}C NMR (100 MHz, CDCl$_3$) δ 170.2, 138.2, 128.8, 127.8, 127.4, 126.6, 65.5, 60.1, 30.4, 25.5, 19.4, 17.6, 14.0, -4.1. HRMS (ESI) calcd for $C_{18}H_{29}O_3Si$ [M+H]$^+$ 321.1880, found 321.1880.

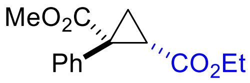

3v

***2-Ethyl 1-methyl 1-phenylcyclopropane-1,2-dicarboxylate* 3v**: Mixture of *two isomers*, colorless liquid, ^{1}H NMR (400 MHz, CDCl$_3$) δ 7.50–7.21 (m, 10.5 H), 4.21–4.15 (m, 2.2 H), 3.88–3.81 (m, 2 H), 3.63 (m, 6.3 H), 2.74–2.71 (m, 1 H), 2.26–2.20 (m, 1 H), 2.11–2.08 (m, 1.1 H), 2.02–1.99 (m, 1.1 H), 1.87–1.84 (m, 1 H), 1.52–1.49 (m, 1.1 H), 1.28 (t, *J* = 7.1 Hz, 3.3 H), 0.95 (t, *J* = 7.1 Hz, 3 H); ^{13}C NMR (100 MHz, CDCl$_3$) δ 172.7, 170.6, 170.1, 168.8, 138.0, 134.6, 130.5, 129.1, 128.5, 128.0, 127.9, 127.7, 61.0, 60.7, 52.8, 52.5, 38.8, 36.5, 29.7, 27.8, 19.3, 18.9, 14.1, 13.8. HRMS (ESI) calcd for $C_{14}H_{17}O_4$ [M+Na]$^+$ 271.0941, found 271.0938.

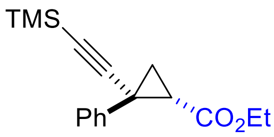

3w

***Ethyl 2-phenyl-2-((trimethylsilyl)ethynyl)cyclopropane-1-carboxylate* 3w**: Mixture of two isomers, colorless liquid, *E-isomers*: ^{1}H NMR (400 MHz, CDCl$_3$) δ 7.40–7.17 (m, 5 H), 4.23–4.16 (m, 2 H), 2.24–2.21 (m, 1 H), 2.06–2.04 (m, 1 H), 1.68–1.64 (m, 1 H), 1.29 (t, *J* = 7.1 Hz, 3 H), 0.15 (s, 9 H); ^{13}C NMR (100 MHz, CDCl$_3$) δ 168.9, 139.9, 128.4, 128.1, 126.0, 104.5, 86.2, 60.9, 33.5, 25.4, 22.9, 14.5, 0.03. *Z-isomers*: ^{1}H NMR (400 MHz, CDCl$_3$) δ 7.40–7.17 (m, 5 H), 3.93–3.67 (m, 2 H), 2.46–2.43 (m, 1 H), 2.10–2.07 (m, 1 H), 1.71–1.63 (m, 1 H), 0.92 (t, *J* = 7.1 Hz, 3 H), 0.10 (s, 9 H); ^{13}C NMR (100 MHz, CDCl$_3$) δ 168.8, 136.3, 129.2, 127.4, 126.9, 109.2, 82.0, 60.6, 31.8, 26.4, 20.0, 13.90, 0.03. HRMS (ESI) calcd for $C_{17}H_{23}O_2Si$ [M+H]$^+$ 287.1462, found 287.1459.

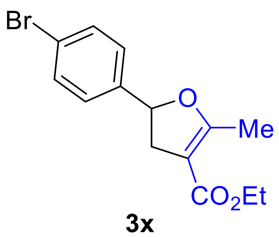

3x

***Ethyl 5-(4-bromophenyl)-2-methyl-4,5-dihydrofuran-3-carboxylate* 3x**: colorless liquid, ^{1}H NMR (400 MHz, CDCl$_3$) δ 7.49 (d, *J* = 8.4 Hz, 2 H), 7.20 (d, *J* = 8.4 Hz, 2 H), 5.53 (dd, *J* = 10.6, 8.3 Hz, 1 H), 4.17 (q, *J* = 7.1 Hz, 2 H), 3.39–3.24 (m, 1 H), 2.91–2.77 (m, 1 H), 2.27 (s, 3 H), 1.27 (t, *J* = 7.1 Hz, 3 H); ^{13}C NMR (100 MHz, CDCl$_3$) δ 167.4, 165.8, 140.6, 131.8, 127.3, 122.0, 101.7, 82.2, 59.6, 38.0, 14.4, 14.0. HRMS (ESI) calcd for $C_{14}H_{16}BrO_3$ [M+H]$^+$ 311.0277, found 311.0218.

Supplementary Table 3. Metal Catalyzed Coupling of Enamides and Diazo Compounds

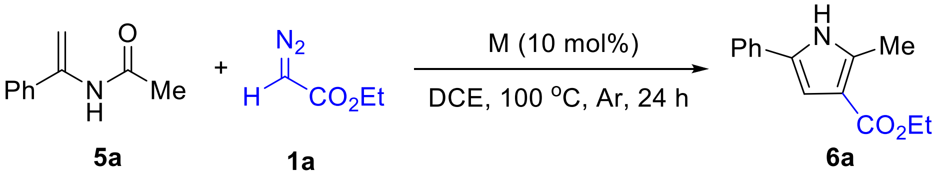

metal catalyst	**5a** conv.	**6a** yield
none	< 5%	trace
$Pd(OAc)_2$	< 5%	trace
CuI	< 5%	trace
$Rh(OAc)_2$	< 5%	trace
$(ArO)_3PAu(TA\text{-}Ph)OTf$	< 20%	n.d.
IPrAu(TA-H)OTf	< 20%	n.d.
$^tBu_3PAuNTf_2$	< 20%	n.d.
10 mol% I_2, 1 mol% $Ru(bpy)_3Cl_2$, blue light, r.t.	<10%	n.d.

Reaction conditions: 10 mol% metal catalyst was added to a DCE solution (3 mL) of enamide **5a** (0.3 mmol) and EDA **1a** (0.3 mmol), and reaction was kept under Ar at 100 °C for 24 h.

Key Cyclopropane Intermediate

Control experiment 1:

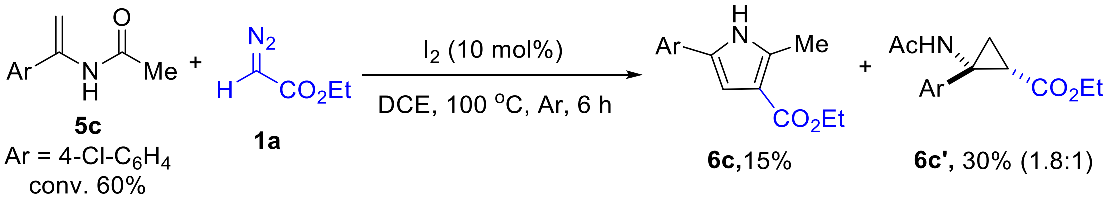

5c
Ar = 4-Cl-C_6H_4
conv. 60%

1a

6c,15%

6c', 30% (1.8:1)

To a 50 mL Schlenk tube with a stir bar was added enamide **5c** (59 mg, 0.3 mmol), **1a** (35 mg, 0.3 mmol) and 3 mL of DCE. The Schlenk tube was vacuumed and purged with argon three times before it was tightly screw-capped. The reaction mixture was stirred at 100 ºC for 6 h, and cooled to room temperature. The reaction solution was evaporated, and the residue was purified by column chromatography (PE/EA) to afford the desired product **6c** (12 mg) in 15% yield. ^1H NMR (400 MHz, $CDCl_3$) δ 8.77 (s, 1 H), 7.41–7.35 (m, 2 H), 7.34–7.27 (m, 2 H), 6.81 (d, J = 3.0 Hz, 1 H), 4.29 (q, J = 7.1 Hz, 2 H), 2.57 (s, 3 H), 1.36 (t, J = 7.2 Hz, 3 H); ^{13}C NMR (100 MHz, $CDCl_3$) δ 165.6, 136.6, 132.1, 130.3, 129.0, 128.9, 124.9, 113.5, 107.8, 59.7, 14.5, 13.4. HRMS (ESI) calcd for $C_{14}H_{14}ClNNaO_2$ $[M+Na]^+$ 286.0605, found 286.0610.

In addition, the key acetamidocyclopropane Intermediate **6c´** (25 mg) was obtained in 30% yield. ***Ethyl 2-acetamido-2-(4-chlorophenyl)cyclopropane-1-carboxylate* 6c´**(1.8:1)**:** ^1H NMR (400 MHz, $CDCl_3$) δ 7.81–7.74 (m, 2 H), 7.39–7.37 (m, 2 H), 5.54 (s, 0.32 H), 4.97 (s, 0.59 H), 4.42–4.13 (m, 2 H), 3.62 (dd, J = 16.5, 6.0 Hz, 0.66 H), 3.41 (dd, J = 15.5, 6.8 Hz, 0.34 H), 3.35–3.06 (m, 2 H), 1.75 (s, 1.95 H), 1.42 (s, 1.15 H), 1.33 (t, J = 7.1 Hz, 3 H); ^{13}C NMR (100 MHz, $CDCl_3$) δ 172.0, 171.4, 171.3, 169.5, 137.63, 137.61,

131.5, 131.4, 129.5, 129.4, 128.83, 128.80, 103.1, 102.2, 61.11, 61.00, 53.7, 51.7, 37.8, 28.9, 24.5, 14.30, 14.26. HRMS (ESI) calcd for $C_{14}H_{18}ClNO_3$ $[M+H]^+$ 282.0891, found 282.0895.

Control experiment 2:

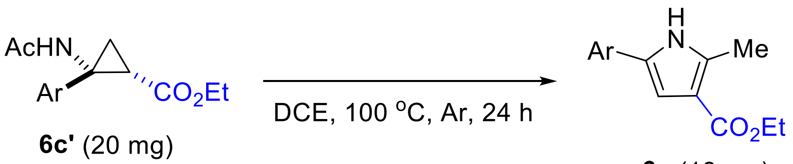

6c' (20 mg)
Ar = 4-Cl-C_6H_4
6c (18 mg)

To a 10 mL tube with a stir bar was added acetamidocyclopropane **6c´** (20 mg) and 1 mL of DCE. The reaction mixture was stirred at 100 ºC for 24 h, and cooled to room temperature. The reaction solution was evaporated to obtain the pure product **6c** (18 mg) in nearly quantitative yield. The results of experiment **3** and **4** indicated that acetamidocyclopropane was the key intermediate.

Gram Synthesis of Pyrroles

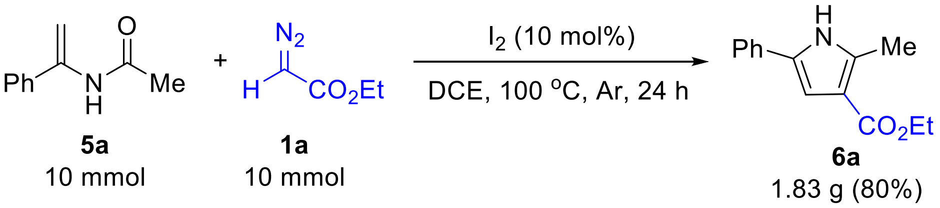

5a 10 mmol
1a 10 mmol
6a 1.83 g (80%)

To a 250 mL Schlenk tube with a stir bar was added enamides **5a** (1.61 g, 10 mmol), diazo compound **1a** (1.14 g, 10 mmol) and 100 mL of DCE, then iodine (254 mg, 1 mmol) was added. The Schlenk tube was vacuumed and purged with argon three times before it was tightly screw-capped. The reaction mixture was stirred at 100 ºC for 24 h, and cooled to room temperature. The reaction solution was evaporated, and the residue was purified by column chromatography (PE/EA) to afford the desired product **6a** in 80% yield (1.83 g).

Experimental Procedure and Date for the Synthesis of Pyrroles from Enamides

To a 50 mL Schlenk tube with a stir bar was added enamides **5** (0.3 mmol), diazo compound **1** (0.3 mmol) and 3 mL of DCE, then iodine (8 mg, 0.03 mmol) was added. The Schlenk tube was vacuumed and purged with argon three times before it was tightly screw-capped. The reaction mixture was stirred at 100 ºC for 24 h, and cooled to room temperature. The reaction solution was evaporated, and the residue was purified by column chromatography (PE/EA) to afford the desired product **6a–6t**.

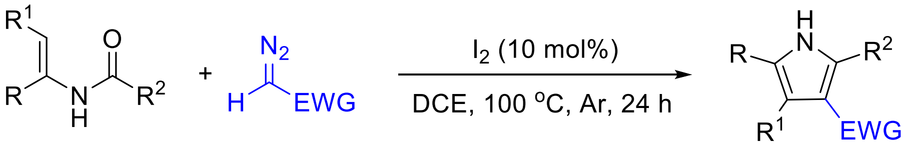

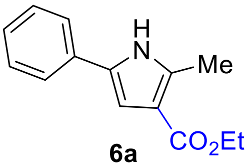

6a

***Ethyl 2-methyl-5-phenyl-1H-pyrrole-3-carboxylate* 6a:** slightly yellow solid: ^{1}H NMR (400 MHz, $CDCl_3$) δ 8.67 (s, 1 H), 7.47–7.45 (m, 2 H), 7.37–7.33 (m, 2 H), 7.23–7.19 (m, 1 H), 6.84 (d, J = 2.9 Hz, 1 H), 4.30 (q, J = 7.1 Hz, 2 H), 2.58 (s, 3 H), 1.36 (t, J = 7.1 Hz, 3 H); ^{13}C NMR (100 MHz, $CDCl_3$) δ 165.6, 136.2, 131.9, 130.0, 128.9, 126.5, 123.7, 113.4, 107.4, 59.5, 14.5, 13.3. HRMS (ESI) calcd for $C_{14}H_{15}NNaO_2$ $[M+Na]^+$ 252.0995, found 252.1002.

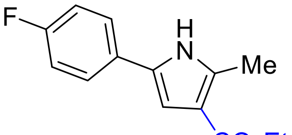

6b

***Ethyl 5-(4-fluorophenyl)-2-methyl-1H-pyrrole-3-carboxylate* 6b**: slightly yellow solid; ^{1}H NMR (400 MHz, $CDCl_3$) δ 8.78 (s, 1 H), 7.50–7.37 (m, 1 H), 7.10–6.99 (m, 1 H), 6.76 (d, J = 2.9 Hz, 1 H), 4.29 (q, J = 7.1 Hz, 2 H), 2.57 (s, 3 H), 1.36 (t, J = 7.1 Hz, 3 H); ^{13}C NMR (100 MHz, $CDCl_3$) δ 165.7, 161.6 ($^1J_{CF}$ = 246.0 Hz), 136.3, 129.2, 128.2 ($^4J_{CF}$ = 3.2 Hz), 125.4 ($^3J_{CF}$ = 7.9 Hz), 115.8 ($^2J_{CF}$ = 21.8 Hz), 113.3, 107.2, 59.6, 14.5, 13.3. HRMS (ESI) calcd for $C_{14}H_{15}FNO_2$ $[M+H]^+$ 248.1081, found 248.1069.

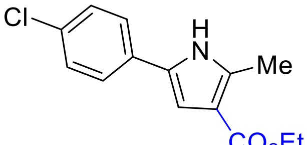

6c

***Ethyl 5-(4-chlorophenyl)-2-methyl-1H-pyrrole-3-carboxyl ate* 6c:** slightly yellow solid; ^{1}H NMR (400 MHz, $CDCl_3$) δ 8.77 (s, 1 H), 7.41–7.35 (m, 2 H), 7.34–7.27 (m, 2 H), 6.81 (d, J = 3.0 Hz, 1 H), 4.29 (q, J = 7.1 Hz, 2 H), 2.57 (s, 3 H), 1.36 (t, J = 7.2 Hz, 3 H); ^{13}C NMR (100 MHz, $CDCl_3$) δ 165.6, 136.6, 132.1, 130.3, 129.0, 128.9, 124.9, 113.5, 107.8, 59.7, 14.5, 13.4. HRMS (ESI) calcd for $C_{14}H_{14}ClNNaO_2$ $[M+Na]^+$ 286.0605, found 286.0610.

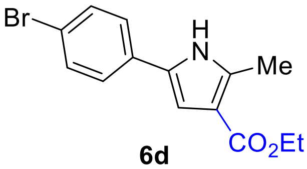

6d

***Ethyl 5-(4-bromophenyl)-2-methyl-1H-pyrrole-3-car boxylate* 6d:** slightly yellow solid; ^{1}H NMR (400 MHz, $CDCl_3$) δ 8.61 (s, 1 H), 7.47 (d, J = 8.5 Hz, 2 H), 7.32 (d, J = 8.5 Hz, 2 H), 6.83 (d, J = 2.9 Hz, 1 H), 4.29 (q, J = 7.1 Hz, 2 H), 2.57 (s, 3 H), 1.36 (t, J = 7.1 Hz, 3 H); ^{13}C NMR (100 MHz, $CDCl_3$) δ 165.4, 136.6, 132.0, 130.7, 128.8, 125.1, 120.1, 113.6, 107.9, 59.6, 14.5, 13.4. HRMS (ESI) calcd for $C_{14}H_{14}BrNNaO_2$ $[M+Na]^+$ 330.0100, found 330.0086.

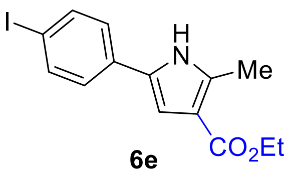

6e

***Ethyl 5-(4-iodophenyl)-2-methyl-1H-pyrrole-3-carboxylate* 6e:** white solid; ^{1}H NMR (400 MHz, DMSO) δ 11.70 (s, 1 H), 7.69 (d, J = 8.4 Hz, 2 H), 7.46 (d, J = 8.4 Hz, 2 H), 6.81 (d, J = 2.4 Hz, 2 H), 4.17 (q, J = 7.1 Hz, 2 H), 2.47 (s, 3 H), 1.26 (t, J = 7.1 Hz,

3 H); ^{13}C NMR (100 MHz, DMSO) δ 164.6, 137.5, 137.1, 131.5, 128.7, 125.6, 112.3, 107.4, 91.2, 58.9, 14.6, 12.9; HRMS (ESI) calcd for $C_{14}H_{15}INO_2$ $[M+H]^+$ 356.0142, found 356.0141.

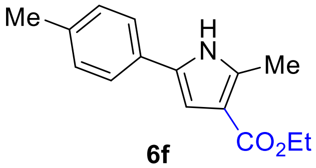

6f

***Ethyl 2-methyl-5-(p-tolyl)-1H-pyrrole-3-carboxylate* 6f:** slightly brown solid; ^1H NMR (400 MHz, $CDCl_3$) δ 8.76 (s, 1 H), 7.36 (d, *J* = 8.1 Hz, 2 H), 7.16 (d, *J* = 8.0 Hz, 2 H), 6.80 (d, *J* = 2.9 Hz, 1 H), 4.30 (q, *J* = 7.1 Hz, 2 H), 2.57 (s, 3 H), 2.34 (s, 3 H), 1.36 (t, *J* = 7.1 Hz, 3 H); ^{13}C NMR (100 MHz, $CDCl_3$) δ 165.8, 136.2, 135.9, 130.1, 129.5, 129.1, 123.6, 113.1, 106.7, 59.5, 21.1, 14.5, 13.3; HRMS (ESI) calcd for $C_{15}H_{17}NNaO_2$ $[M+Na]^+$ 266.1151, found 266.1154.

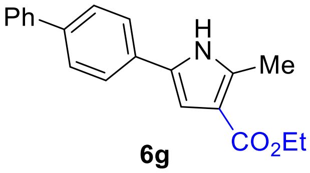

6g

***Ethyl 5-([1,1'-biphenyl]-4-yl)-2-methyl-1H-pyrrole-3-carboxylate* 6g:** slightly brown solid; ^1H NMR (400 MHz, $CDCl_3$) δ 9.02 (s, 1 H), 7.60–7.53 (m, 6 H), 7.46–7.38 (m, 2 H), 7.36–7.33 (m, 1 H), 6.92 (d, *J* = 2.6 Hz, 1 H), 4.33 (q, *J* = 7.1 Hz, 2 H), 2.60 (s, 3 H), 1.39 (t, *J* = 7.1 Hz, 3 H); ^{13}C NMR (100 MHz, $CDCl_3$) δ 165.8, 140.4, 139.0, 136.5, 130.7, 129.7, 128.8, 127.4, 127.2, 126.7, 124.0, 113.3, 107.5, 59.6, 14.5, 13.4; HRMS (ESI) calcd for $C_{20}H_{20}NO_2$ $[M+H]^+$ 306.1489, found 306.1503.

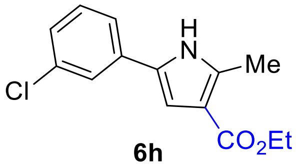

6h

***Ethyl 5-(3-chlorophenyl)-2-methyl-1H-pyrrole-3-carboxylate* 6h:** off white solid; ^1H NMR (400 MHz, $CDCl_3$) δ 8.92 (s, 1 H), 7.44 (t, *J* = 1.8 Hz, 1 H), 7.35–7.31 (m, 1 H), 7.26 (t, *J* = 7.8 Hz, 1 H), 7.16 (ddd, *J* = 7.8, 1.7, 1.1 Hz, 1 H), 6.85 (d, *J* = 2.9 Hz, 1 H), 4.30 (q, *J* = 7.1 Hz, 2 H), 2.57 (s, 3 H), 1.36 (t, *J* = 7.1 Hz, 3 H); ^{13}C NMR (100 MHz, $CDCl_3$) δ 165.6, 136.9, 134.8, 133.6, 130.1, 128.6, 126.3, 123.6, 121.7, 113.5, 108.3, 59.7, 14.5, 13.3; HRMS (ESI) calcd for $C_{14}H_{14}ClNNaO_2$ $[M+Na]^+$ 286.0605, found 286.0613.

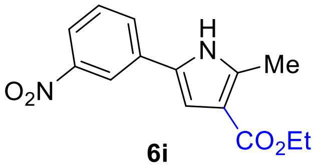

6i

***Ethyl 2-methyl-5-(3-nitrophenyl)-1H-pyrrole-3-carboxylate* 6i:** yellow solid; ^1H NMR (400 MHz, DMSO) δ 11.92 (s, 1 H), 8.45 (s, 1 H), 8.05 (d, *J* = 7.9 Hz, 1 H), 7.95 (dd, *J* = 8.1, 1.2 Hz, 1 H), 7.57 (t, *J* = 8.0 Hz, 1 H), 6.96 (d, *J* = 2.6 Hz, 1 H), 4.15 (q, *J* = 7.1 Hz, 2 H), 2.46 (s, 3 H), 1.24 (t, *J* = 7.1 Hz, 3 H); ^{13}C NMR (100 MHz, DMSO) δ 164.4, 148.6, 137.8, 133.6, 130.3, 129.9, 127.5, 120.4, 117.3, 112.7, 108.9, 59.0, 14.5, 12.8; HRMS (ESI) calcd for $C_{14}H_{15}N_2O_4$ $[M+H]^+$ 275.1026, found 275.1024.

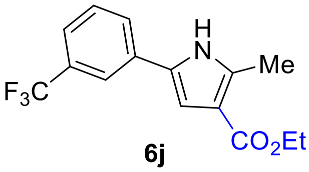

6j

***Ethyl 2-methyl-5-(3-(trifluoromethyl)phenyl)-1H-pyrrole-3-carboxylate* 6j:** slight yellow solid; ^{1}H NMR (400 MHz, DMSO) δ 11.78 (s, 1 H), 7.96 (s, 1 H), 7.90 (d, *J* = 7.8 Hz, 1 H), 7.51 (t, *J* = 7.8 Hz, 1 H), 7.44 (d, *J* = 7.7 Hz, 1 H), 6.92 (d, *J* = 2.7 Hz, 1 H), 4.13 (q, *J* = 7.1 Hz, 2 H), 2.45 (s, 3 H), 1.22 (t, *J* = 7.1 Hz, 3 H); ^{13}C NMR (100 MHz, DMSO) δ 164.5, 137.5, 132.9, 129.9, 129.8 ($^2J_{CF}$ = 31.5 Hz), 128.1, 127.4, 124.4 ($^1J_{CF}$ = 272.5 Hz), 122.26 ($^3J_{CF}$ = 3.8 Hz), 119.61 ($^3J_{CF}$ = 3.8 Hz), 112.5, 108.3, 58.9, 14.5, 12.9; HRMS (ESI) calcd for $C_{15}H_{15}F_3NO_2$ [M+H]$^+$ 298.1038, found 298.1049.

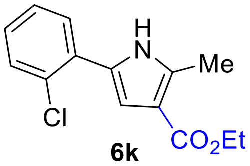

6k

***Ethyl 5-(2-chlorophenyl)-2-methyl-1H-pyrrole-3-carboxylate* 6k:** yellow solid; ^{1}H NMR (400 MHz, $CDCl_3$) δ 8.99 (s, 1 H), 7.53 (dd, *J* = 7.8, 1.5 Hz, 1H), 7.40 (dd, *J* = 8.0, 1.0 Hz, 1H), 7.29–7.2 (m, 1 H), 7.19–7.15 (m, 1 H), 6.91 (d, *J* = 2.9 Hz, 1 H), 4.29 (q, *J* = 7.1 Hz, 2 H), 2.60 (s, 3 H), 1.36 (t, *J* = 7.1 Hz, 3 H); ^{13}C NMR (100 MHz, $CDCl_3$) δ 165.4, 136.0, 130.7, 130.2, 129.9, 129.3, 127.8, 127.3, 127.1, 112.8, 111.1, 59.5, 14.5, 13.5; HRMS (ESI) calcd for $C_{14}H_{15}ClNO_2$ [M+H]$^+$ 264.0786, found 264.0789.

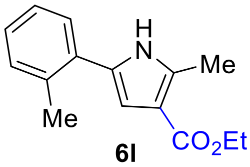

6l

***Ethyl 2-methyl-5-(o-tolyl)-1H-pyrrole-3-carboxylate* 6l:** slight yellow solid; ^{1}H NMR (400 MHz, $CDCl_3$) δ 8.34 (s, 1 H), 7.36–7.13 (m, 4 H), 6.64 (d, *J* = 2.9 Hz, 1 H), 4.28 (q, *J* = 7.1 Hz, 2 H), 2.58 (s, 3 H), 2.45 (s, 3 H), 1.35 (t, *J* = 7.1 Hz, 3 H); ^{13}C NMR (100 MHz, $CDCl_3$) δ 165.7, 135.3 (2 C), 131.8, 131.0, 129.1, 127.6, 127.1, 126.0, 112.8, 110.3, 59.5, 21.2, 14.5, 13.3; HRMS (ESI) calcd for $C_{15}H_{18}NO_2$ [M+H]$^+$ 244.1332, found 244.1334.

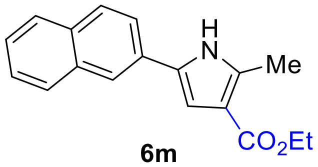

6m

***Ethyl 2-methyl-5-(naphthalen-2-yl)-1H-pyrrole-3-car boxylate* 6m:** yellow solid; ^{1}H NMR (400 MHz, $CDCl_3$) δ 8.76 (s, 1 H), 7.92–7.72 (m, 4 H), 7.64 (dd, *J* = 8.7, 1.8 Hz, 1 H), 7.51–7.38 (m, 2 H), 6.98 (d, *J* = 2.9 Hz, 1 H), 4.32 (q, *J* = 7.1 Hz, 2 H), 2.63 (s, 3 H), 1.38 (t, *J* = 7.1 Hz, 3 H); ^{13}C NMR (100 MHz, $CDCl_3$) δ 165.6, 136.6, 133.6, 132.2, 130.0, 129.2, 128.7, 127.7, 127.6, 126.6, 125.58, 122.9, 120.9, 113.6, 108.0, 59.6, 14.5, 13.4; HRMS (ESI) calcd for $C_{18}H_{17}NNaO_2$ [M+Na]$^+$ 302.1151, found 302.1158.

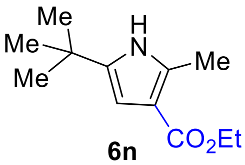

6n

***Ethyl 5-(tert-butyl)-2-methyl-1H-pyrrole-3-carboxylate* 6q:** slight yellow solid; ^{1}H NMR (400 MHz, $CDCl_3$) δ 8.21 (s, 1 H), 6.23 (d, *J* = 3.0 Hz, 1 H), 4.25 (q, *J* = 7.1 Hz, 2 H), 2.49 (s, 3 H), 1.33 (t, *J* = 7.1 Hz, 3 H), 1.27 (s, 9 H); ^{13}C NMR (100 MHz, $CDCl_3$) δ 165.9, 139.9, 134.1, 111.0, 104.0, 59.2, 31.1, 30.2, 14.5, 13.2. HRMS (ESI) calcd for $C_{12}H_{19}NNaO_2$ [M+Na]$^+$ 232.1308, found 232.1306.

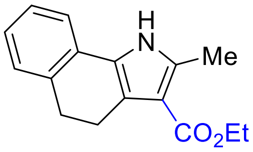

6o

***Ethyl 2-methyl-4,5-dihydro-1H-benzo[g]indole-3-carboxylate* 6n:** slight yellow solid; ^{1}H NMR (400 MHz, $CDCl_3$) δ 8.71 (s, 1 H), 7.21–7.14 (m, 3 H), 7.10–7.03 (m, 1 H), 4.31 (q, J = 7.1 Hz, 2 H), 3.08–2.85 (m, 4 H), 2.59 (s, 3 H), 1.37 (t, J = 7.1 Hz, 3 H); ^{13}C NMR (100 MHz, $CDCl_3$) δ 166.2, 136.5, 134.6, 128.5, 128.2, 126.4, 126.1, 125.3, 121.7, 118.0, 110.5, 59.3, 29.5, 21.5, 14.5, 13.9; HRMS (ESI) calcd for $C_{16}H_{17}NNaO_2$ $[M+Na]^+$ 278.1151, found 278.1153.

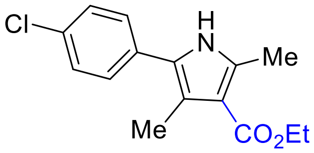

6p

***Ethyl 5-(4-chlorophenyl)-2,4-dimethyl-1H-pyrrole-3-carboxylate* 6o:** brown solid; ^{1}H NMR (400 MHz, $CDCl_3$) δ 8.34 (s, 1 H), 7.38–7.31 (m, 2 H), 7.31–7.27 (m, 2 H), 4.27 (q, J = 7.1 Hz, 2 H), 2.53 (s, 3 H), 2.34 (s, 3 H), 1.35 (t, J = 7.1 Hz, 3 H); ^{13}C NMR (100 MHz, $CDCl_3$) δ 166.3, 135.9, 132.3, 131.2, 128.8, 128.4, 126.1, 118.3, 112.4, 59.3, 14.5, 14.0, 11.8; HRMS (ESI) calcd for $C_{15}H_{16}ClNNaO_2$ $[M+Na]^+$ 300.0762, found 30.0768.

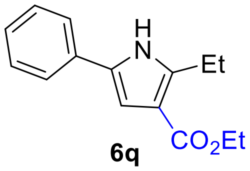

6q

***Ethyl 2-ethyl-5-phenyl-1H-pyrrole-3-carboxylate* 6p:** slight yellow solid; ^{1}H NMR (400 MHz, $CDCl_3$) δ 9.06 (s, 1 H), 7.51–7.49 (m, 2 H), 7.35 (t, J = 7.8 Hz, 2 H), 7.21 (t, J = 7.4 Hz, 1 H), 6.90–6.82 (m, 1 H), 4.31 (q, J = 7.1 Hz, 2 H), 3.03 (q, J = 7.5 Hz, 2 H), 1.37 (t, J = 7.1 Hz, 3 H), 1.29 (t, J = 7.6 Hz, 3 H); ^{13}C NMR (100 MHz, $CDCl_3$) δ 165.6, 142.3, 131.8, 130.0, 128.8, 126.4, 123.7, 112.3, 107.4, 59.5, 20.7, 14.4, 13.8; HRMS (ESI) calcd for $C_{15}H_{17}NNaO_2$ $[M+Na]^+$ 266.1151, found 266.1146.

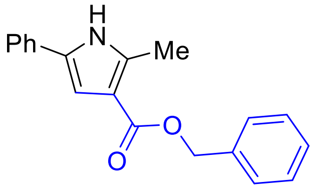

6r

***Benzyl 2-methyl-5-phenyl-1H-pyrrole-3-carboxylate* 6r:** slight yellow solid; ^{1}H NMR (400 MHz, $CDCl_3$) δ 8.82 (s, 1 H), 7.45–7.41 (m, 4 H), 7.39–7.29 (m, 5 H), 7.19 (t, J = 7.4 Hz, 1 H), 6.87–6.86 (m, 1 H), 5.29 (s, 2 H), 2.55 (s, 3 H); ^{13}C NMR (100 MHz, $CDCl_3$) δ 165.5, 136.7, 131.7, 130.14, 130.09, 128.9, 128.5, 127.9 (2 C), 126.5, 123.7, 112.8, 107.4, 65.4, 13.4; HRMS (ESI) calcd for $C_{19}H_{18}NO_2$ $[M+H]^+$ 292.1332, found 292.1345.

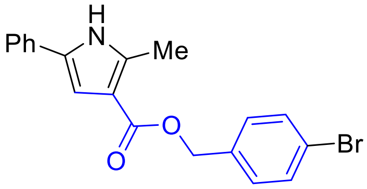

6s

***4-Bromobenzyl 2-methyl-5-phenyl-1H-pyrrole-3-car boxylate* 6s:** slight yellow solid; ^{1}H NMR (400 MHz, DMSO) δ 11.63 (s, 1 H), 7.58 (d, J = 7.4 Hz, 2 H), 7.50 (d, J = 8.4 Hz, 2 H), 7.32 (d, J = 8.4 Hz, 2 H), 7.28 (t, J = 7.8 Hz, 2 H), 7.11 (t, J = 7.4 Hz, 1 H), 6.76 (d, J = 2.7 Hz, 1 H), 5.14 (s, 2 H), 2.44 (s, 3 H); ^{13}C NMR (100 MHz, DMSO) δ 164.3, 137.2, 136.7, 131.9, 131.4, 129.9, 129.9, 128.8, 126.2, 123.6, 121.0, 111.6, 106.7, 63.7, 13.0; HRMS (ESI) calcd for $C_{19}H_{16}BrNNaO_2$ $[M+Na]^+$ 392.0257, found 392.0269.

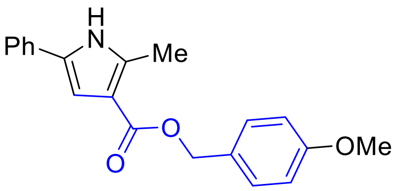

6t

***4-Methoxybenzyl 2-methyl-5-phenyl-1H-pyrrole-3-carboxylate* 6t:** white solid; ^{1}H NMR (400 MHz, $CDCl_3$) δ 8.56 (s, 1 H), 7.47–7.42 (m, 2 H), 7.39–7.33 (m, 4 H), 7.23–7.20 (m, 1 H), 6.94–6.87 (m, 2 H), 6.85 (d, *J* = 2.9 Hz, 1 H), 5.23 (s, 2 H), 3.81 (s, 3 H), 2.58 (s, 3 H); ^{13}C NMR (100 MHz, $CDCl_3$) δ 165.4, 159.4, 136.4, 131.7, 130.0, 129.8, 129.0, 128.9, 126.6, 123.7, 113.9, 113.1, 107.4, 65.2, 55.3, 13.5; HRMS (ESI) calcd for $C_{20}H_{19}NNaO_3$ $[M+Na]^+$ 344.1257, found 344.1254.

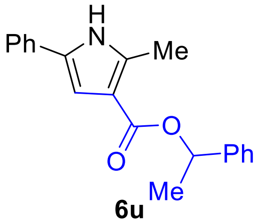

6u

***1-Phenylethyl 2-methyl-5-phenyl-1H-pyrrole-3-carboxylate* 6v:** slight yellow solid; ^{1}H NMR (400 MHz, $CDCl_3$) δ 8.97 (s, 1 H), 7.44 (d, *J* = 7.8 Hz, 2 H), 7.40 (d, *J* = 7.5 Hz, 2 H), 7.34–7.27 (m, 4 H), 7.25–7.22 (m, 1 H), 7.18 (t, *J* = 7.4 Hz, 1 H), 6.87 (d, *J* = 2.8 Hz, 1 H), 6.06 (q, *J* = 6.5 Hz, 1 H), 2.50 (s, 3 H), 1.61 (d, *J* = 6.6 Hz, 3 H); ^{13}C NMR (100 MHz, $CDCl_3$) δ 165.0, 142.5, 136.6, 131.8, 130.1, 128.8, 128.4, 127.5, 126.5, 125.9, 123.7, 113.1, 107.3, 71.4, 22.7, 13.4; HRMS (ESI) calcd for $C_{20}H_{20}NO_2$ $[M+H]^+$ 306.1489, found 306.1491.

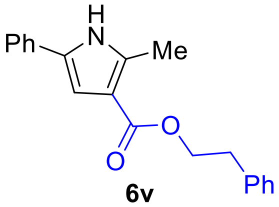

6v

***Phenethyl 2-methyl-5-phenyl-1H-pyrrole-3-carboxylate* 6u:** brown solid; ^{1}H NMR (400 MHz, $CDCl_3$) δ 8.77 (s, 1 H), 7.44 (d, *J* = 7.6 Hz, 2 H), 7.36–7.17 (m, 8 H), 6.81 (d, *J* = 2.9 Hz, 1 H), 4.45 (t, *J* = 7.0 Hz, 2 H), 3.04 (t, *J* = 7.0 Hz, 2 H), 2.50 (s, 3 H); ^{13}C NMR (100 MHz, $CDCl_3$) δ 165.6, 138.2, 136.4, 131.7, 130.0, 128.9, 128.9, 128.4, 126.5, 126.4, 123.7, 113.1, 107.3, 64.2, 35.4, 13.4; HRMS (ESI) calcd for $C_{20}H_{19}NNaO_2$ $[M+Na]^+$ 328.1308, found 328.1314.

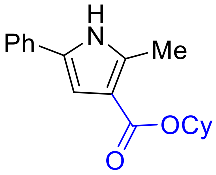

6w

***Cyclohexyl 2-methyl-5-phenyl-1H-pyrrole-3-carboxylate* 6w:** slight yellow solid; ^{1}H NMR (400 MHz, $CDCl_3$) δ 9.06 (s, 1 H), 7.50–7.47 (m, 2 H), 7.34 (t, *J* = 7.8 Hz, 2 H), 7.20 (t, *J* = 7.4 Hz, 1 H), 6.86 (d, *J* = 2.9 Hz, 1 H), 5.03–4.93 (m, 1 H), 2.58 (s, 3 H), 1.94–1.93 (m, 2 H), 1.81–1.72 (m, 2 H); 1.61–1.53 (m, 3 H); 1.46–1.32 (m, 3 H); ^{13}C NMR (100 MHz, $CDCl_3$) δ 165.3, 136.1, 131.9, 130.0, 128.8, 126.4, 123.7, 113.6, 107.4, 71.5, 31.8, 25.5, 23.7, 13.5; HRMS (ESI) calcd for $C_{18}H_{21}NNaO_2$ $[M+Na]^+$ 306.1465, found 306.1468.

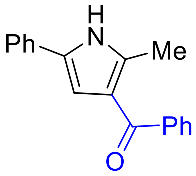

6x

***(2-Methyl-5-phenyl-1H-pyrrol-3-yl)(phenyl)methanone* 6x:** slight yellow solid; ^{1}H NMR (400 MHz, DMSO) δ 11.88 (s, 1 H), 7.76–7.71 (m, 2 H), 7.67–7.63 (m, 2 H), 7.59–7.56 (m, 1 H), 7.54–7.48 (m, 2 H), 7.36 (t, *J* = 7.8 Hz, 2 H), 7.19 (t, *J* = 7.4 Hz, 1 H), 6.67 (d, J = 2.6 Hz, 1 H), 2.51 (s, 3 H); ^{13}C NMR (100 MHz, DMSO) δ 190.9, 140.5, 137.9, 131.8, 131.2, 129.6, 128.9, 128.6, 128.3, 126.3, 123.7, 120.2, 108.4, 13.5; HRMS (ESI) calcd for $C_{18}H_{15}NNaO$ $[M+Na]^+$ 284.1046, found 284.1038.

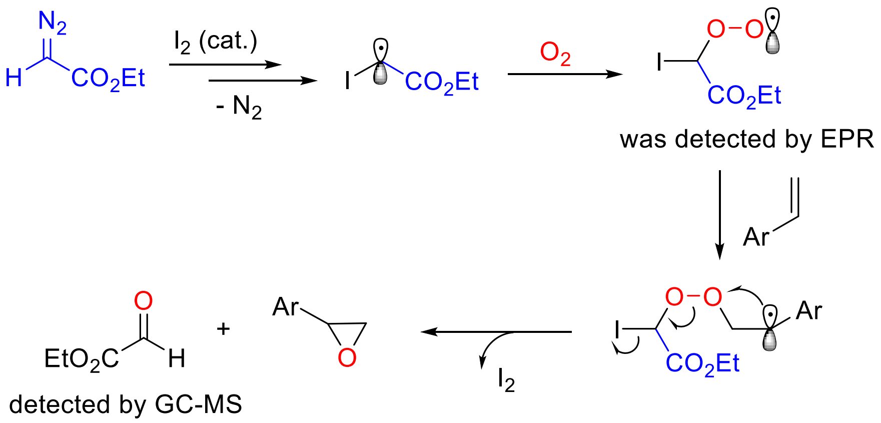

Supplementary Figure 6. iodine catalyzed diazo activation toward olefin epoxidation

Supplementary Table 4. Olefin epoxidation from iodine catalyzed diazo activation.

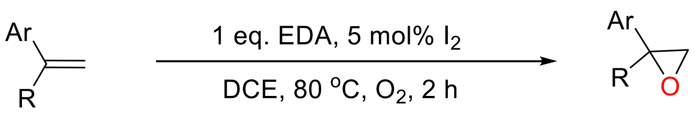

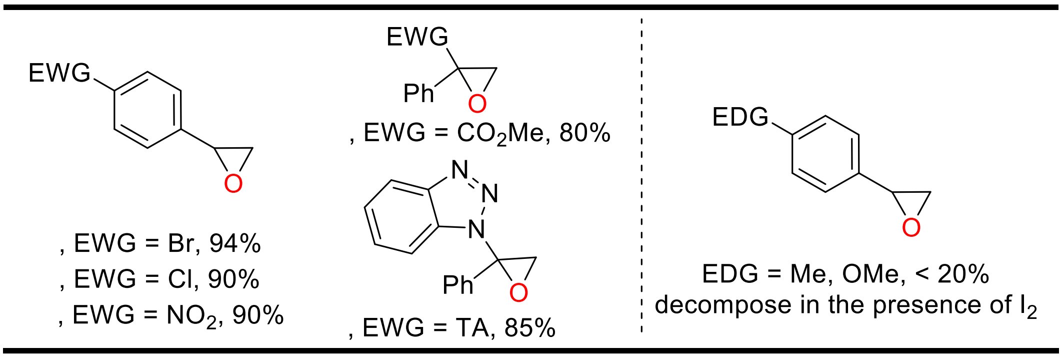

Reaction conditions: 5 mol% I_2 was added to a DCE solution of olefin **2** (0.3 mmol) and **1** (0.3 mmol), and reaction was kept under O_2 at 80 °C for 2 h.

Experimental Procedure and Date for Olefin Epoxidation

To a 50 mL Schlenk tube with a stir bar was added olefin **2** (0.3 mmol), ethyl diazoacetate **1a** (35 mg, 0.3 mmol) and DCE (3 mL), followed by iodine (4 mg, 0.015 mmol). The Schlenk tube was vacuumed and purged with oxygen three times before it was tightly screw-capped. The reaction mixture was stirred at 80 ºC for 2 h, and cooled to room temperature. Then the reaction solution was evaporated, and the residue was purified by column chromatography (PE/EA) to afford the desired epoxide **7a**–**7e**.

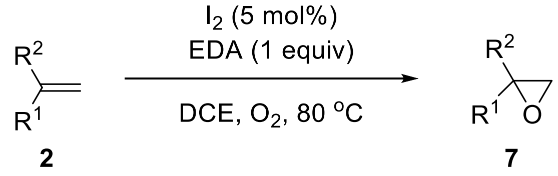

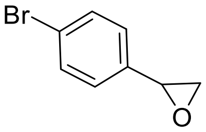

7a

2-(4-Bromophenyl)oxirane **7a**: colorless liquid, ^{1}H NMR (400 MHz, $CDCl_3$) δ 7.46 (d, *J* = 8.4 Hz, 1H), 7.14 (d, *J* = 8.4 Hz, 1H), 3.85–3.75 (m, 1H), 3.13 (dd, *J* = 5.2, 4.3 Hz, 1H), 2.74 (dd, *J* = 5.4, 2.5 Hz, 1H); ^{13}C NMR (100 MHz, $CDCl_3$) δ 136.7, 131.6, 127.1, 122.0, 51.8, 51.2. HRMS (ESI) calcd for C_8H_8BrO $[M+H]^+$ 198.9753, found 198.9767.

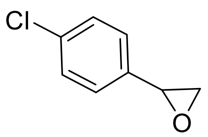

7b

2-(4-Chlorophenyl)oxirane **7b**: colorless liquid, ^{1}H NMR (400 MHz, $CDCl_3$) δ 7.32 (d, *J* = 8.4 Hz, 2 H), 7.21 (d, *J* = 8.4 Hz, 2 H), 3.85–3.80 (m, 1 H), 3.20–3.12 (m, 1 H), 2.76 (dd, *J* = 5.4, 2.4 Hz, 1 H); ^{13}C NMR (100 MHz, $CDCl_3$) δ 136.1, 133.9, 128.7, 126.8, 51.7, 51.2. HRMS (ESI) calcd for C_8H_8ClO $[M+H]^+$ 155.0258, found 155.0265.

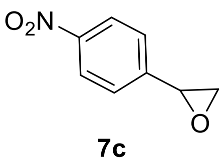

7c

2-(4-nitrophenyl)oxirane **7c**: faint yellow solid, ^{1}H NMR (400 MHz, $CDCl_3$) δ 8.22 (d, *J* = 8.7 Hz, 2 H), 7.45 (d, *J* = 8.7 Hz, 2 H), 3.99–3.93 (m, 1 H), 3.23 (dd, *J* = 5.4, 4.2 Hz, 1 H), 2.78 (dd, J = 5.5, 2.4 Hz, 1 H); ^{13}C NMR (100 MHz, $CDCl_3$) δ 147.7, 145.2, 126.2, 123.7, 51.6, 51.4. HRMS (ESI) calcd for $C_8H_8NO_3$ $[M+H]^+$ 166.0499, found 166.0452.

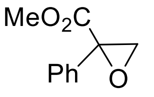

7d

Methyl 2-phenyloxirane-2-carboxylate **7d**: colorless liquid, ^{1}H NMR (400 MHz, $CDCl_3$) δ 7.54–7.48 (m, 2 H), 7.40–7.25 (m, 3 H), 7.26 (s, 3 H), 3.79 (s, 1 H), 3.43 (d, *J* = 6.4 Hz, 1 H), 2.98 (d, *J* = 6.4 Hz, 1 H); ^{13}C NMR (100 MHz, $CDCl_3$) δ 169.7, 134.2, 128.6, 128.3, 127.2, 58.1, 54.1, 53.0. HRMS (ESI) calcd for $C_{10}H_{11}O_3$ $[M+H]^+$ 179.0703, found 179.0694.

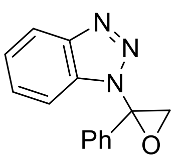

7e

1-(2-Phenyloxiran-2-yl)-1H-benzo[d][1,2,3]triazole **7e**: faint yellow solid, ^{1}H NMR (400 MHz, $CDCl_3$) δ 8.11–8.03 (m, H), 7.42–7.29 (m, 5 H), 7.22–7.19 (m, 3 H), 4.31 (d, *J* = 5.3 Hz, H), 3.39 (d, *J* = 5.3 Hz, H); ^{13}C NMR (100 MHz, $CDCl_3$) δ 146.3, 134.0, 133.2, 129.6, 128.9, 128.1, 125.7, 124.5, 120.1, 110.9, 69.2, 56.3. HRMS (ESI) calcd for $C_{14}H_{12}N_3O$ $[M+H]^+$ 238.0975, found 238.0978.

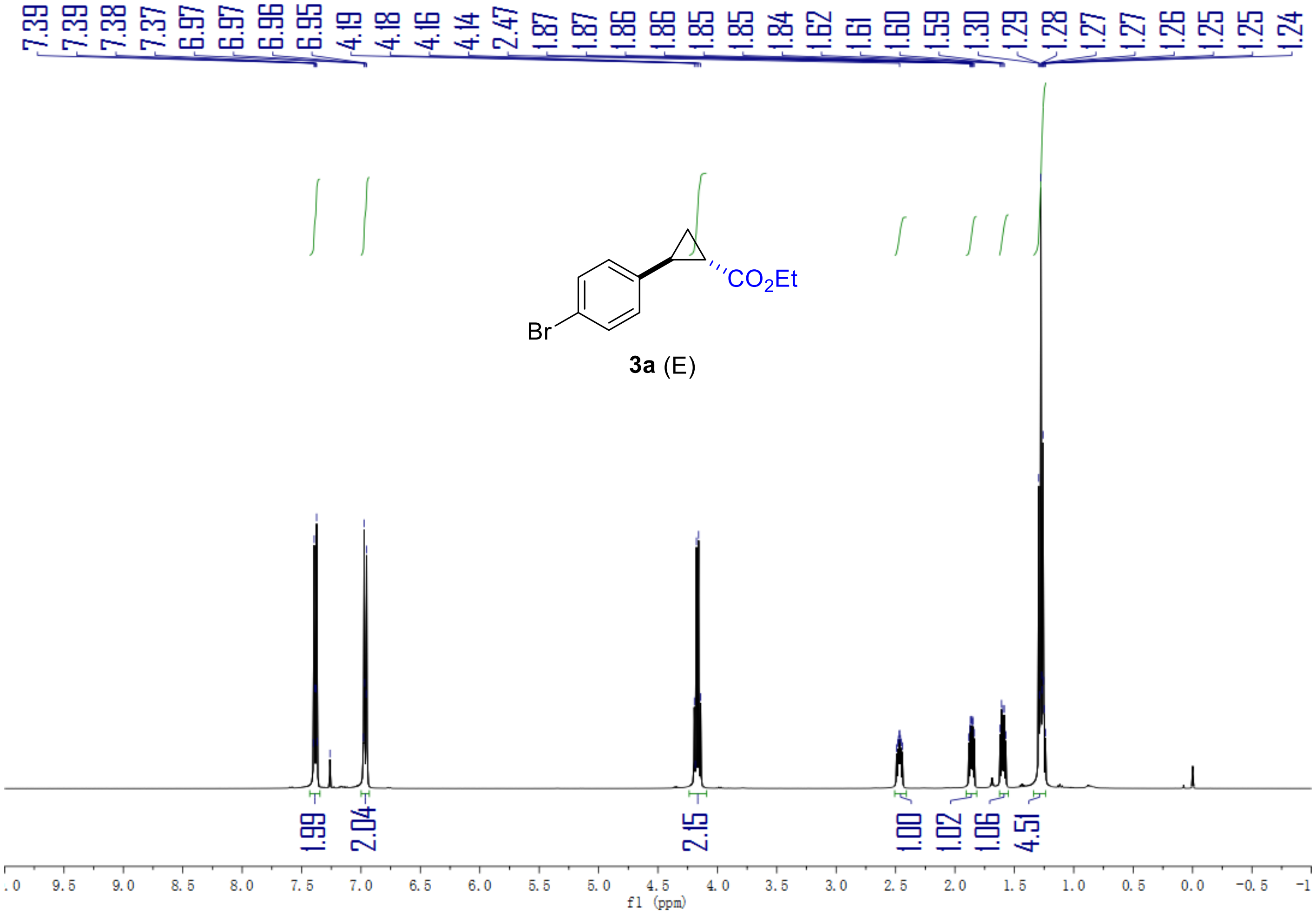

Supplementary Figure 7. ^{1}H NMR of **3a** (E)

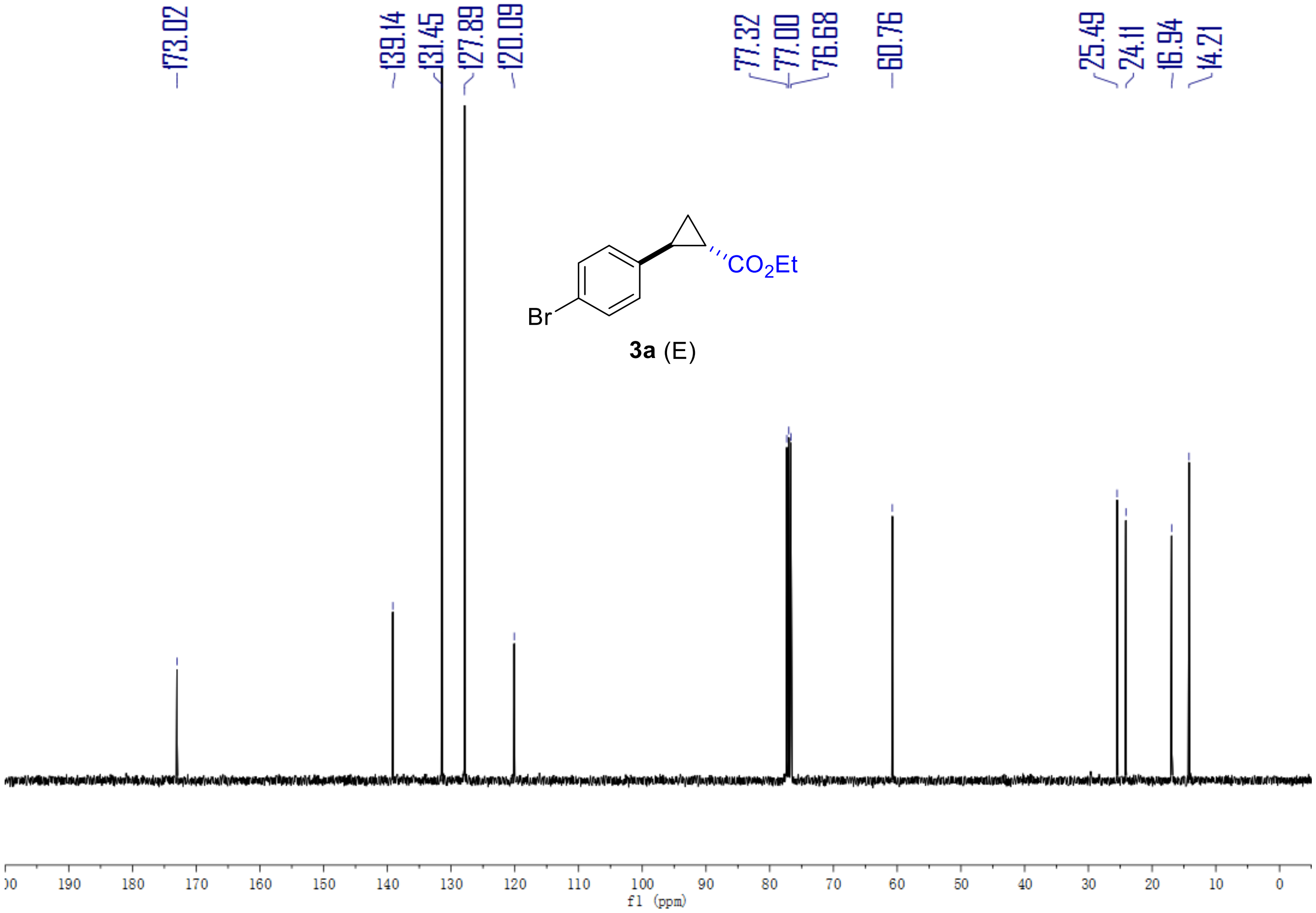

Supplementary Figure 8. ^{13}C NMR of **3a** (E)

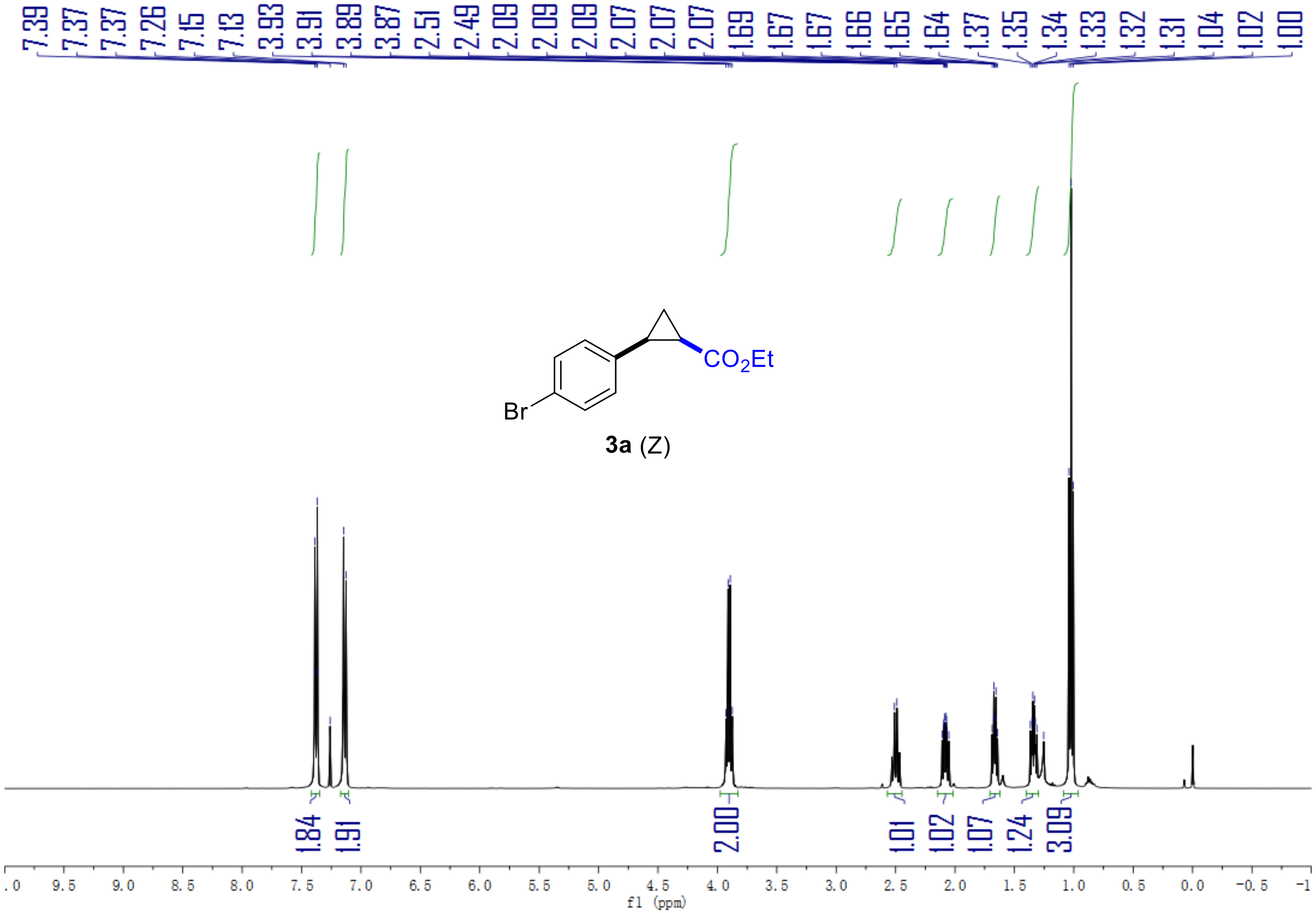

Supplementary Figure 9. ^{1}H NMR of **3a** (Z)

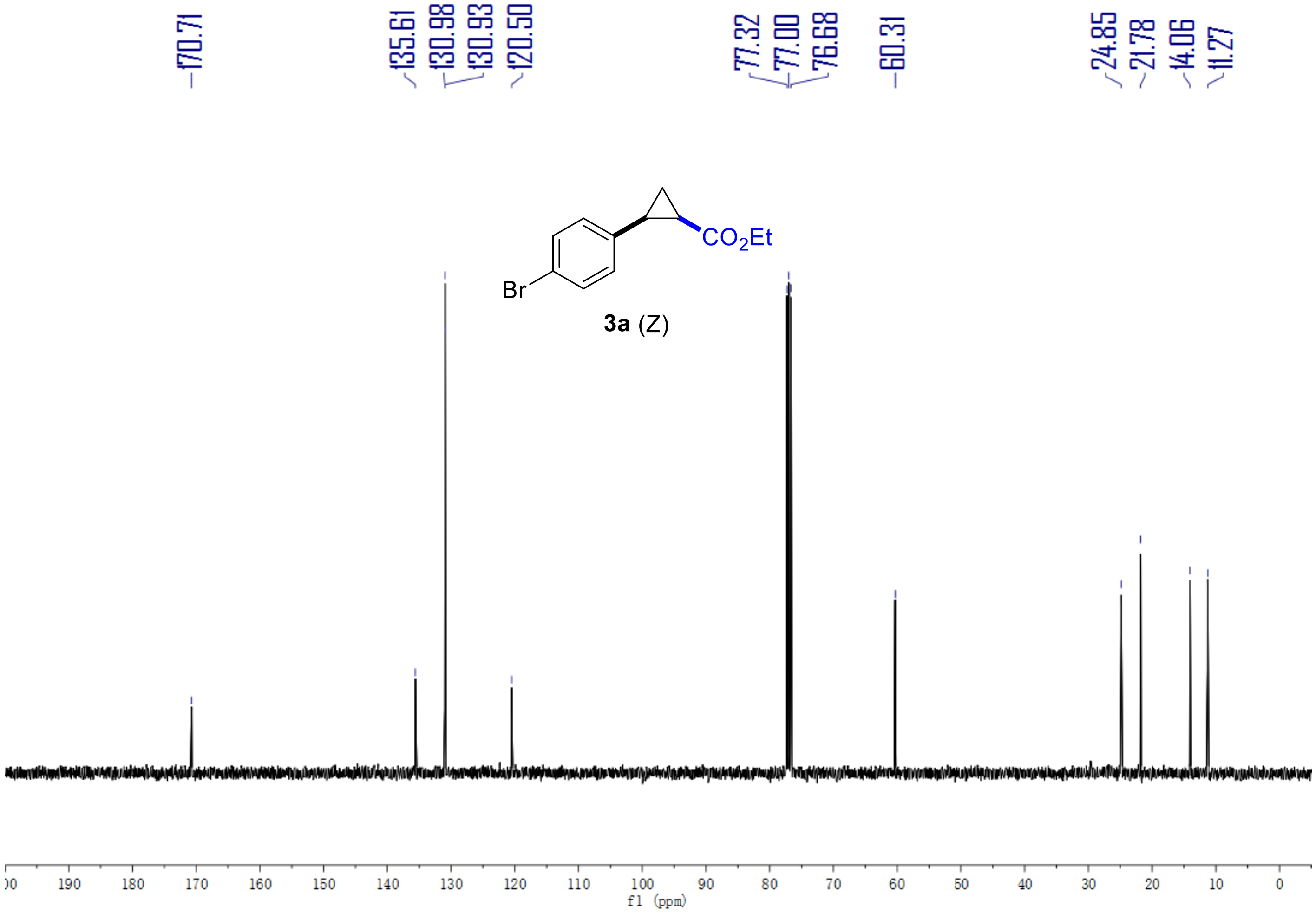

Supplementary Figure 10. ^{13}C NMR of **3a** (Z)

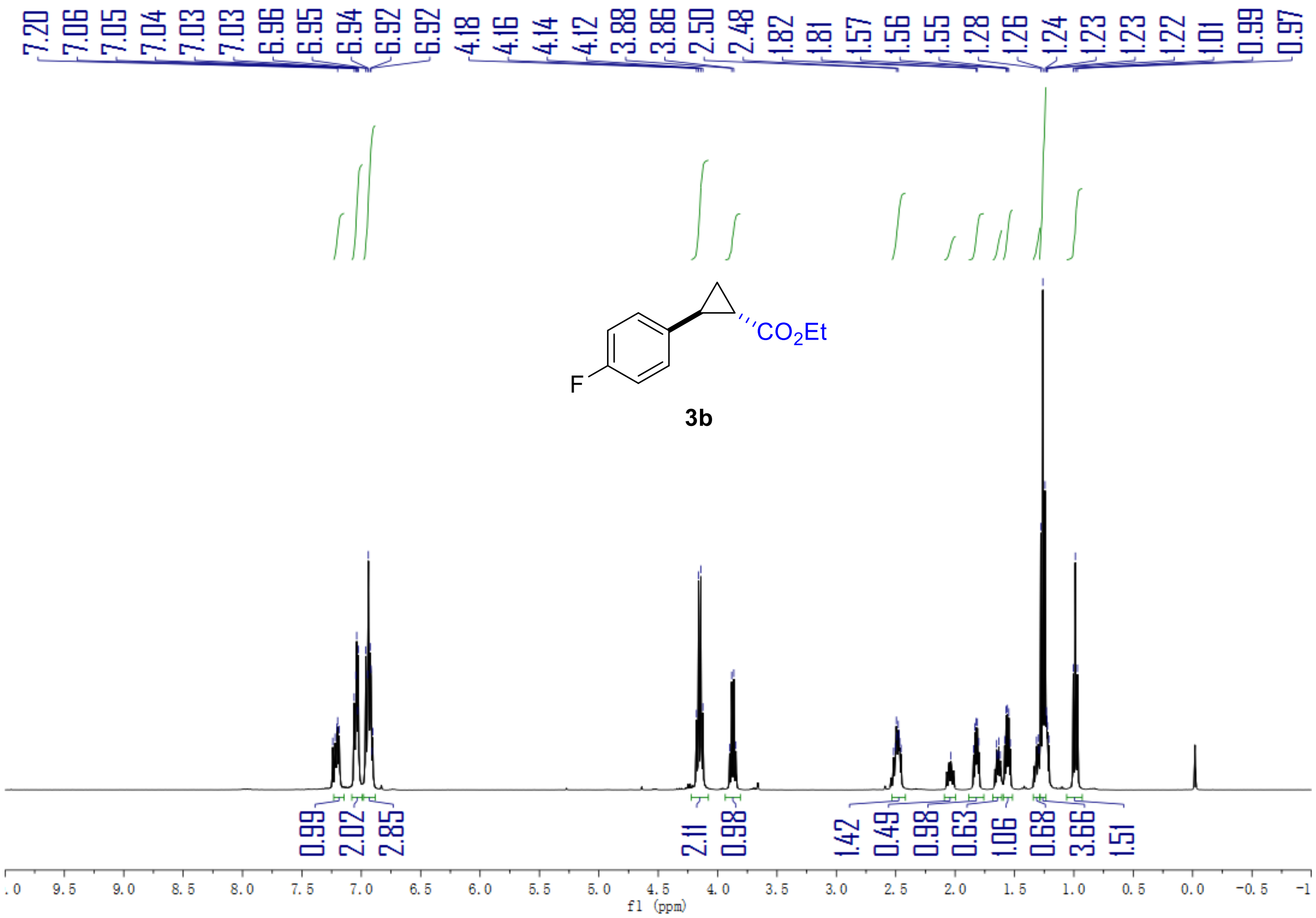

Supplementary Figure 11. ^{1}H NMR of **3b**

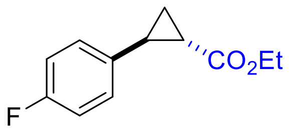

3b

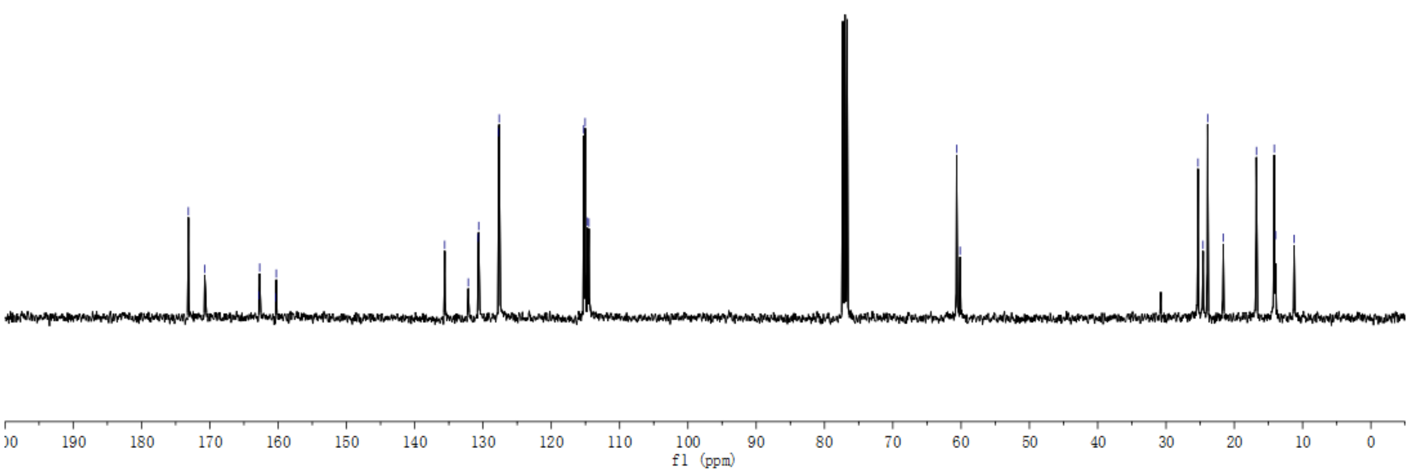

Supplementary Figure 12. ^{13}C NMR of **3b**

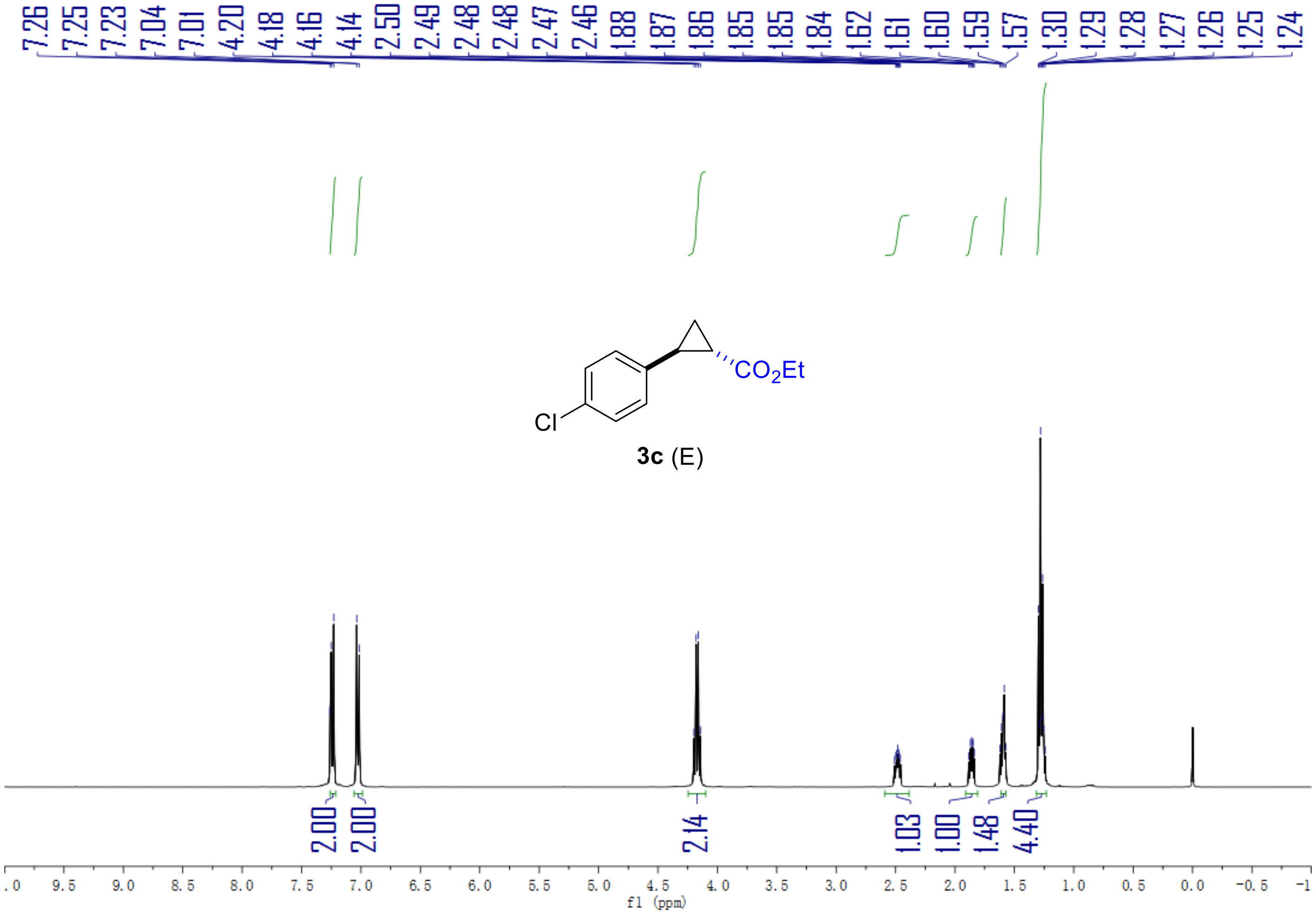

Supplementary Figure 13. ^{1}H NMR of **3c** (E)

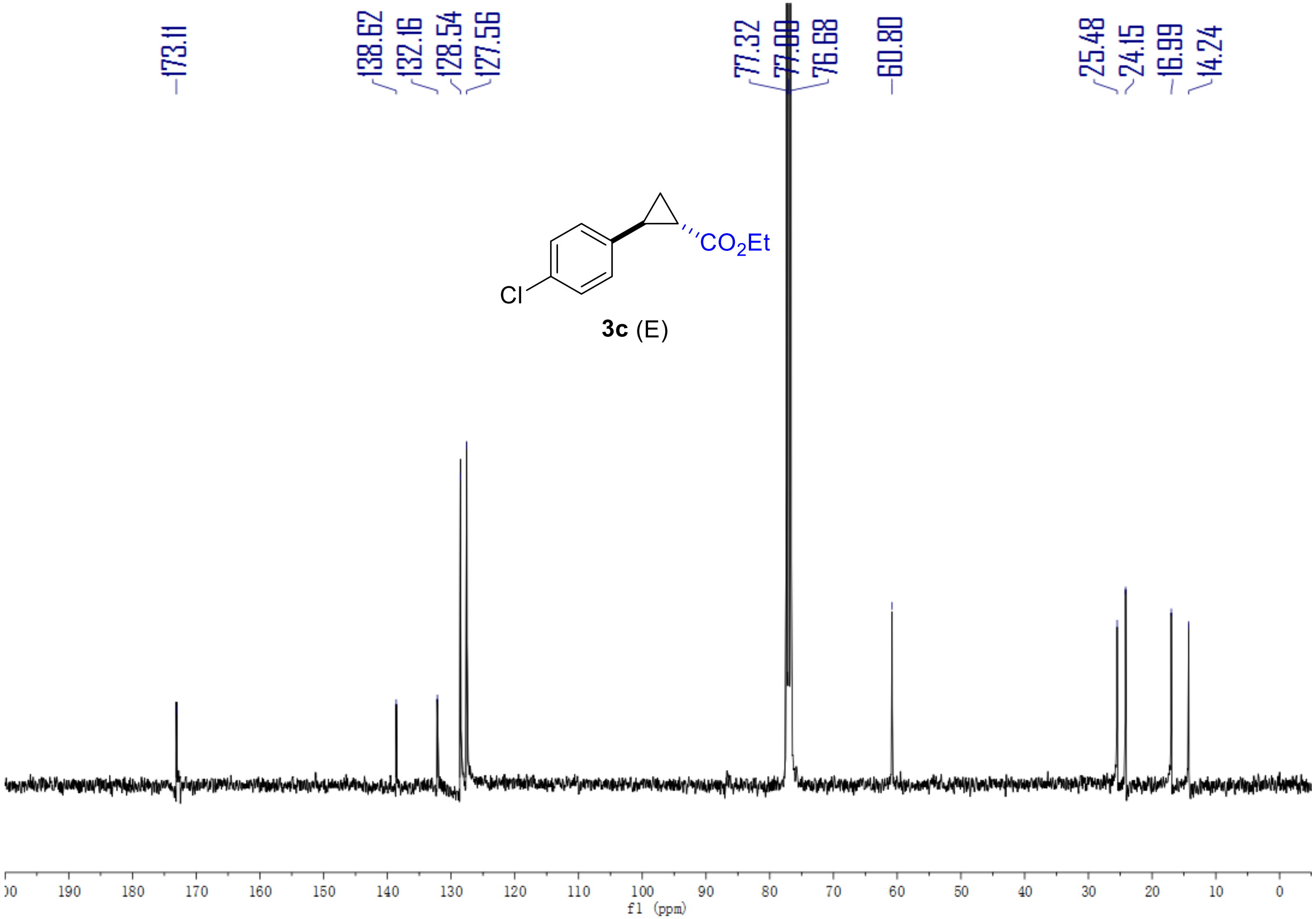

Supplementary Figure 14. ^{13}C NMR of **3c** (E)

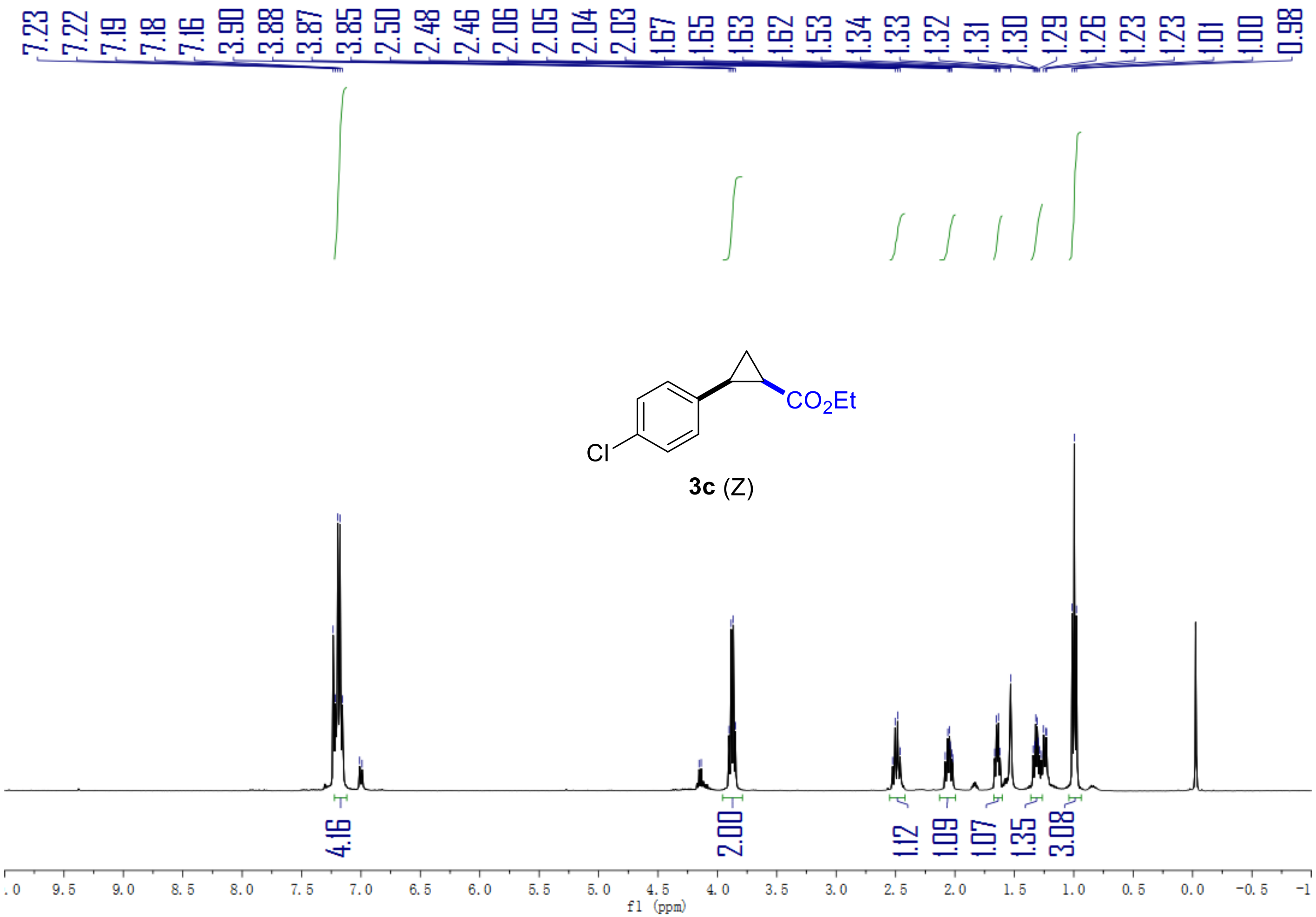

Supplementary Figure 15. ^{1}H NMR of **3c** (Z)

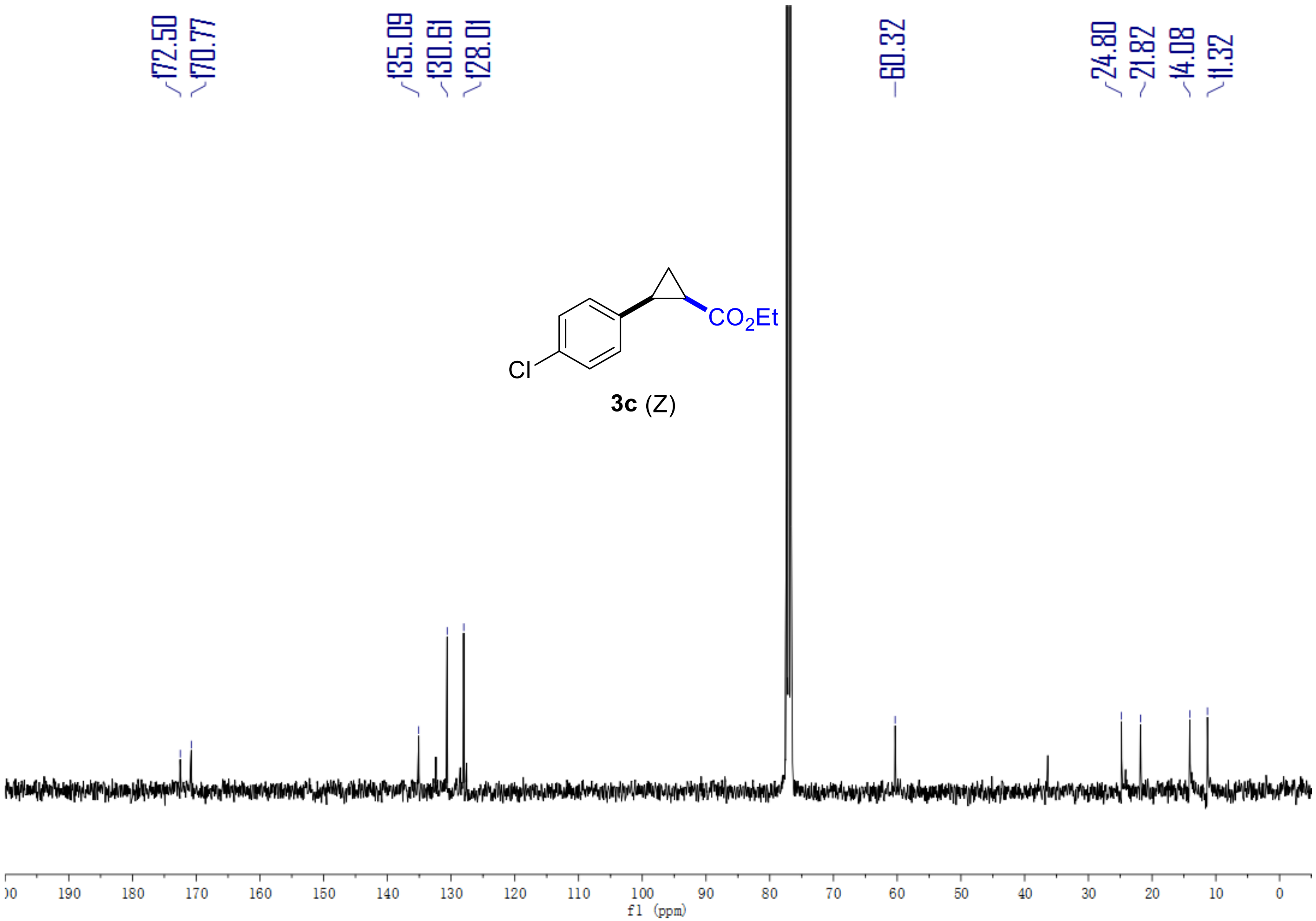

Supplementary Figure 16. ^{13}C NMR of **3c** (Z)

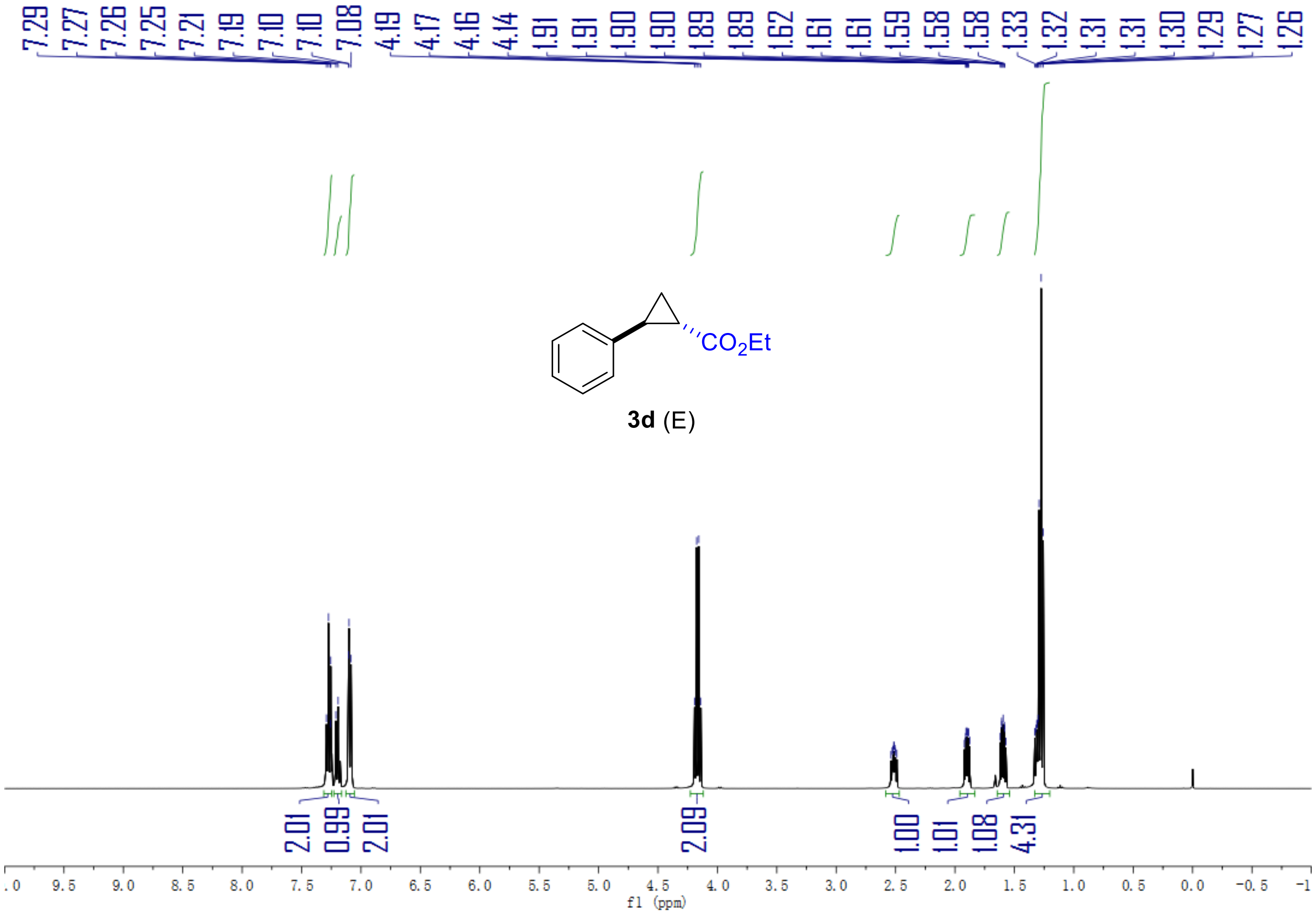

Supplementary Figure 17. ^{1}H NMR of **3d** (E)

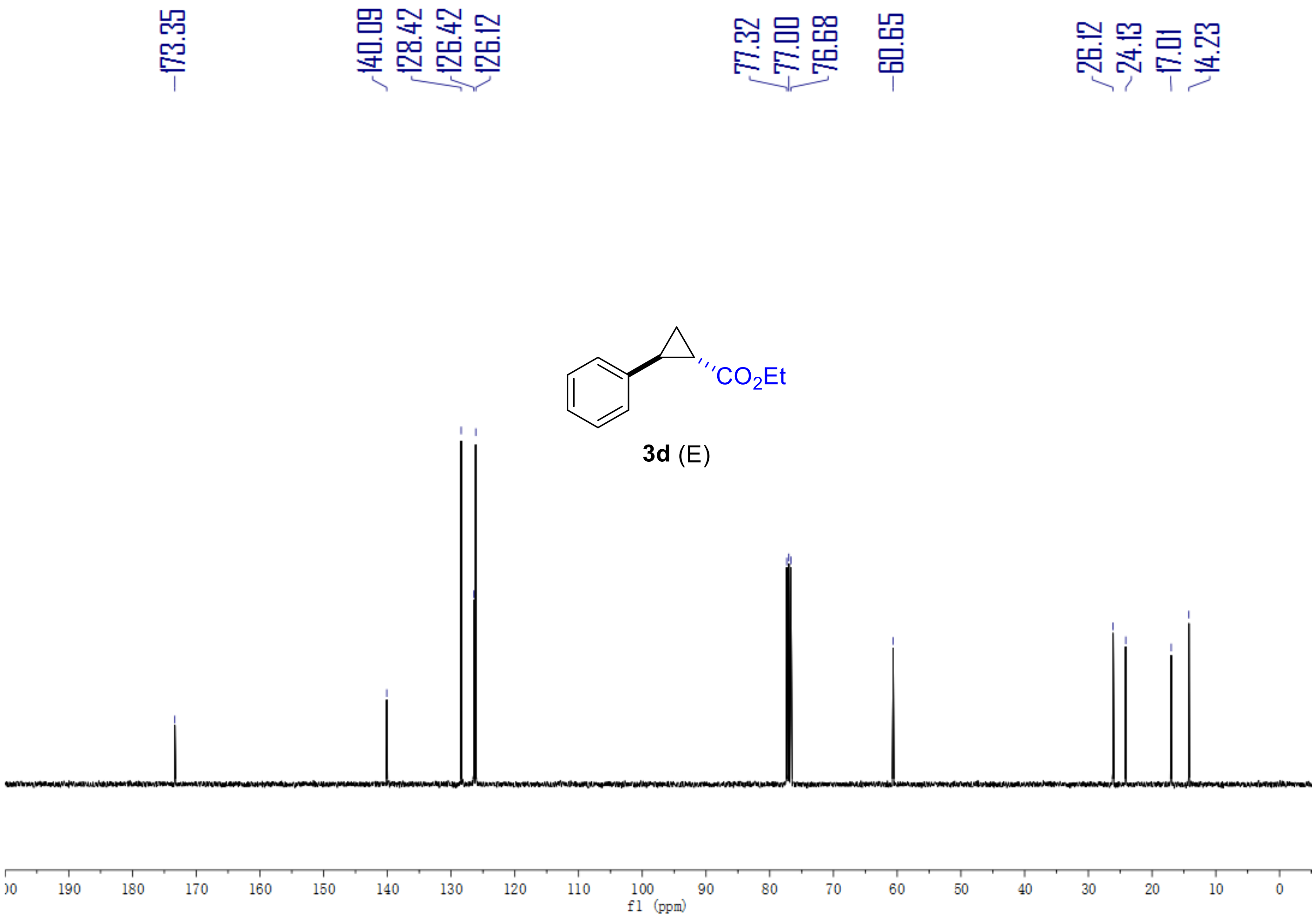

Supplementary Figure 18. ^{13}C NMR of **3d** (E)

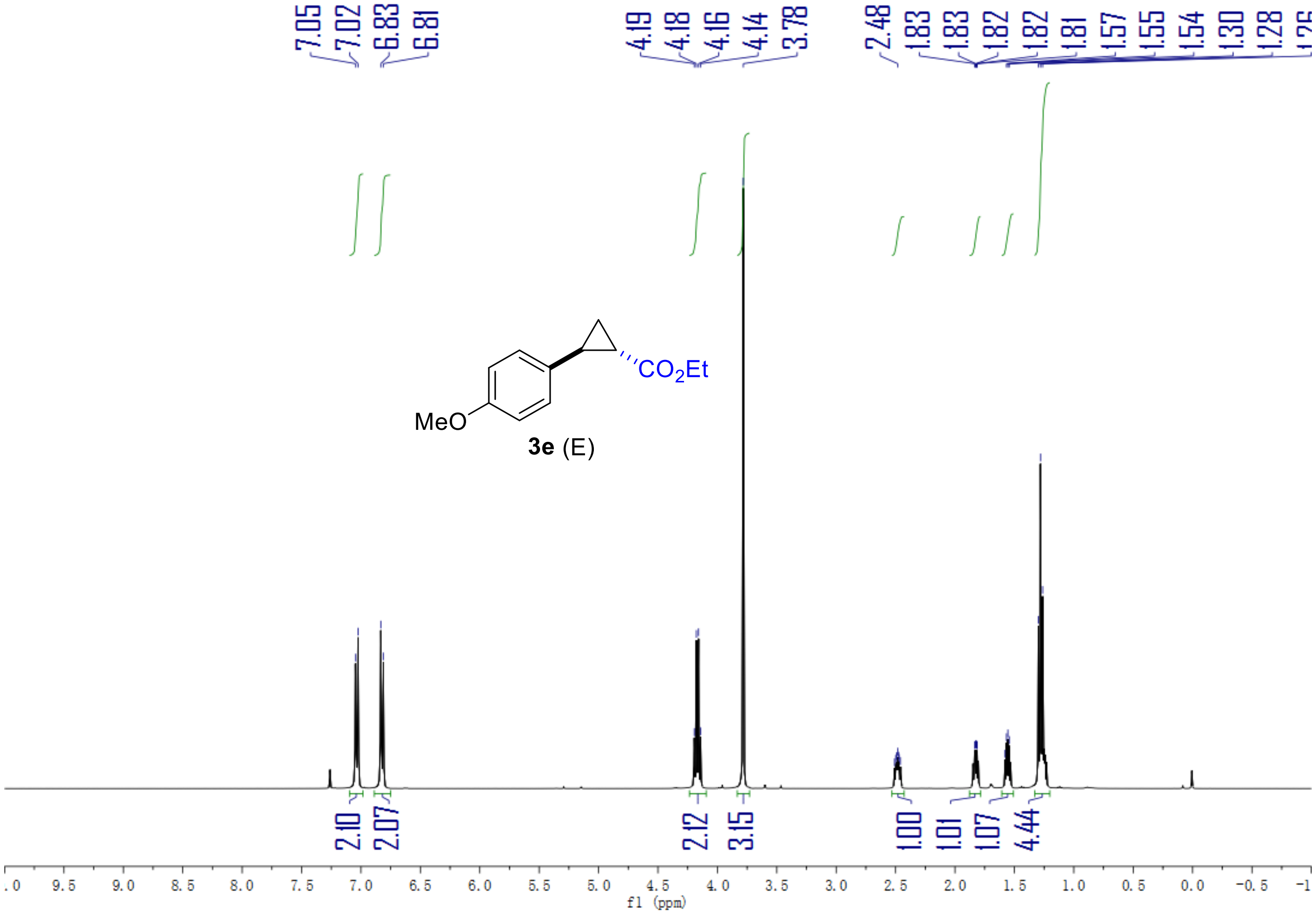

Supplementary Figure 19. ^1H NMR of **3e** (E)

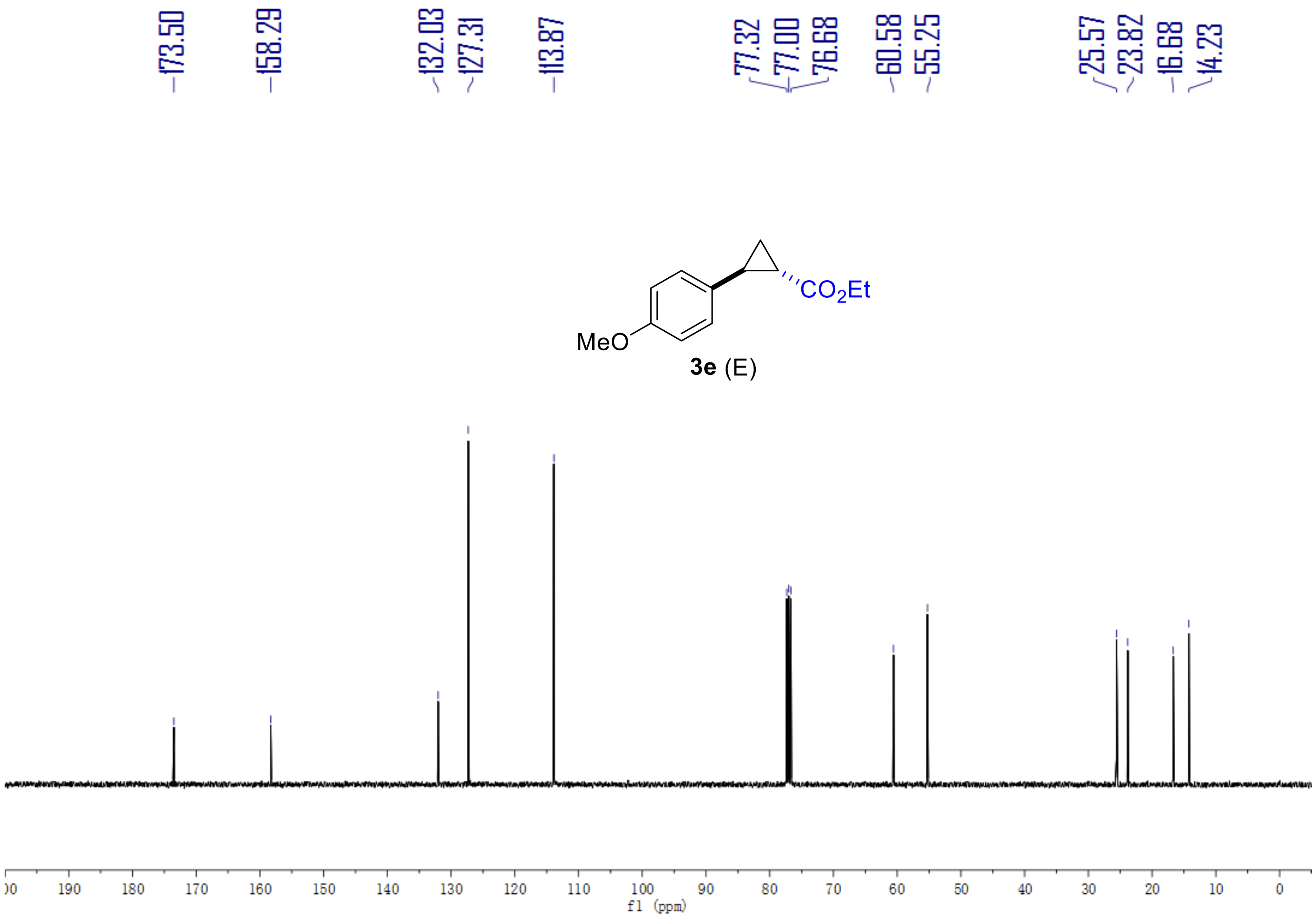

Supplementary Figure 20. ^{13}C NMR of **3e** (E)

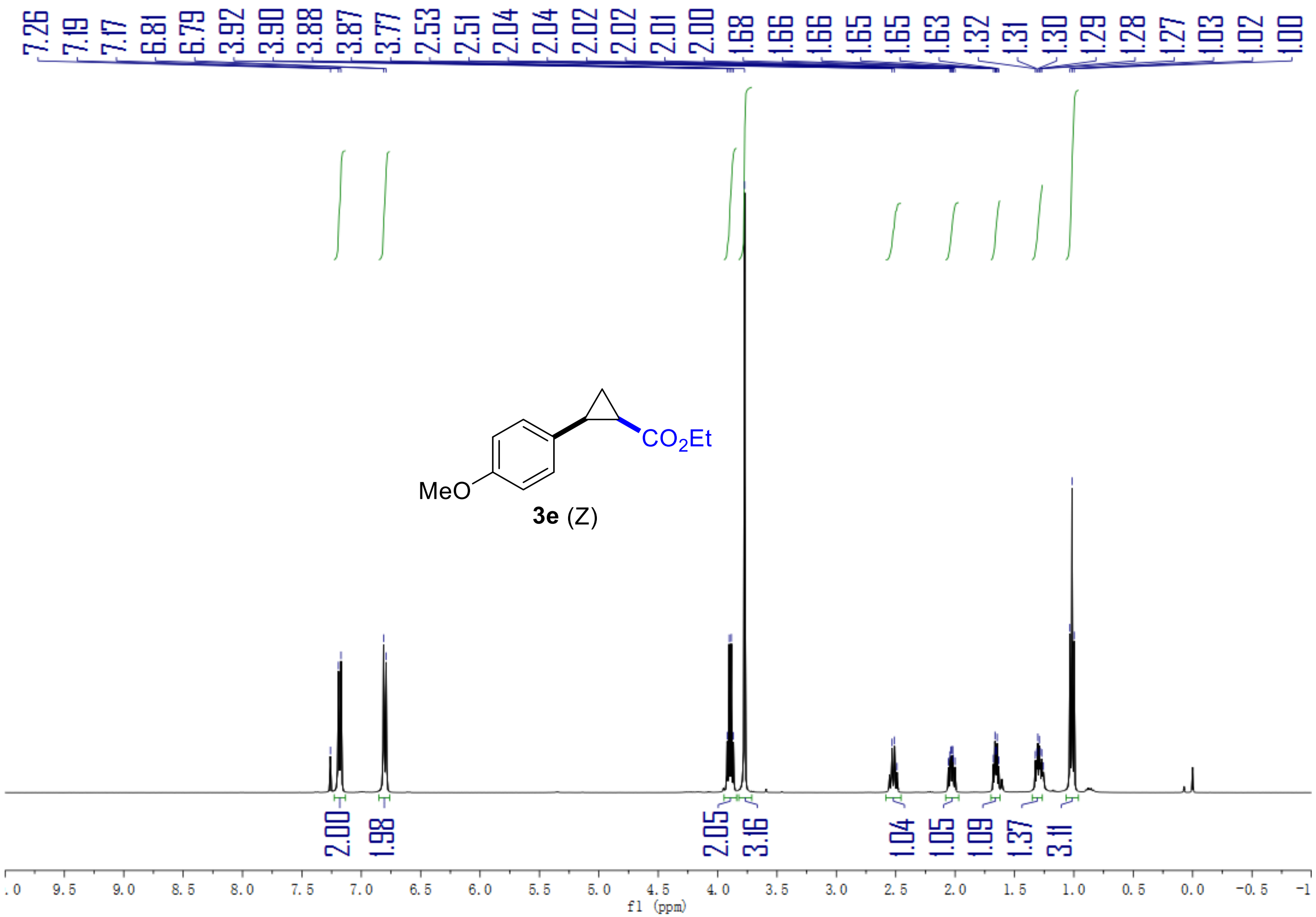

Supplementary Figure 21. ^{1}H NMR of **3e** (Z)

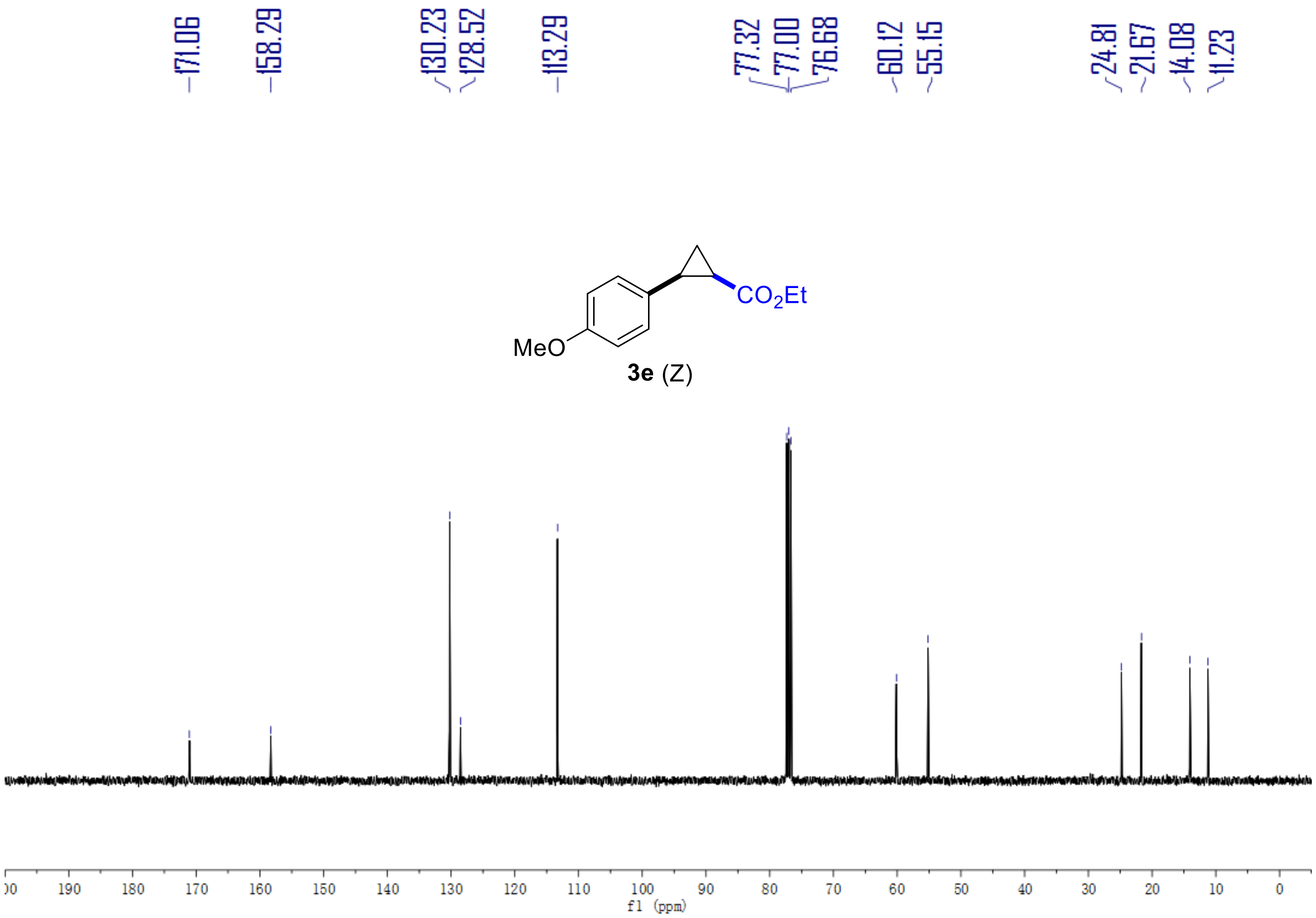

Supplementary Figure 22. ^{13}C NMR of **3e** (E)

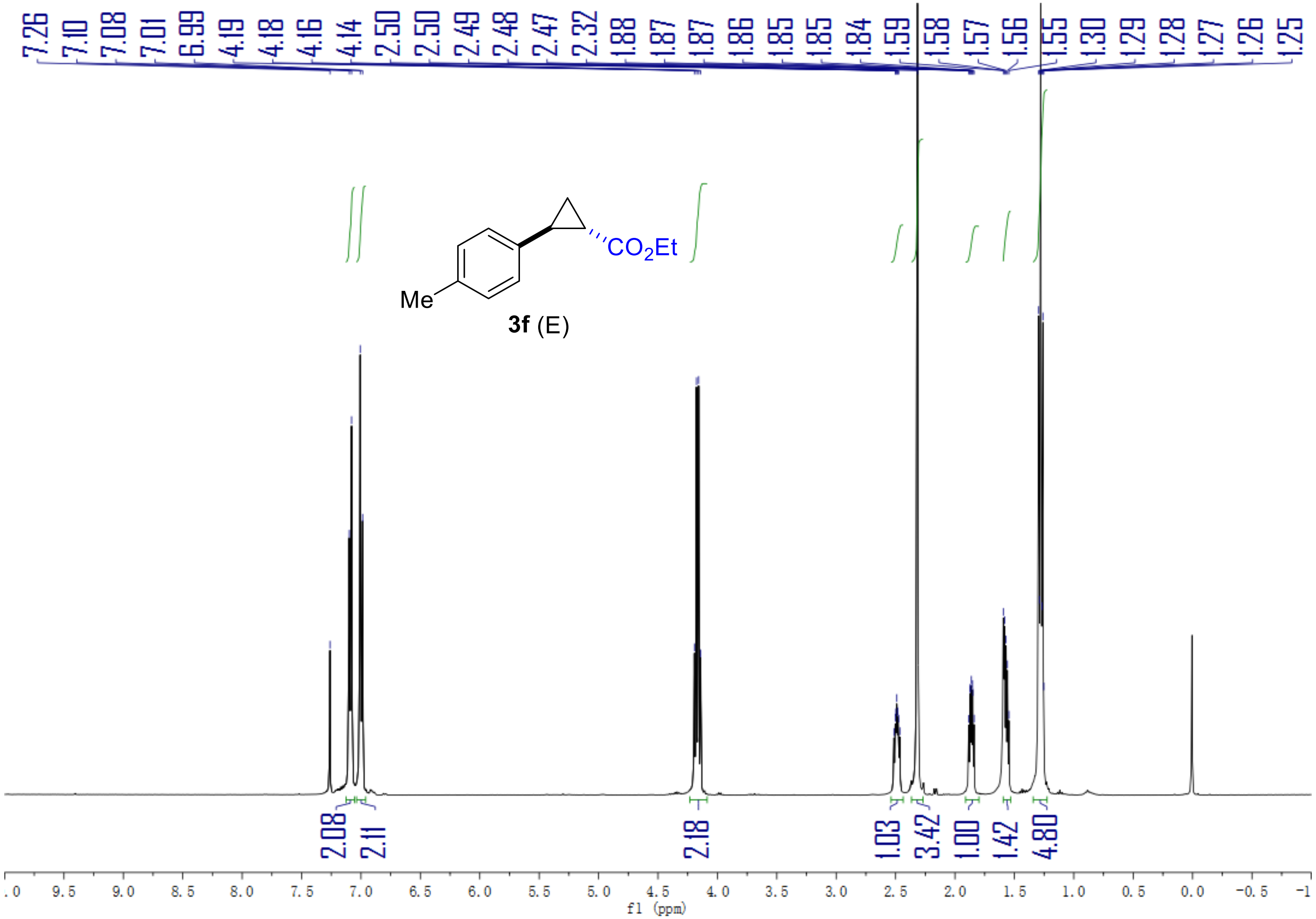

Supplementary Figure 23. ^{1}H NMR of **3f** (E)

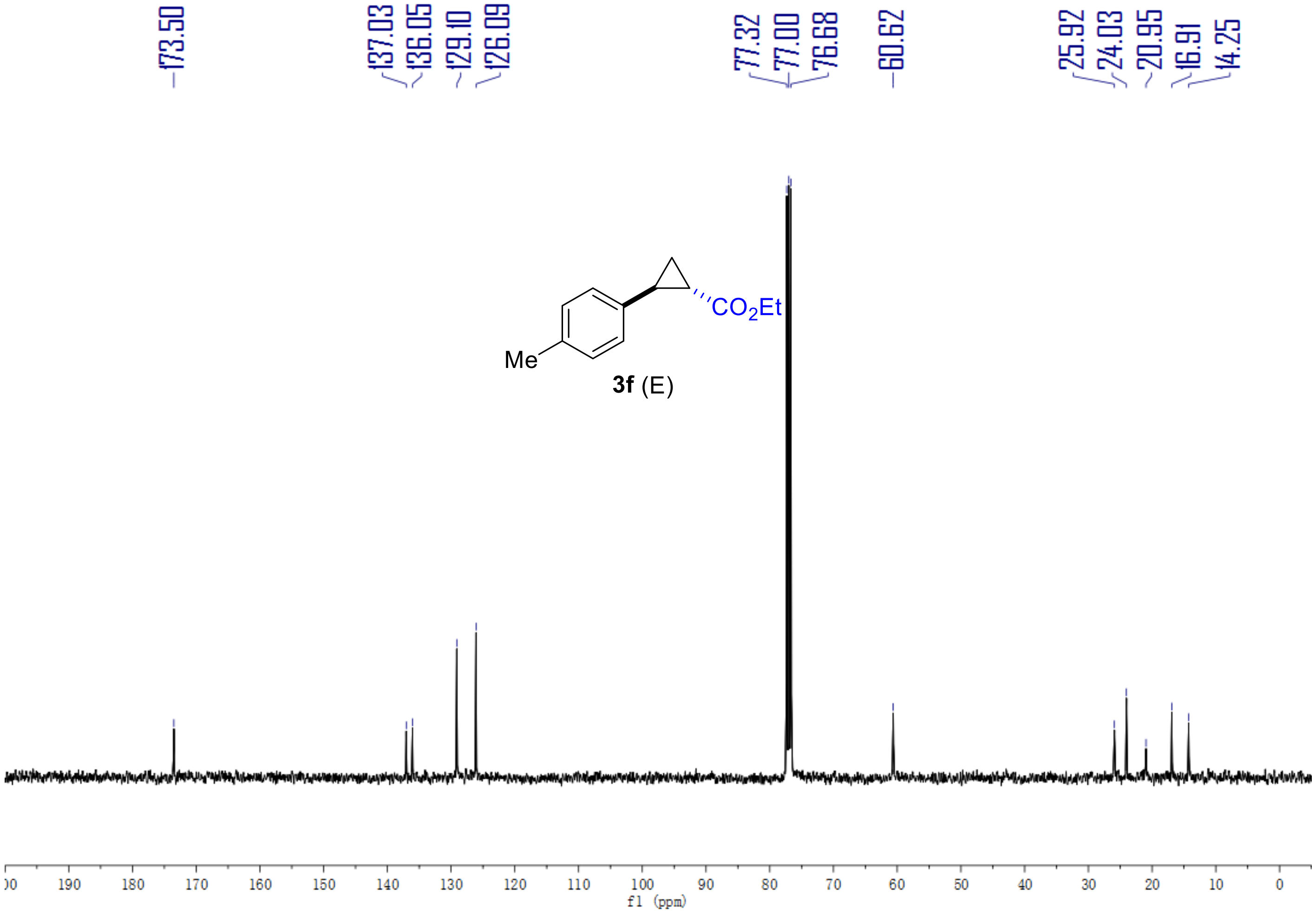

Supplementary Figure 24. ^{13}C NMR of **3f** (E)

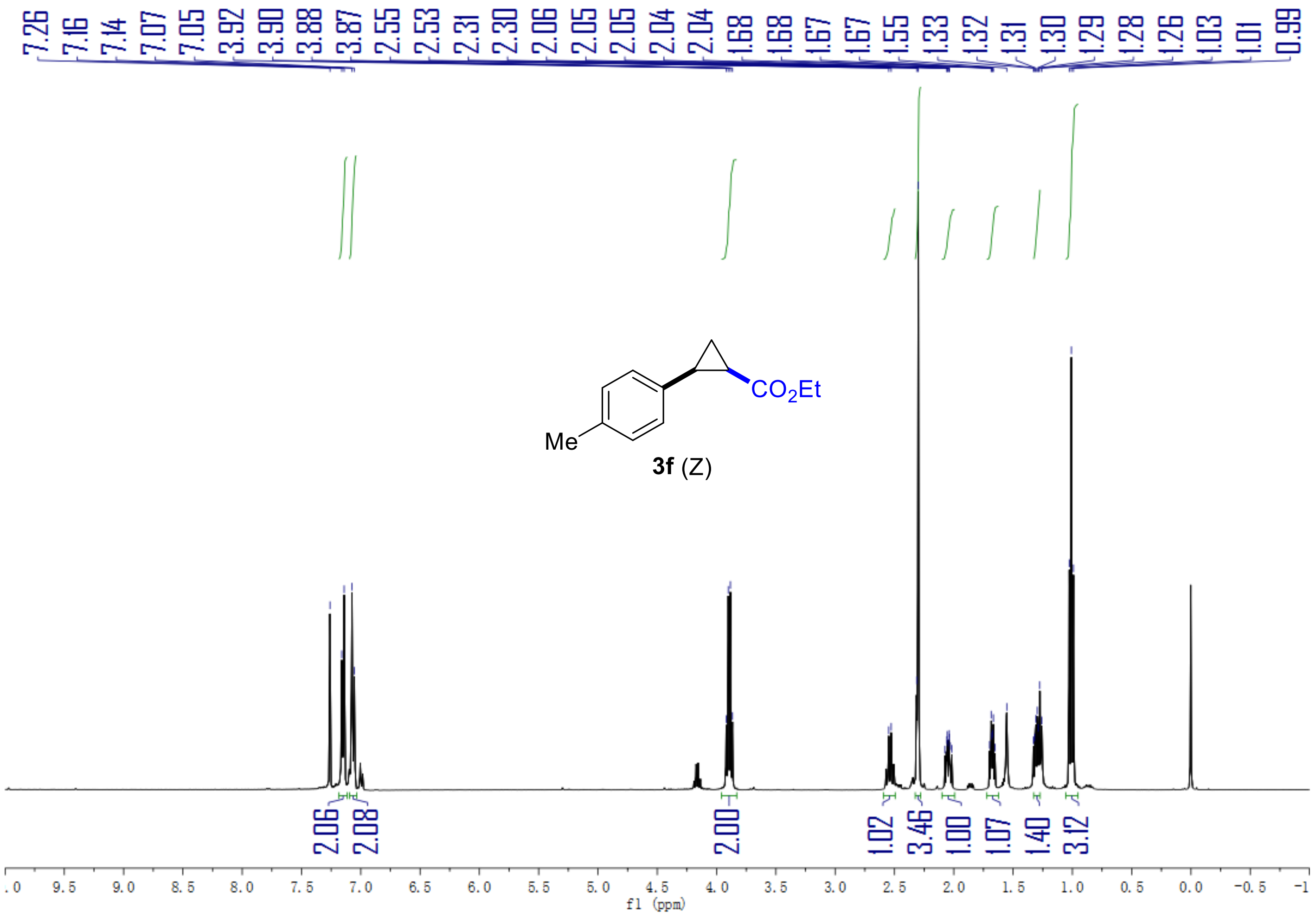

Supplementary Figure 25. ^{1}H NMR of **3f** (Z)

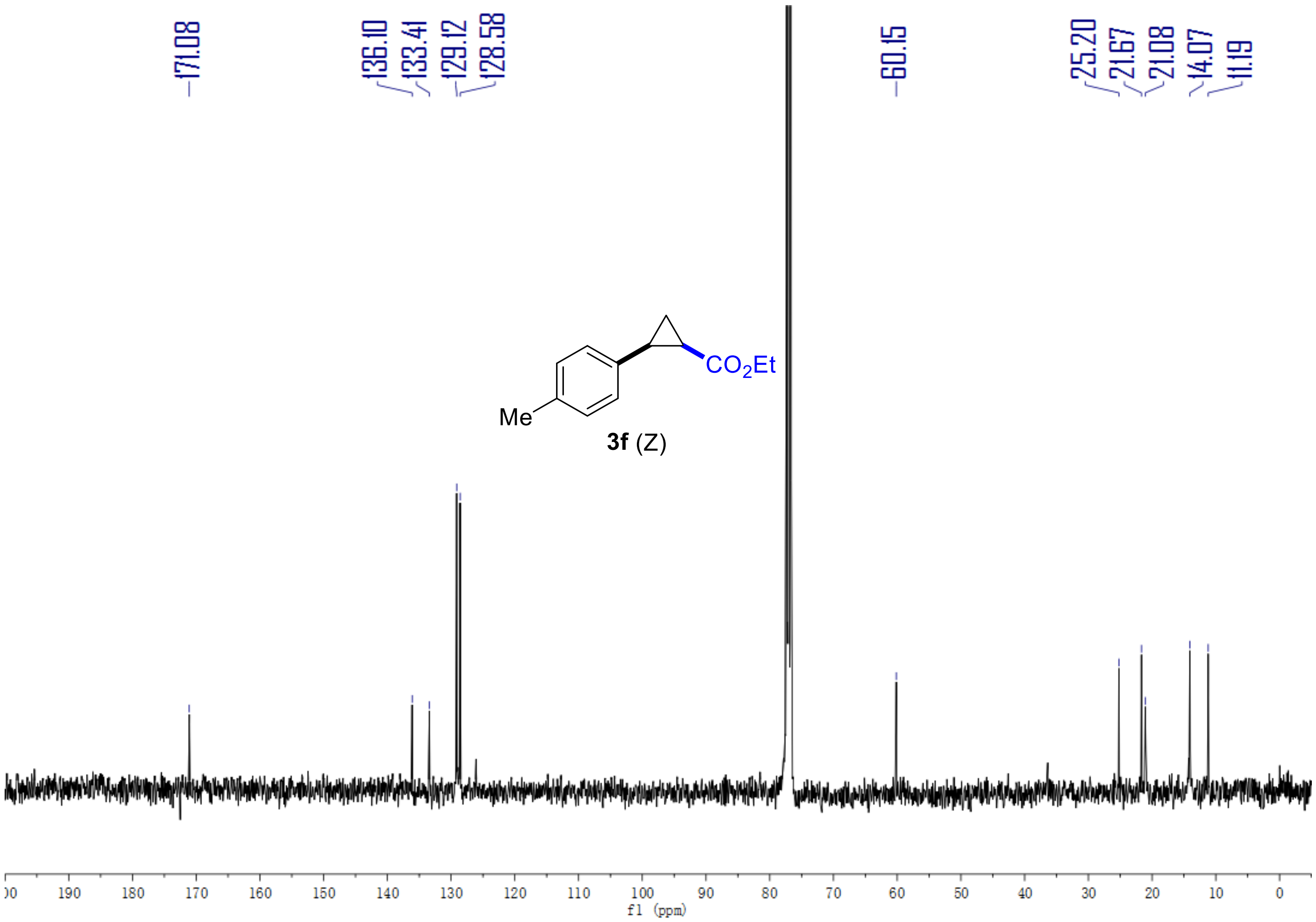

Supplementary Figure 26. ^{13}C NMR of **3f** (Z)

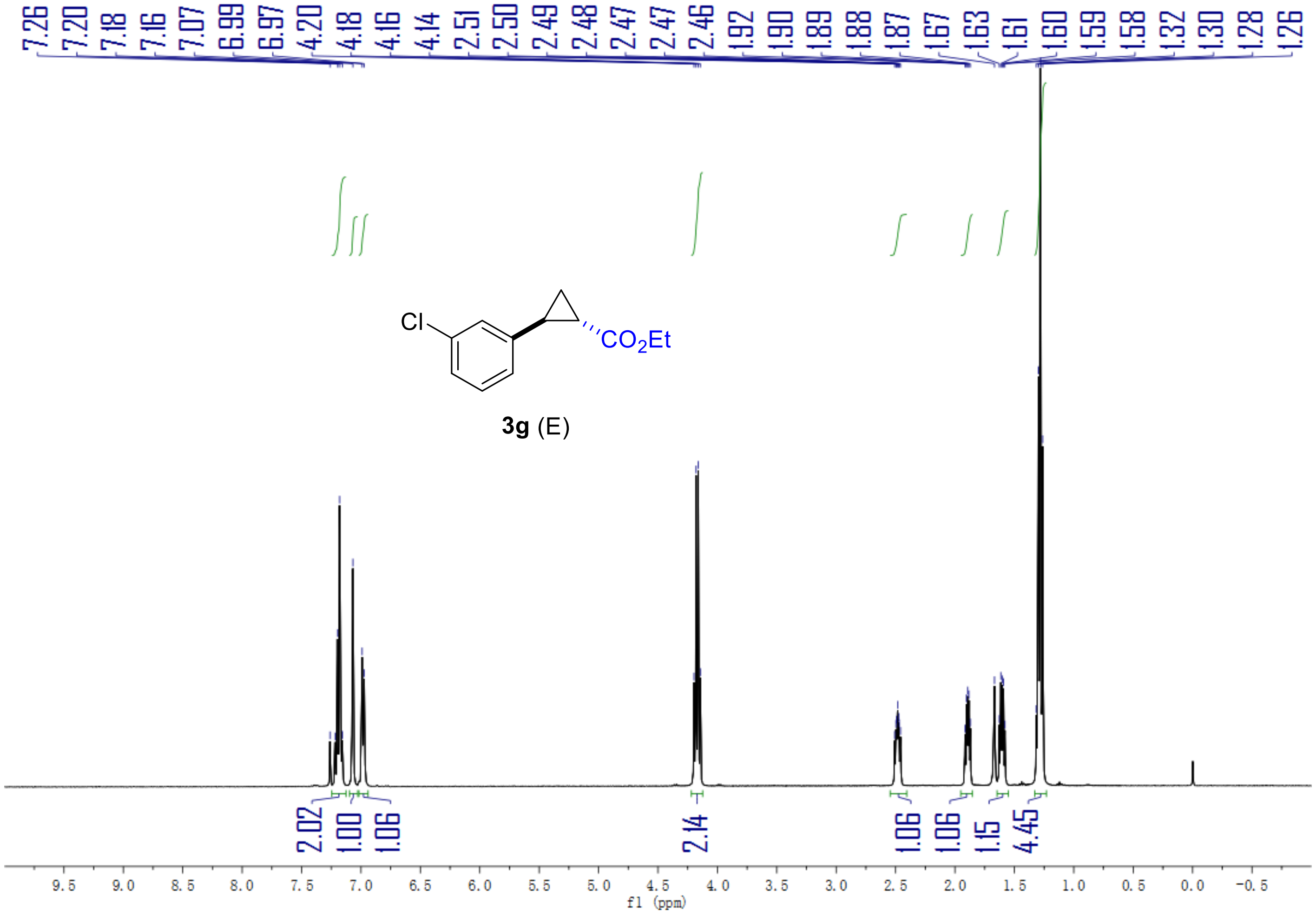

Supplementary Figure 27. ^{1}H NMR of **3g** (E)

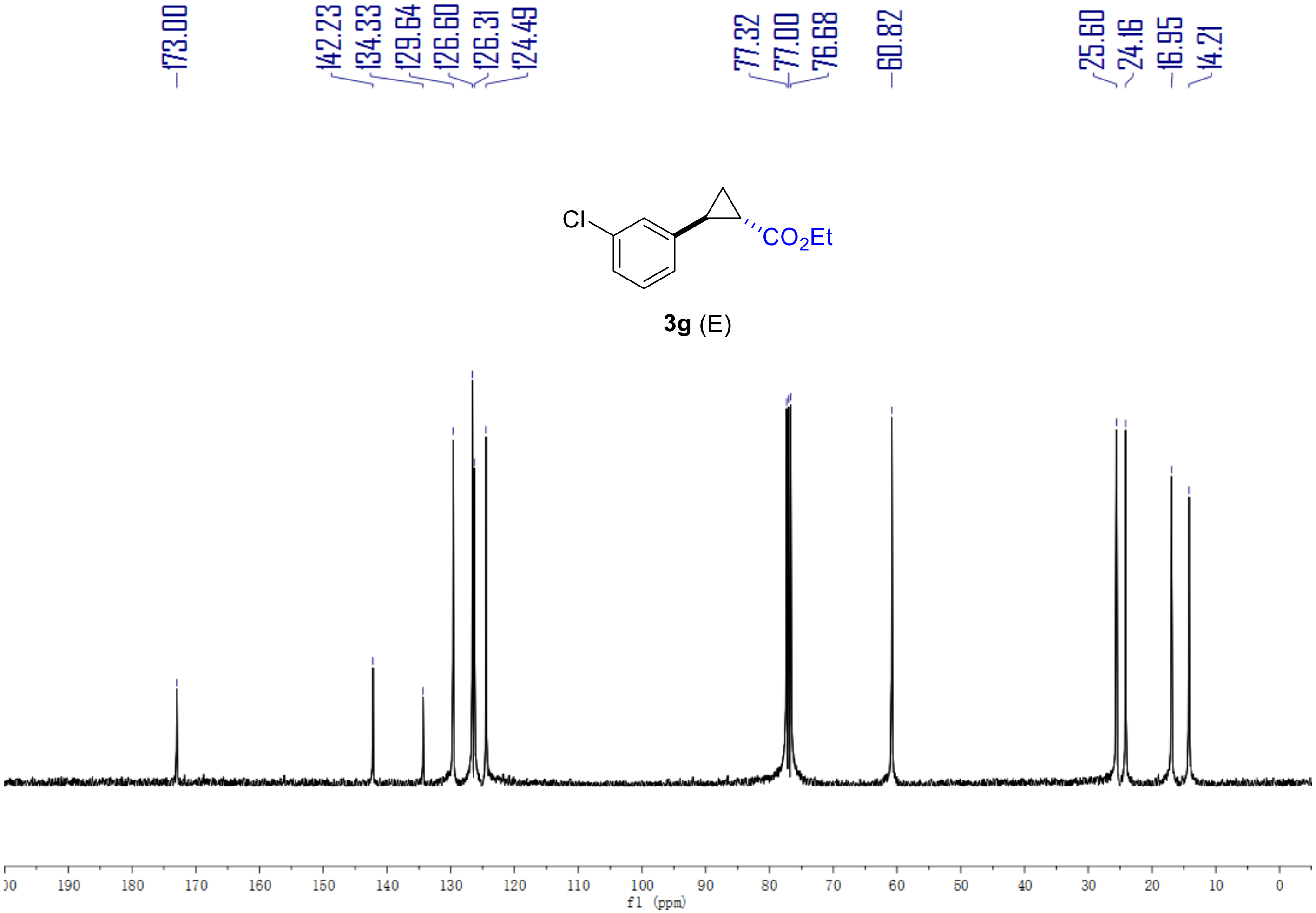

Supplementary Figure 28. ^{13}C NMR of **3g** (E)

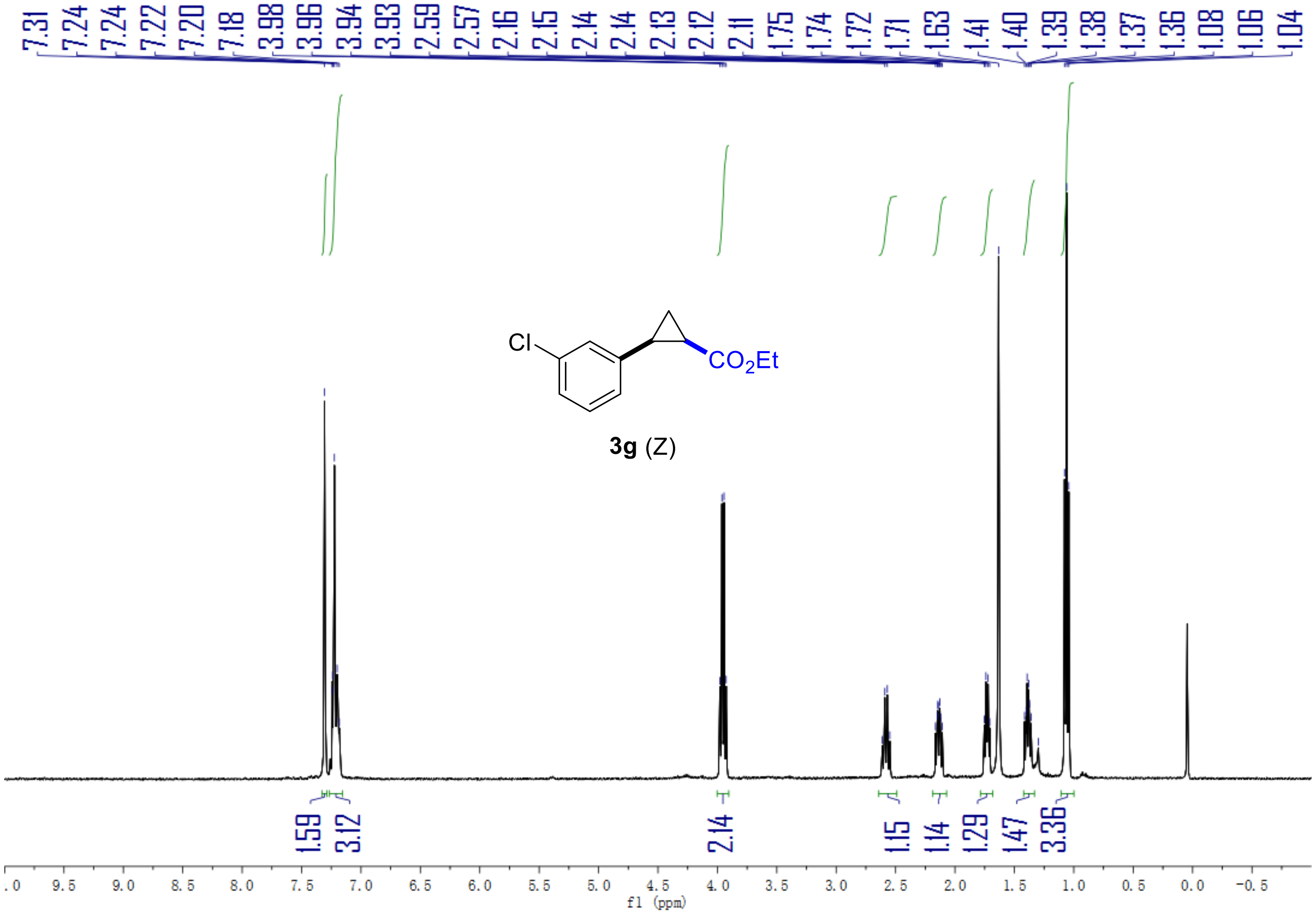

Supplementary Figure 29. ^{1}H NMR of **3g** (Z)

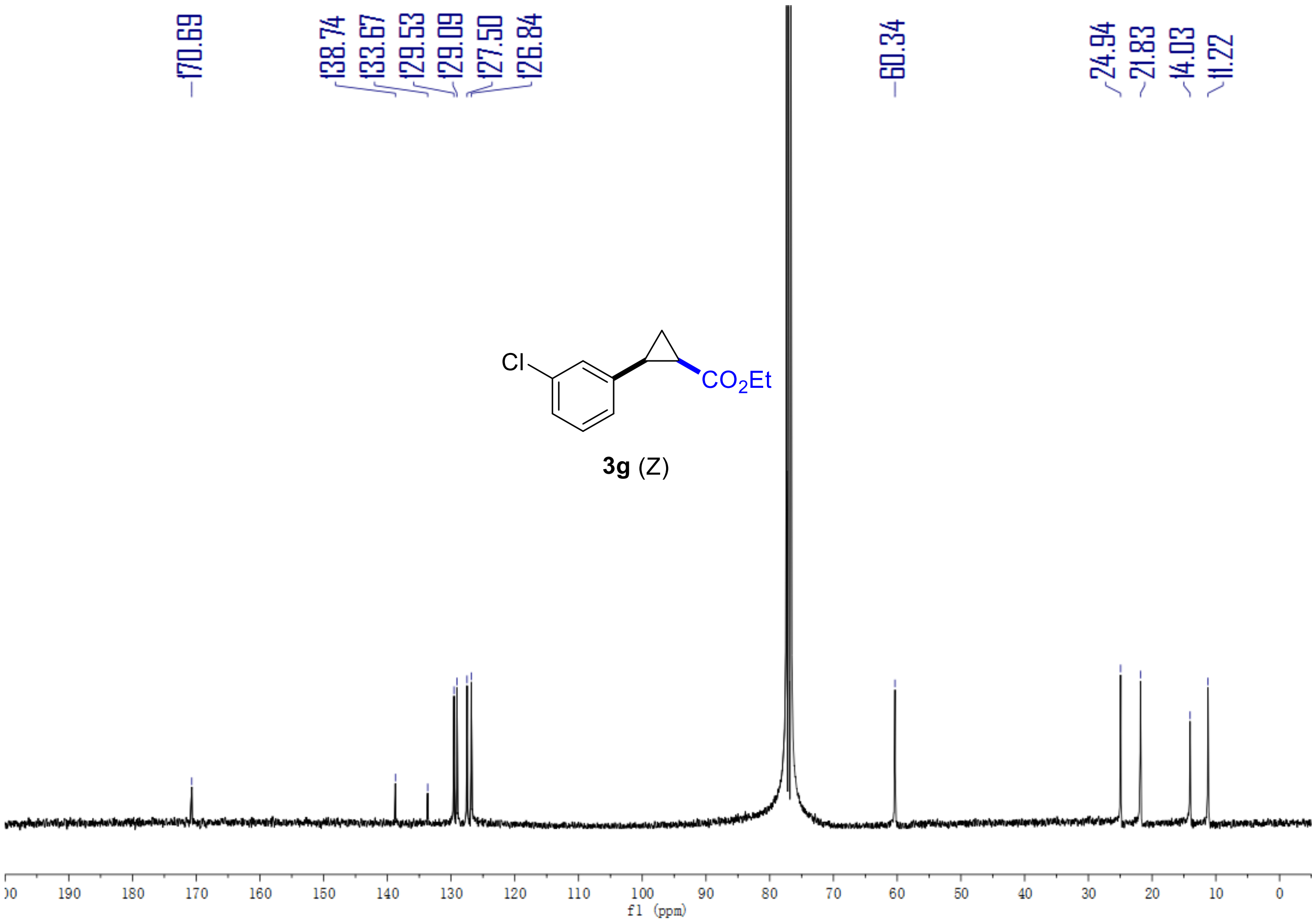

Supplementary Figure 30. ^{13}C NMR of **3g** (Z)

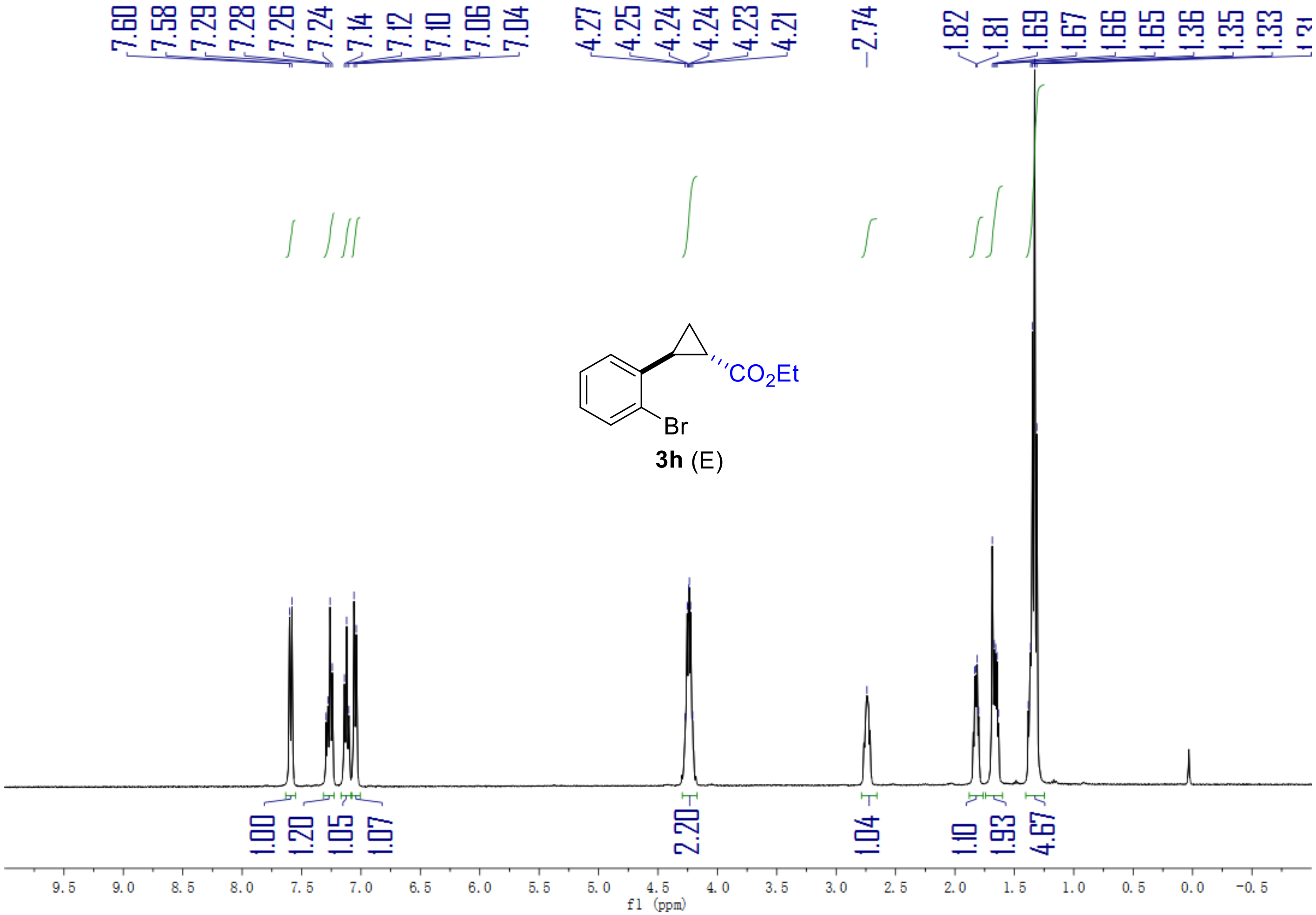

Supplementary Figure 31. ^{1}H NMR of **3h** (E)

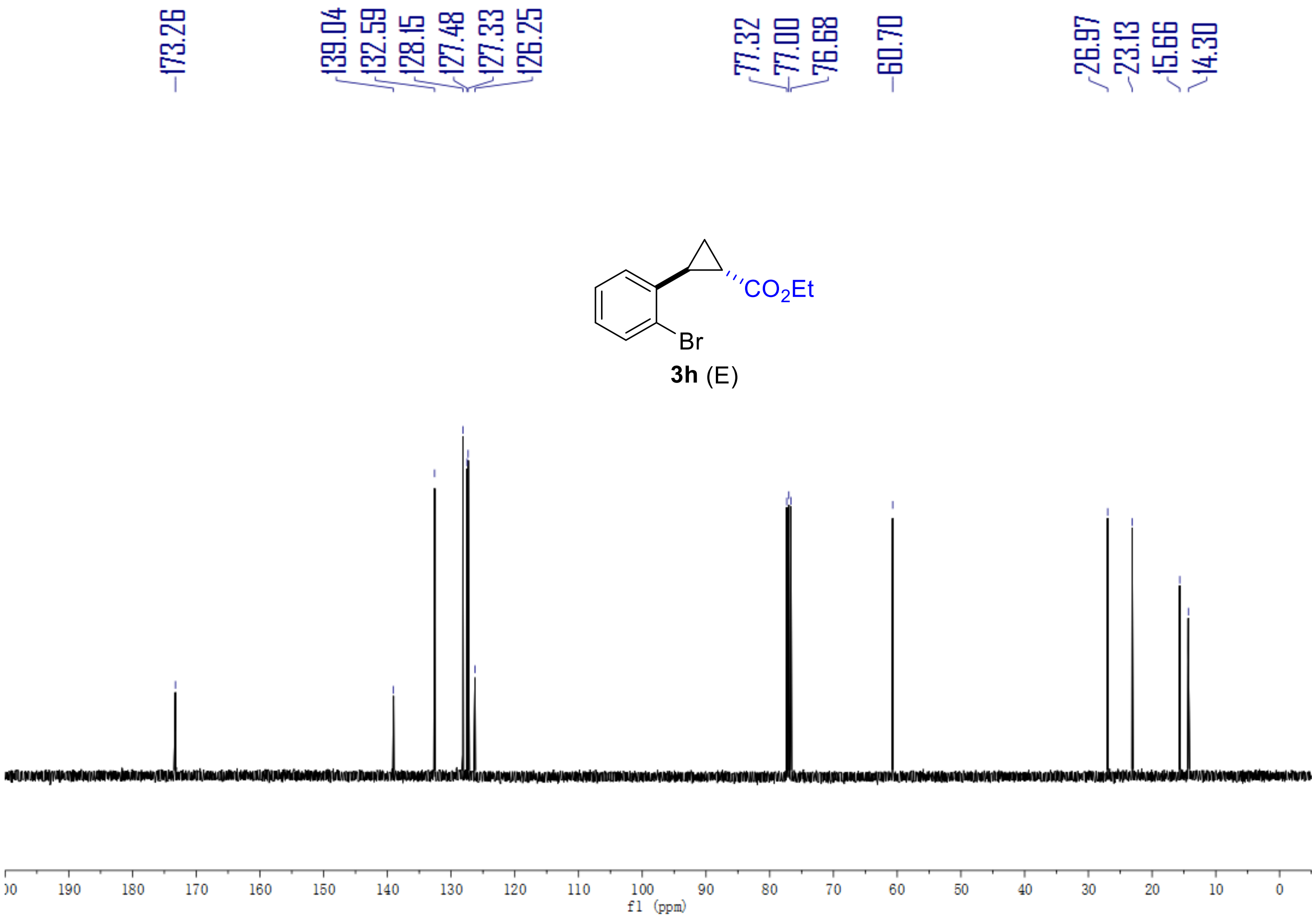

Supplementary Figure 32. ^{13}C NMR of **3h** (E)

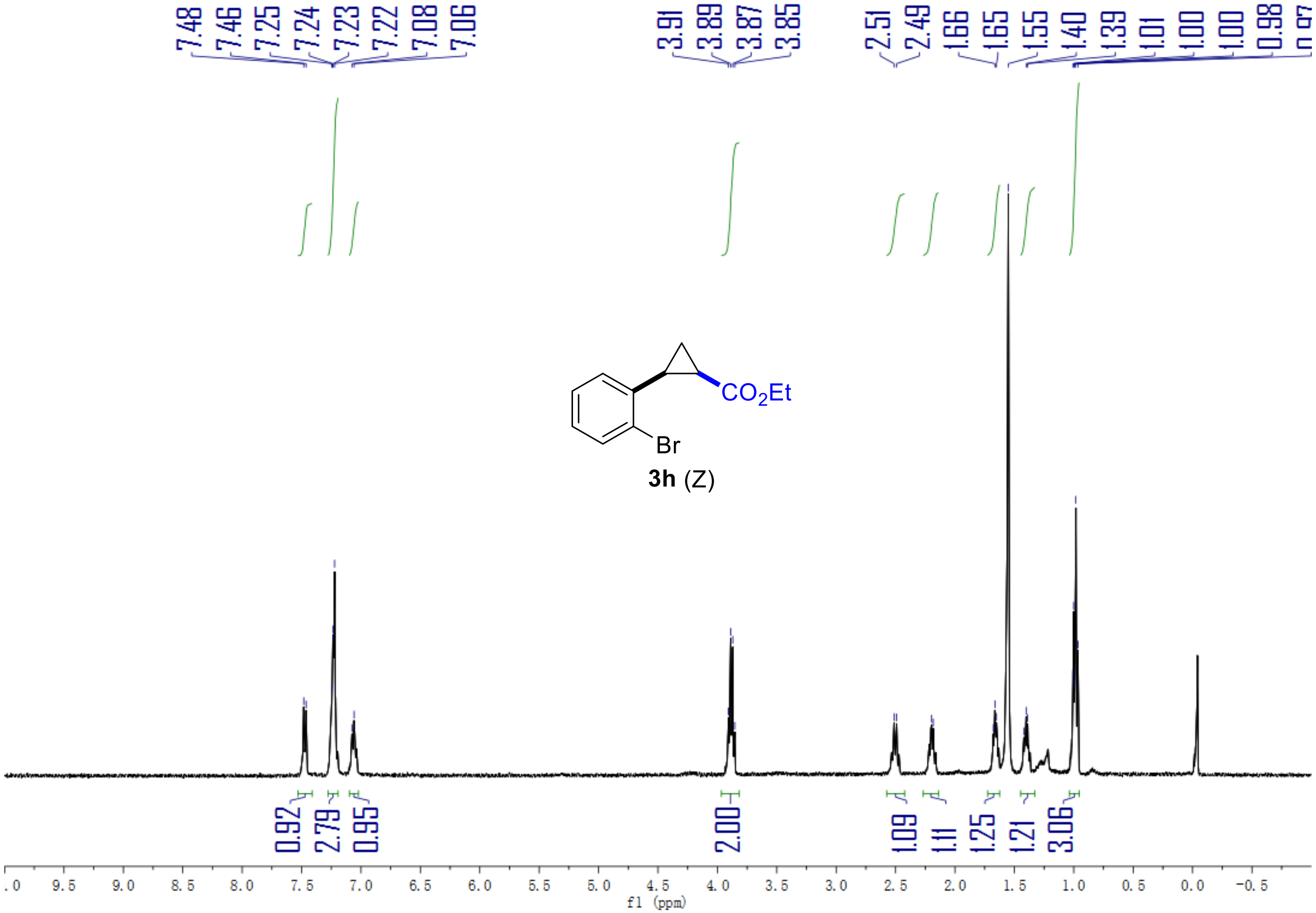

Supplementary Figure 33. ^{1}H NMR of **3h** (Z)

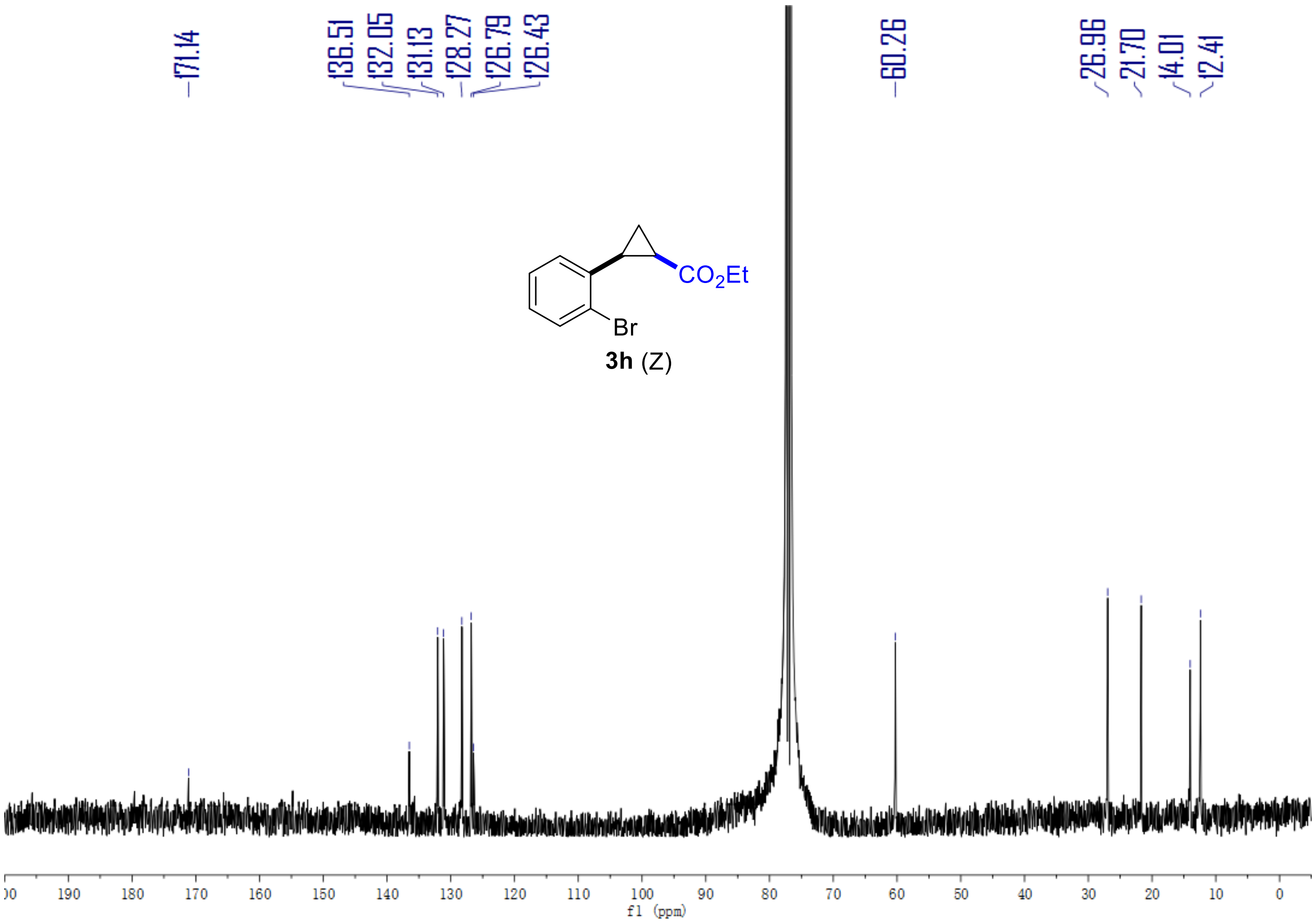

Supplementary Figure 34. ^{13}C NMR of **3h** (Z)

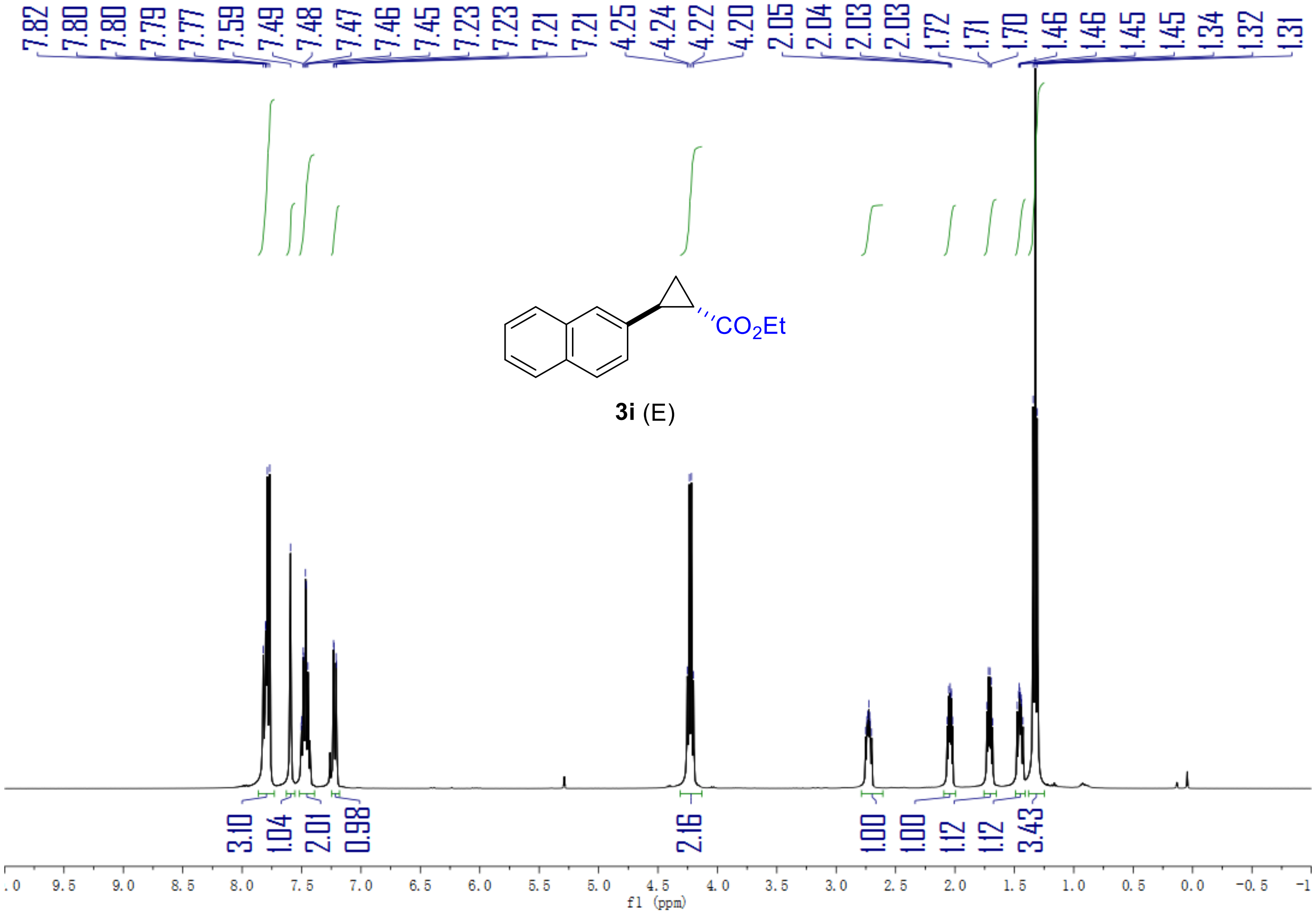

Supplementary Figure 35. ^{1}H NMR of **3i** (E)

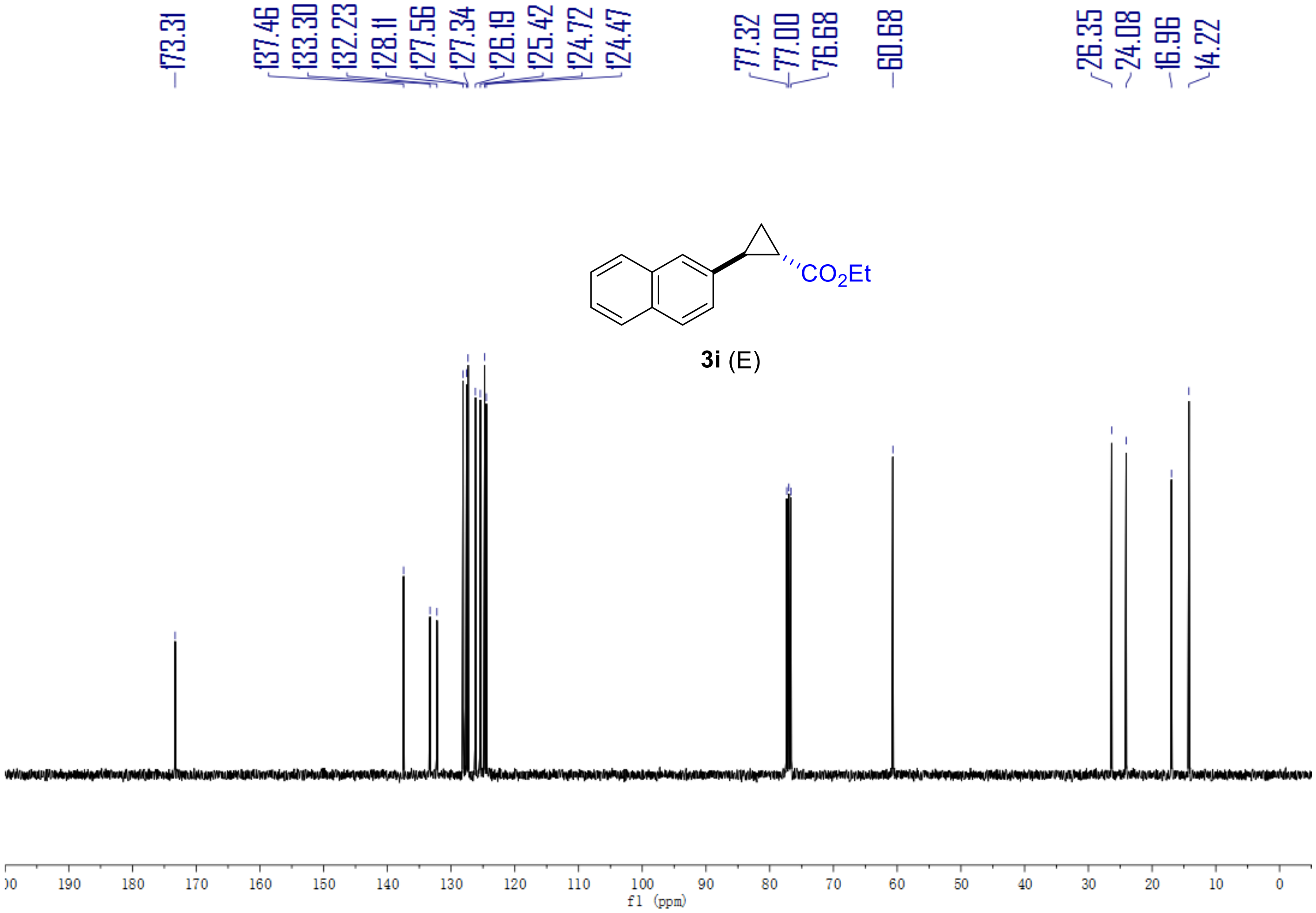

Supplementary Figure 36. ^{13}C NMR of **3i** (E)

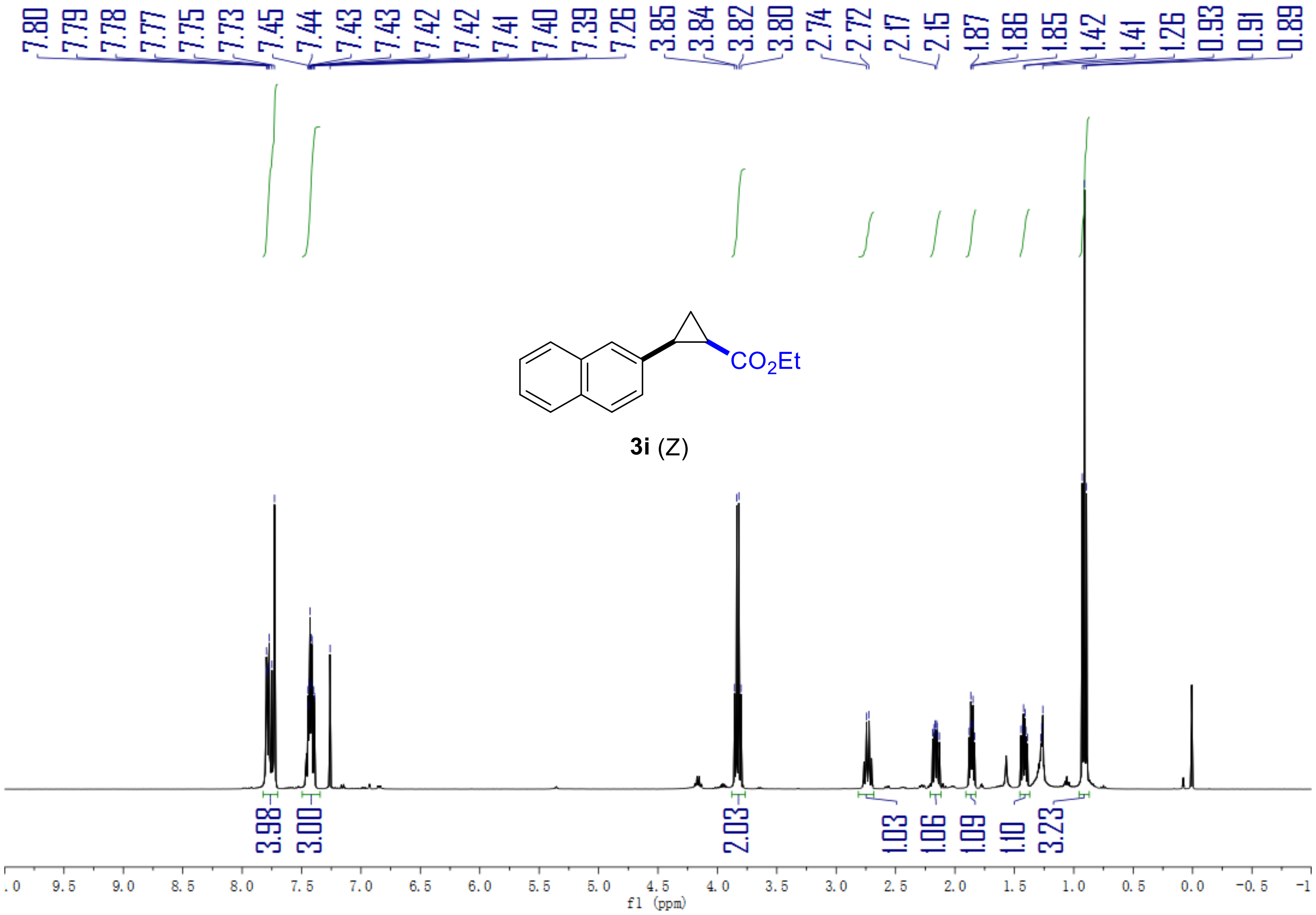

Supplementary Figure 37. ^{1}H NMR of **3i** (Z)

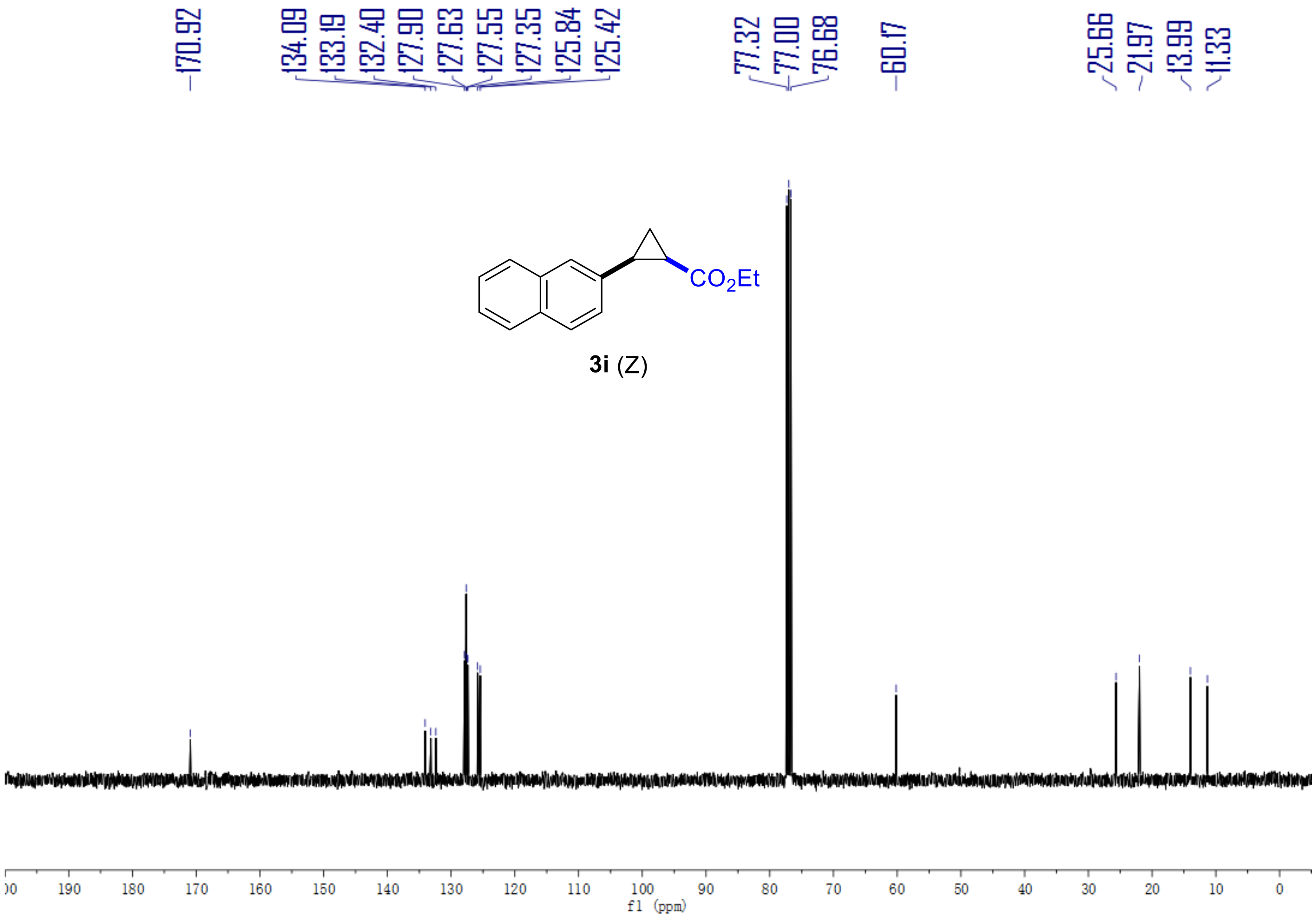

Supplementary Figure 38. ^{13}C NMR of **3i** (Z)

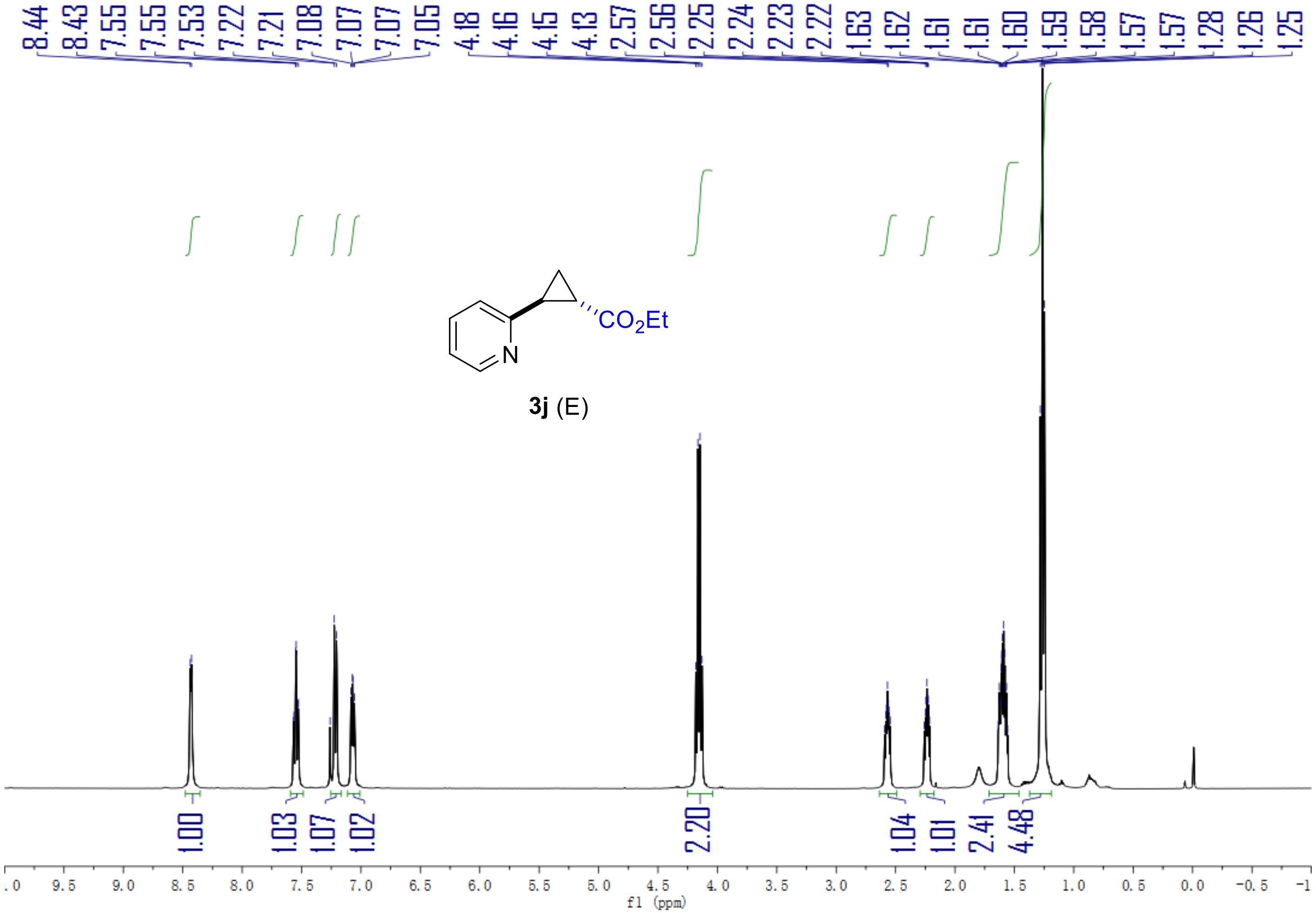

Supplementary Figure 39. ^{1}H NMR of **3j** (E)

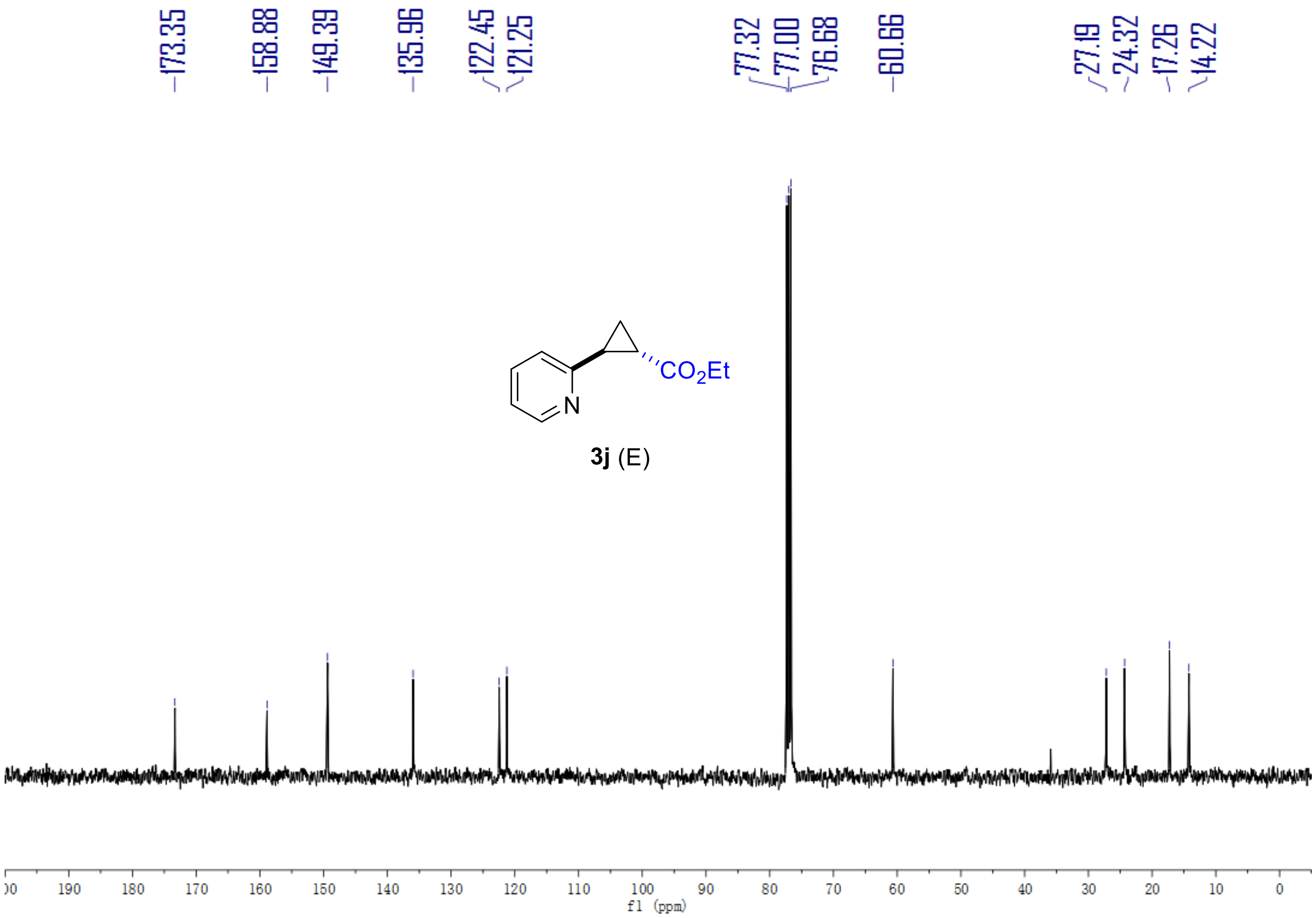

Supplementary Figure 40. ^{13}C NMR of **3j** (E)

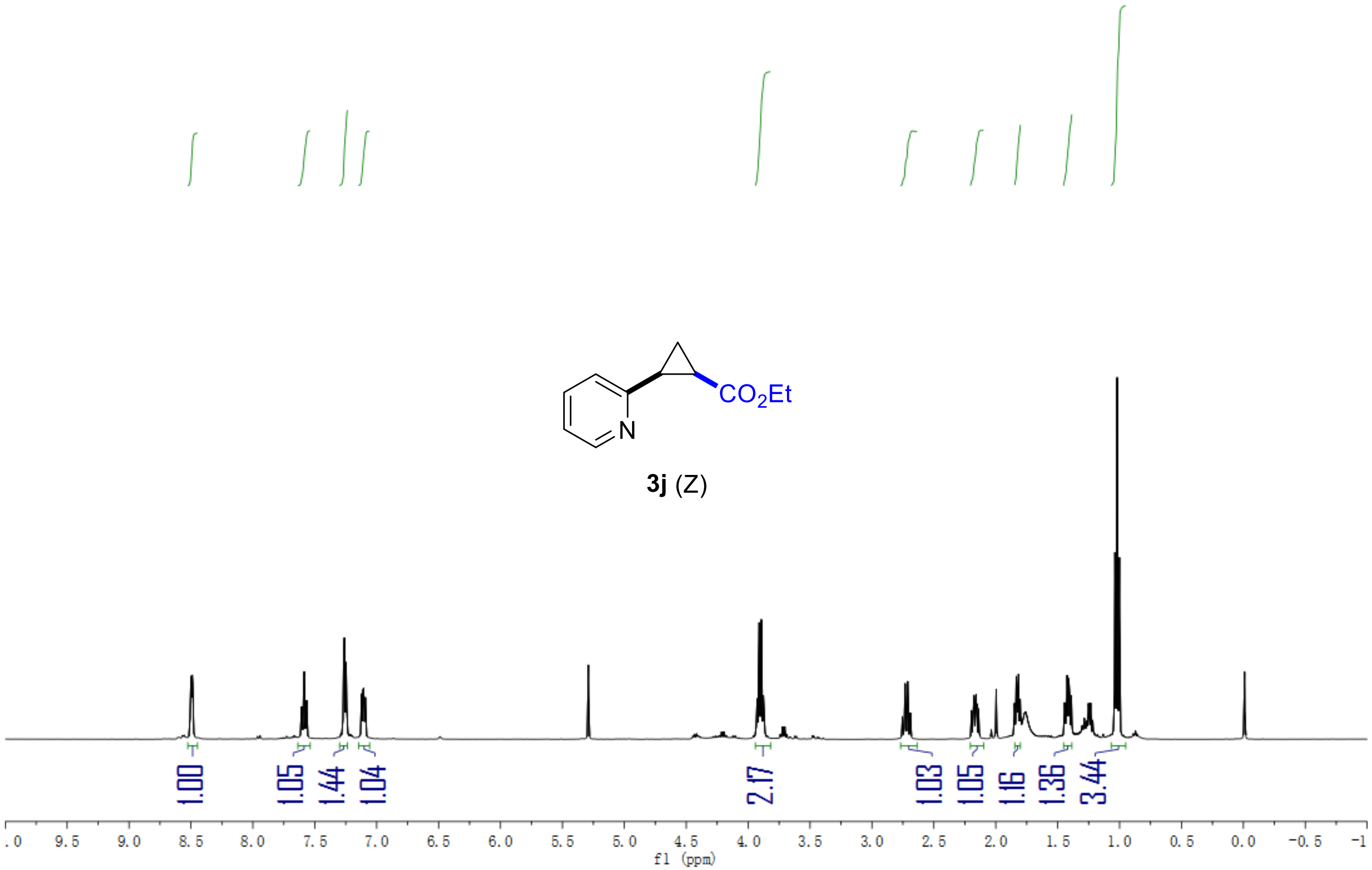

Supplementary Figure 41. ^{1}H NMR of **3j** (Z)

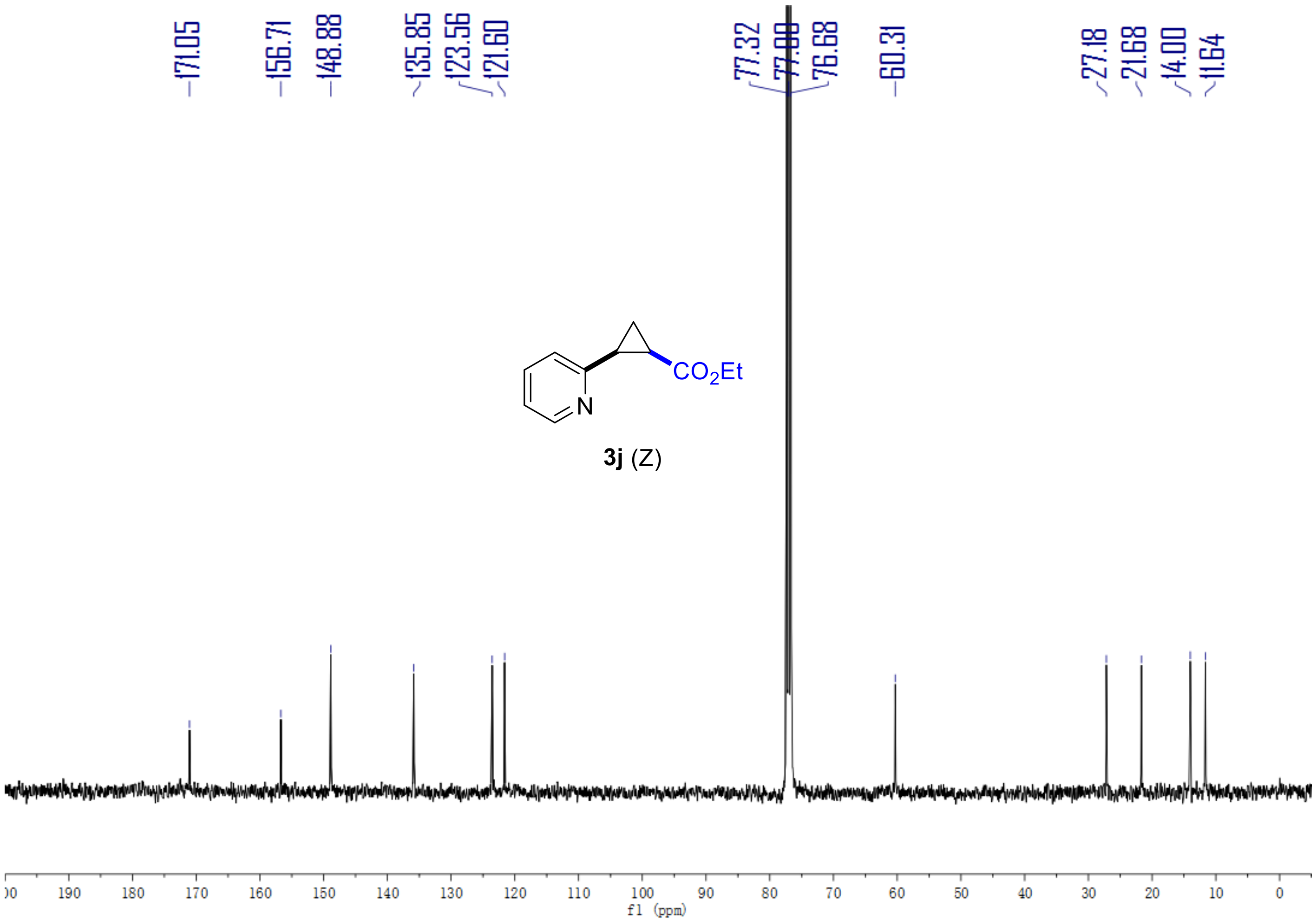

Supplementary Figure 42. ^{13}C NMR of **3j** (Z)

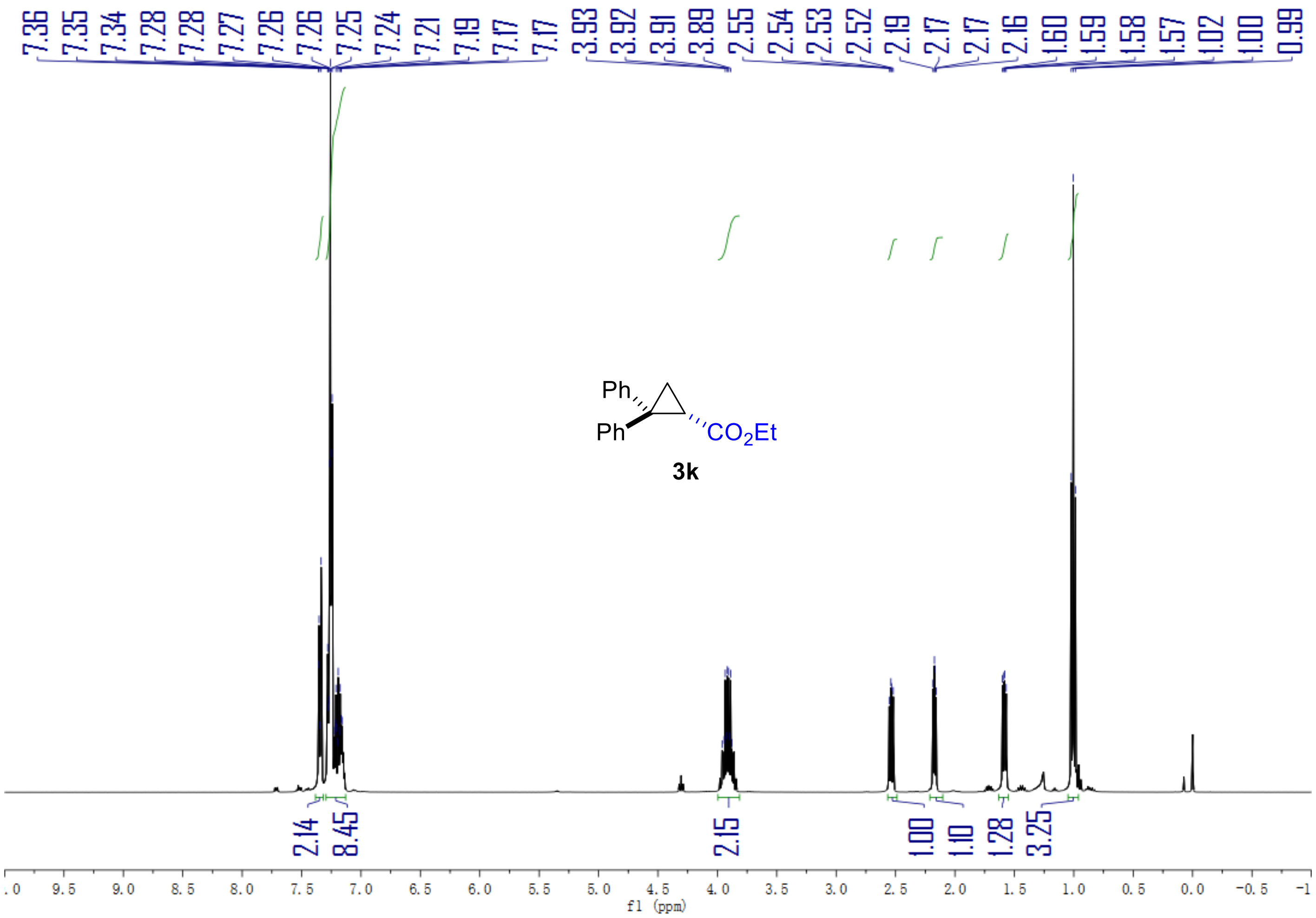

Supplementary Figure 43. ^{1}H NMR of **3k**

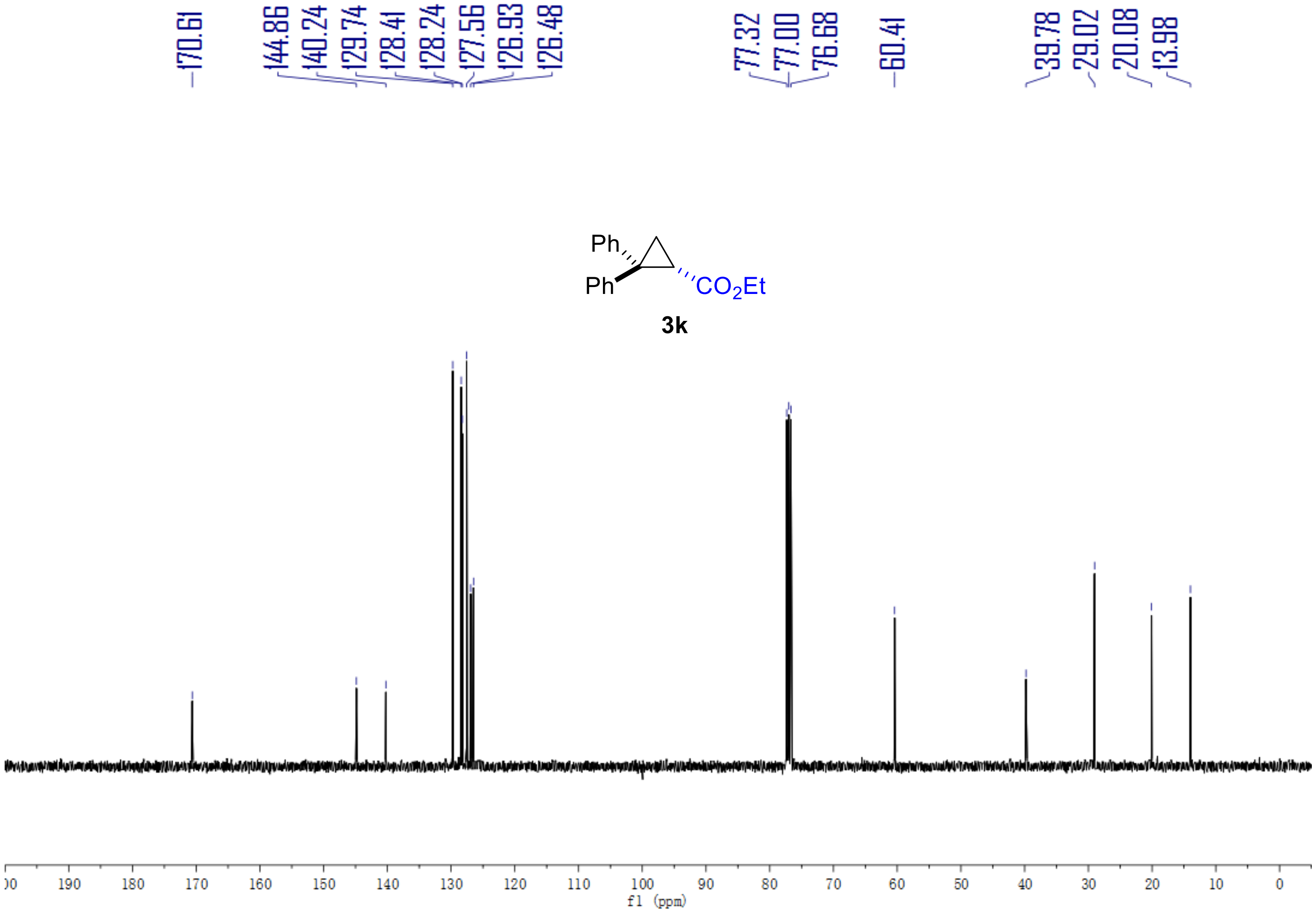

Supplementary Figure 44. ^{13}C NMR of **3k**

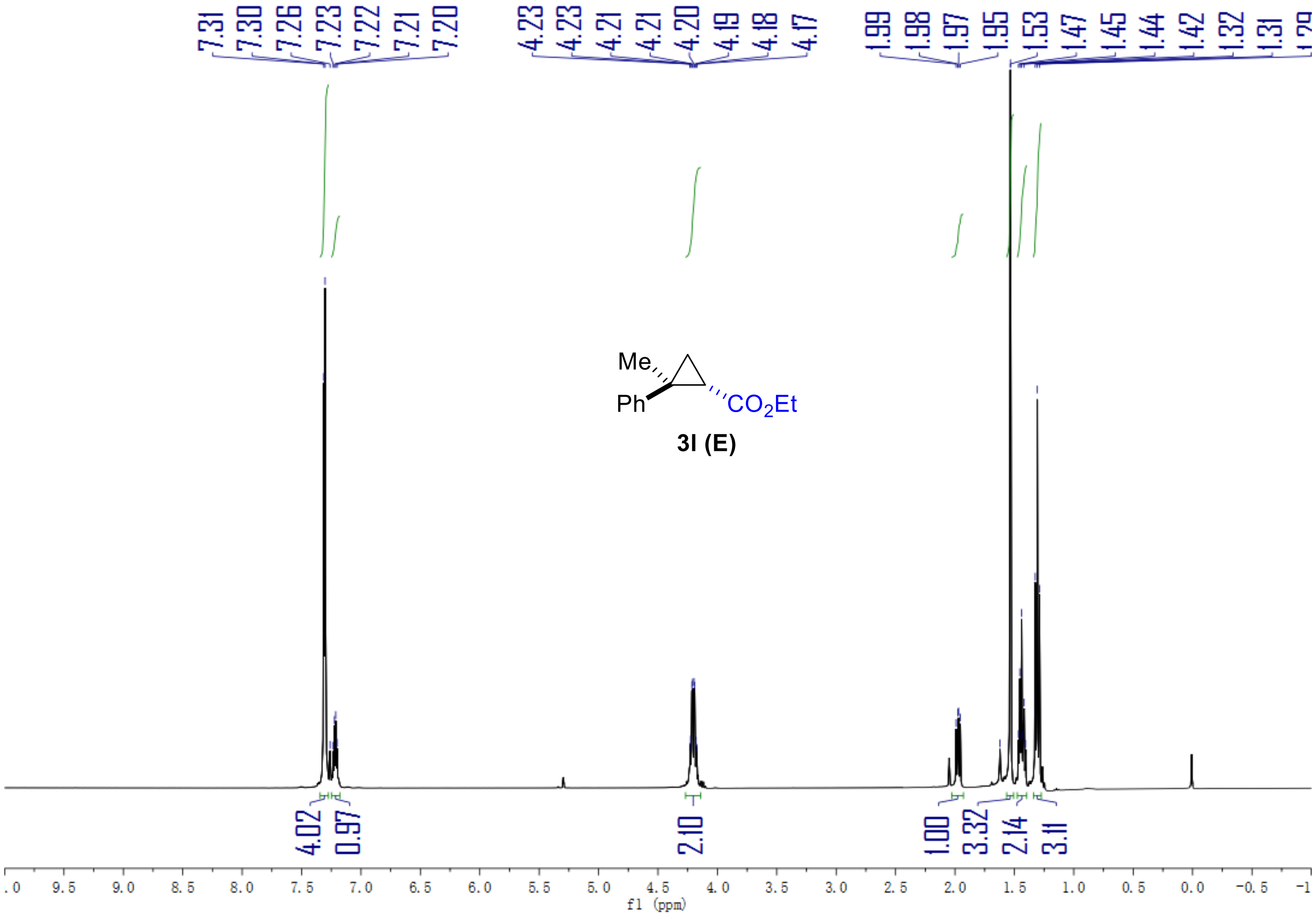

Supplementary Figure 45. ^{1}H NMR of **3l** (E)

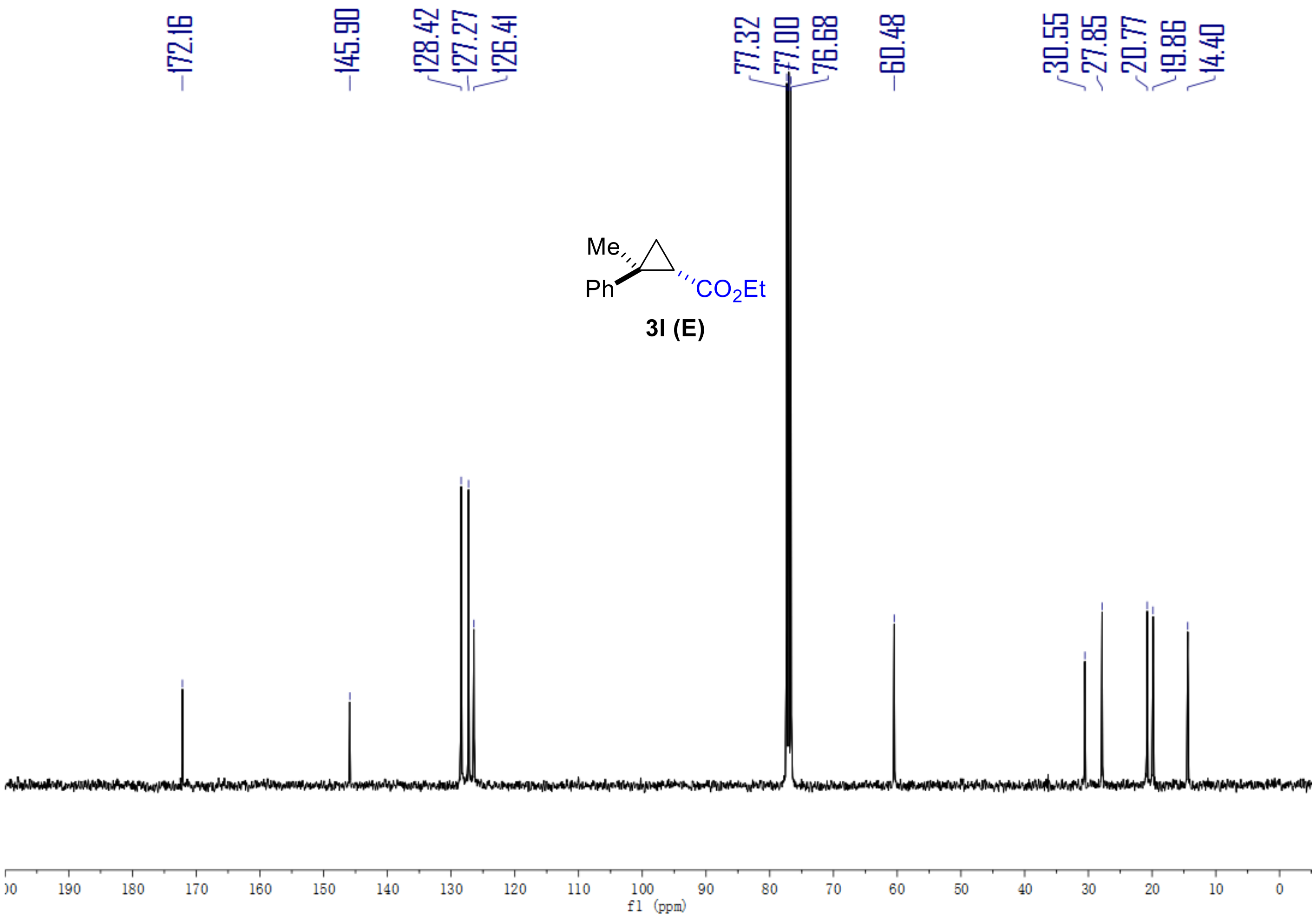

Supplementary Figure 46. ^{13}C NMR of **3l** (E)

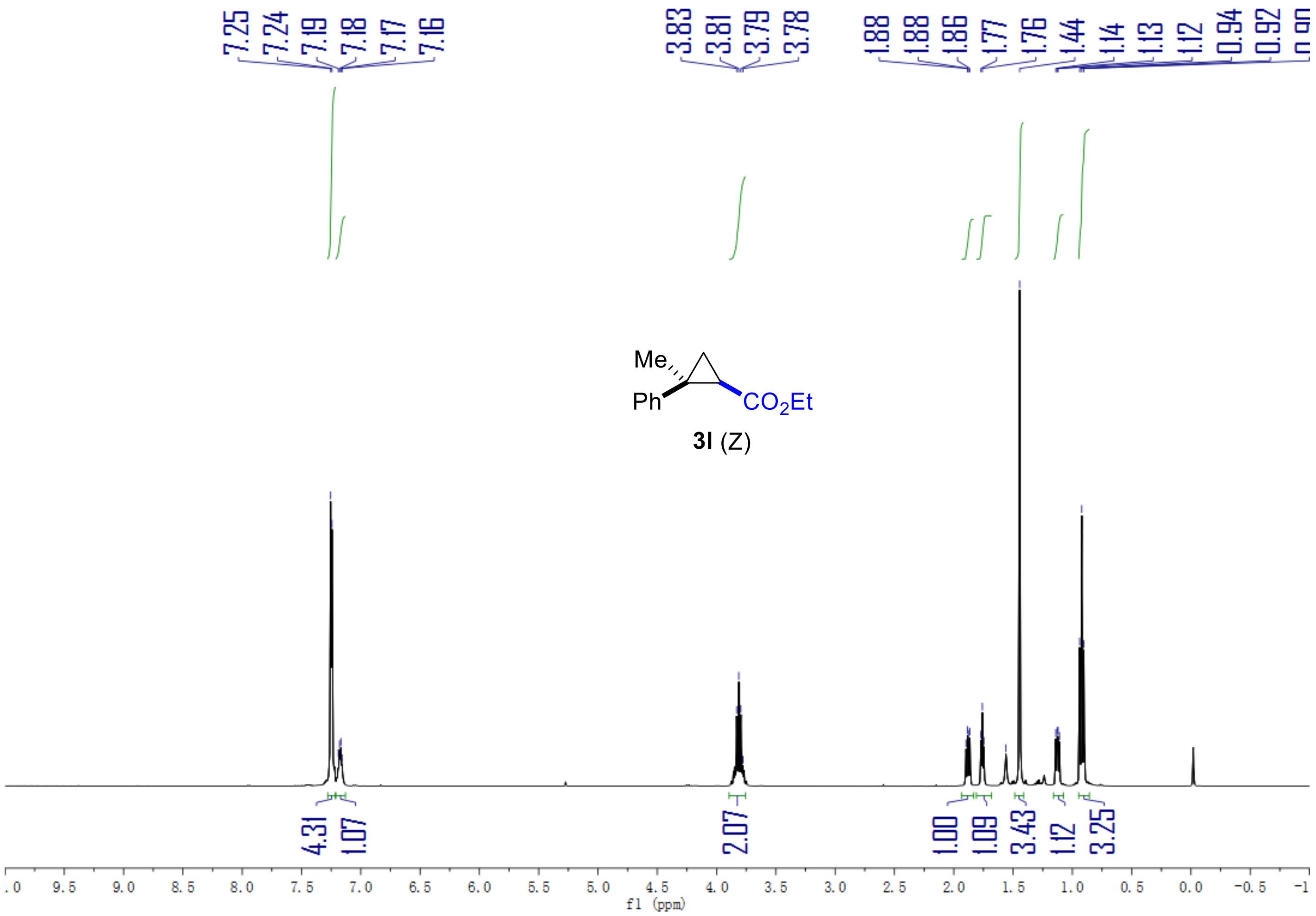

Supplementary Figure 47. ^{1}H NMR of **3l** (Z)

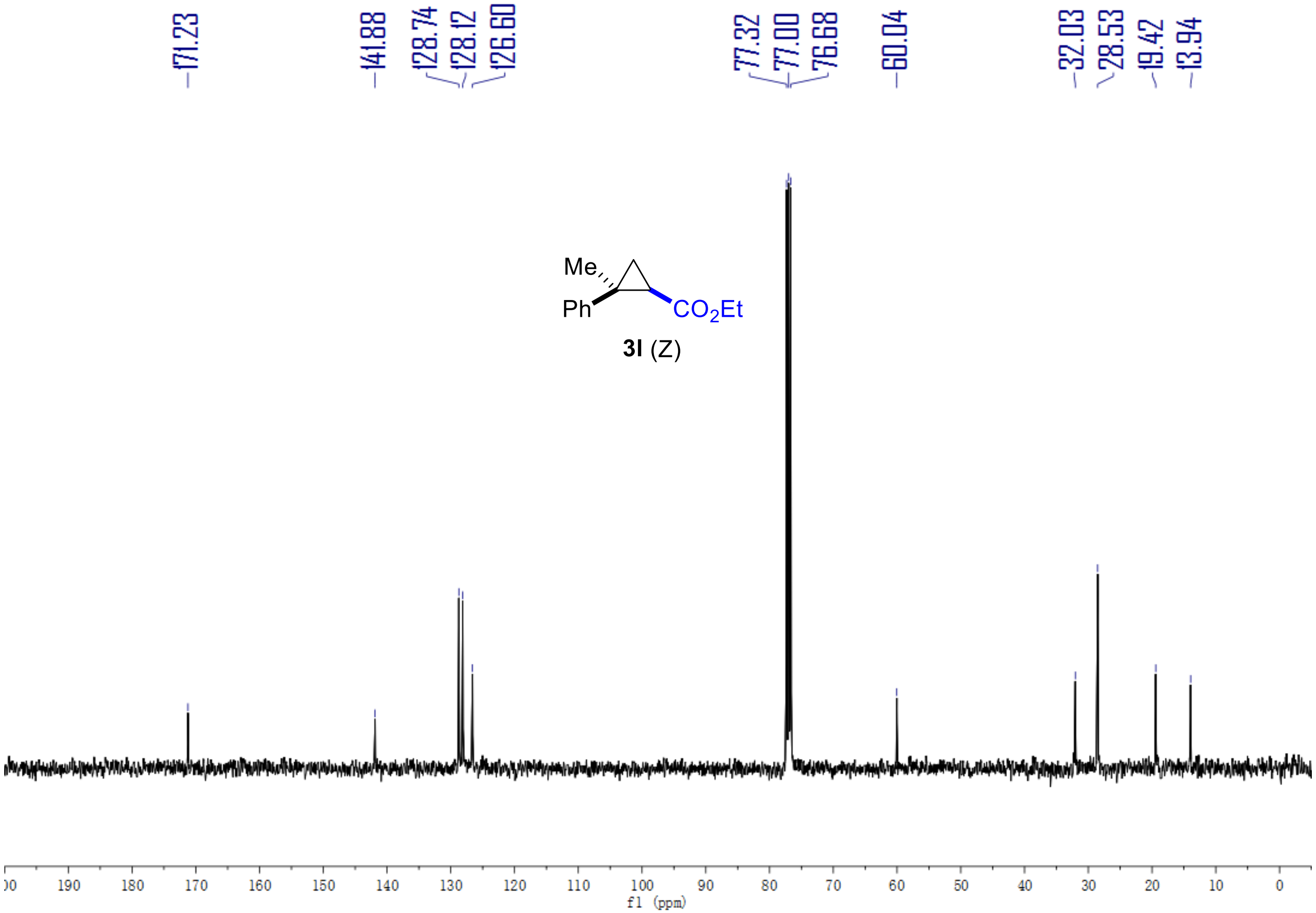

Supplementary Figure 48. ^{13}C NMR of **3l** (Z)

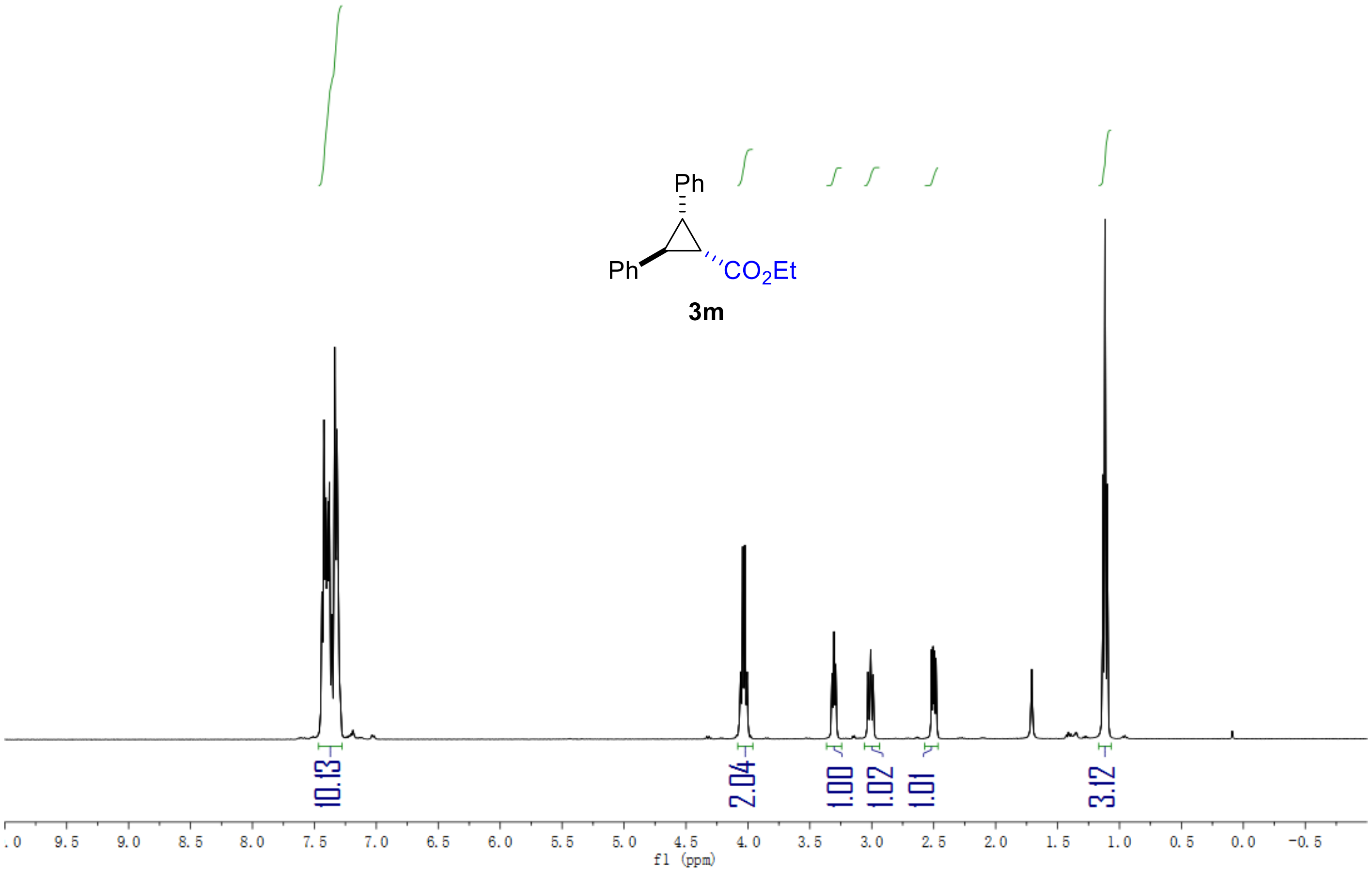

Supplementary Figure 49. ^{1}H NMR of **3m**

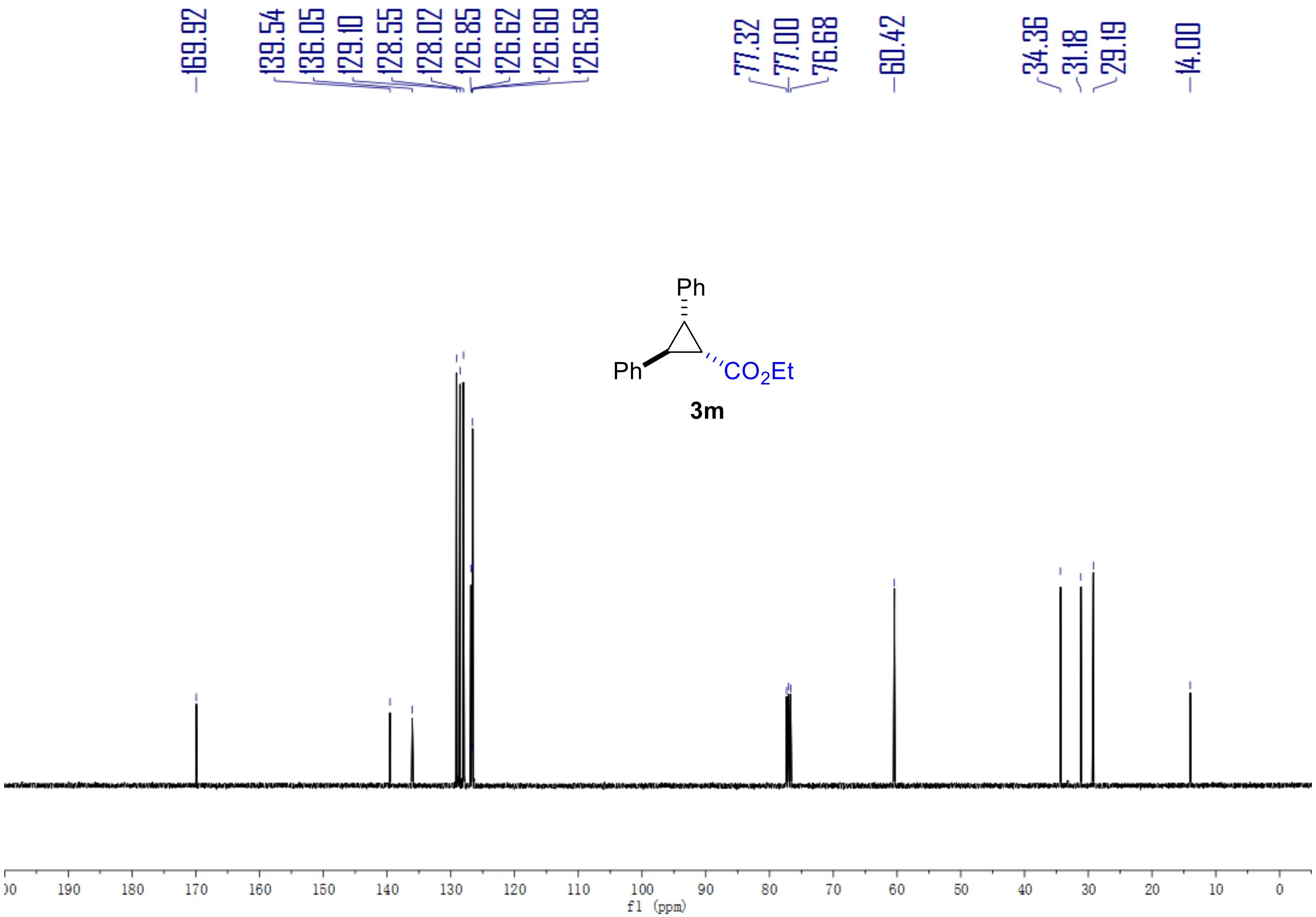

Supplementary Figure 50. ^{13}C NMR of **3m**

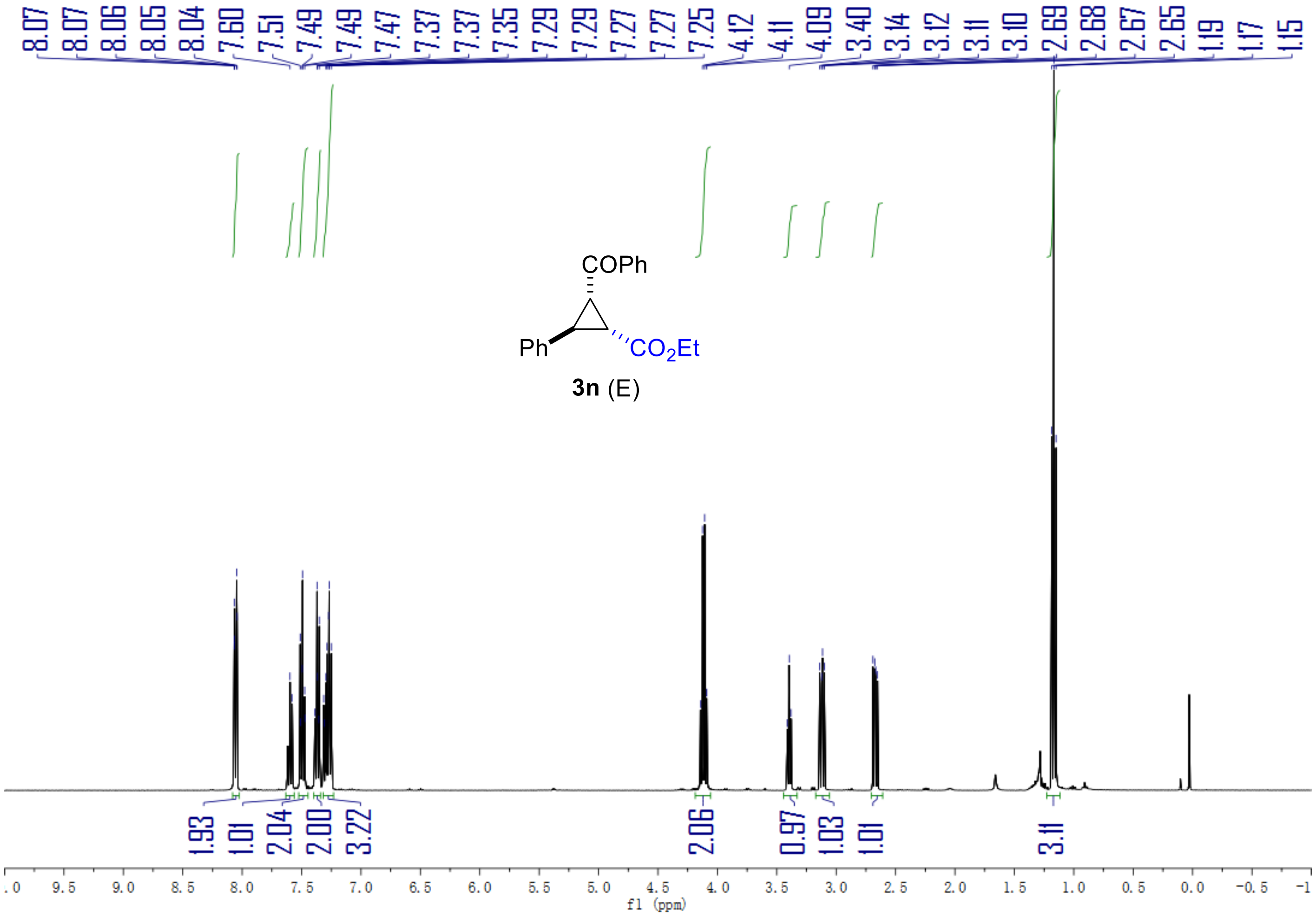

Supplementary Figure 51. ^{1}H NMR of **3n** (E)

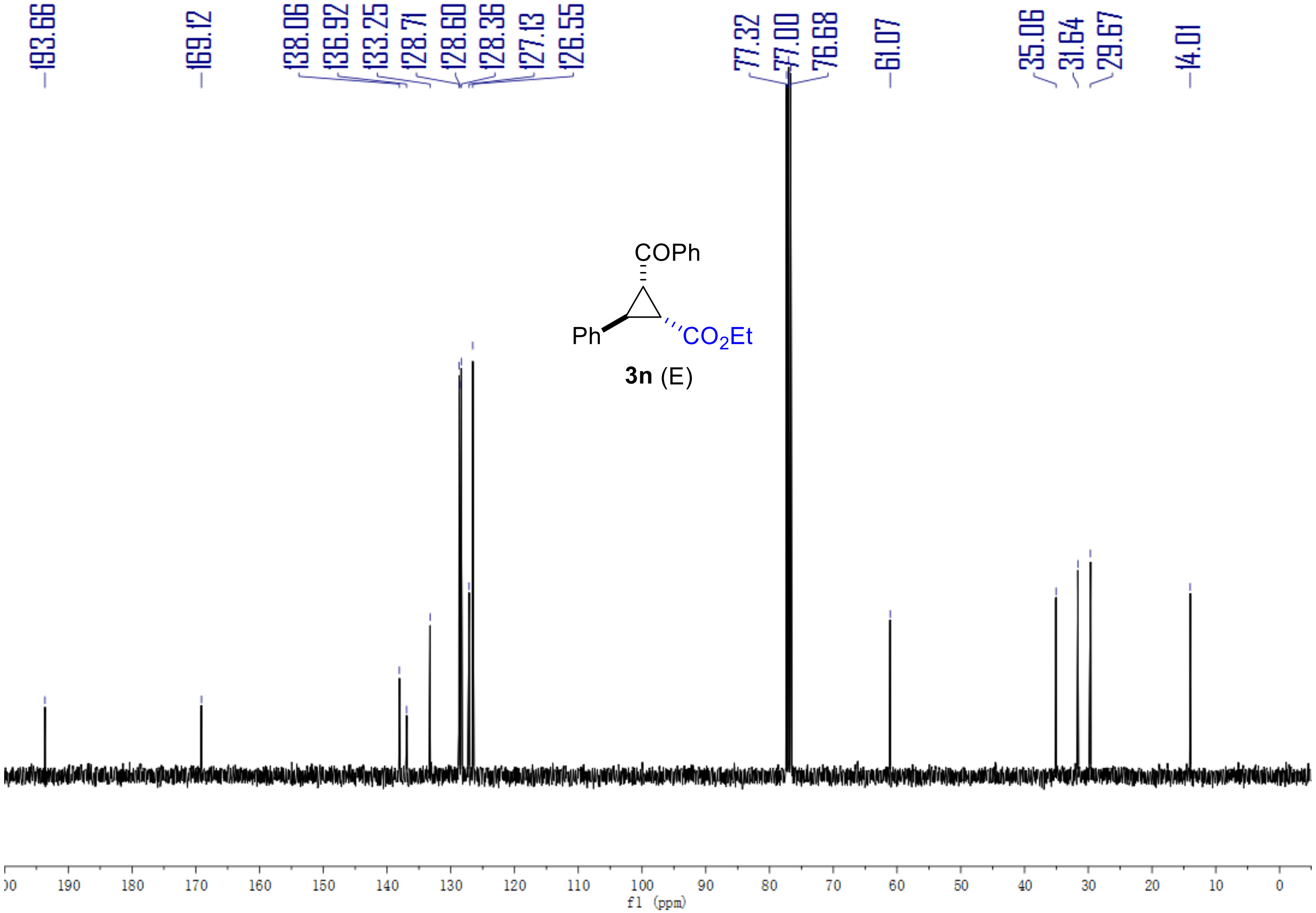

Supplementary Figure 52. ^{13}C NMR of **3n** (E)

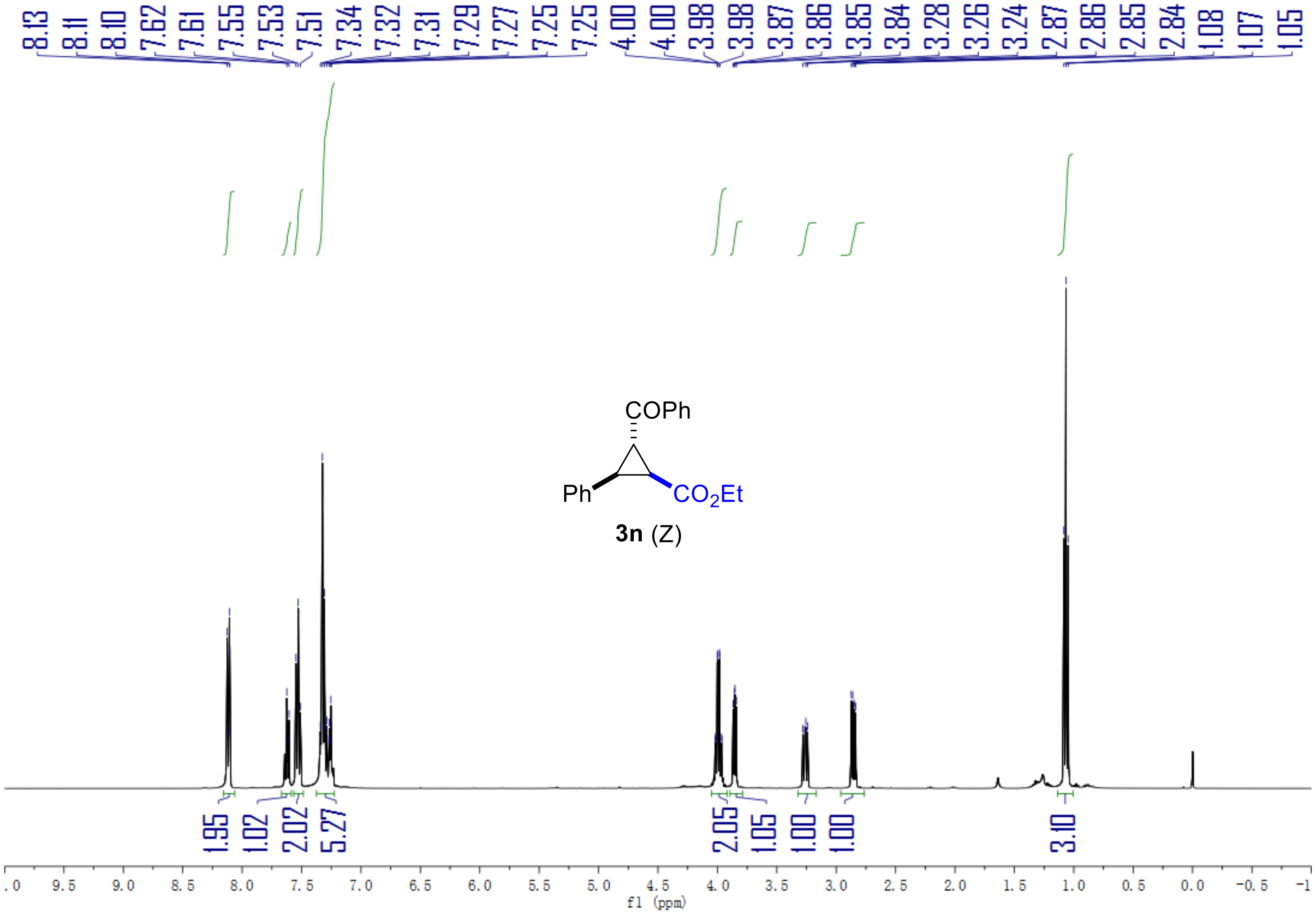

Supplementary Figure 53. ^{1}H NMR of **3n** (Z)

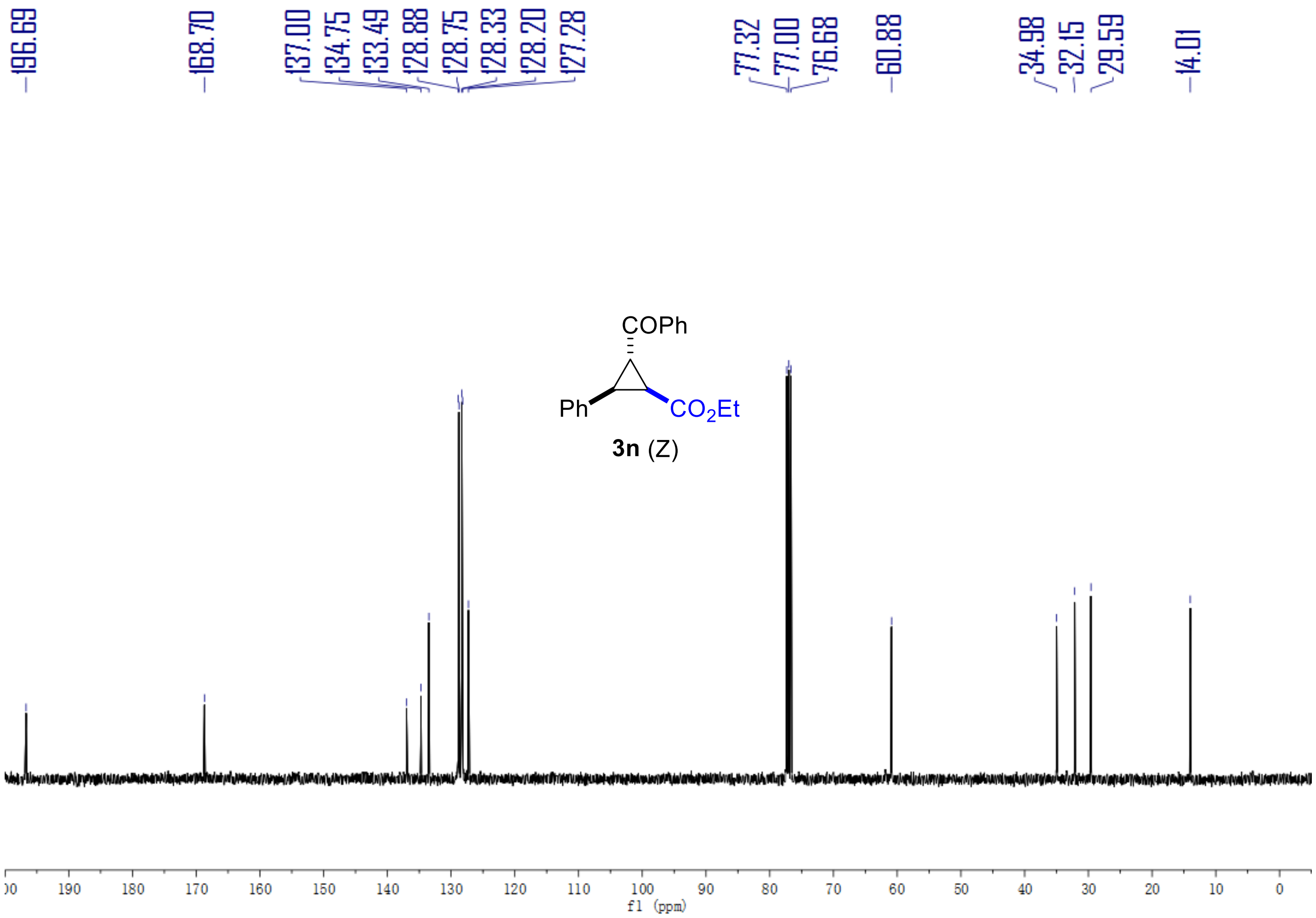

Supplementary Figure 54. ^{13}C NMR of **3n** (Z)

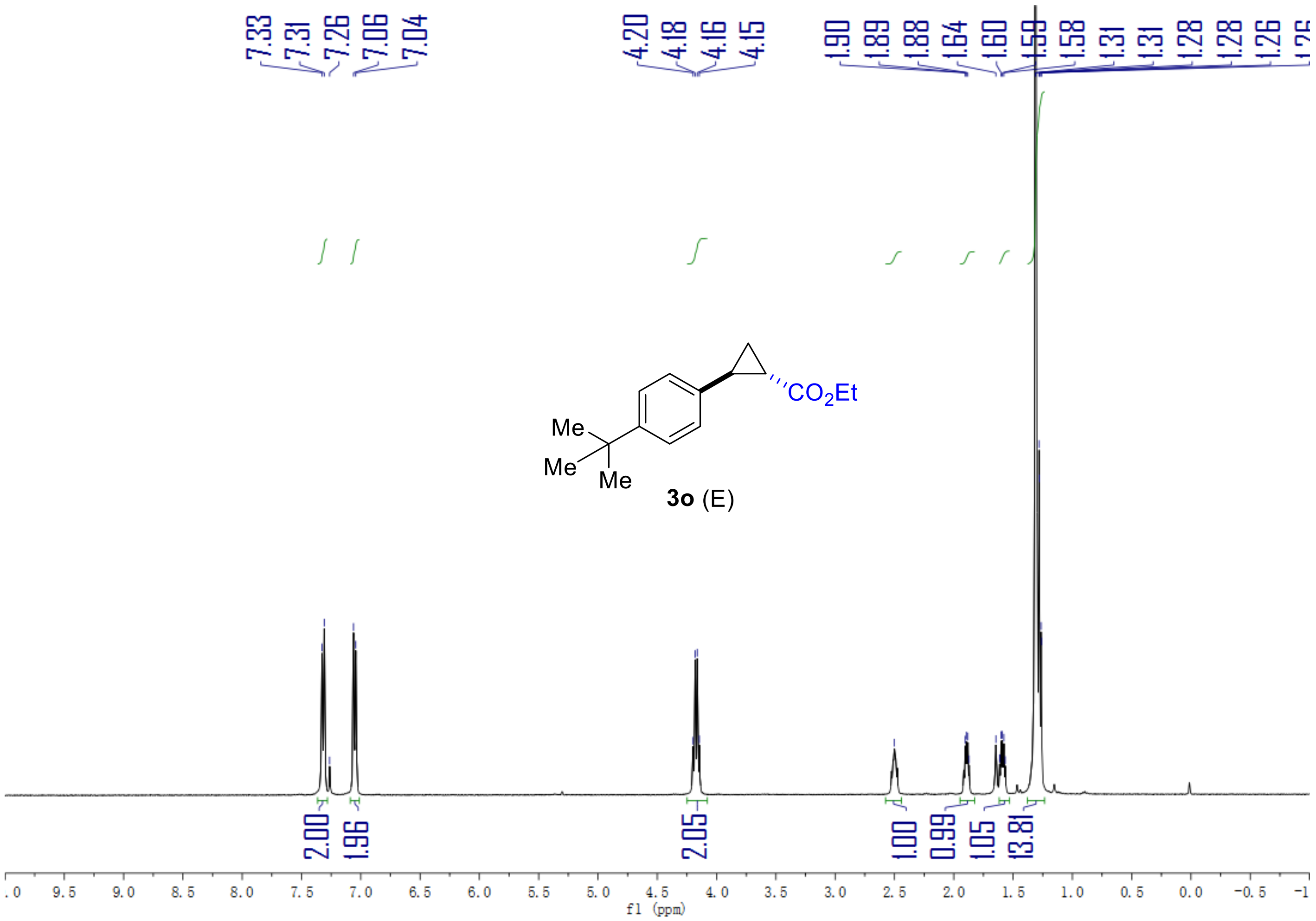

Supplementary Figure 55. ^{1}H NMR of **3o** (E)

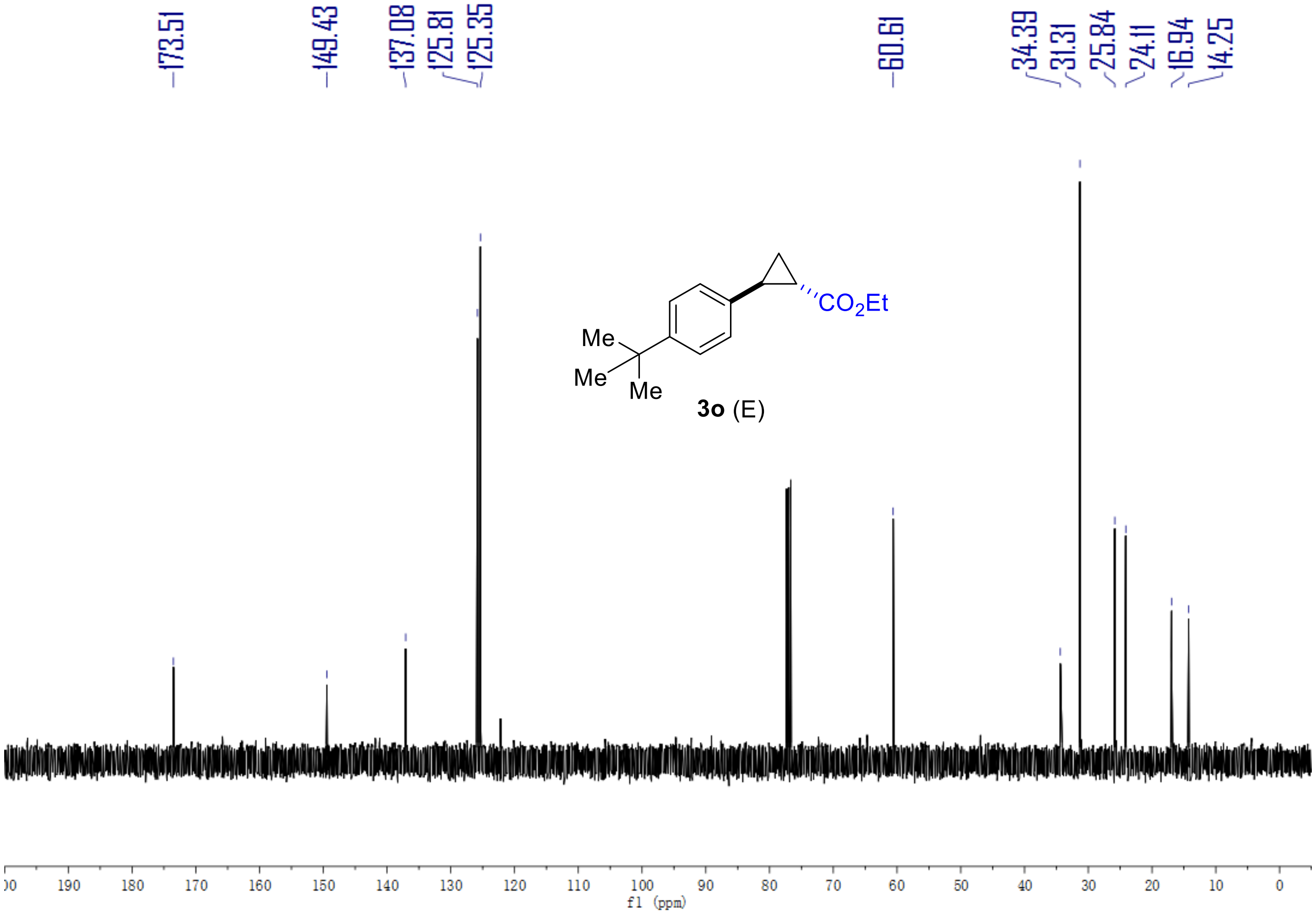

Supplementary Figure 56. ^{13}C NMR of **3o** (E)

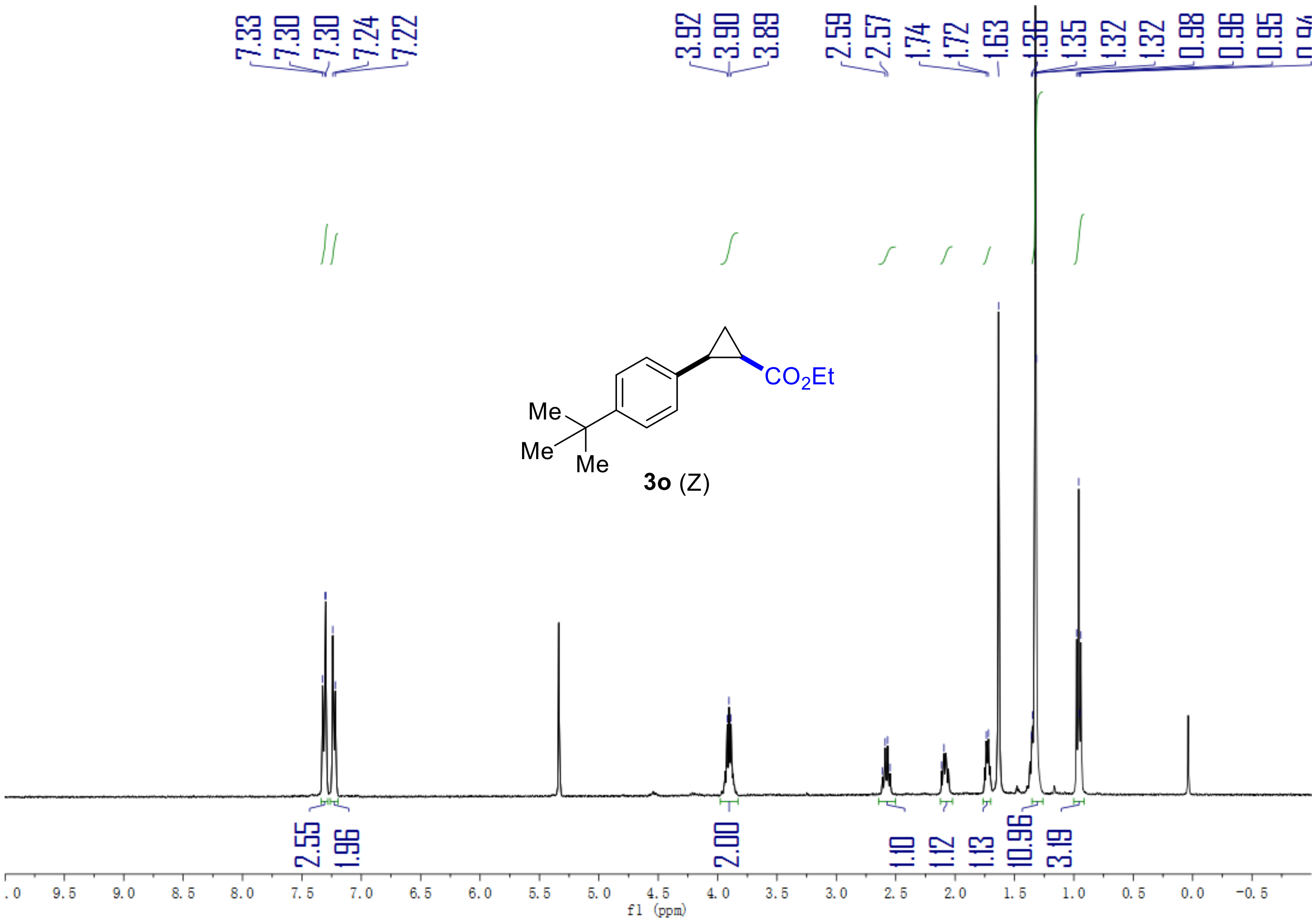

Supplementary Figure 57. ^{1}H NMR of **3o** (Z)

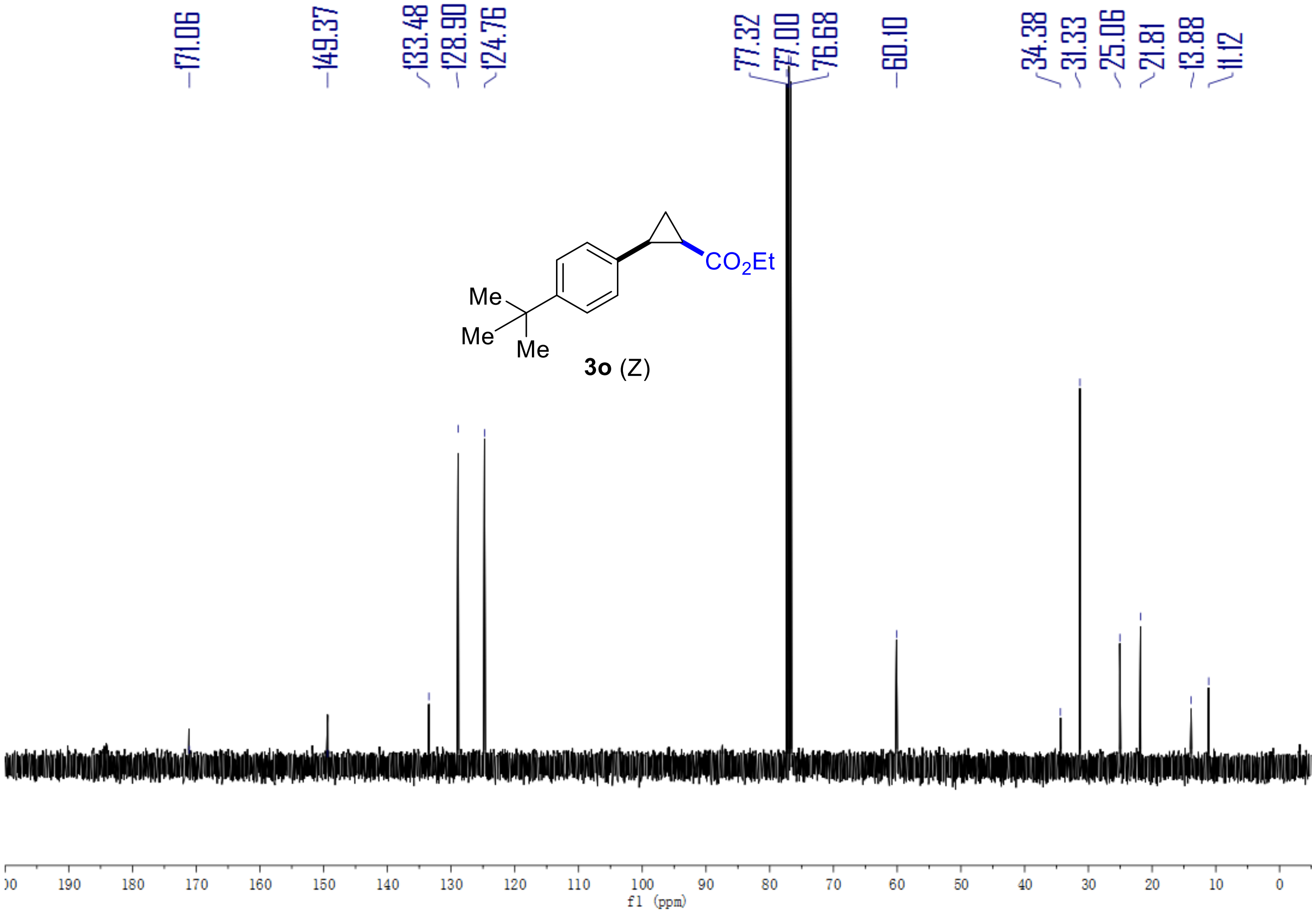

Supplementary Figure 58. ^{13}C NMR of **3o** (Z)

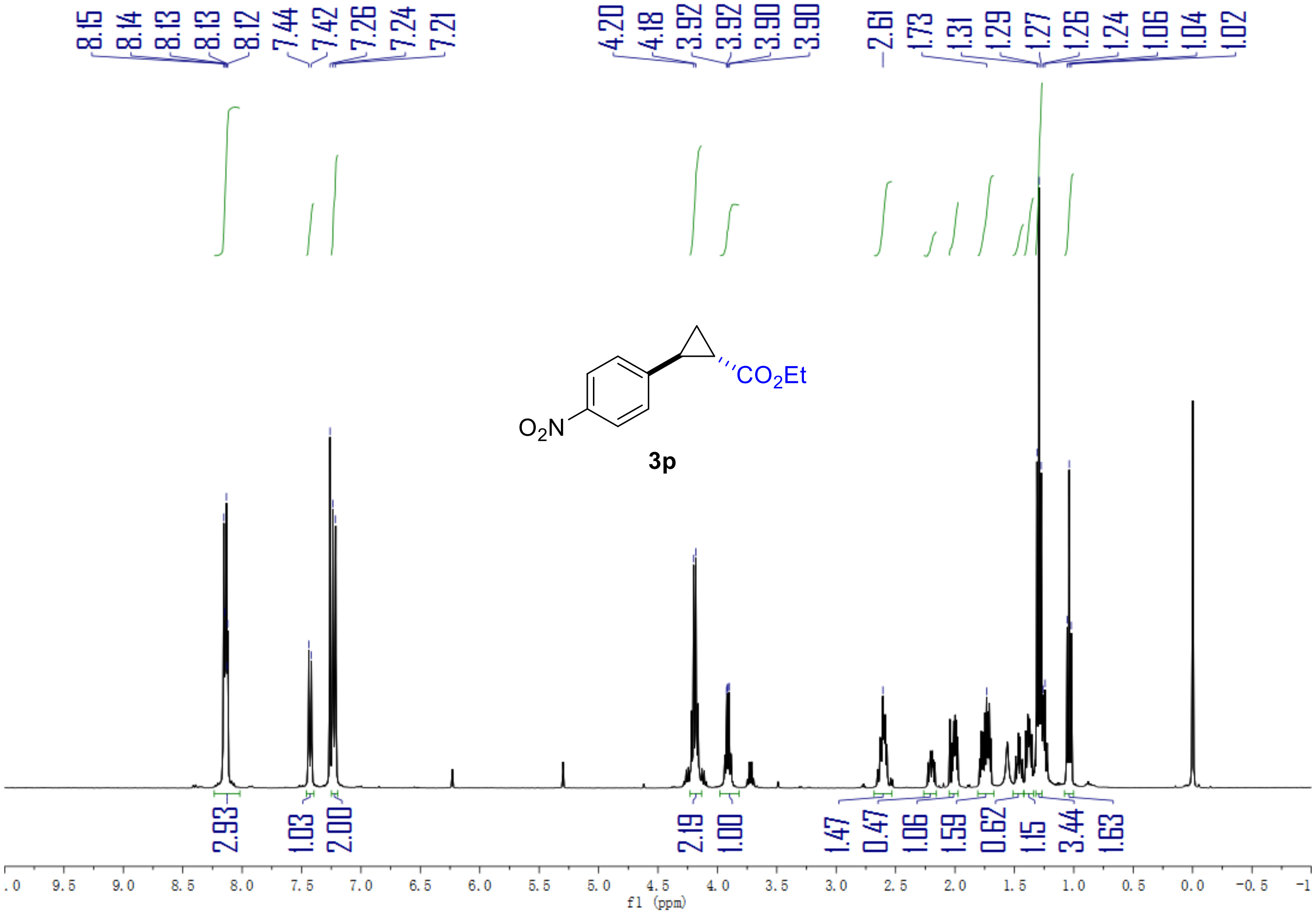

Supplementary Figure 59. ^{1}H NMR of **3p**

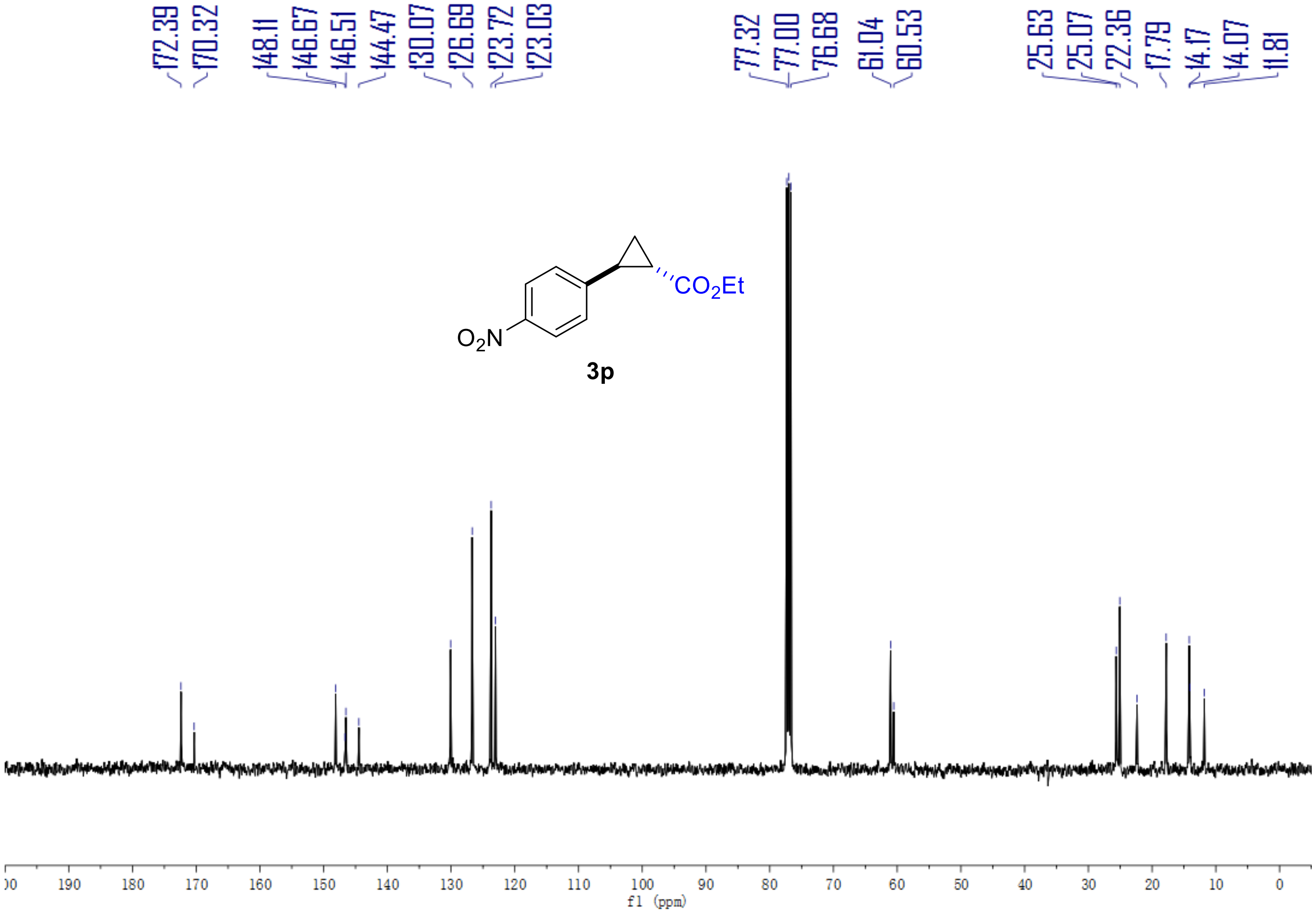

Supplementary Figure 60. ^{13}C NMR of **3p**

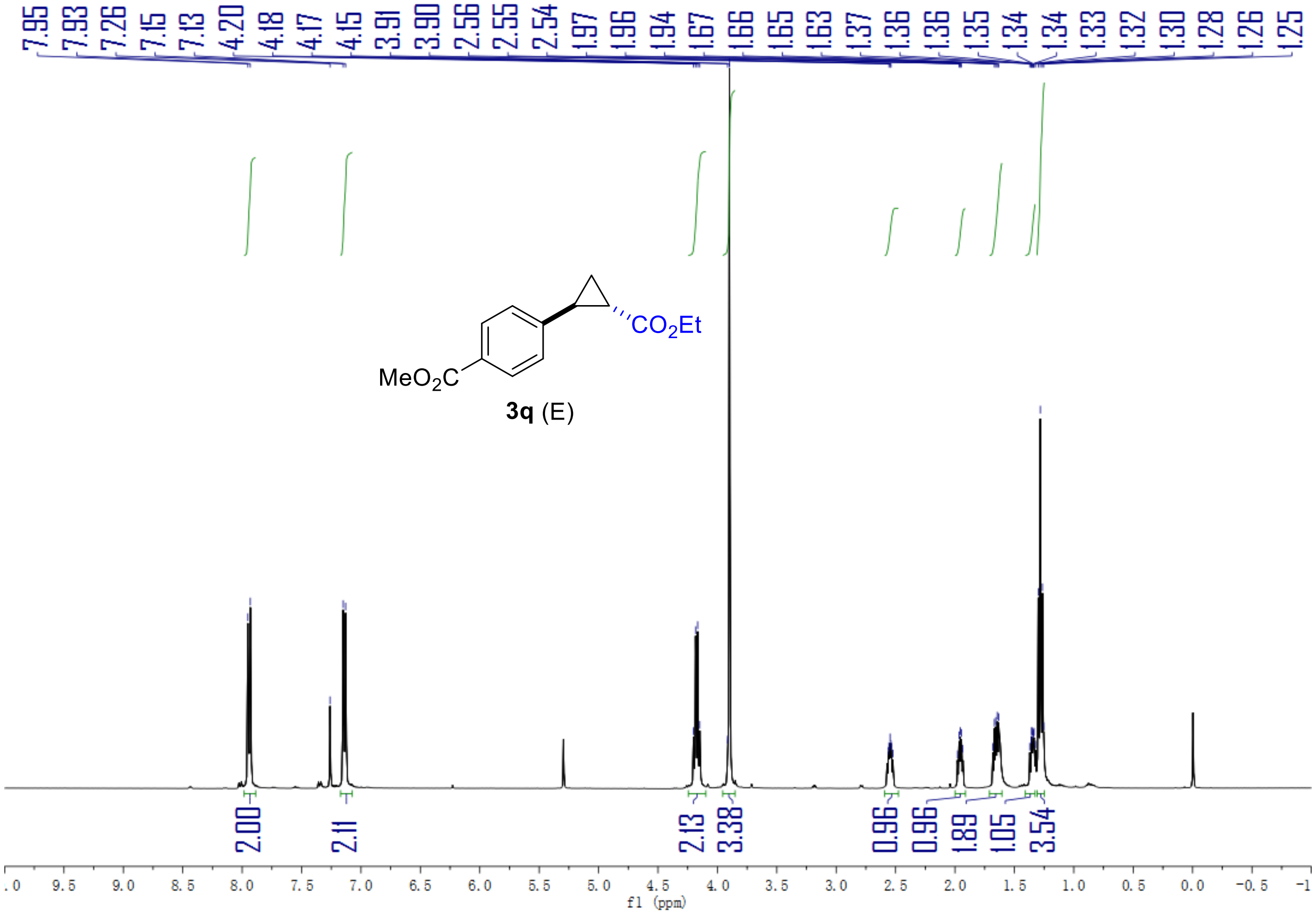

Supplementary Figure 61. ^{1}H NMR of **3q** (E)

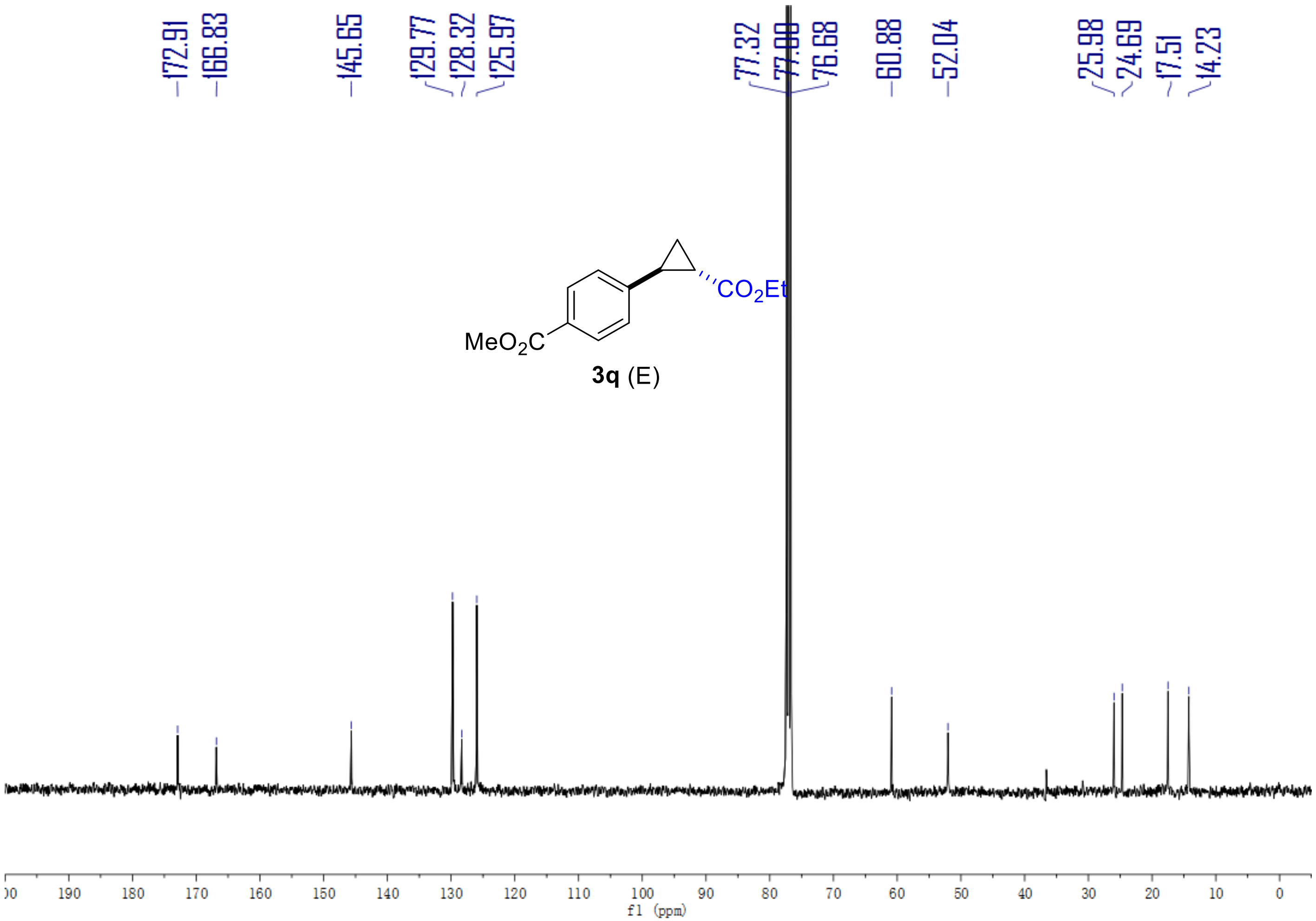

Supplementary Figure 62. ^{13}C NMR of **3q** (E)

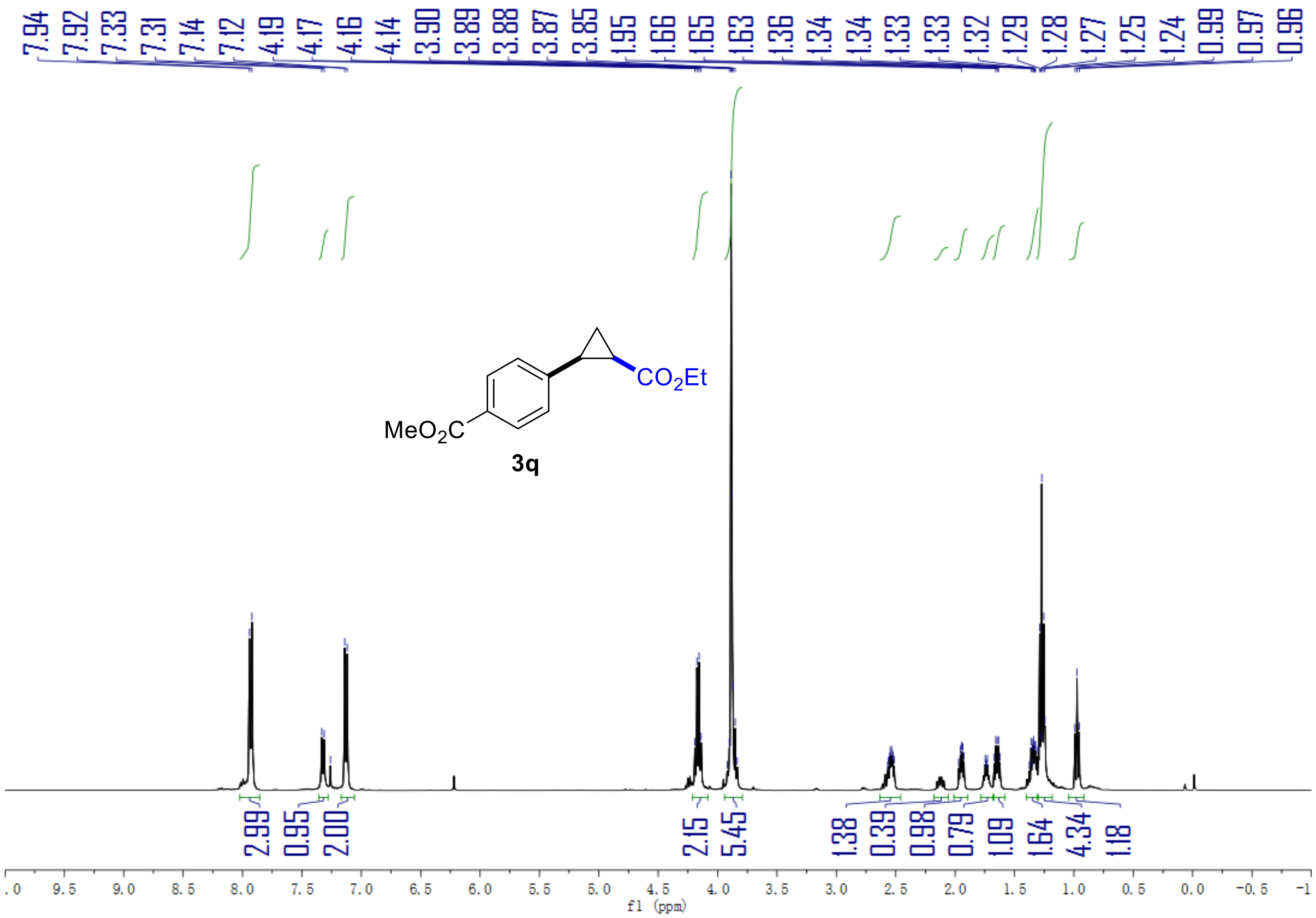

Supplementary Figure 63. ^{1}H NMR of **3q**

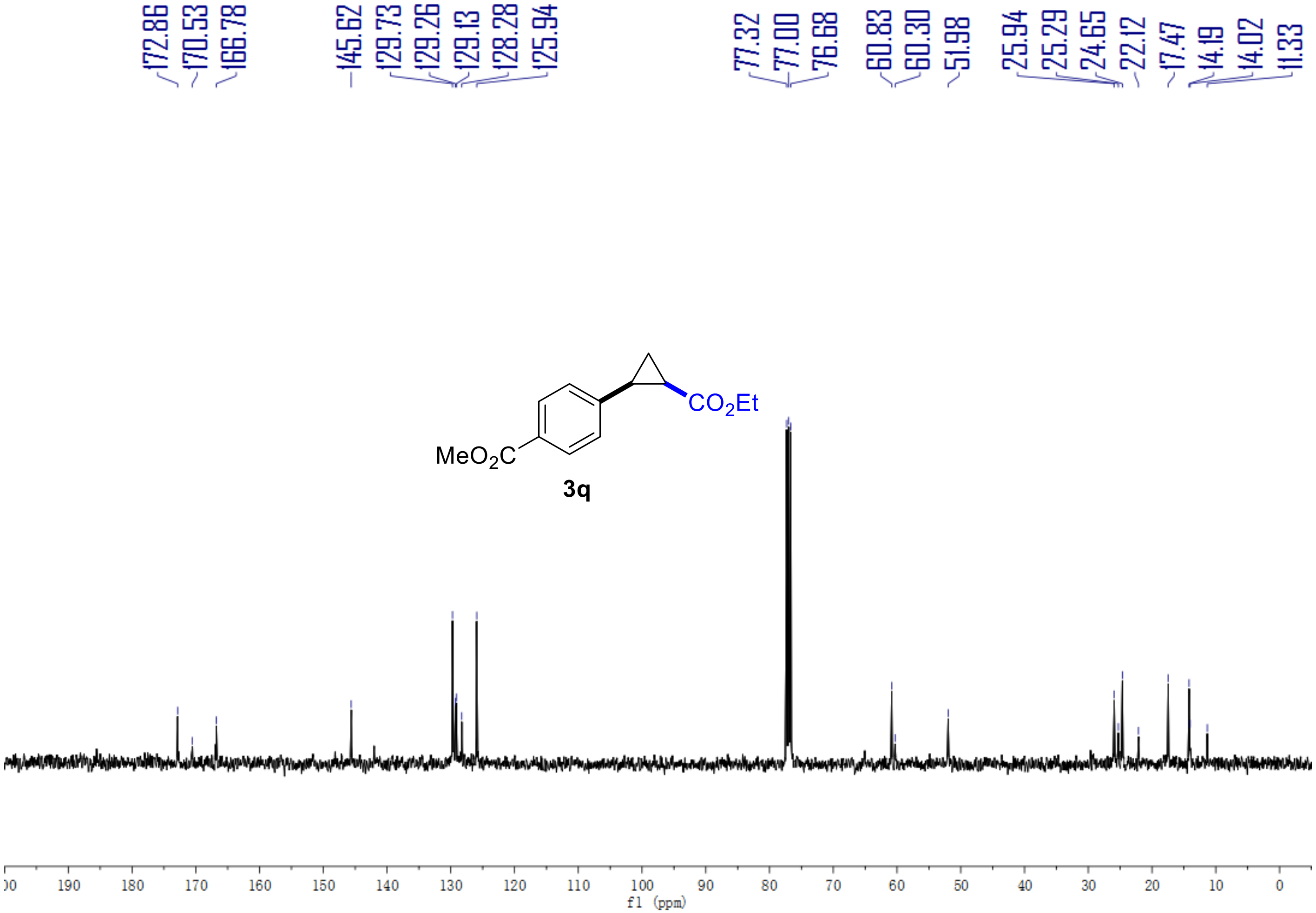

Supplementary Figure 64. ^{13}C NMR of **3q**

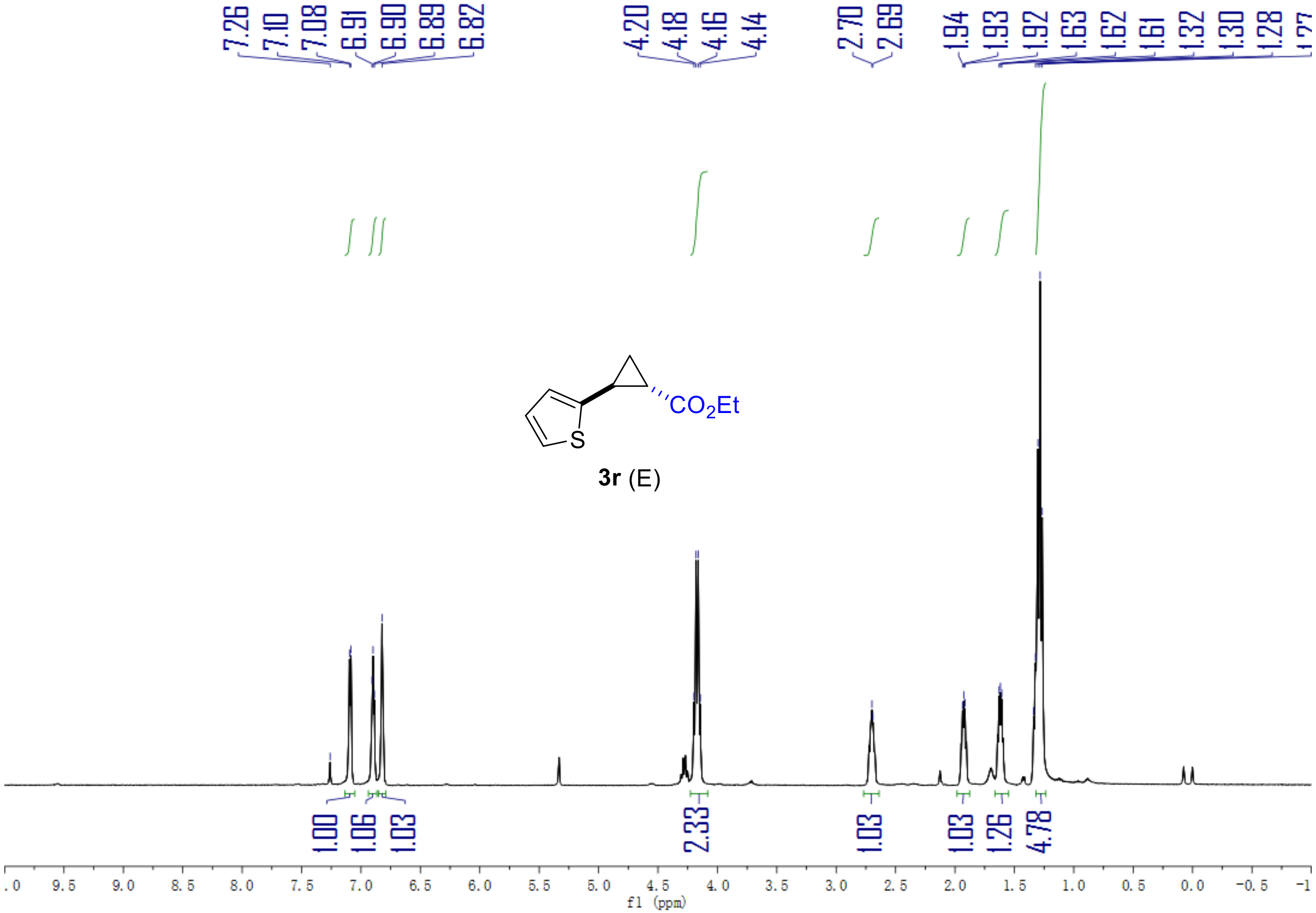

Supplementary Figure 65. ^{1}H NMR of **3r** (E)

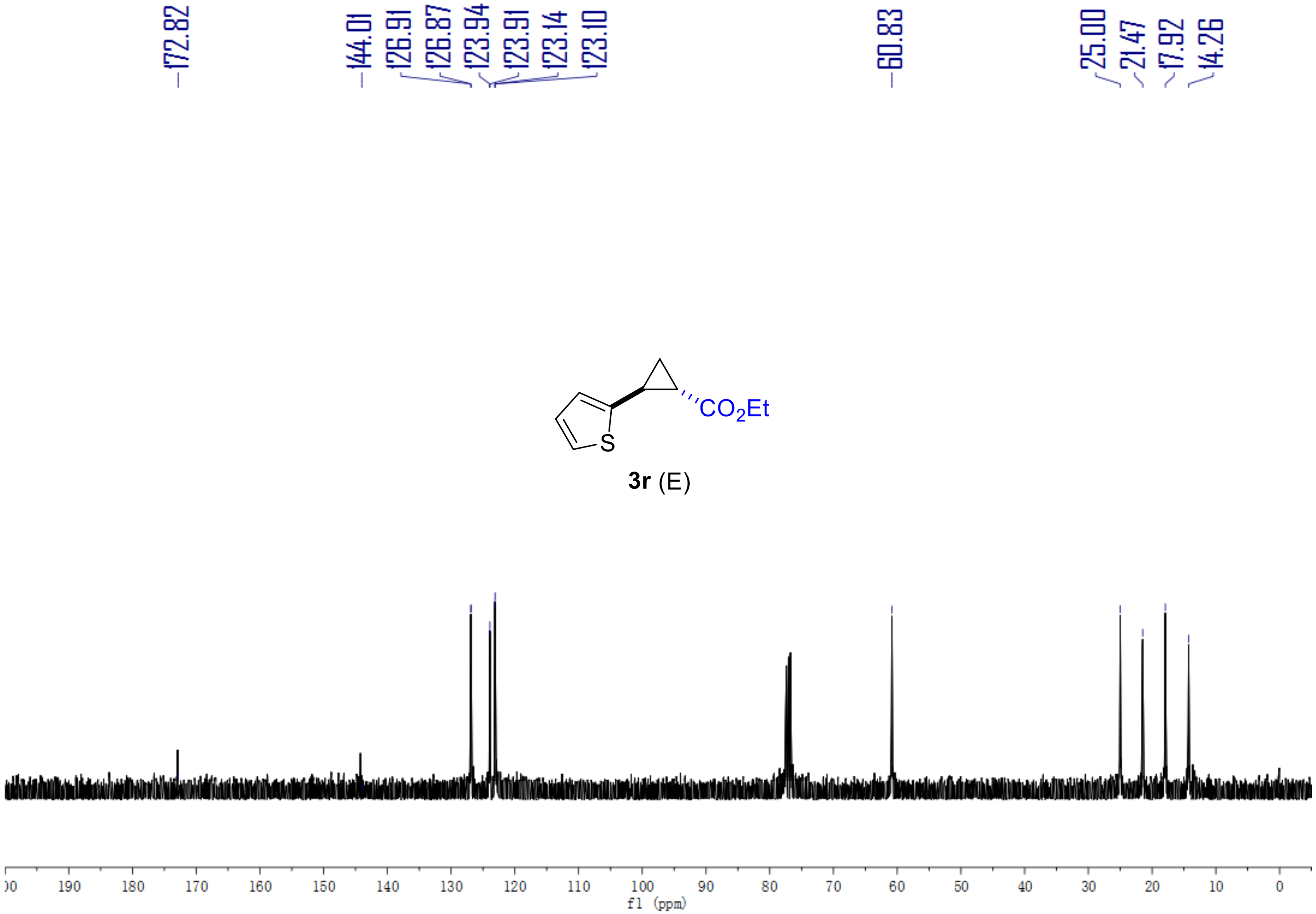

3r (E)

Supplementary Figure 66. ^{13}C NMR of **3r** (E)

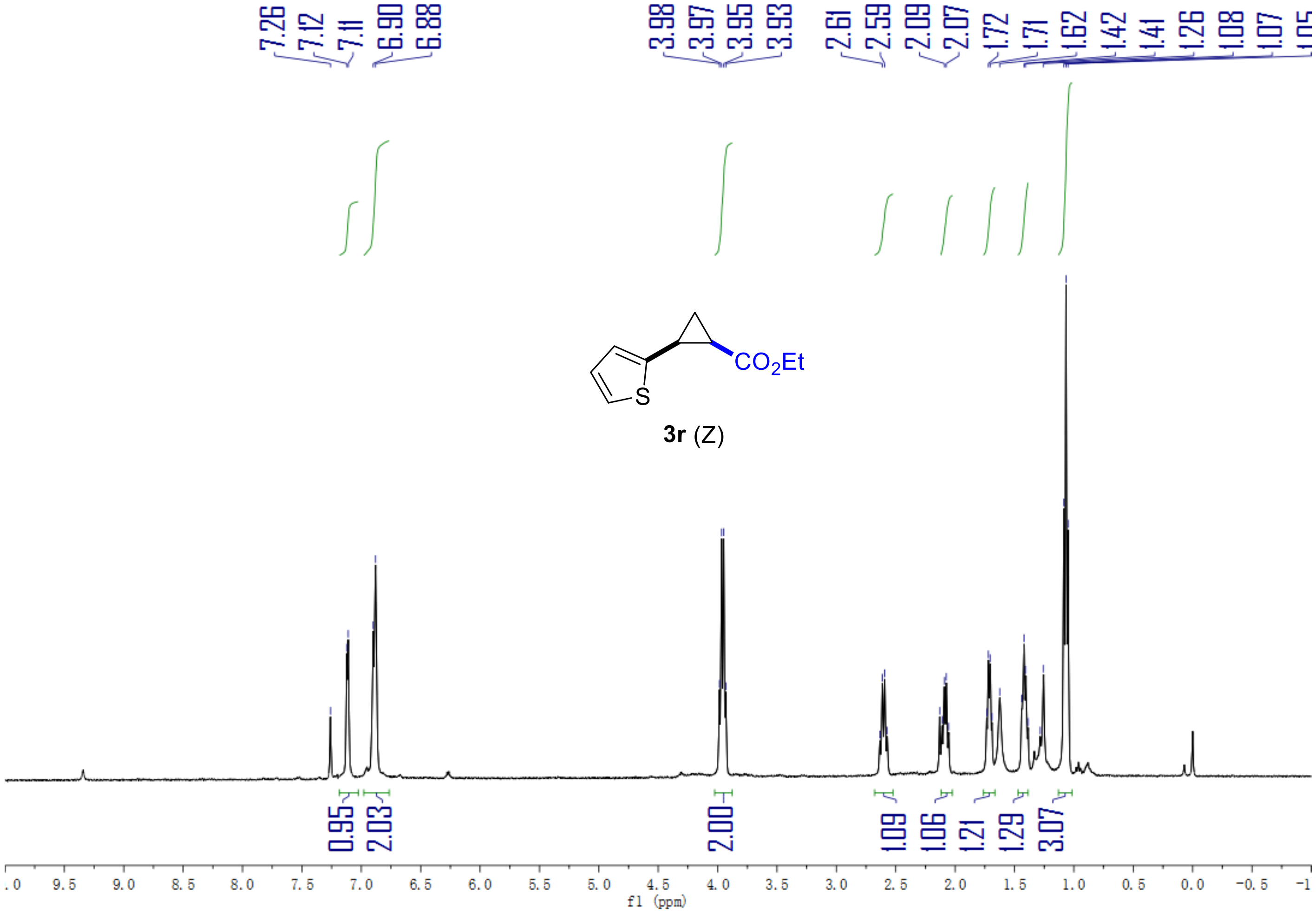

Supplementary Figure 67. ^{1}H NMR of **3r** (Z)

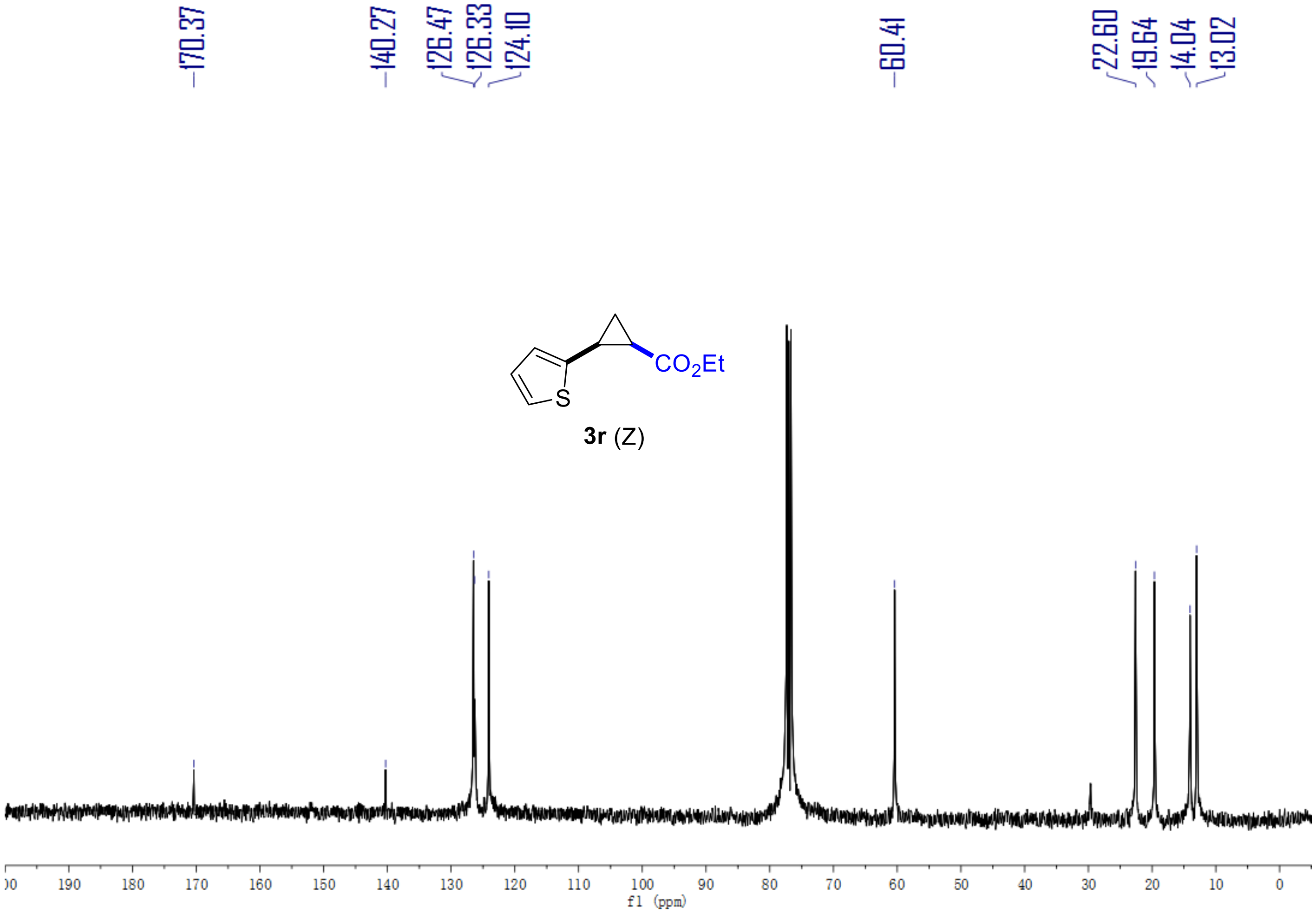

Supplementary Figure 68. ^{13}C NMR of **3r** (Z)

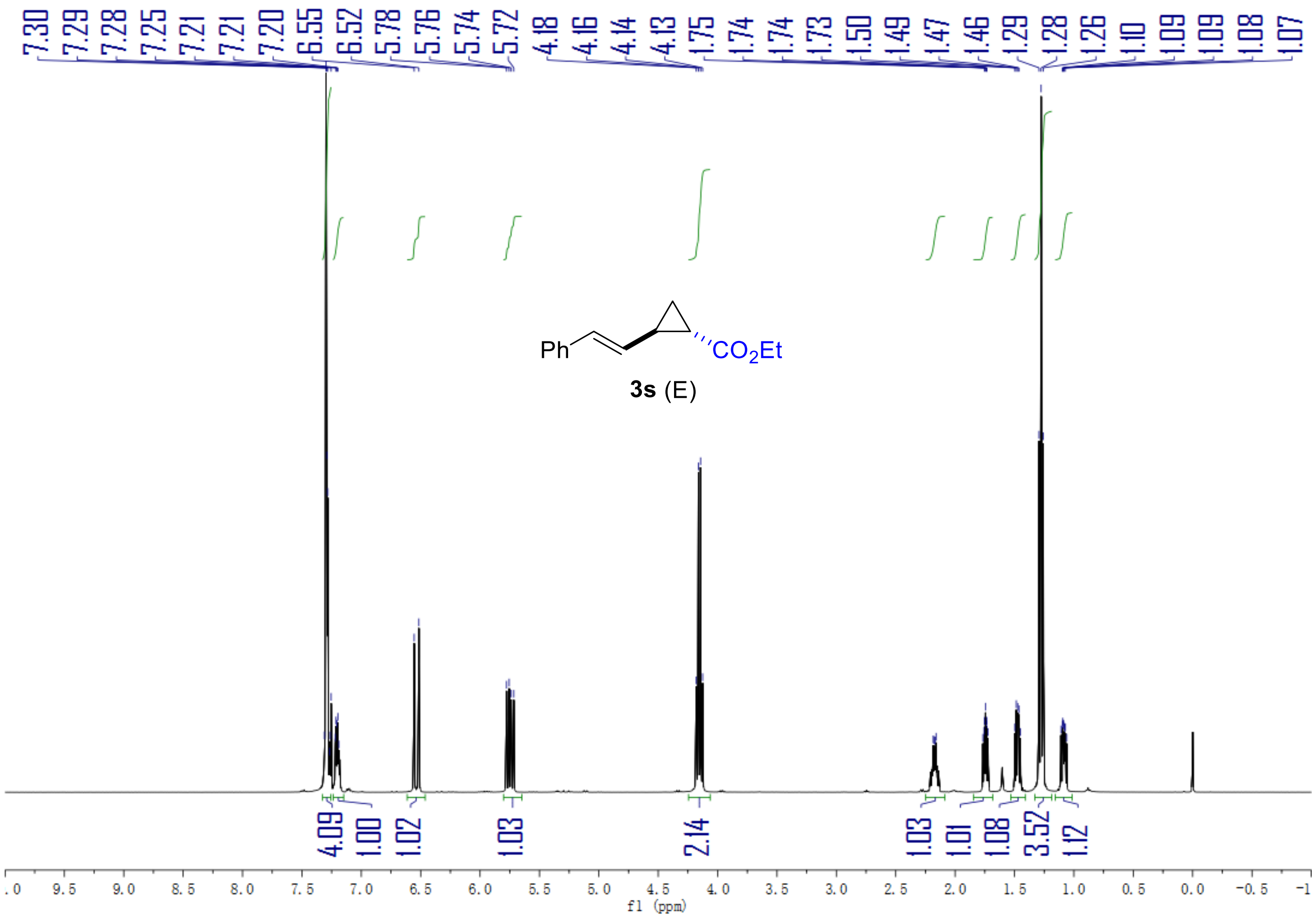

Supplementary Figure 69. ^{1}H NMR of **3s** (E)

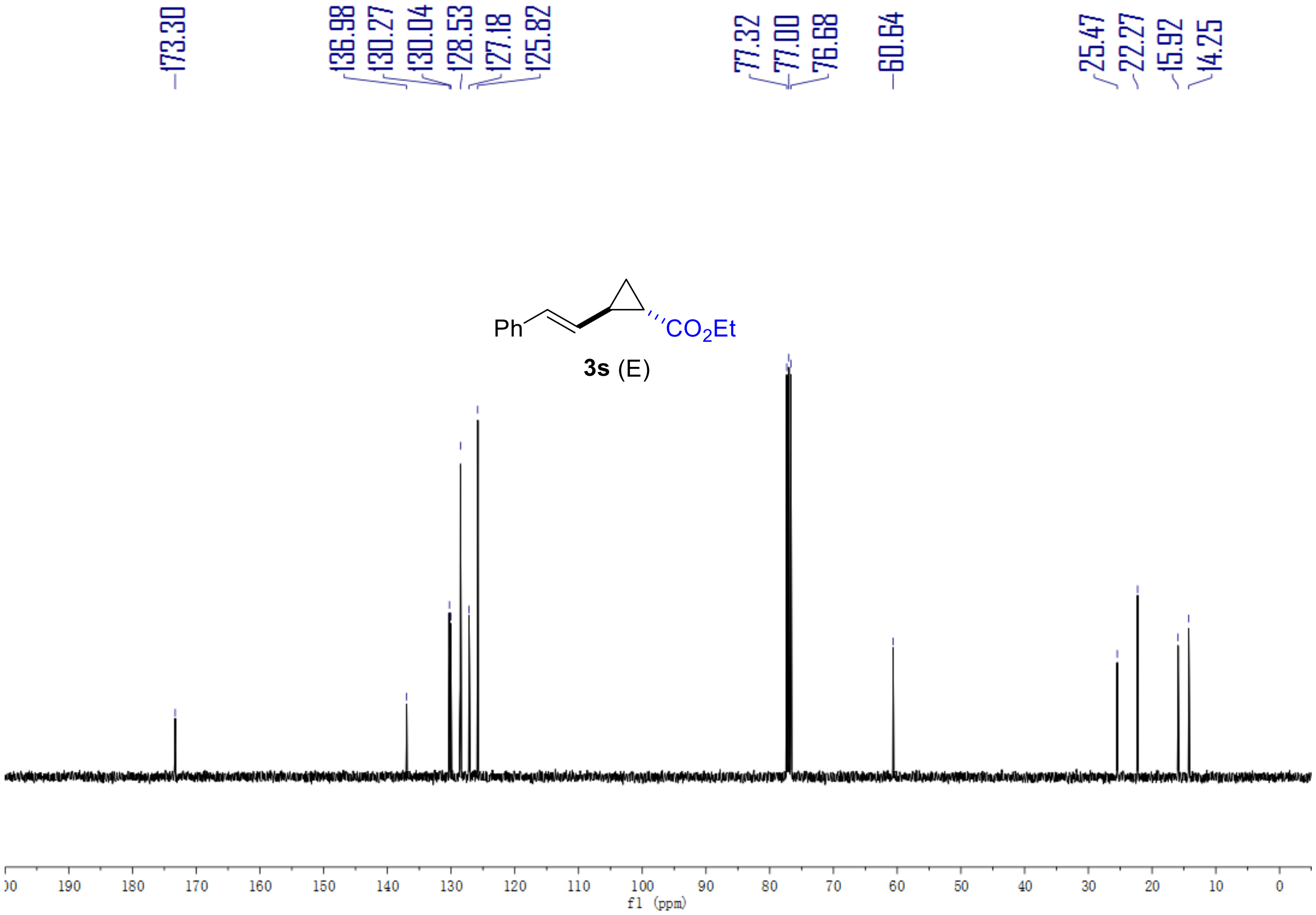

Supplementary Figure 70. ^{13}C NMR of **3s** (E)

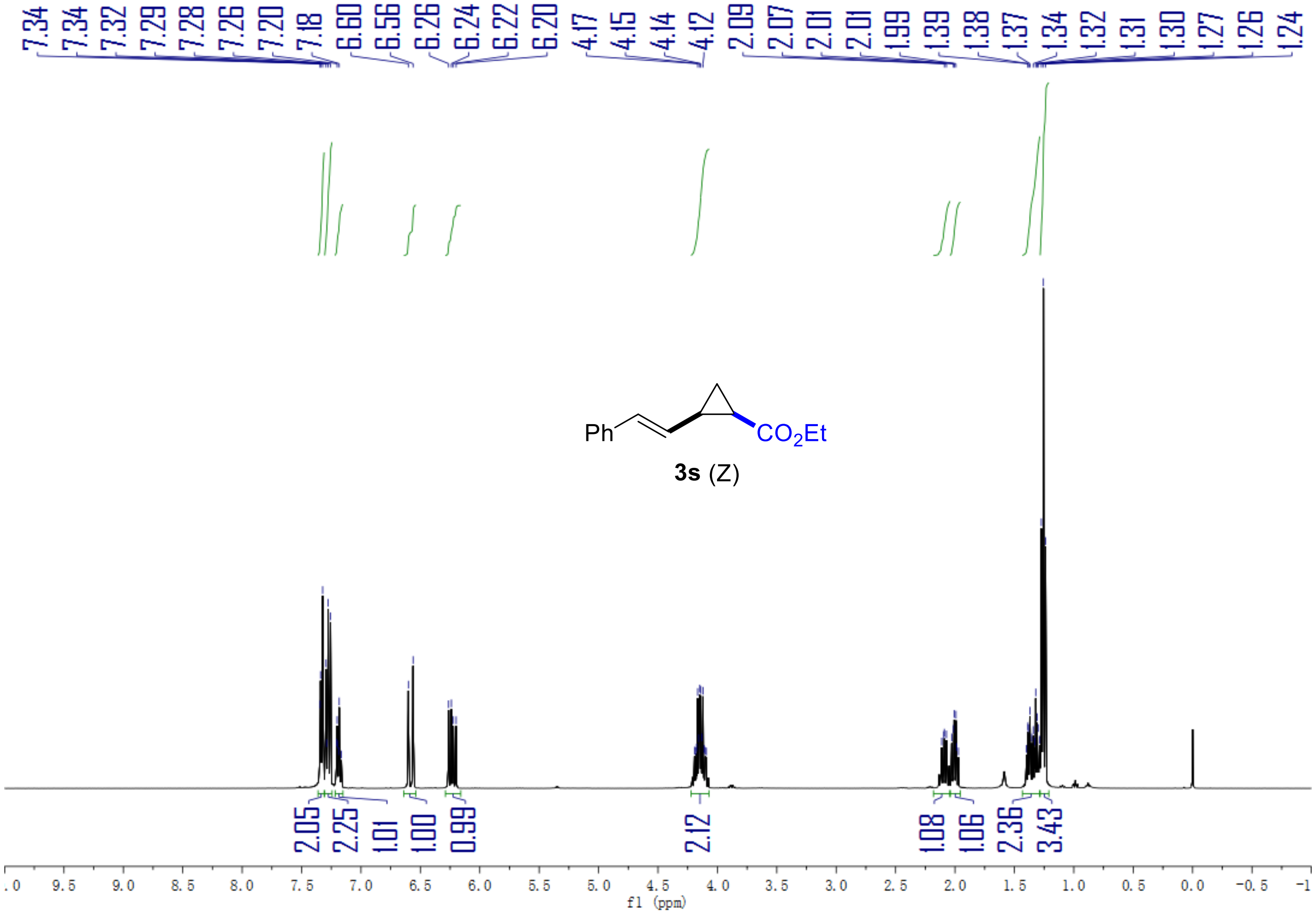

Supplementary Figure 71. ^1H NMR of **3s** (Z)

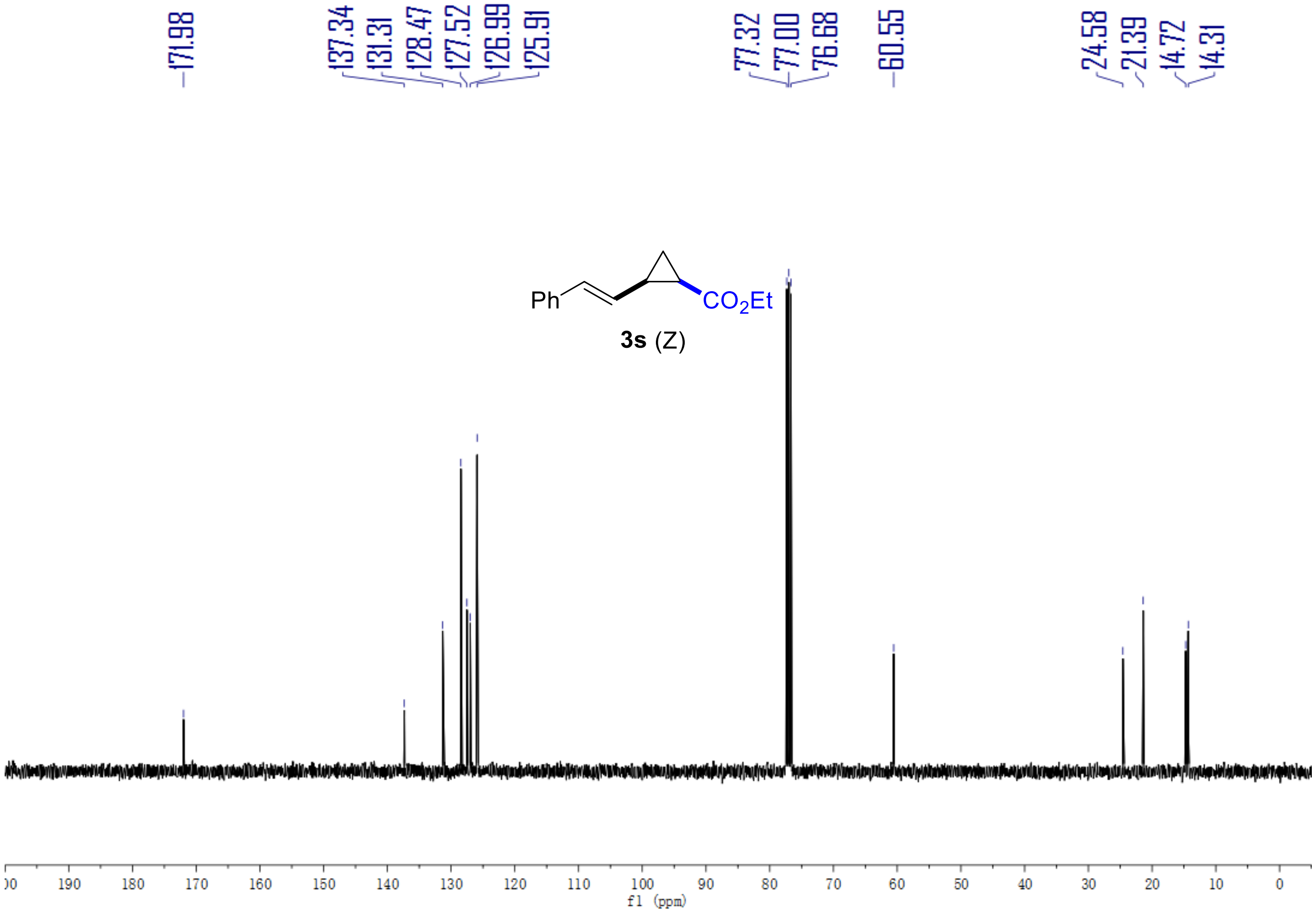

Supplementary Figure 72. ^{13}C NMR of **3s** (Z)

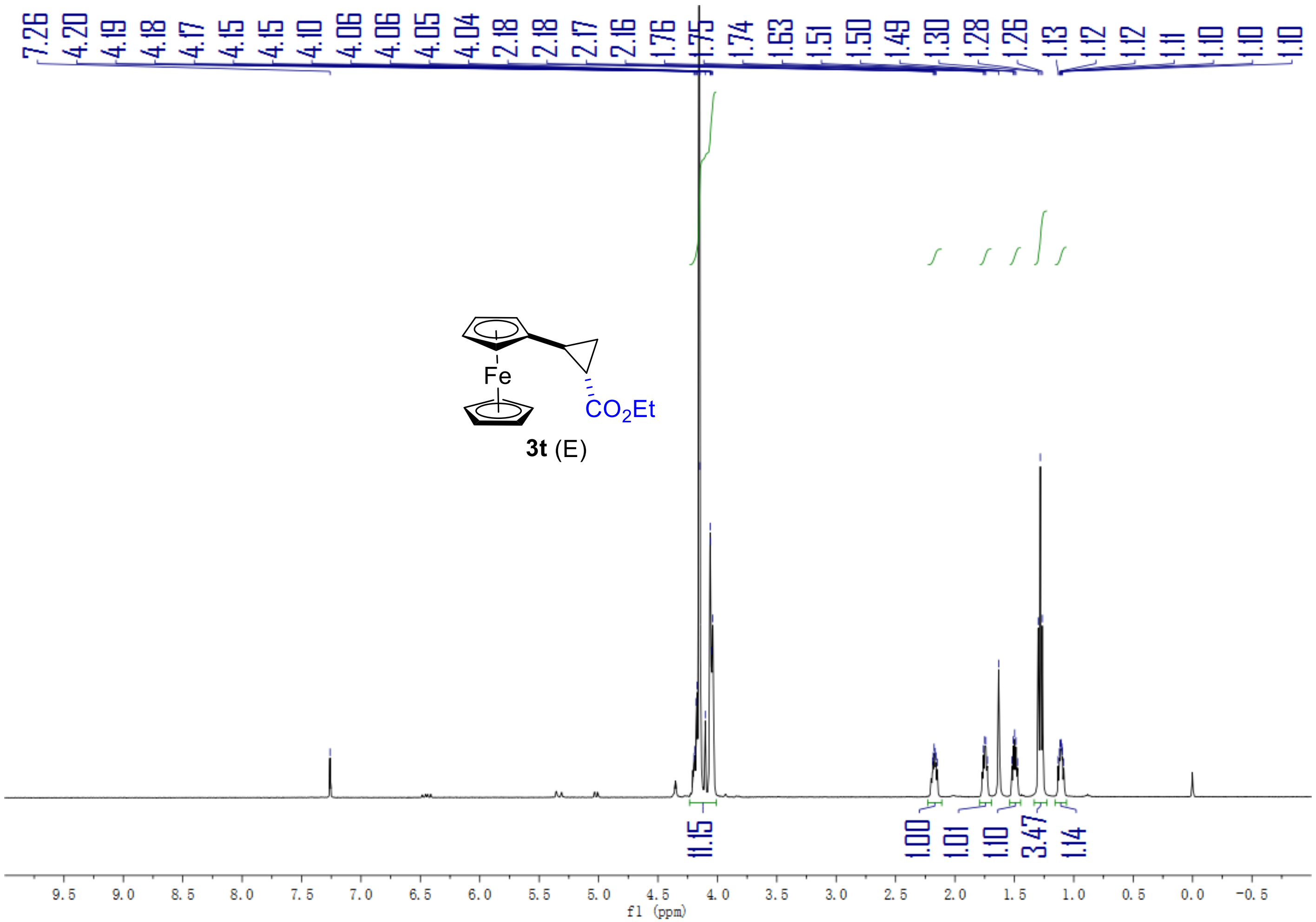

Supplementary Figure 73. ^{1}H NMR of **3t** (E)

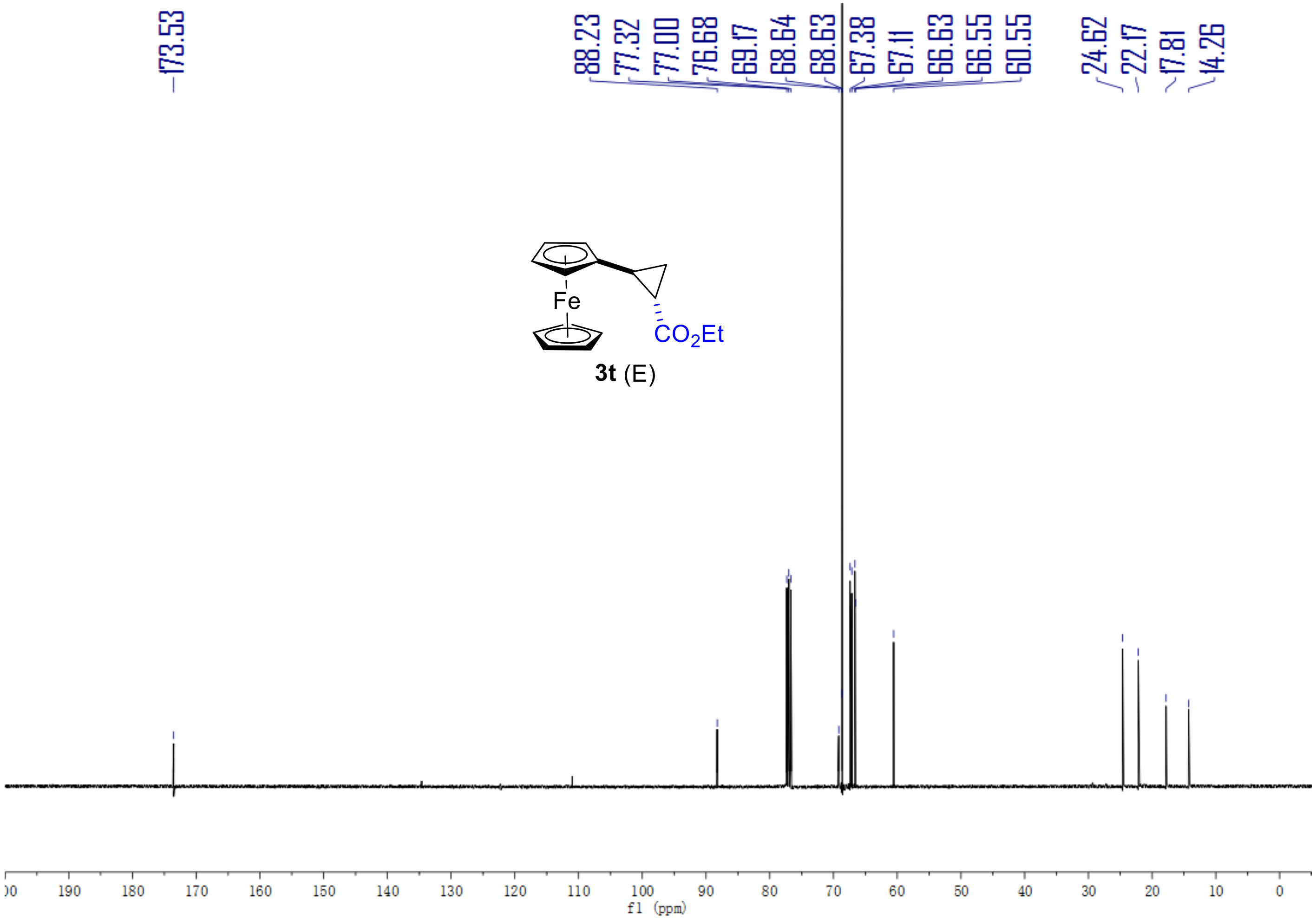

Supplementary Figure 74. ^{13}C NMR of **3t** (E)

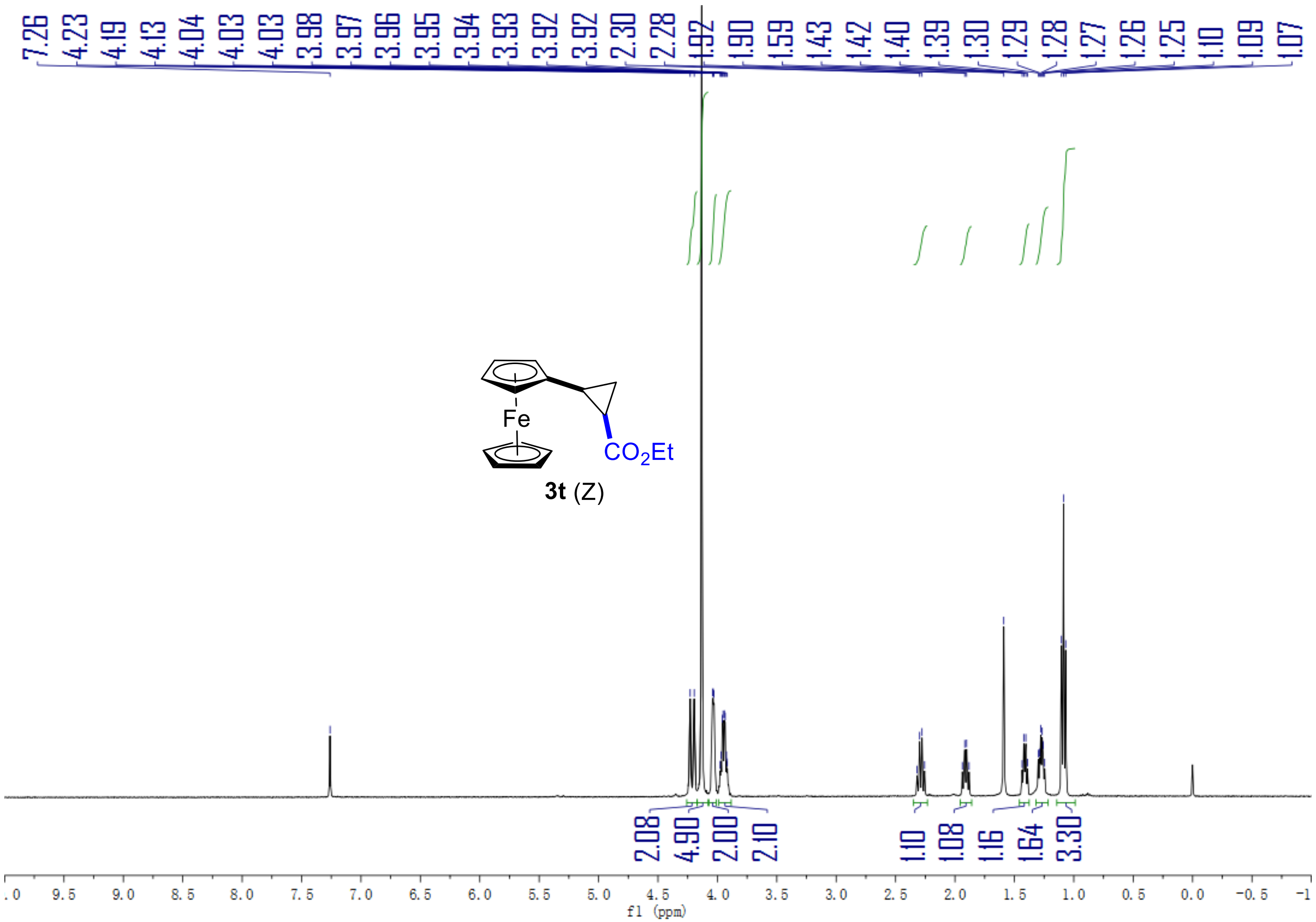

Supplementary Figure 75. ^{1}H NMR of **3t** (Z)

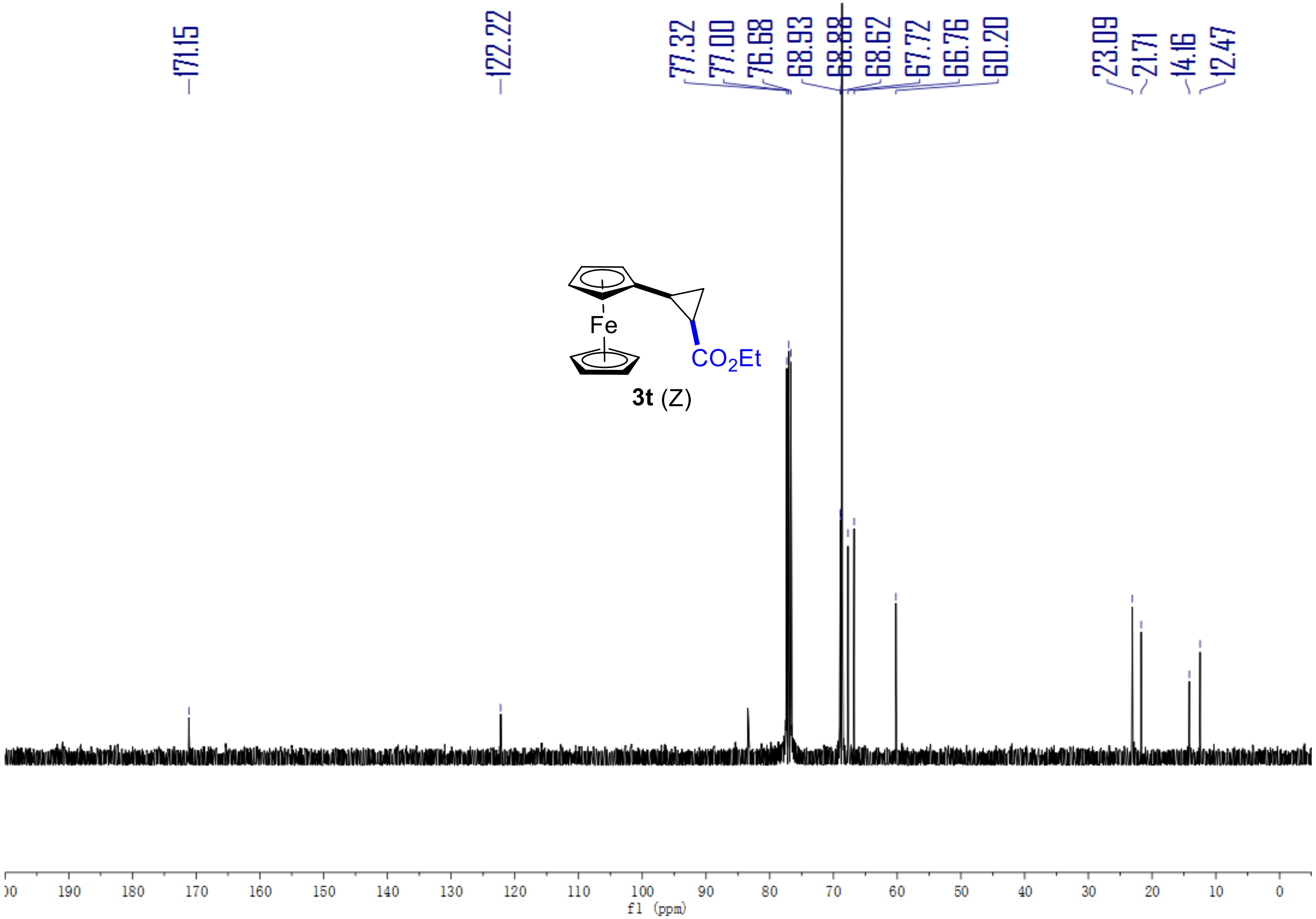

Supplementary Figure 76. ^{13}C NMR of **3t** (Z)

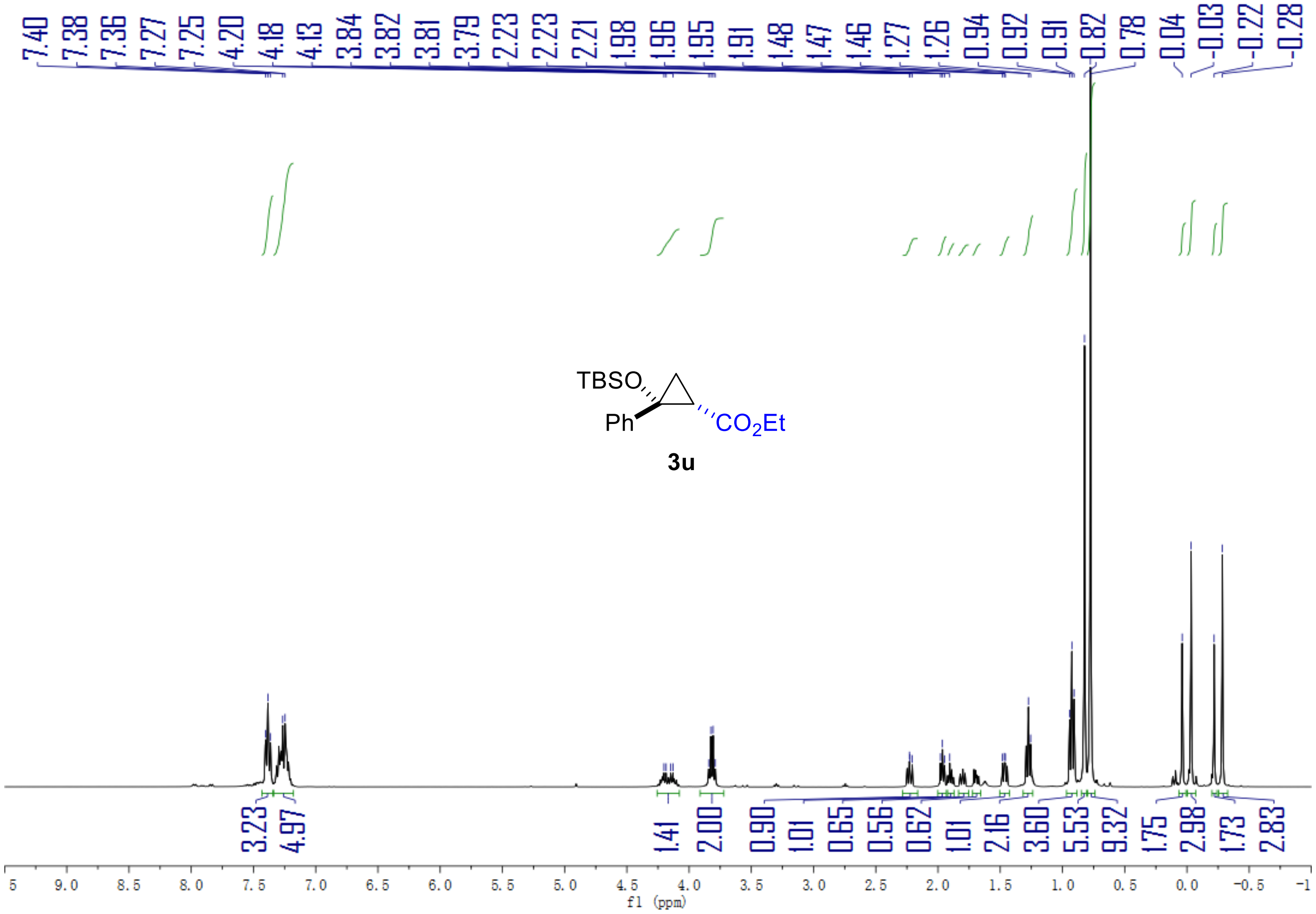

Supplementary Figure 77. ^{1}H NMR of **3u**

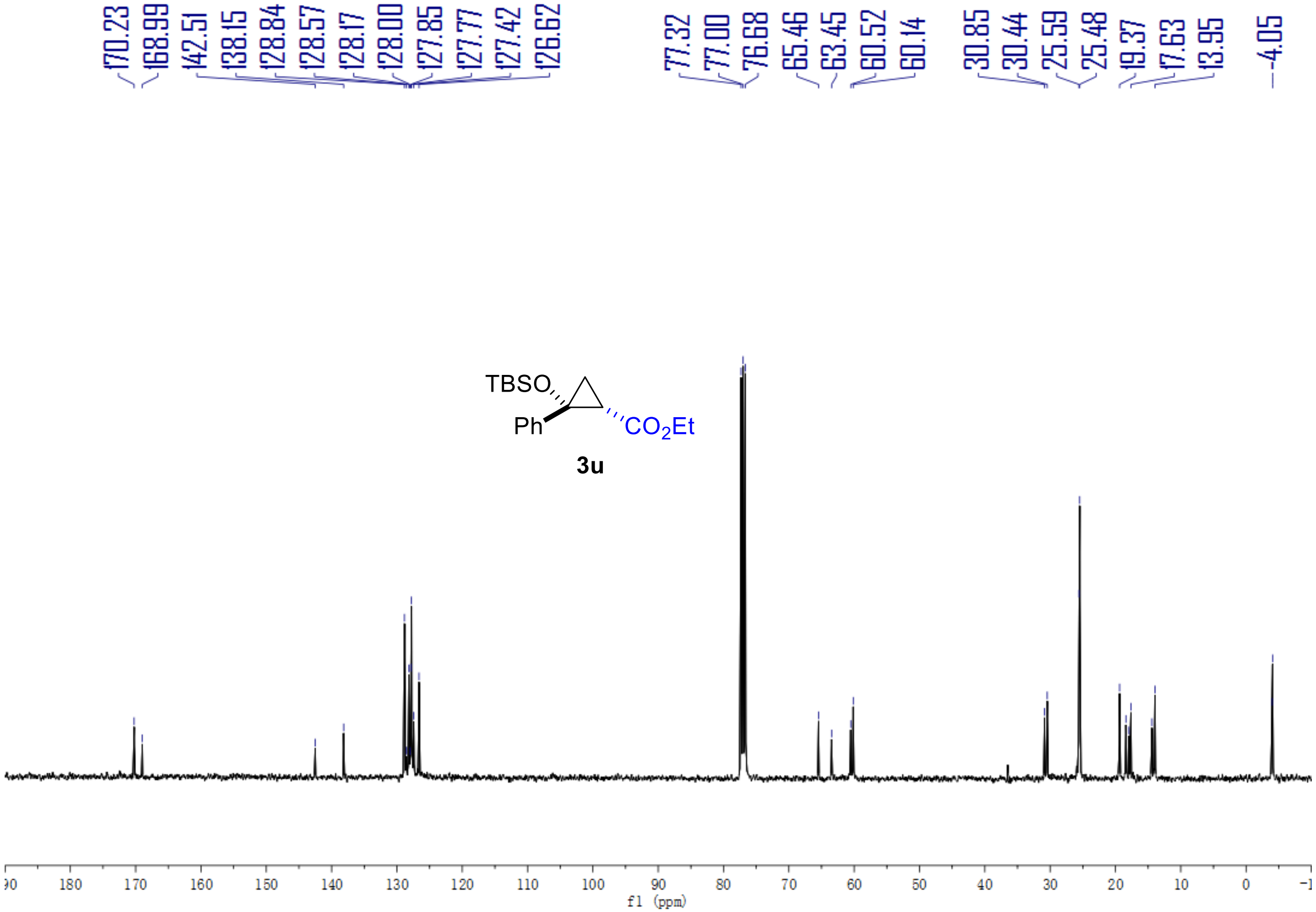

Supplementary Figure 78. ^{13}C NMR of **3u**

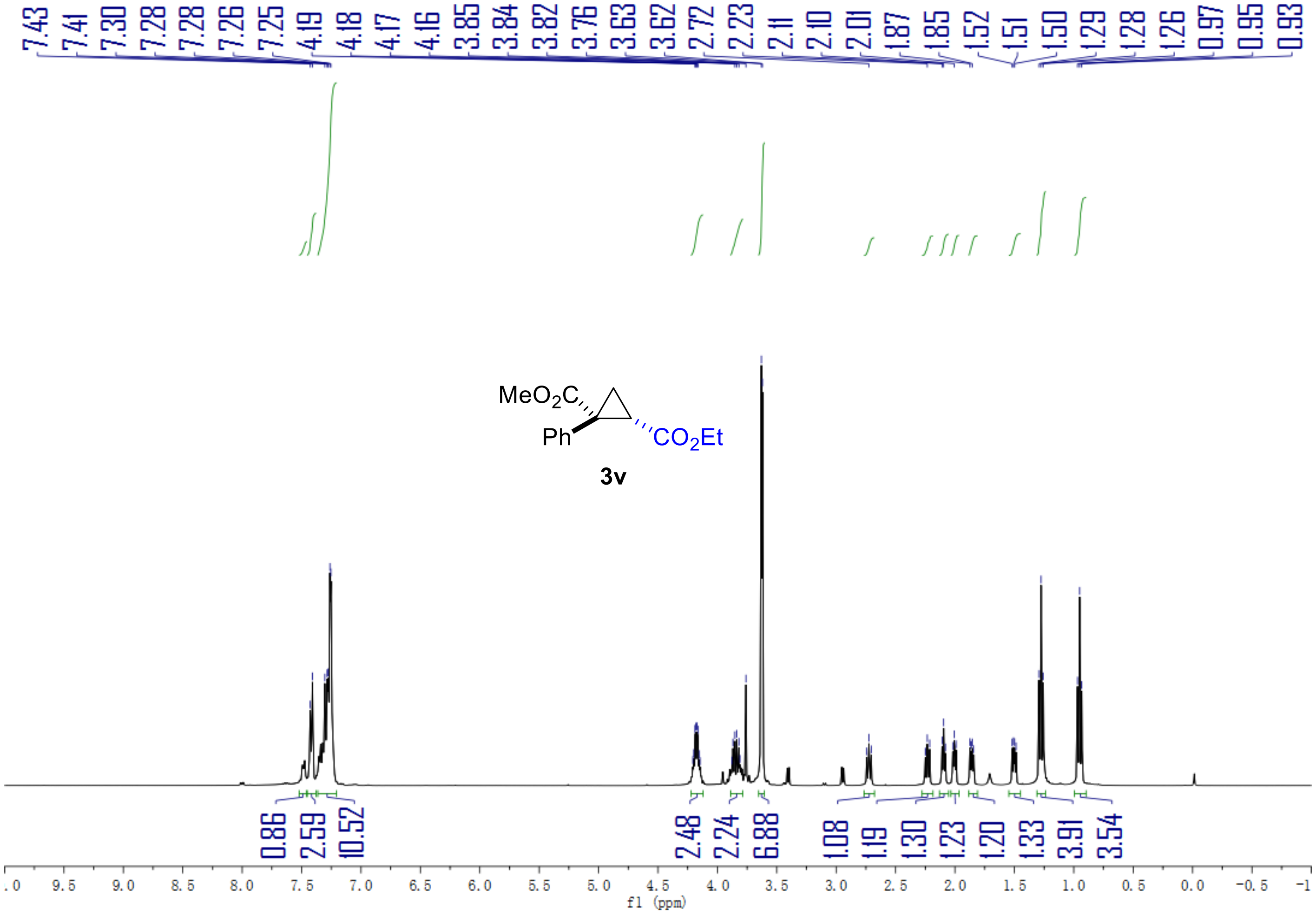

Supplementary Figure 79. ^{1}H NMR of **3v**

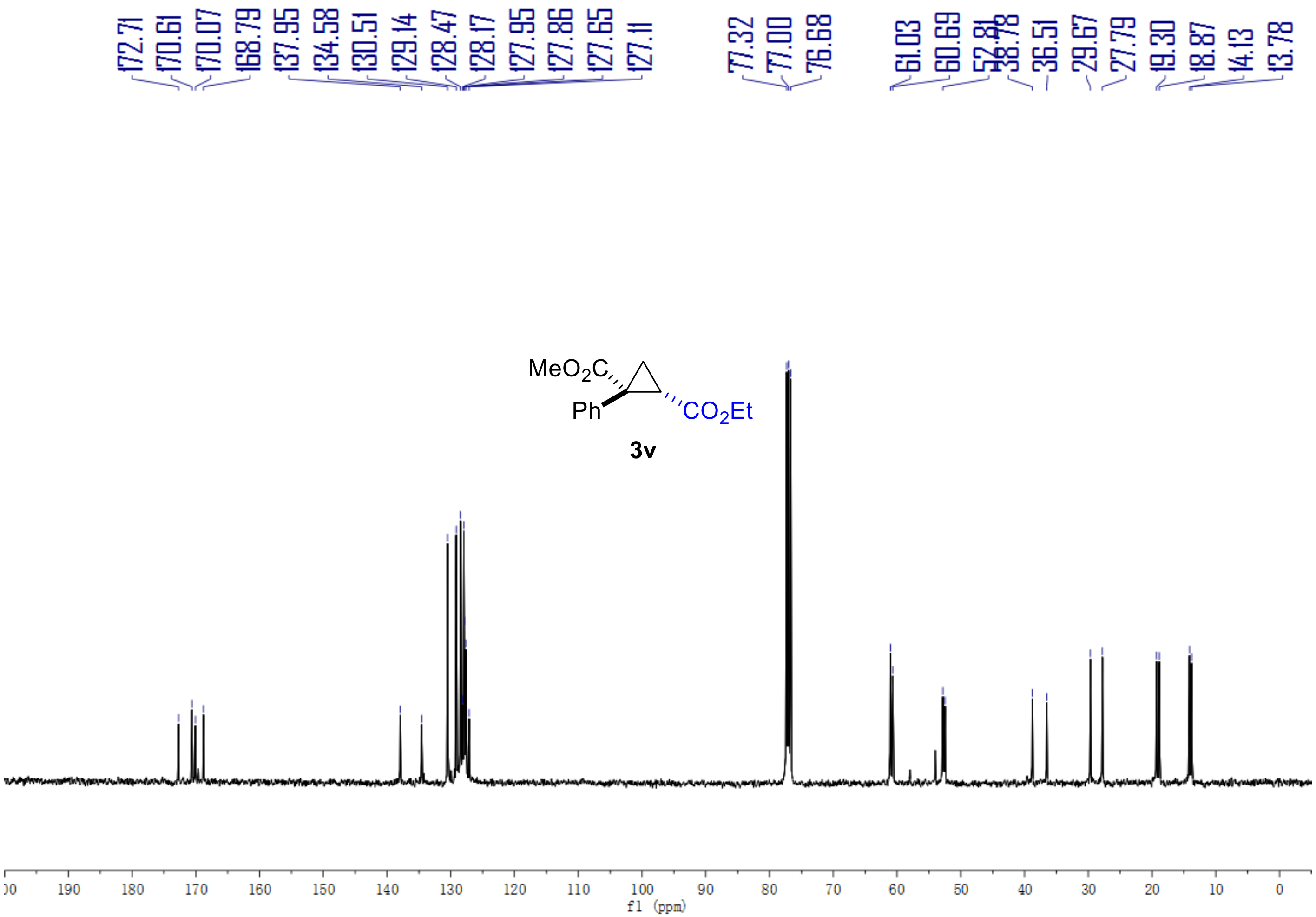

Supplementary Figure 80. ^{13}C NMR of **3v**

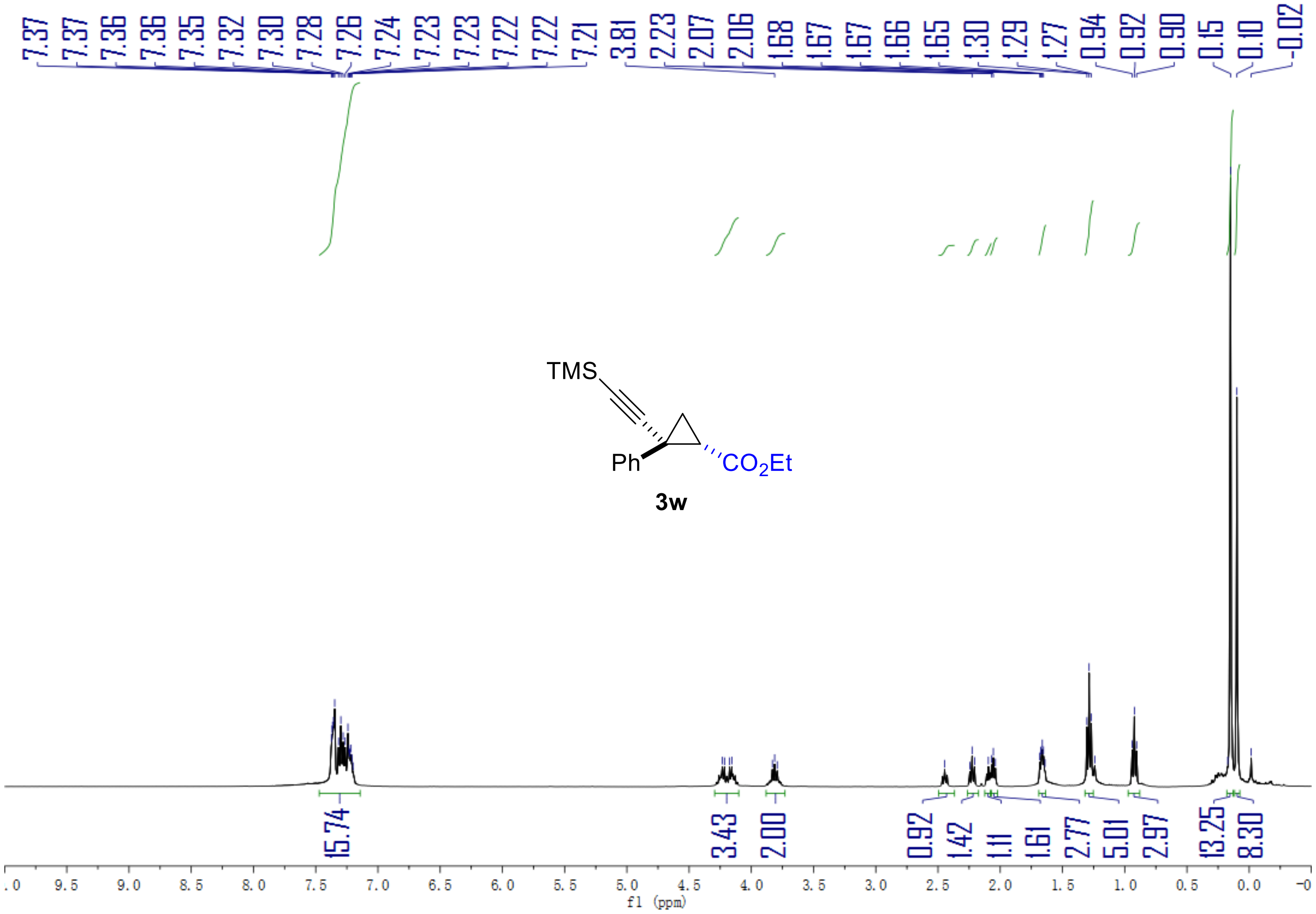

Supplementary Figure 81. ^{1}H NMR of **3w**

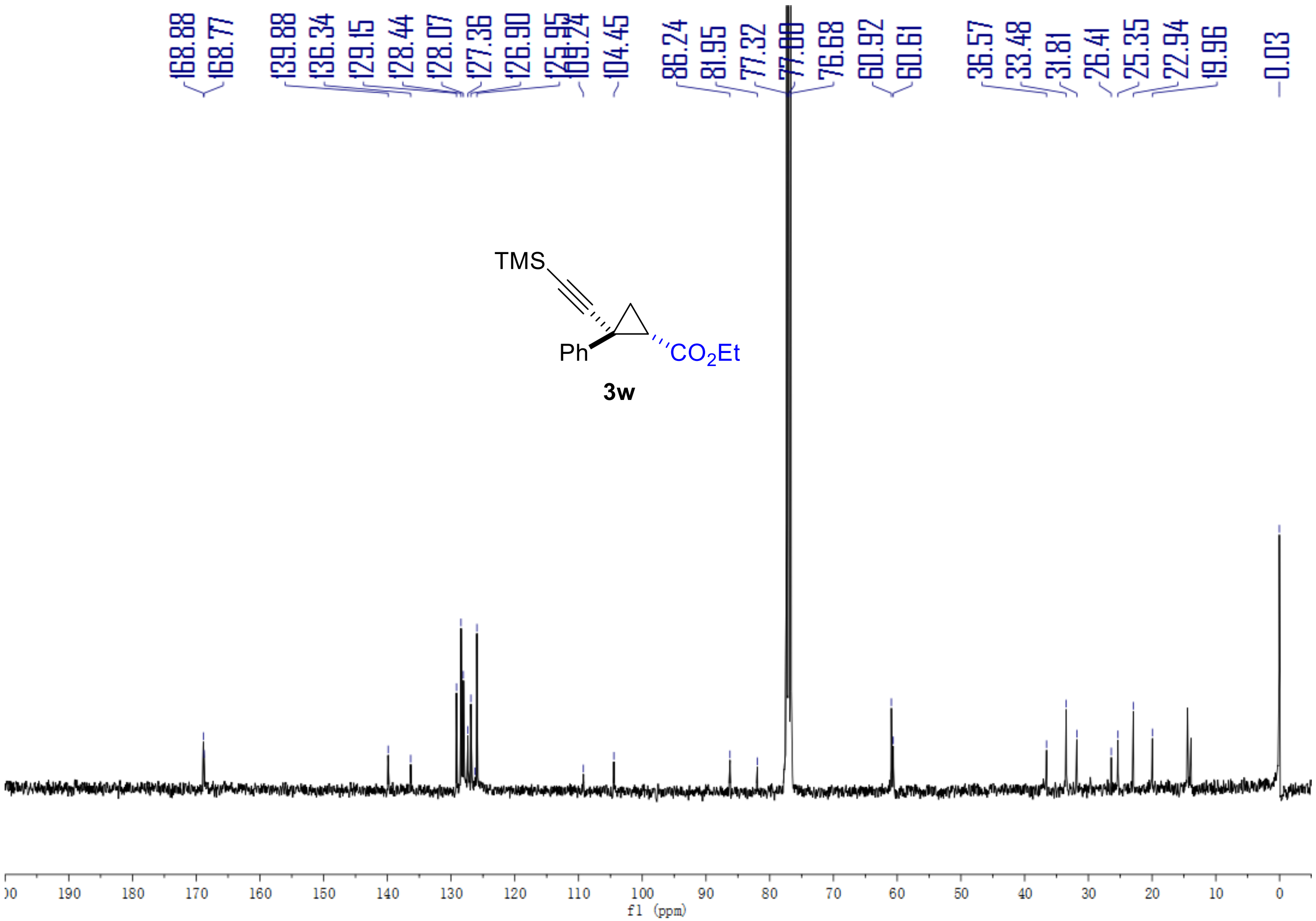

Supplementary Figure 82. ^{13}C NMR of **3w**

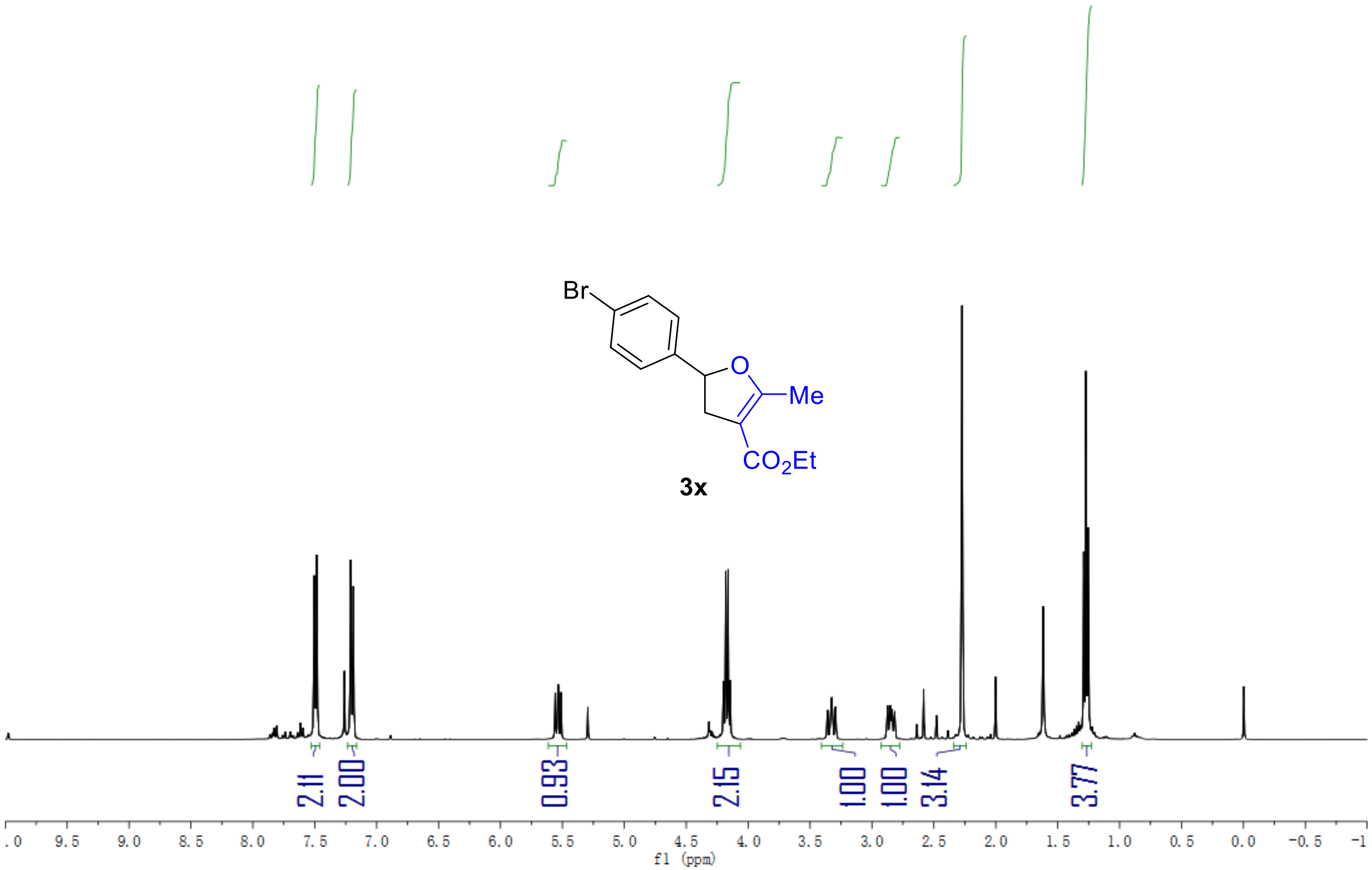

Supplementary Figure 83. ^{1}H NMR of **3x**

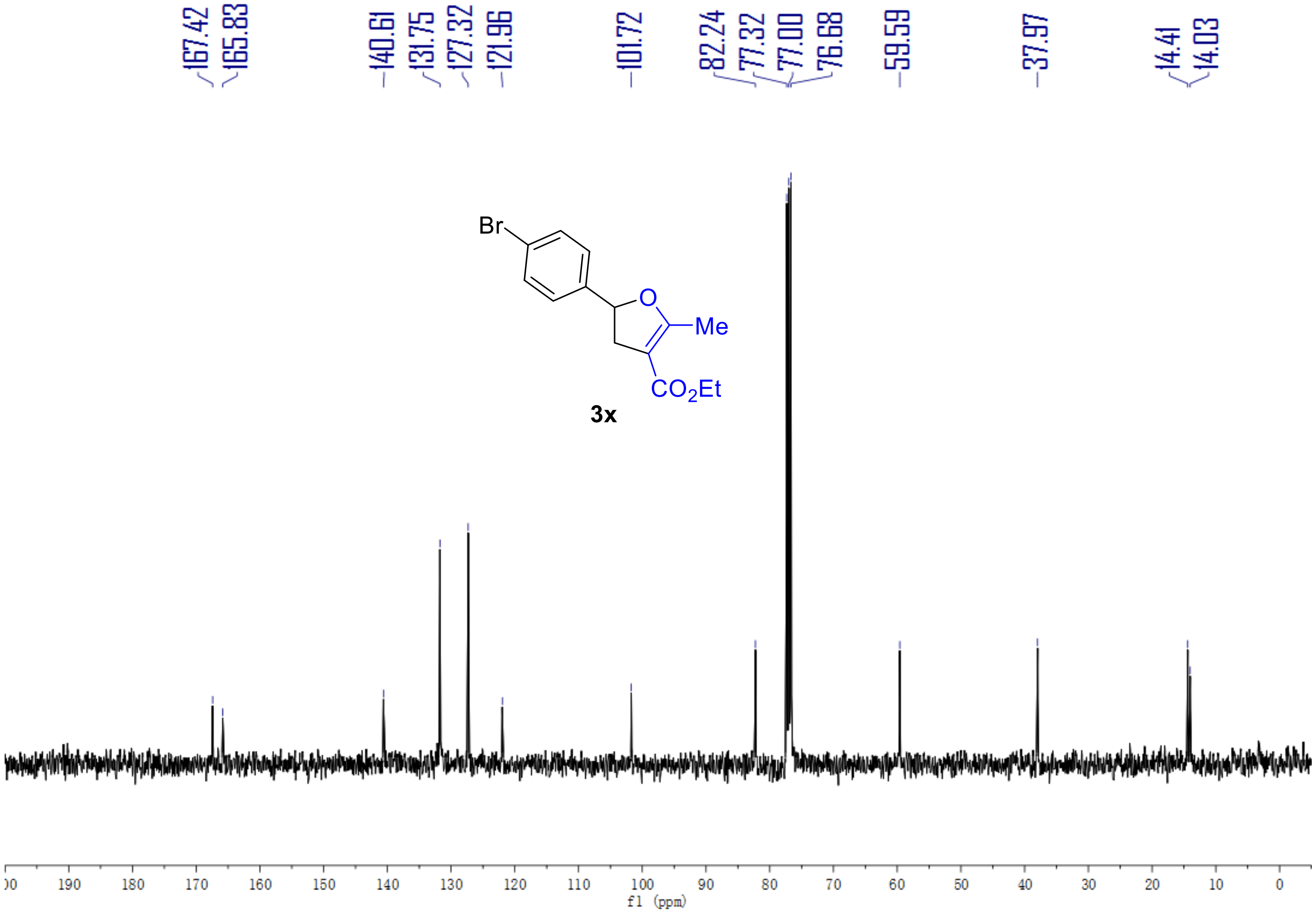

Supplementary Figure 84. ^{13}C NMR of **3x**

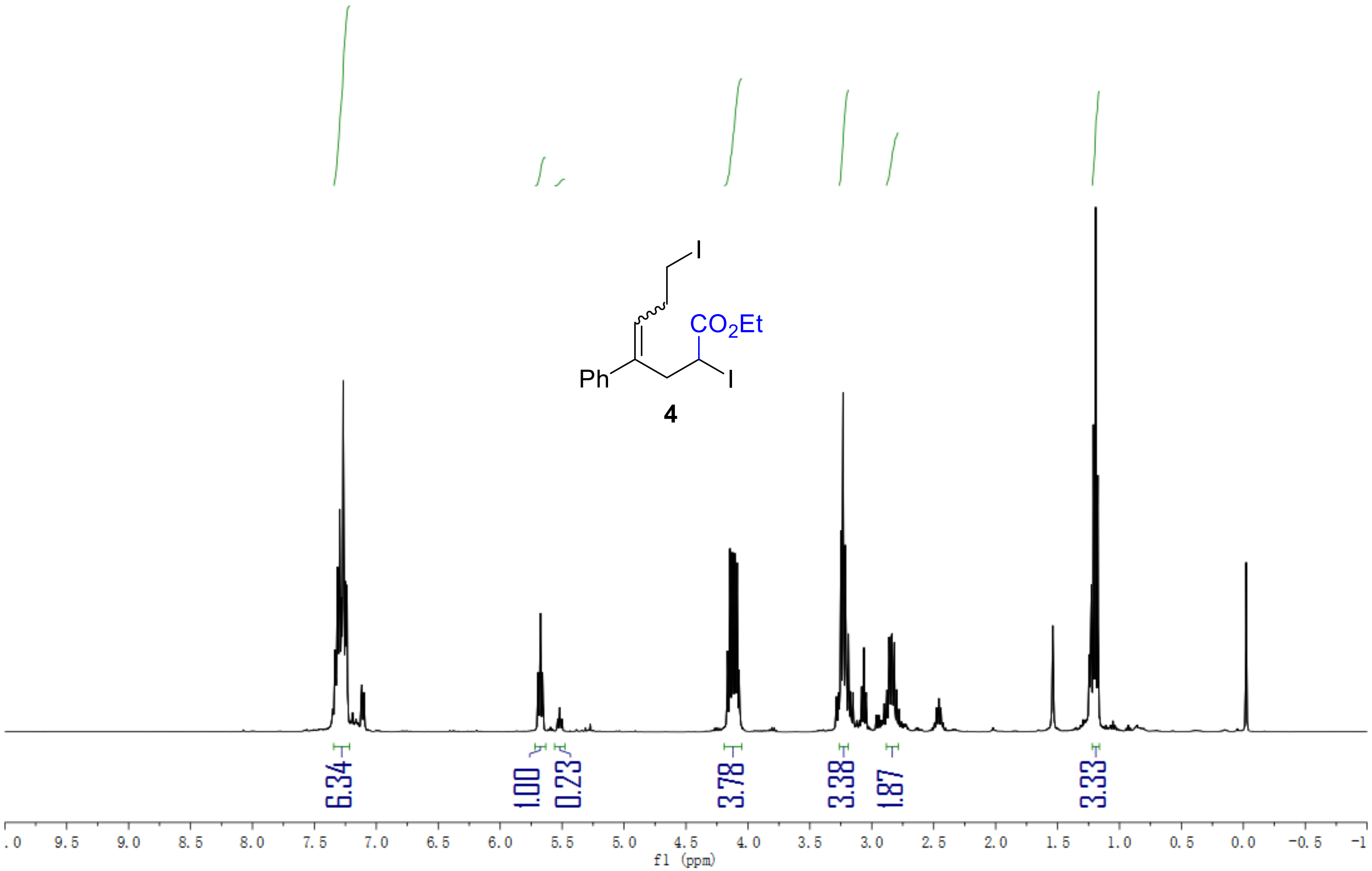

Supplementary Figure 85. ^{1}H NMR of **4**

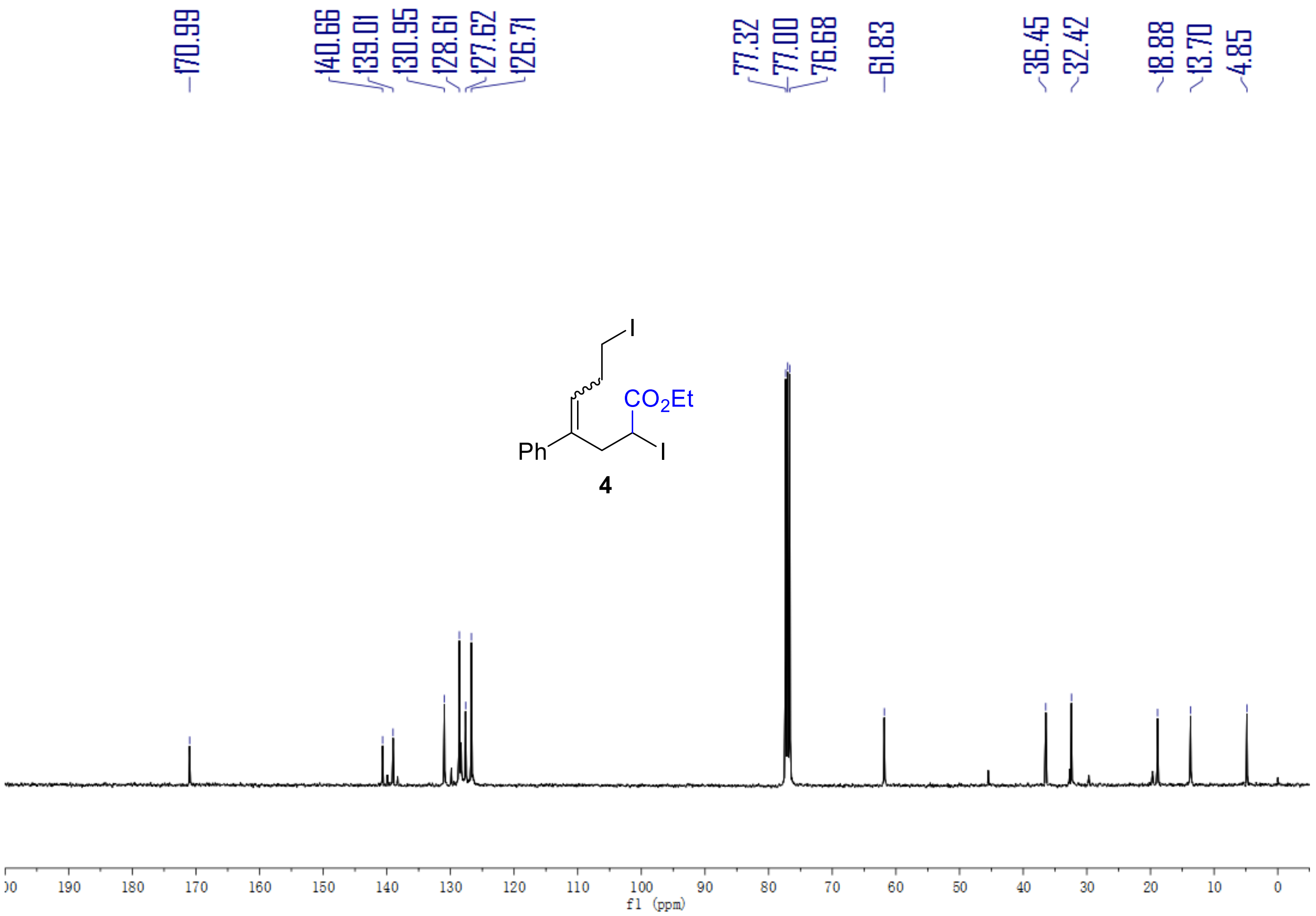

Supplementary Figure 86. ^{13}C NMR of **4**

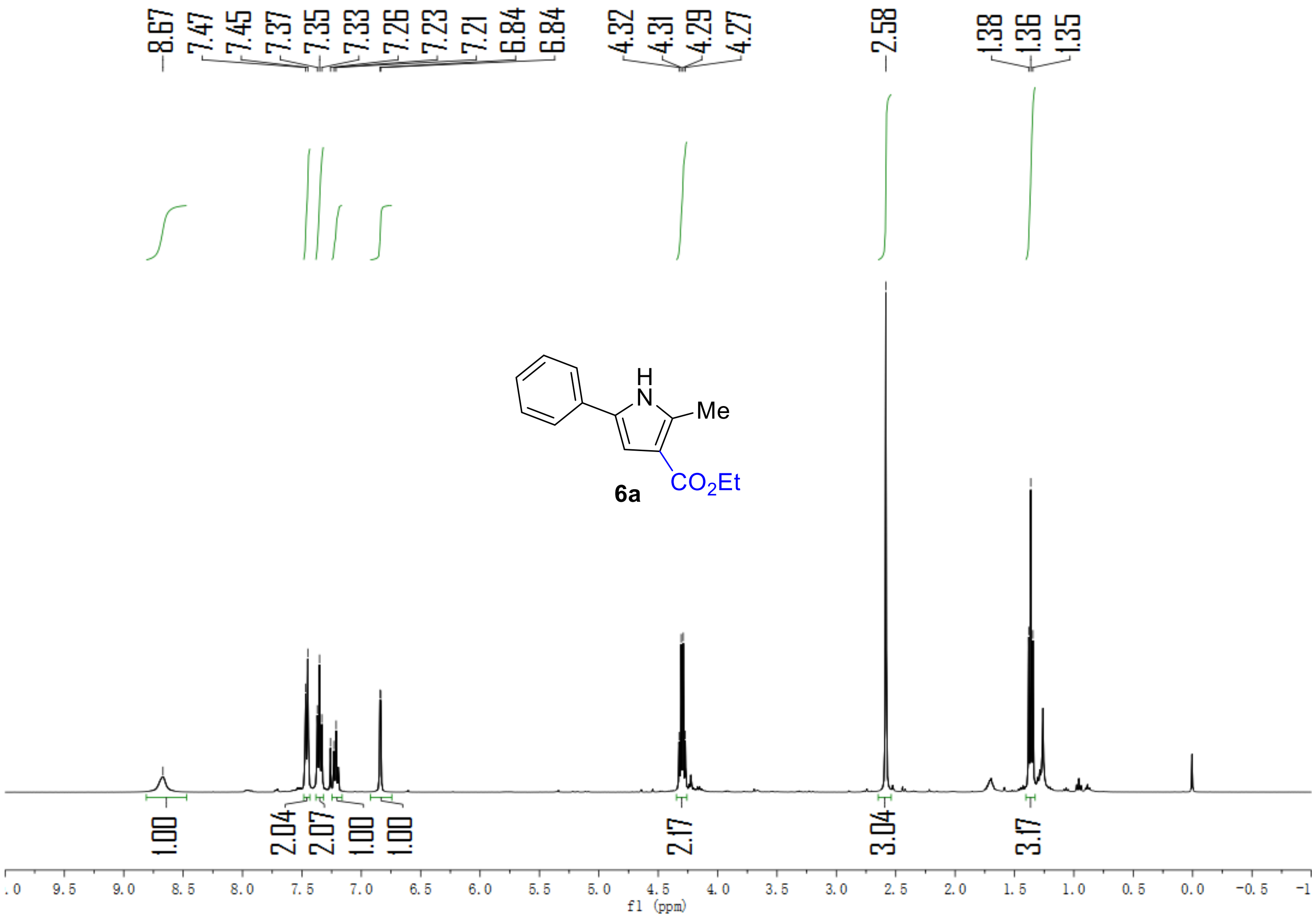

Supplementary Figure 87. ^{1}H NMR of **6a**

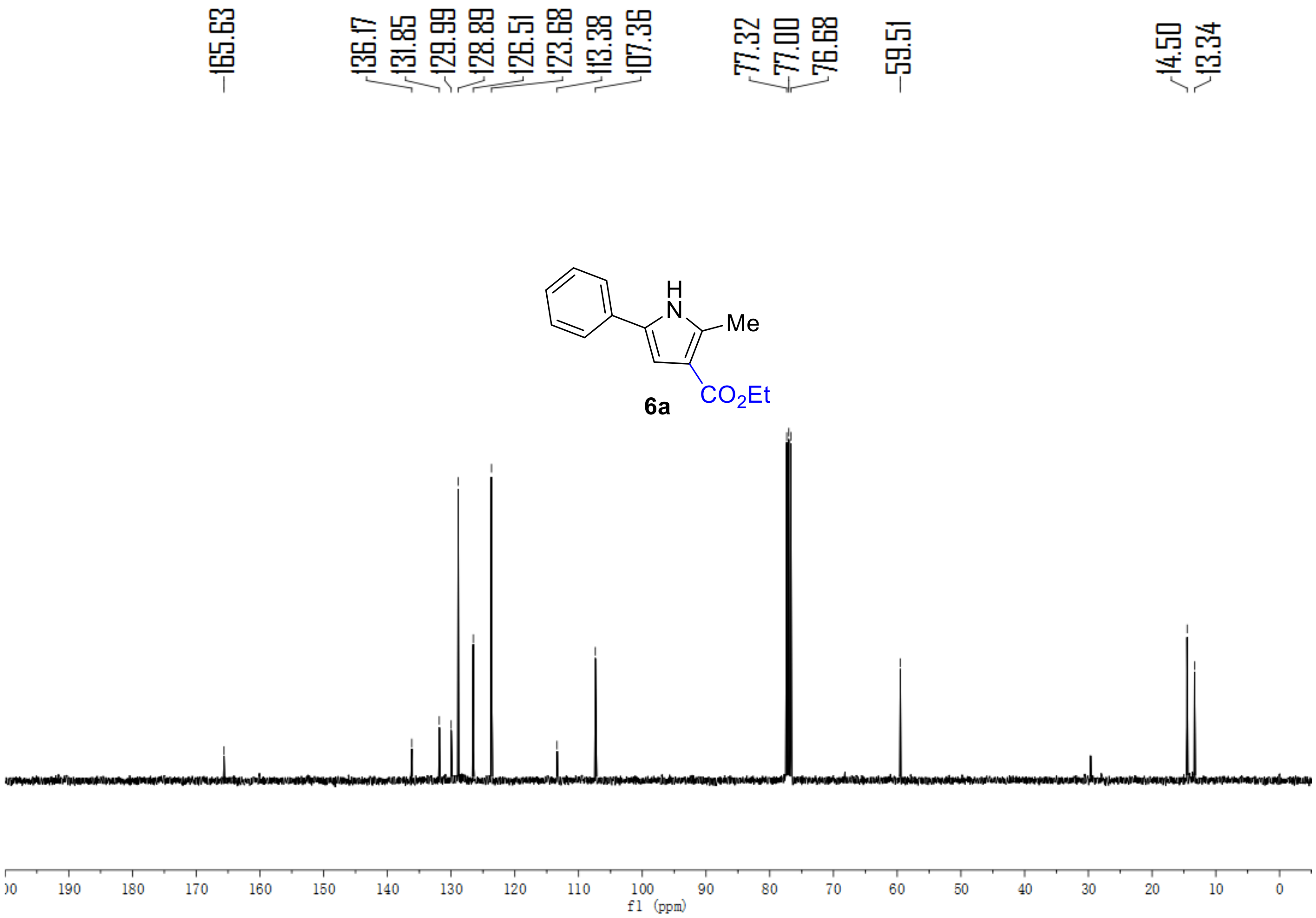

Supplementary Figure 88. ^{13}C NMR of **6a**

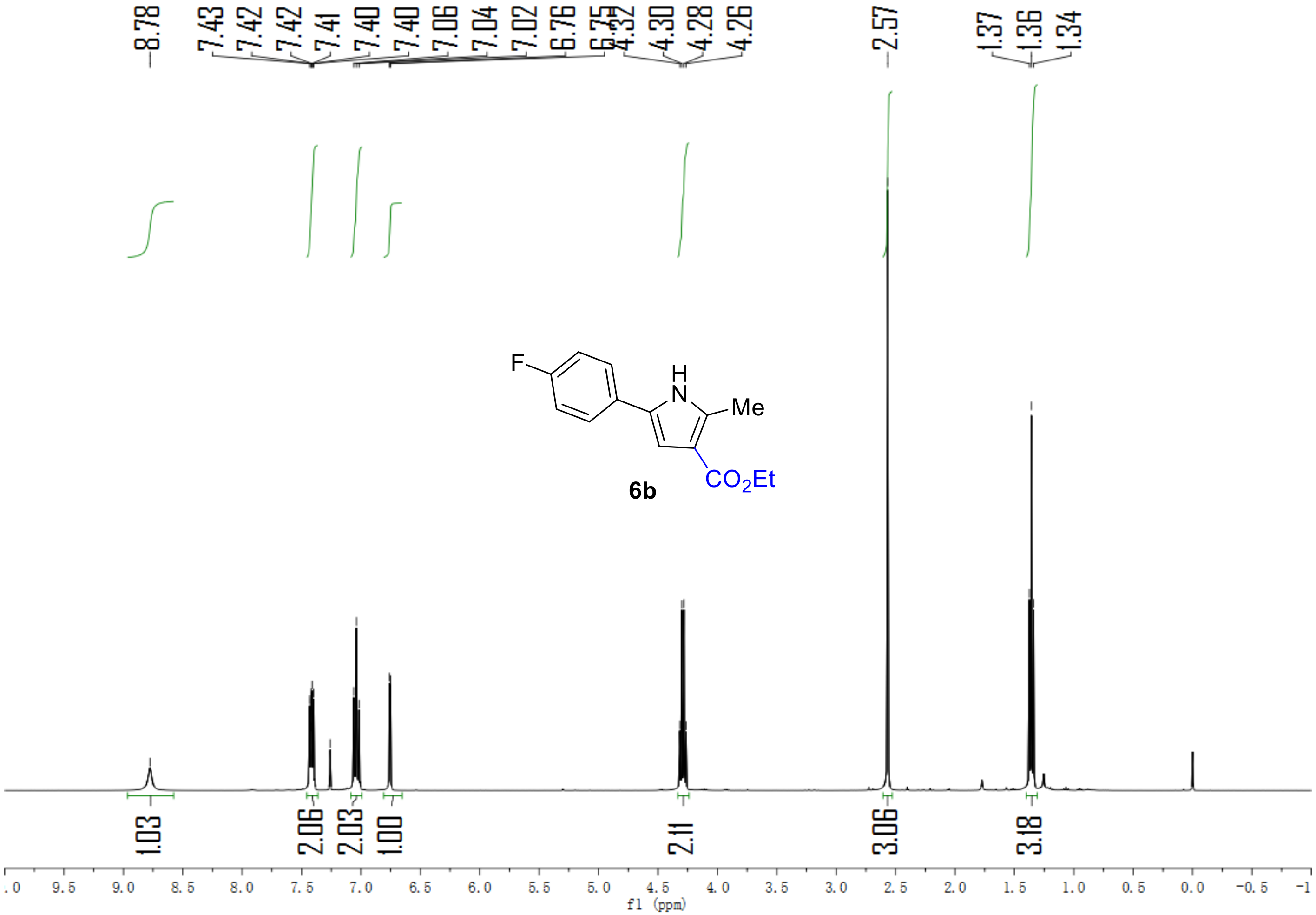

Supplementary Figure 89. ¹H NMR of **6b**

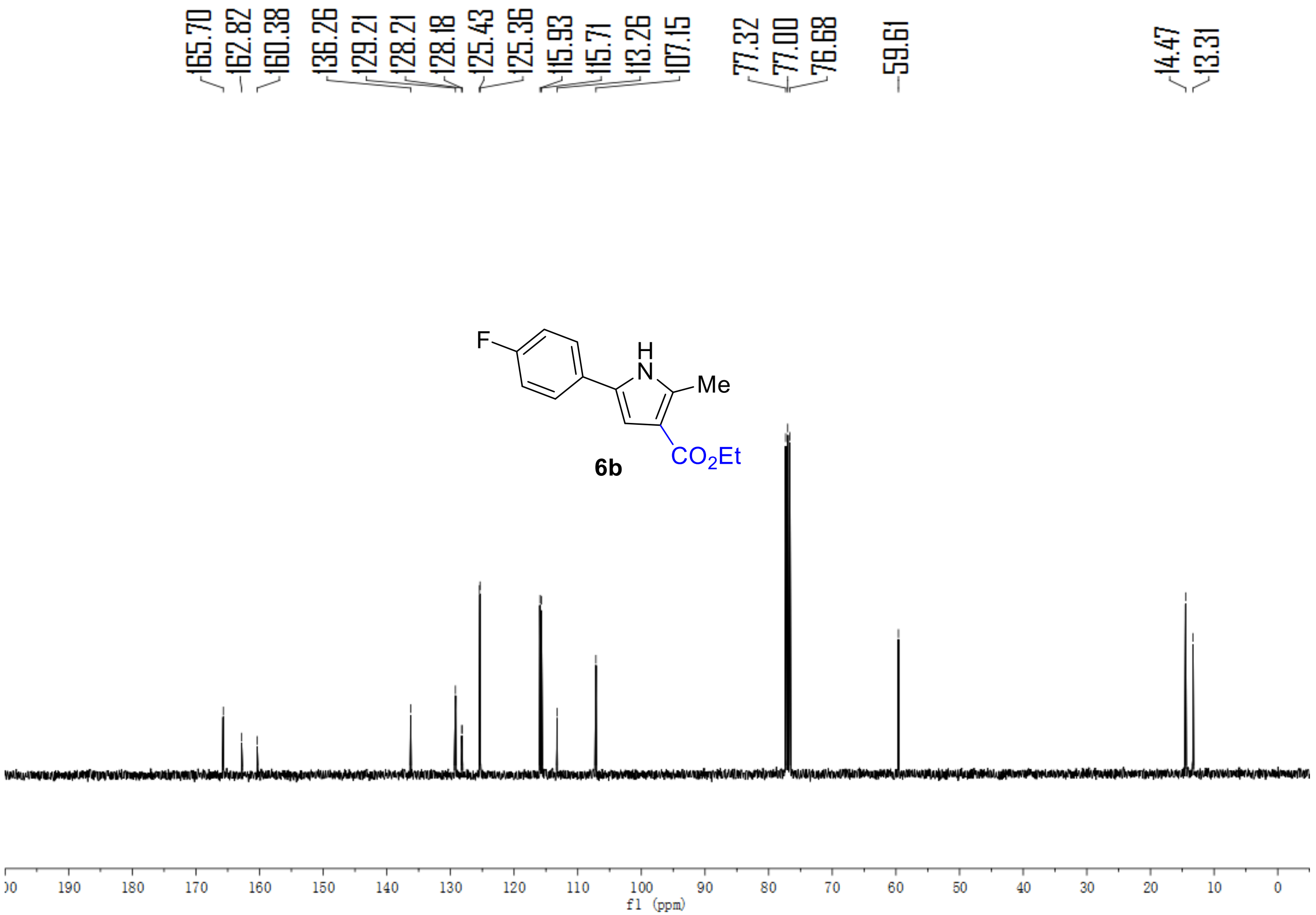

Supplementary Figure 90. ^{13}C NMR of **6b**

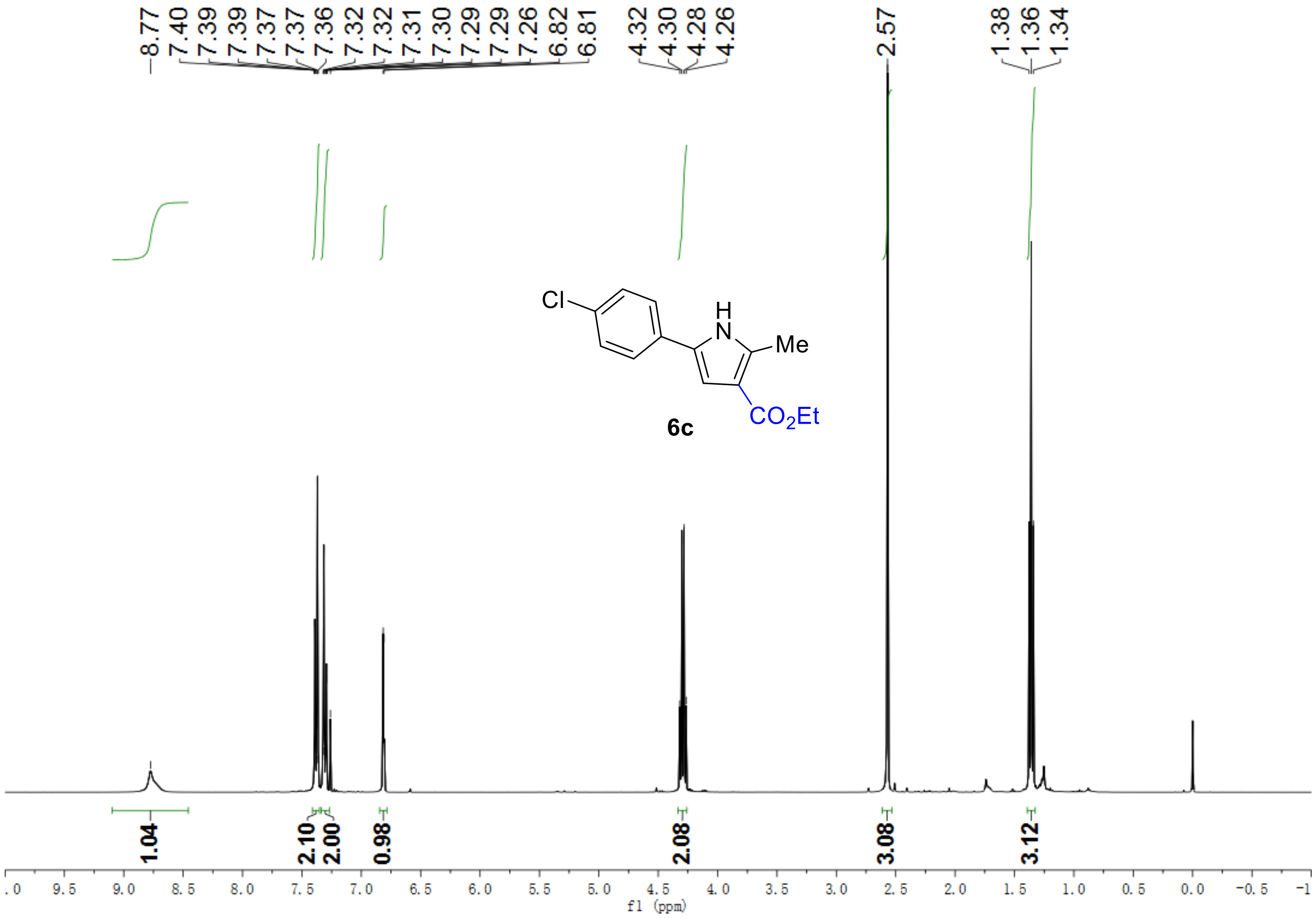

Supplementary Figure 91. ^{1}H NMR of **6c**

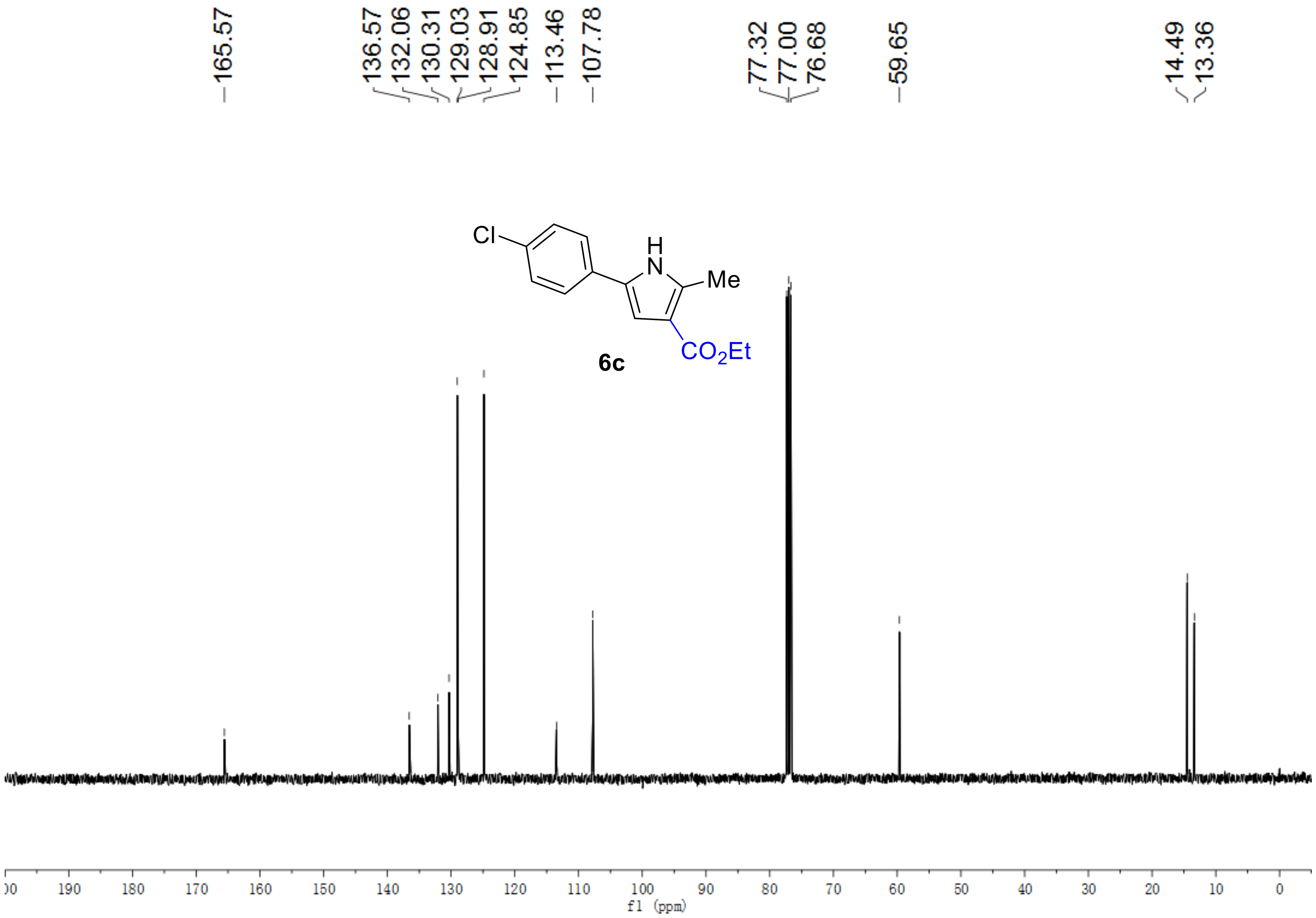

Supplementary Figure 92. ^{13}C NMR of **6c**

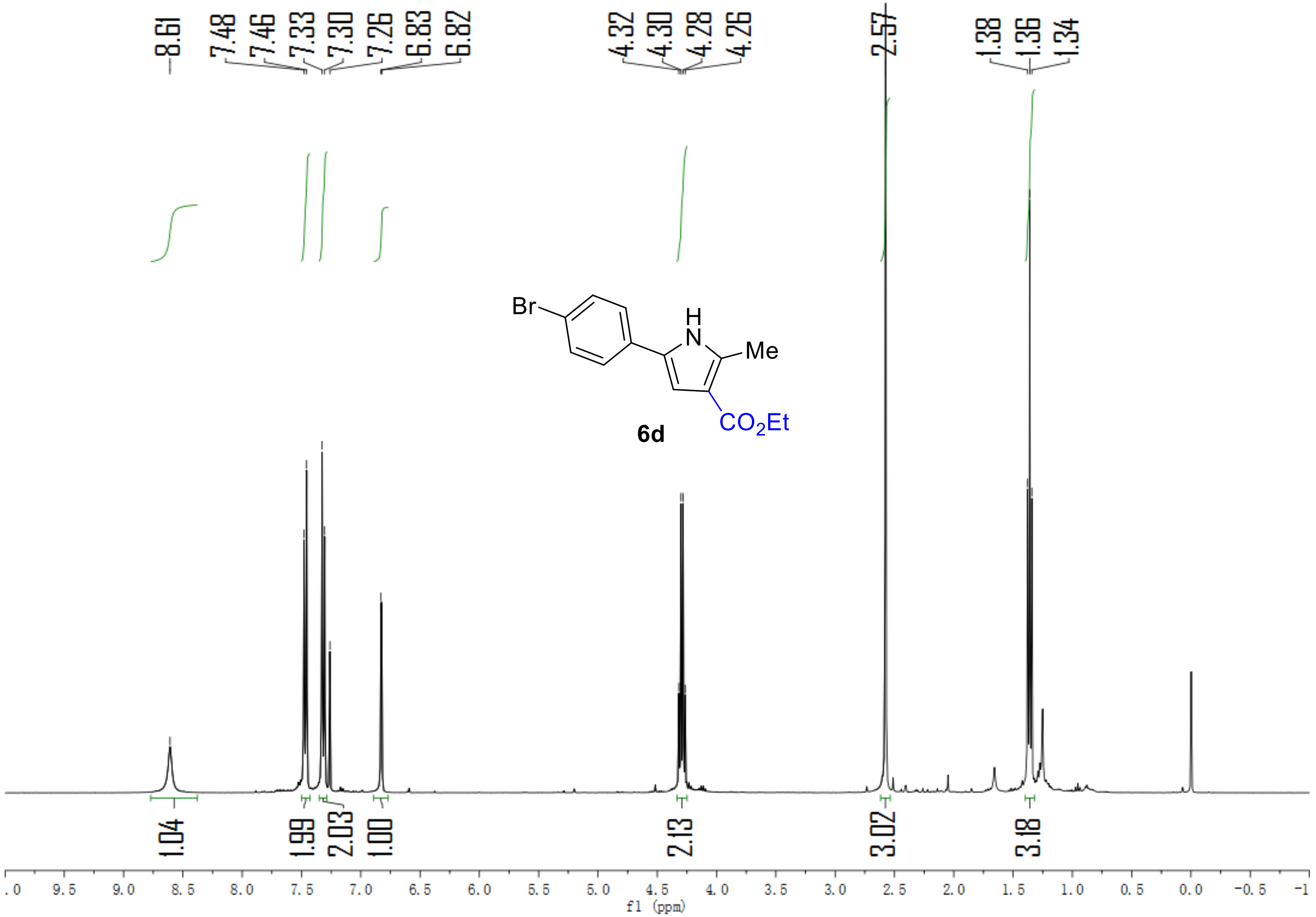

Supplementary Figure 93. ^{1}H NMR of **6d**

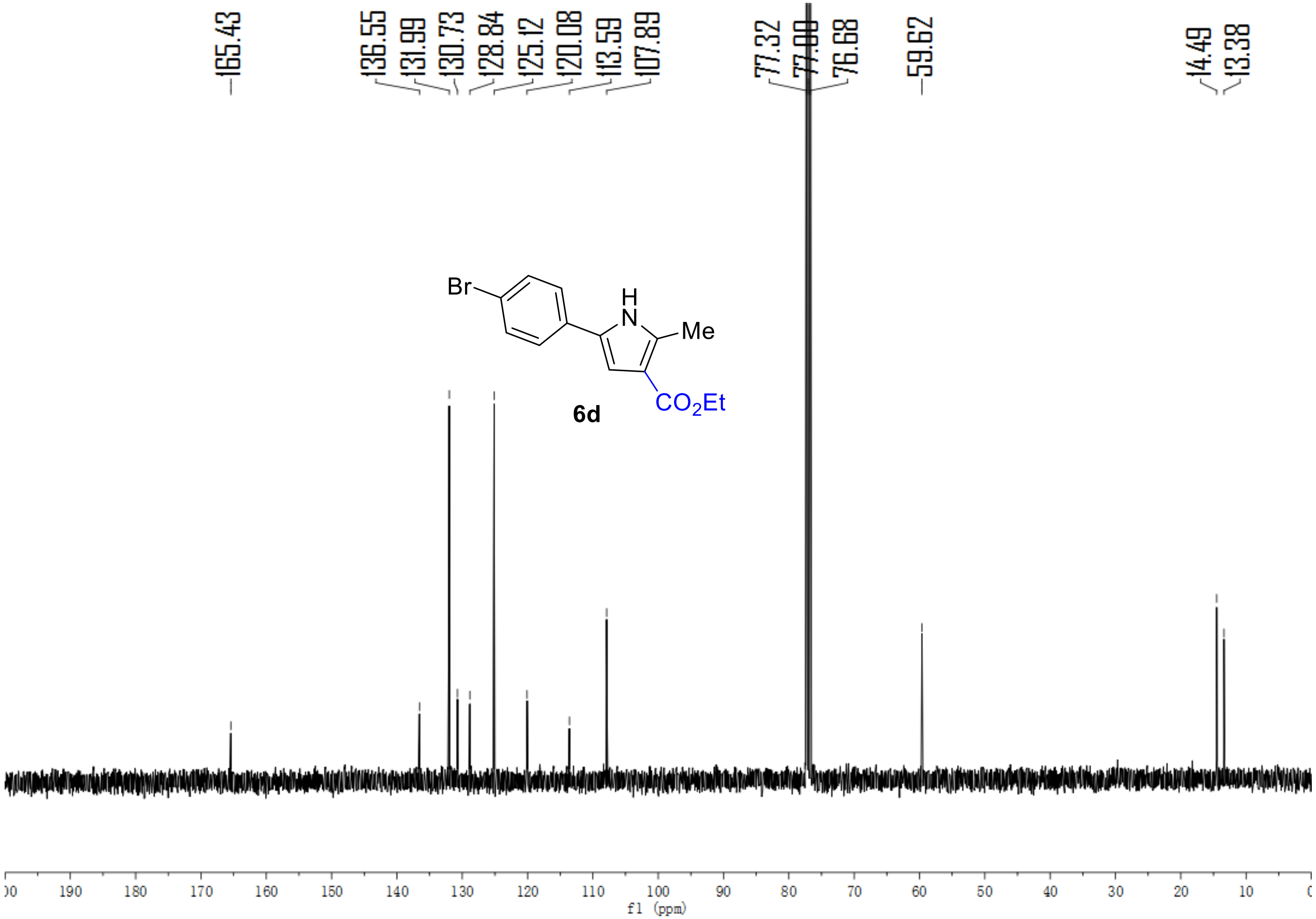

Supplementary Figure 94. ^{13}C NMR of **6d**

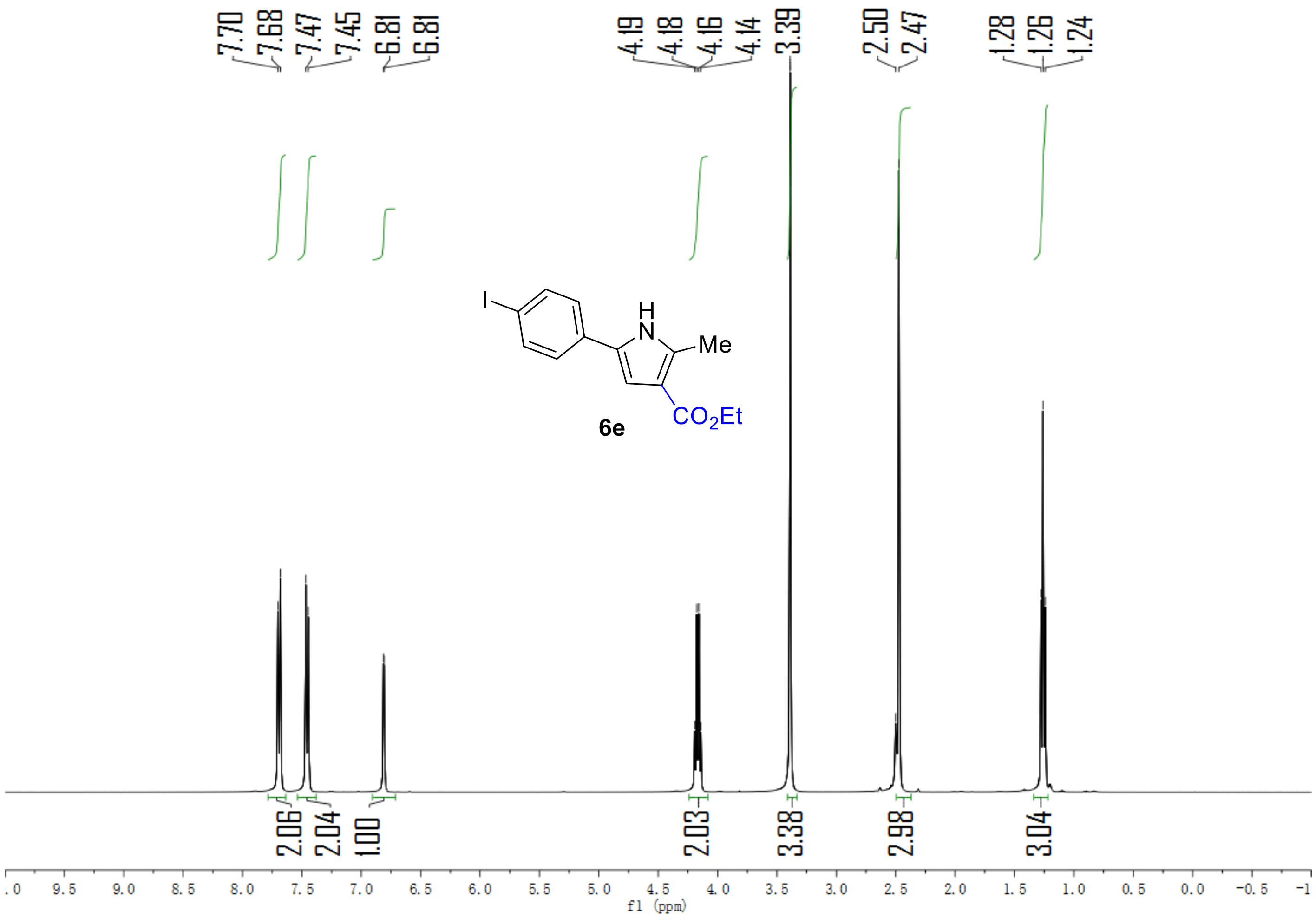

Supplementary Figure 95. ^{1}H NMR of **6e**

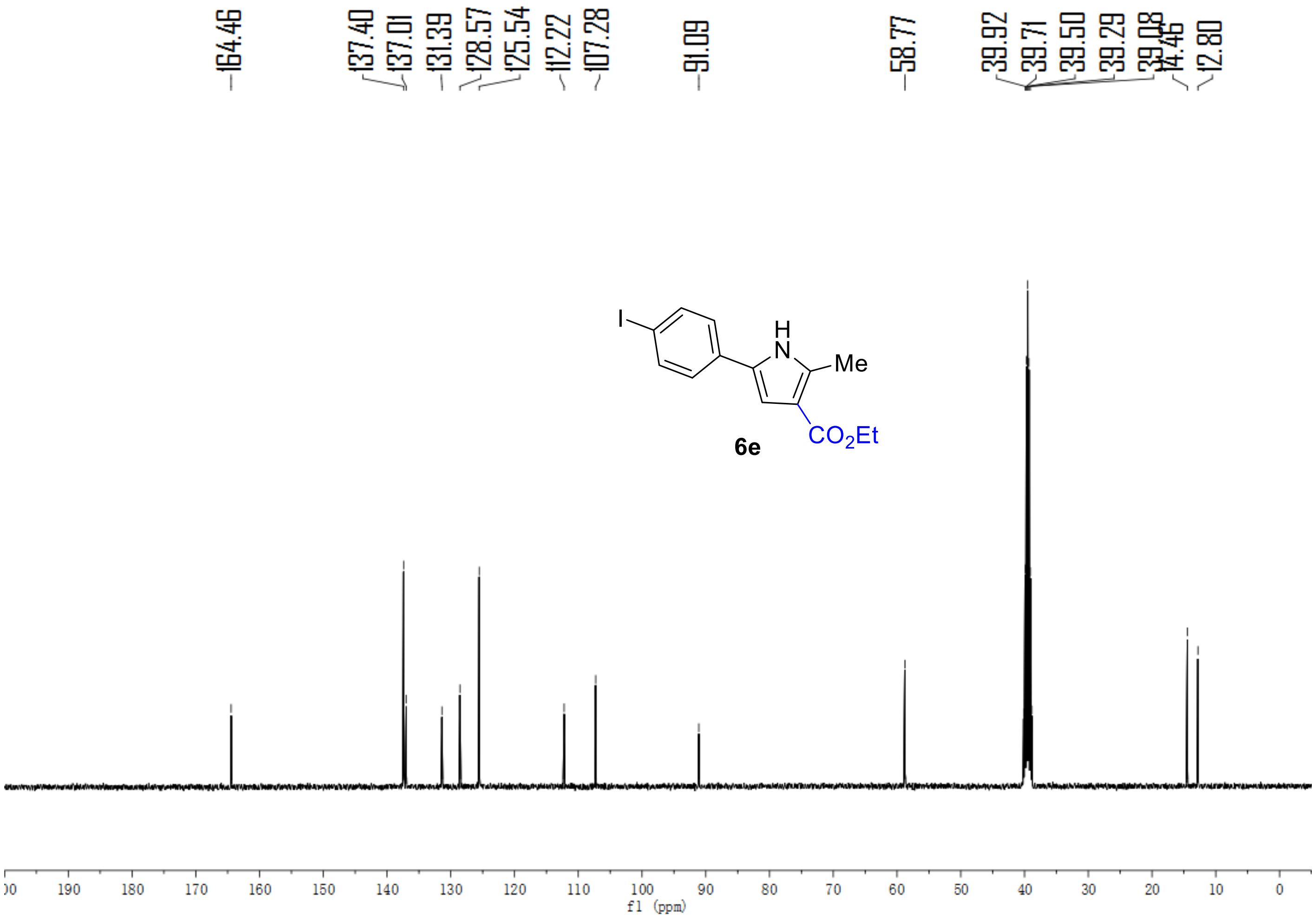

Supplementary Figure 96. ^{13}C NMR of **6e**

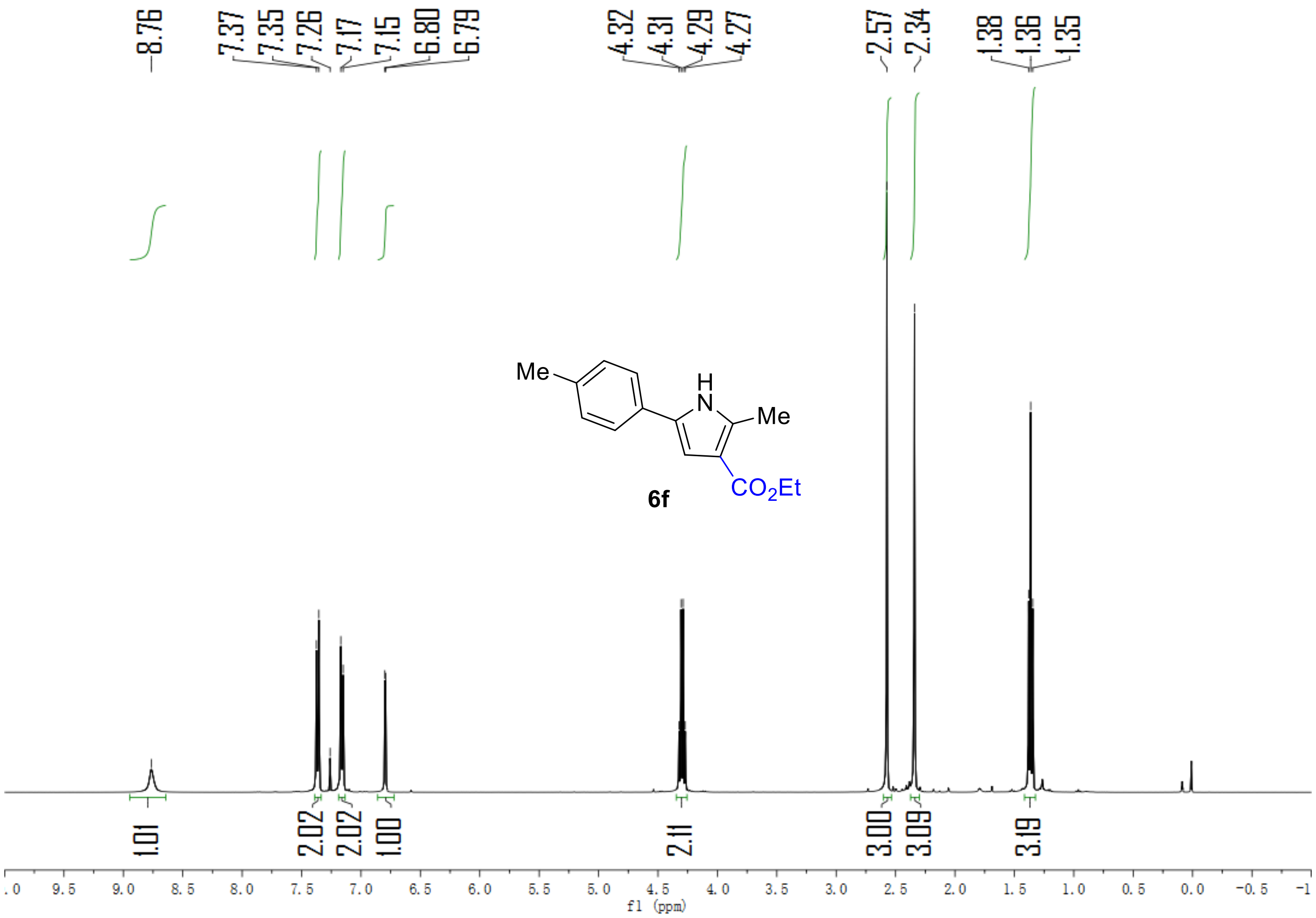

Supplementary Figure 97. ^{1}H NMR of **6f**

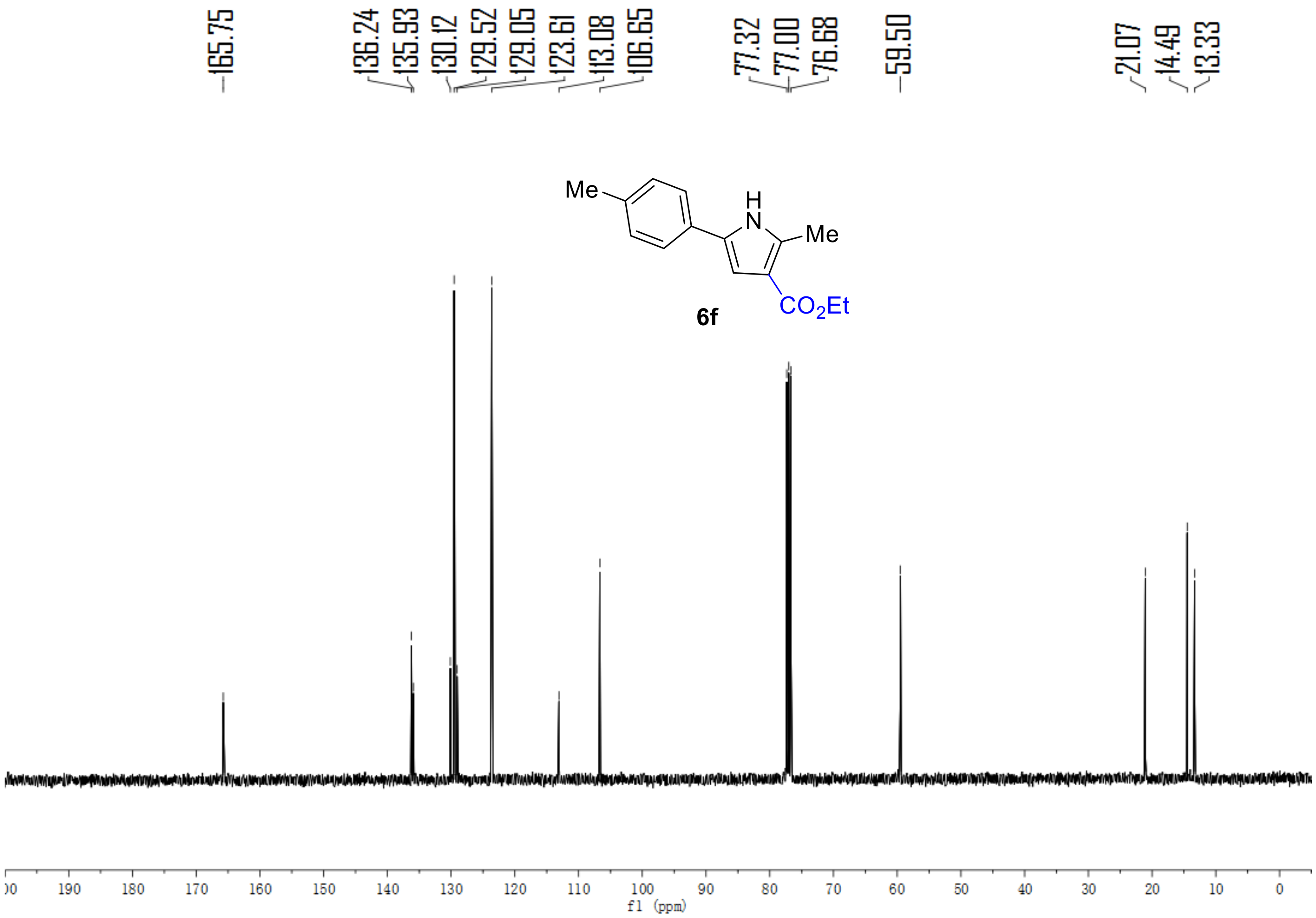

Supplementary Figure 98. ^{13}C NMR of **6f**

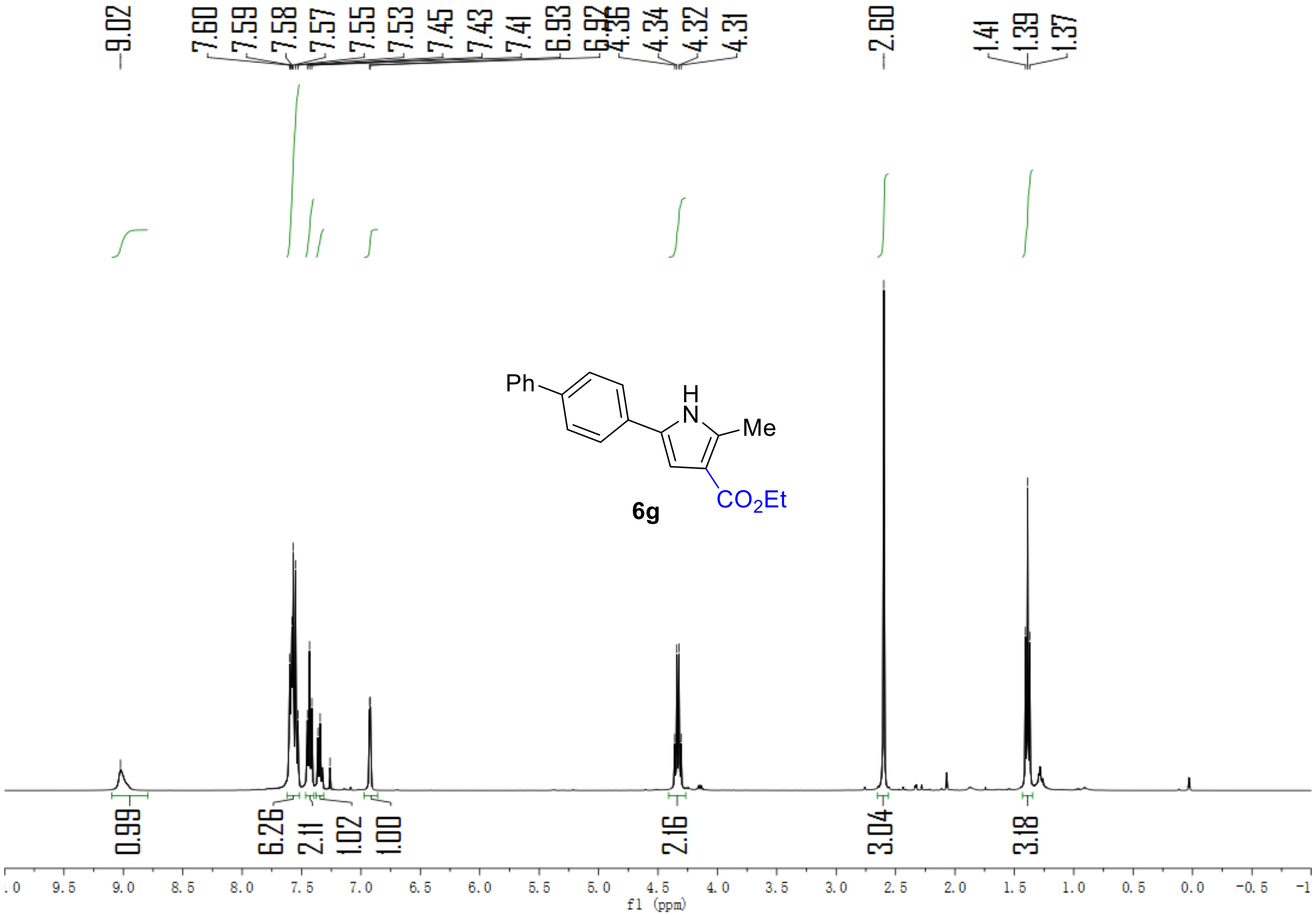

Supplementary Figure 99. ^{1}H NMR of **6g**

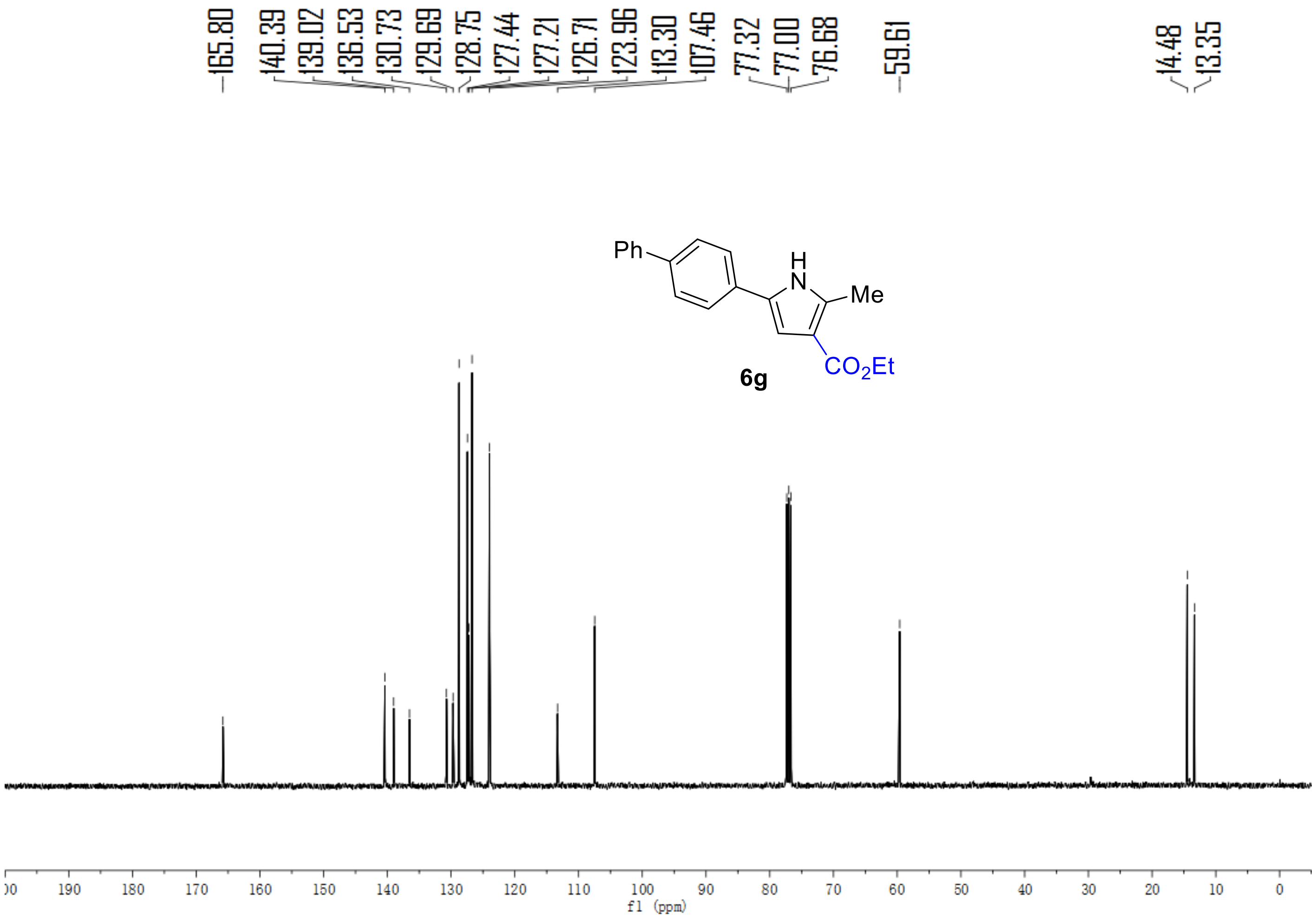

Supplementary Figure 100. ^{13}C NMR of **6g**

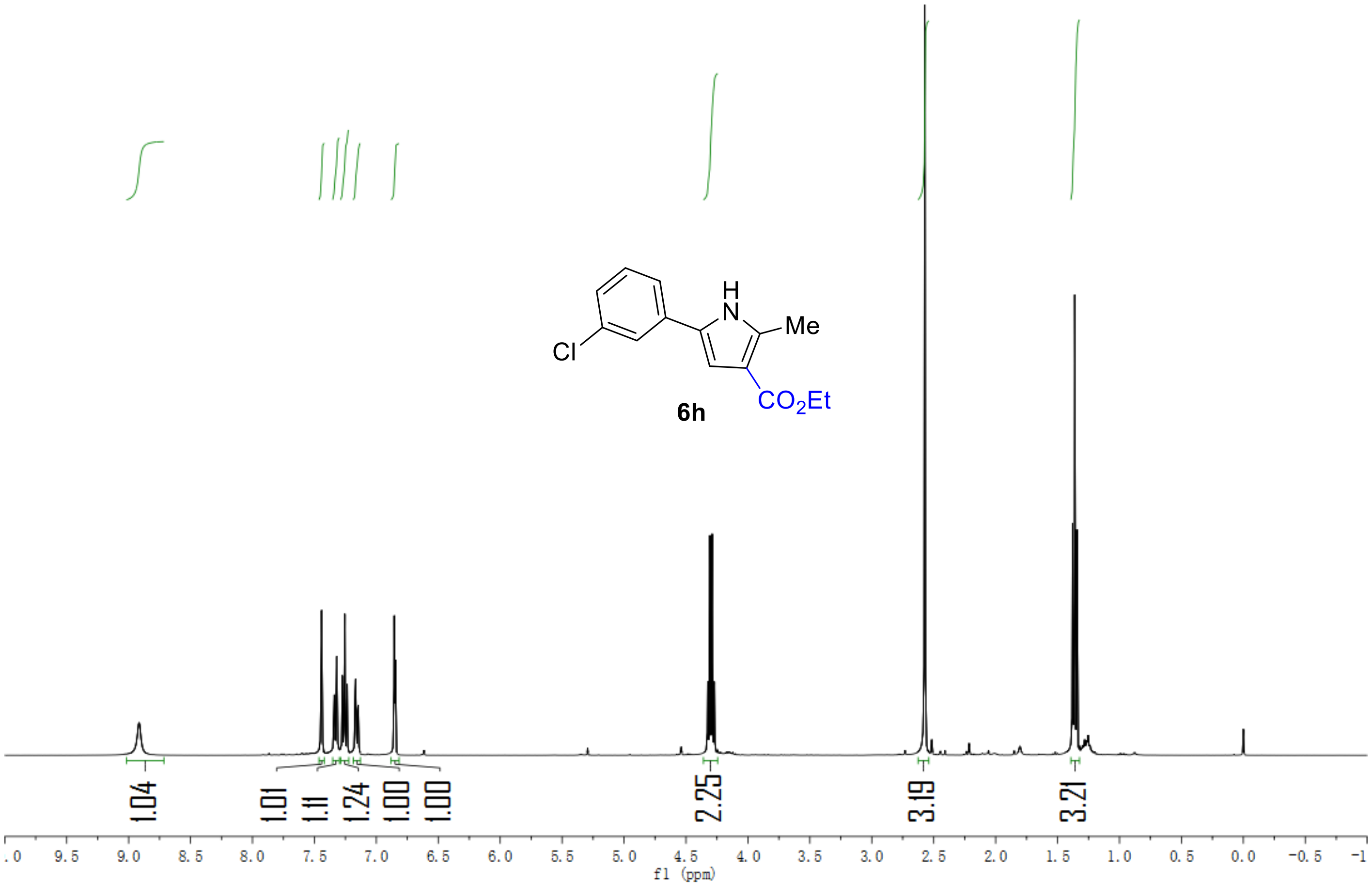

Supplementary Figure 101. ^{1}H NMR of **6h**

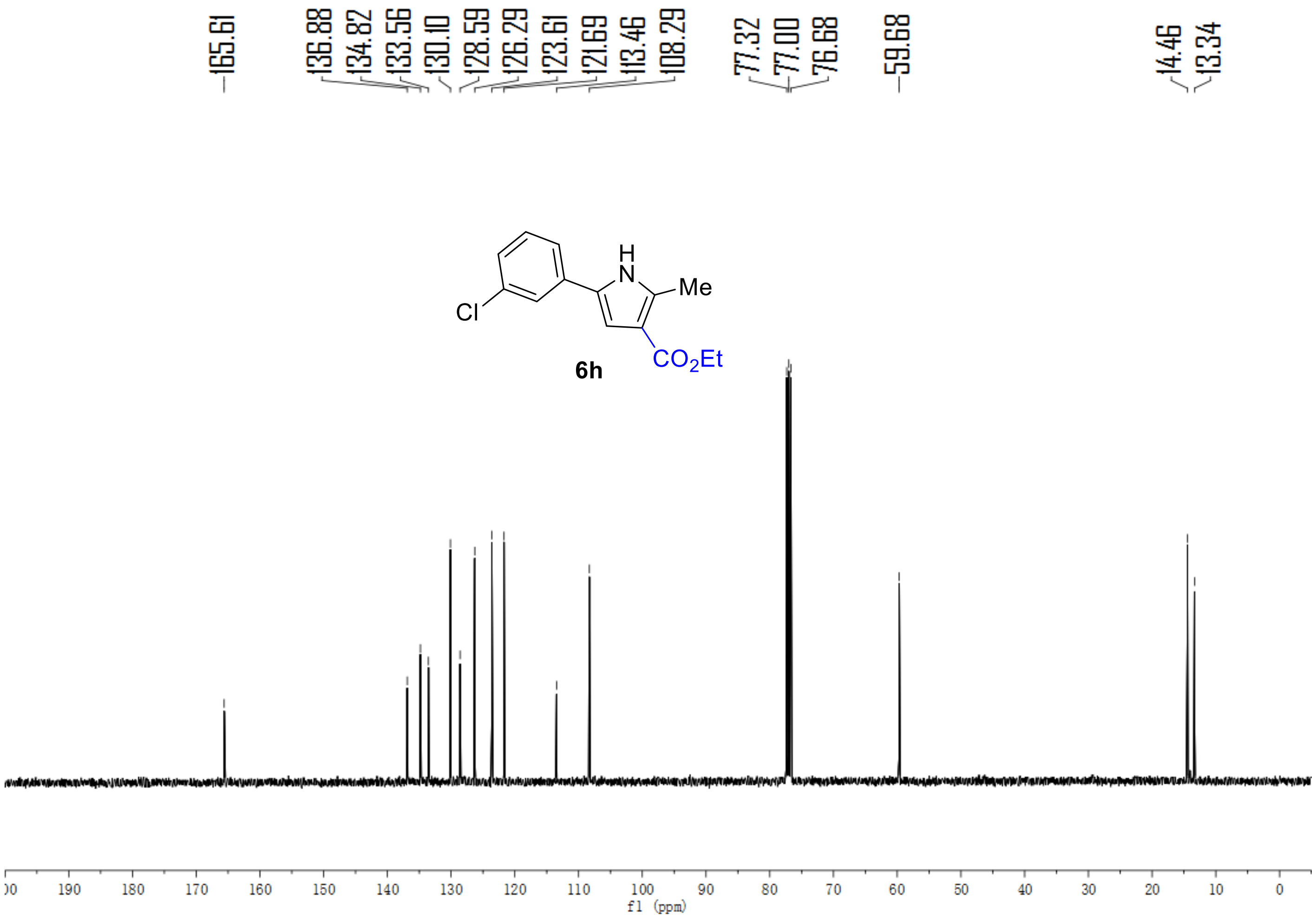

Supplementary Figure 102. ^{13}C NMR of **6h**

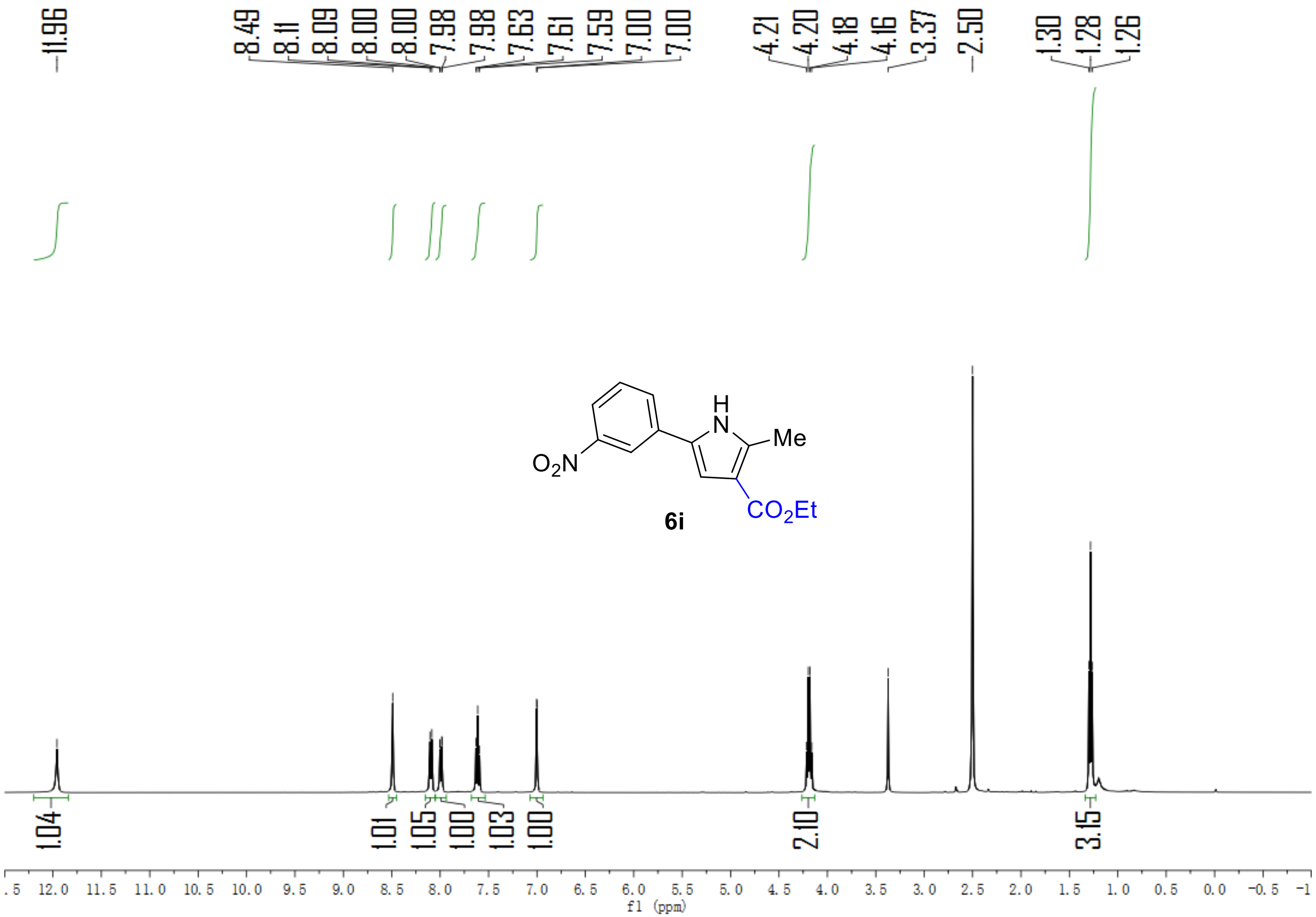

Supplementary Figure 103. ^{1}H NMR of **6i**

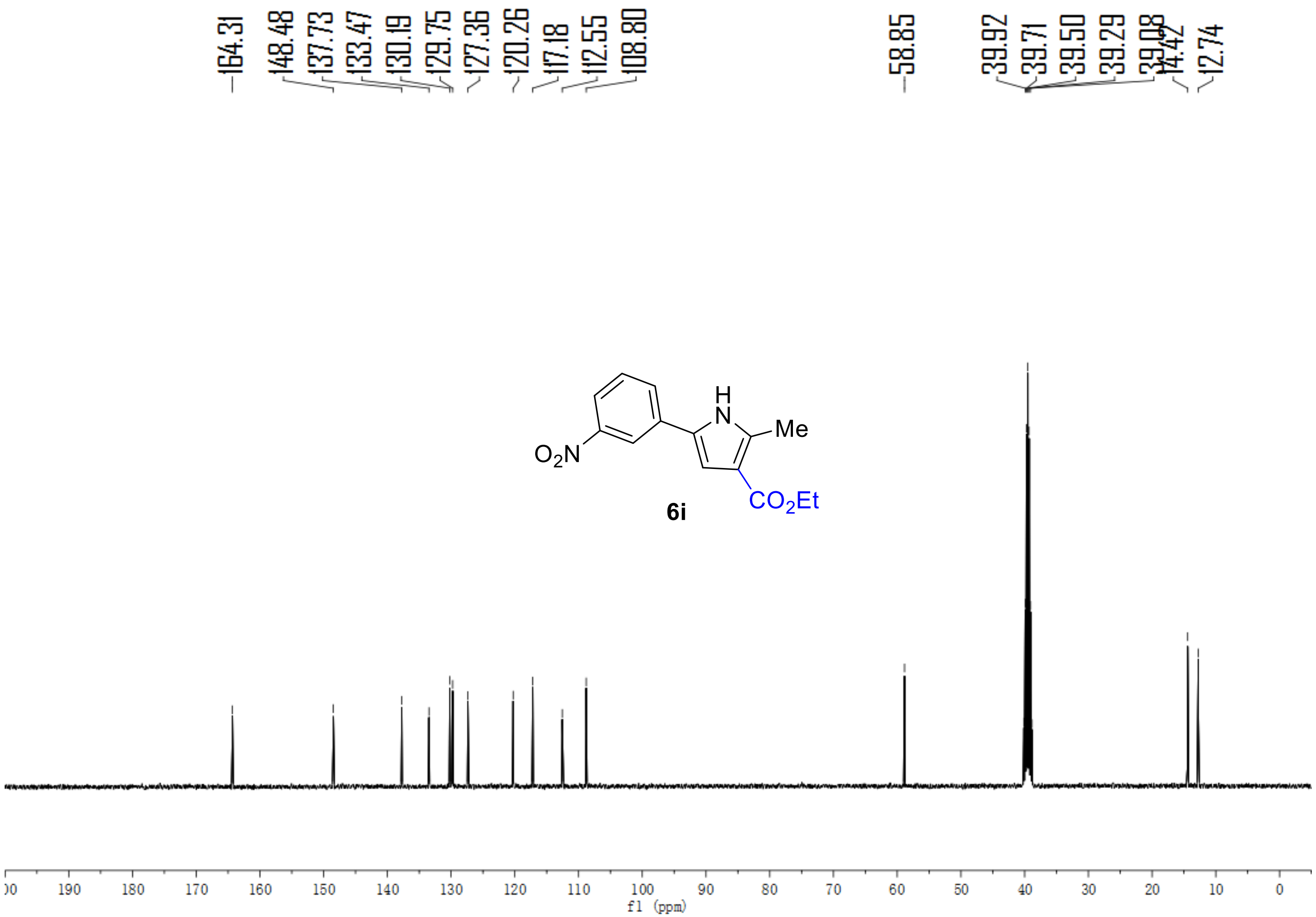

Supplementary Figure 104. ^{13}C NMR of **6i**

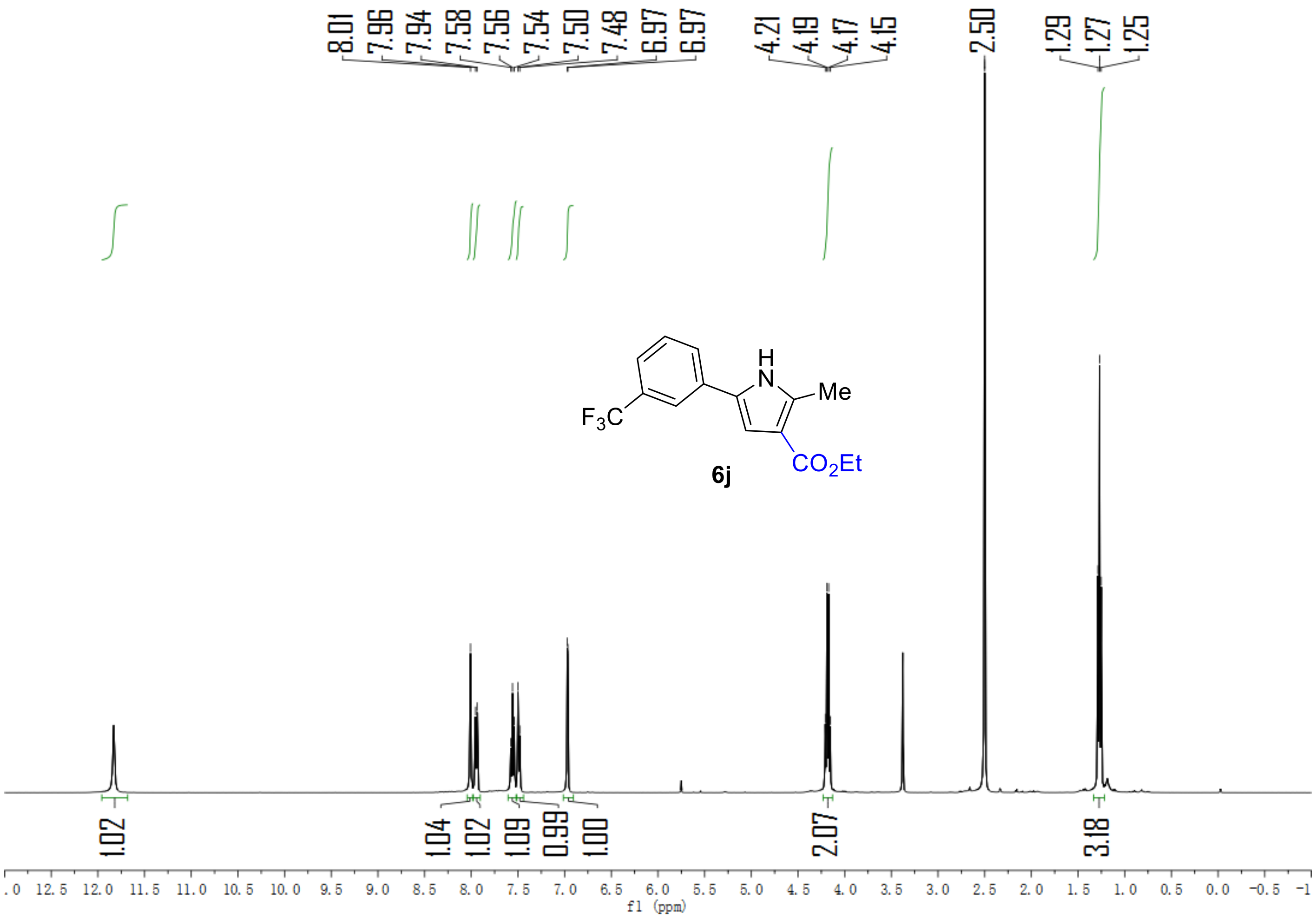

Supplementary Figure 105. ^{1}H NMR of **6j**

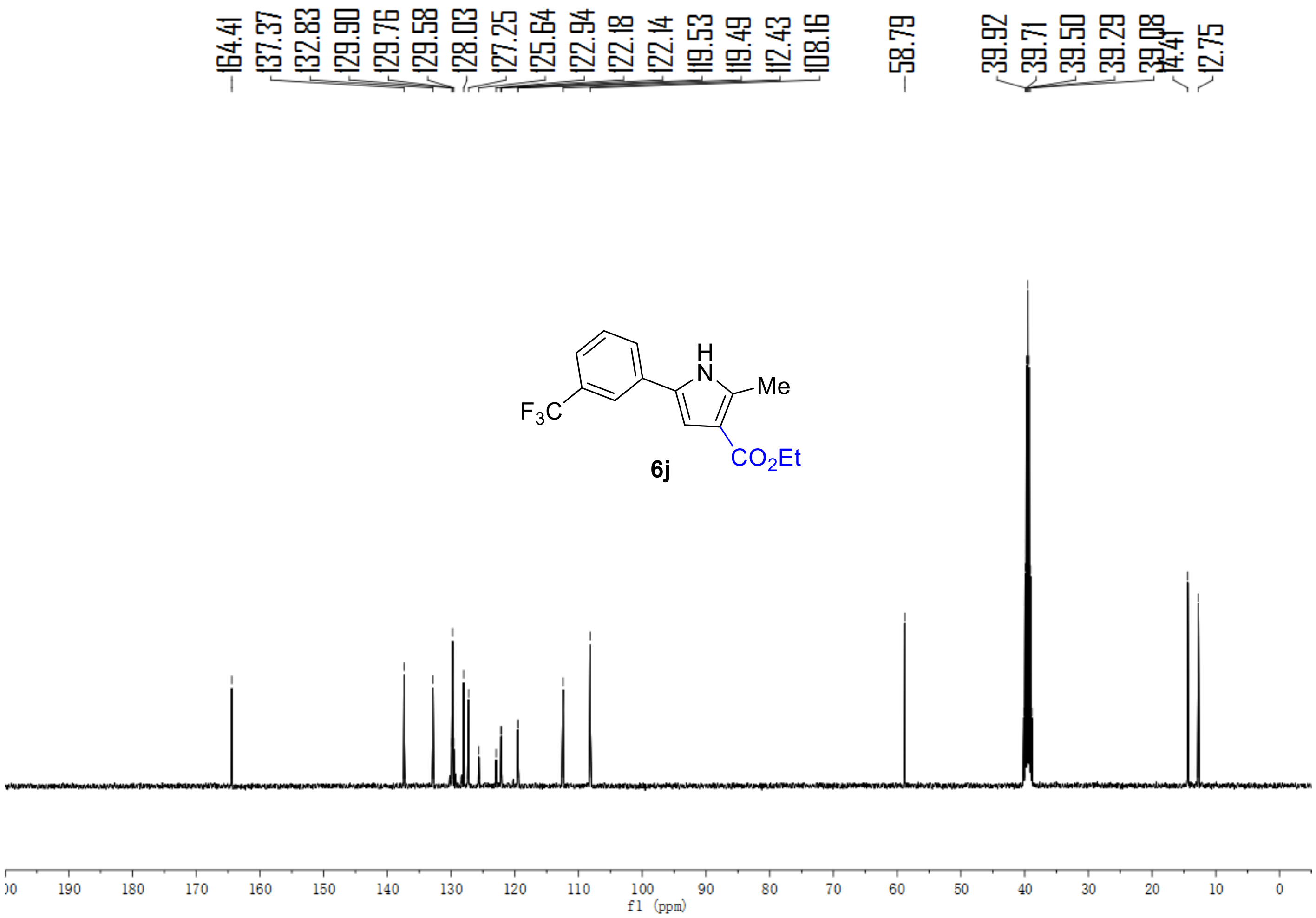

Supplementary Figure 106. ^{13}C NMR of **6j**

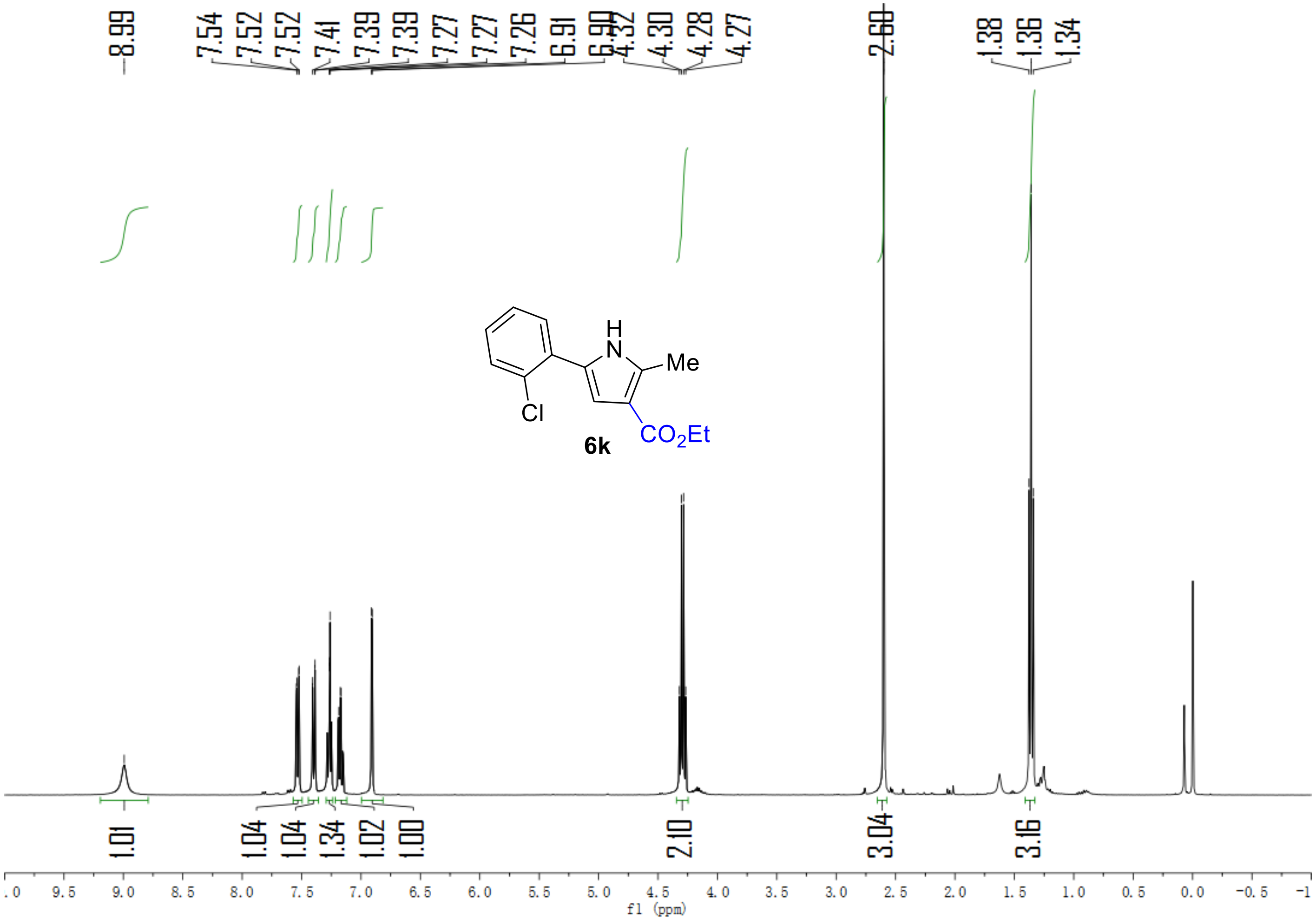

Supplementary Figure 107. ^{1}H NMR of **6k**

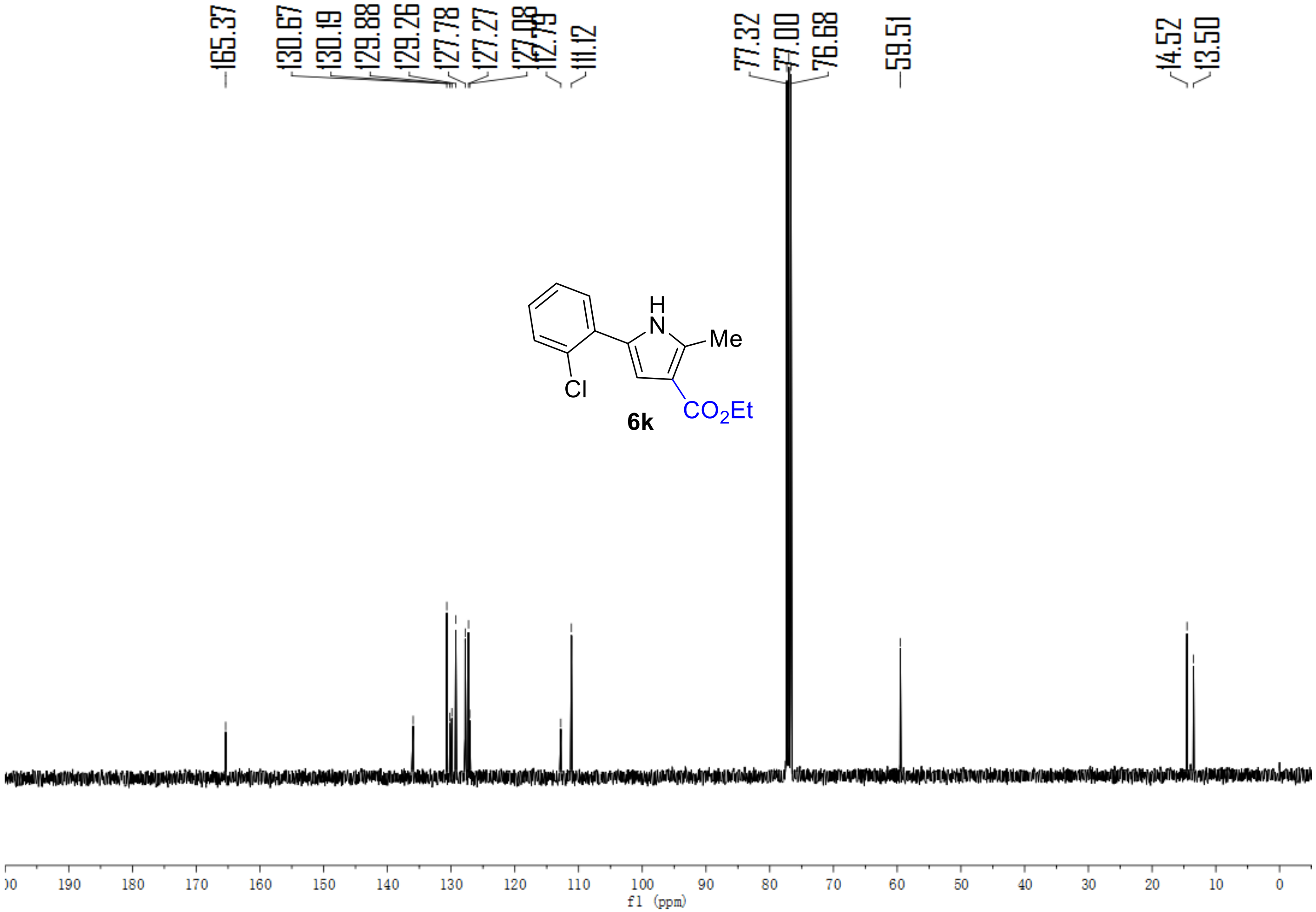

Supplementary Figure 108. ^{13}C NMR of **6k**

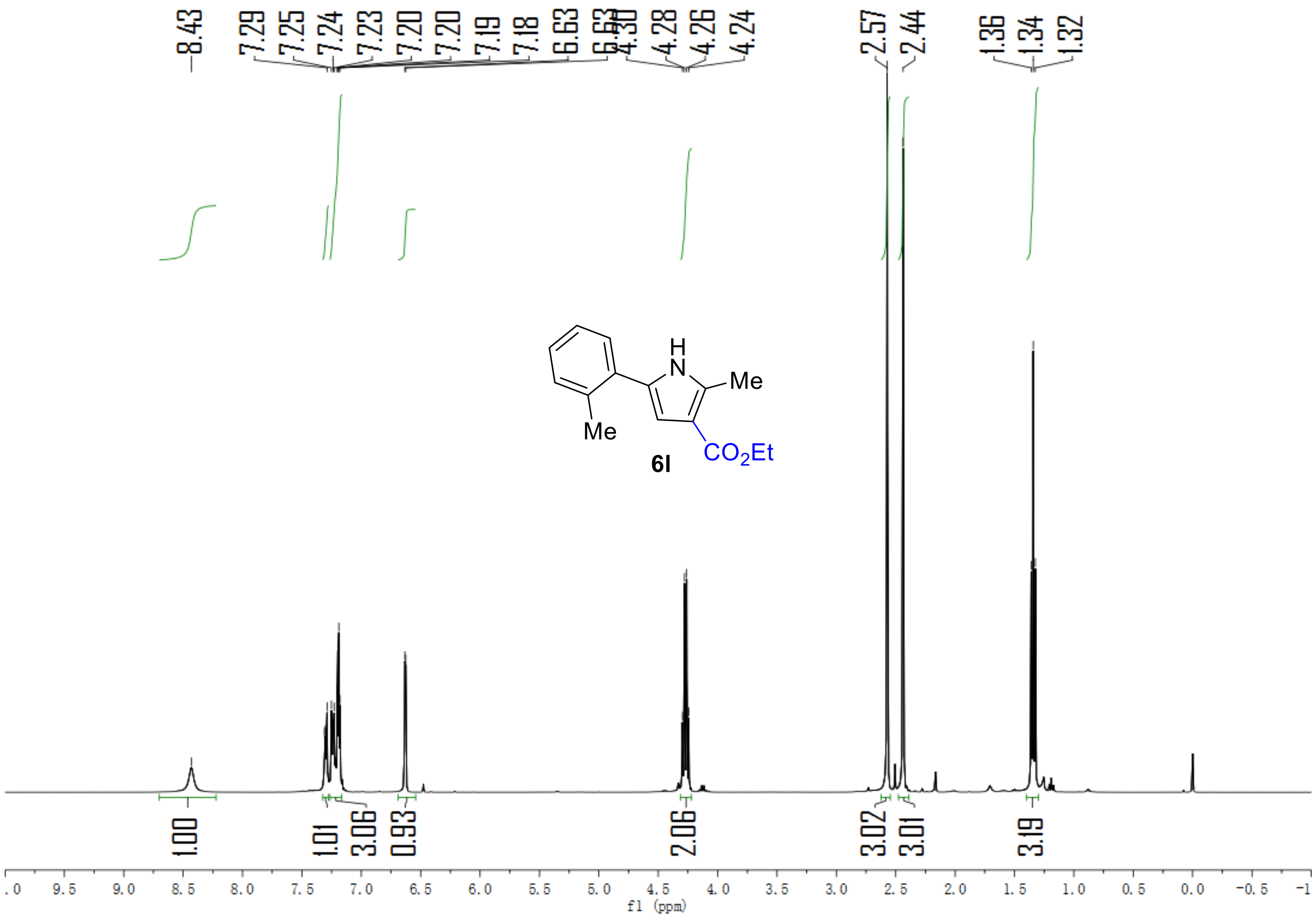

Supplementary Figure 109. ^{1}H NMR of **6l**

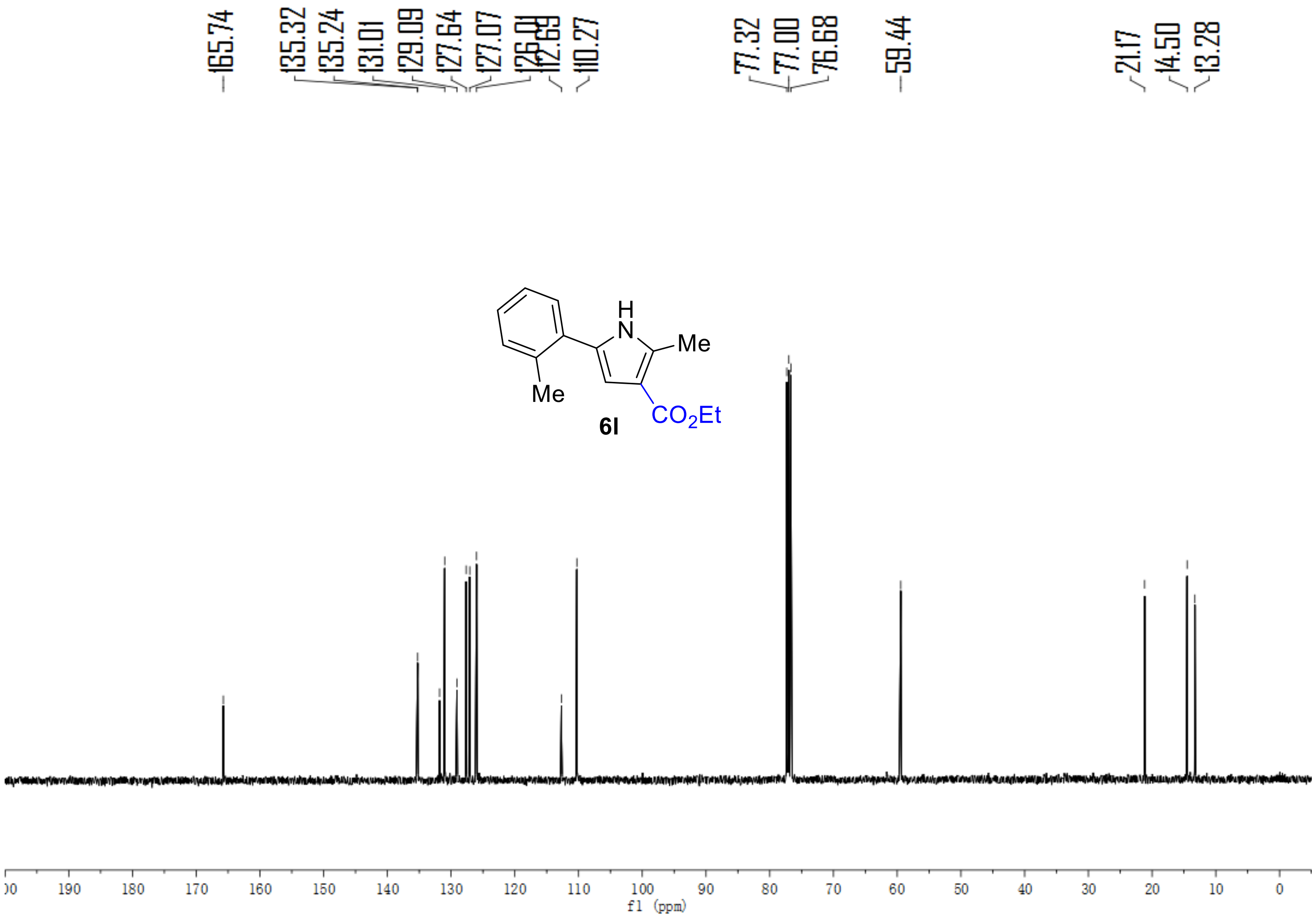

Supplementary Figure 110. ^{13}C NMR of **6l**

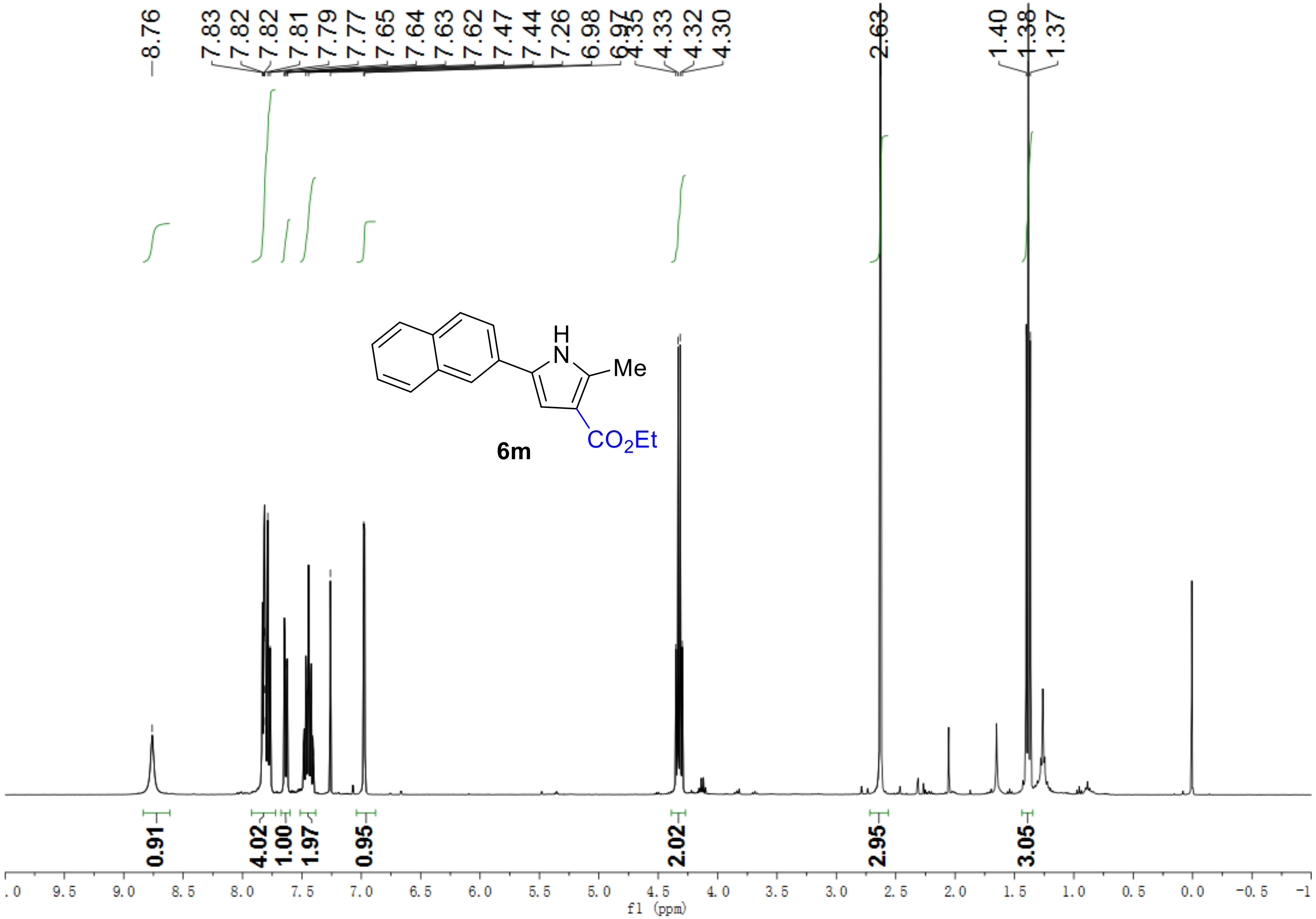

Supplementary Figure 111. ^{1}H NMR of **6m**

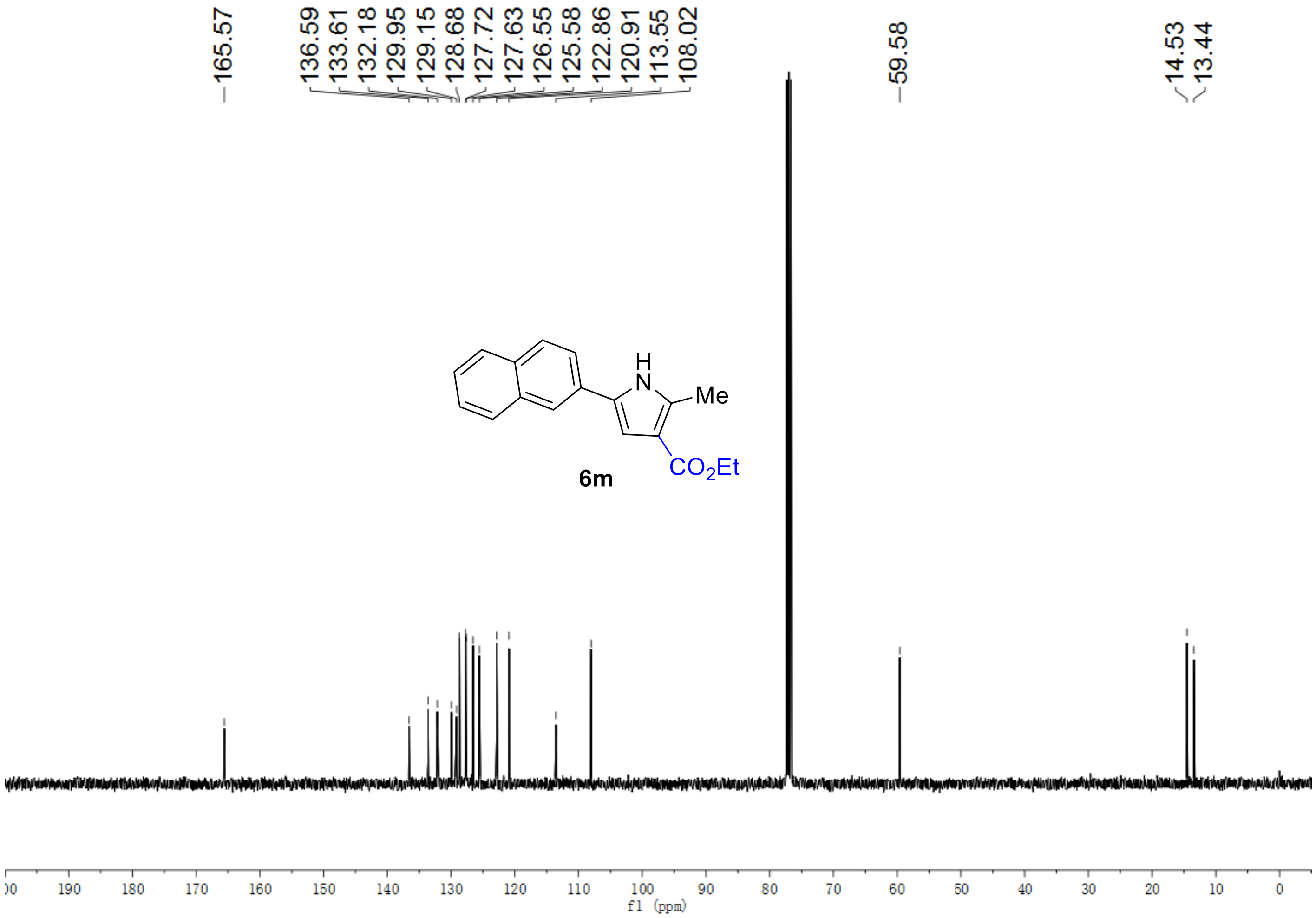

Supplementary Figure 112. ^{13}C NMR of **6m**

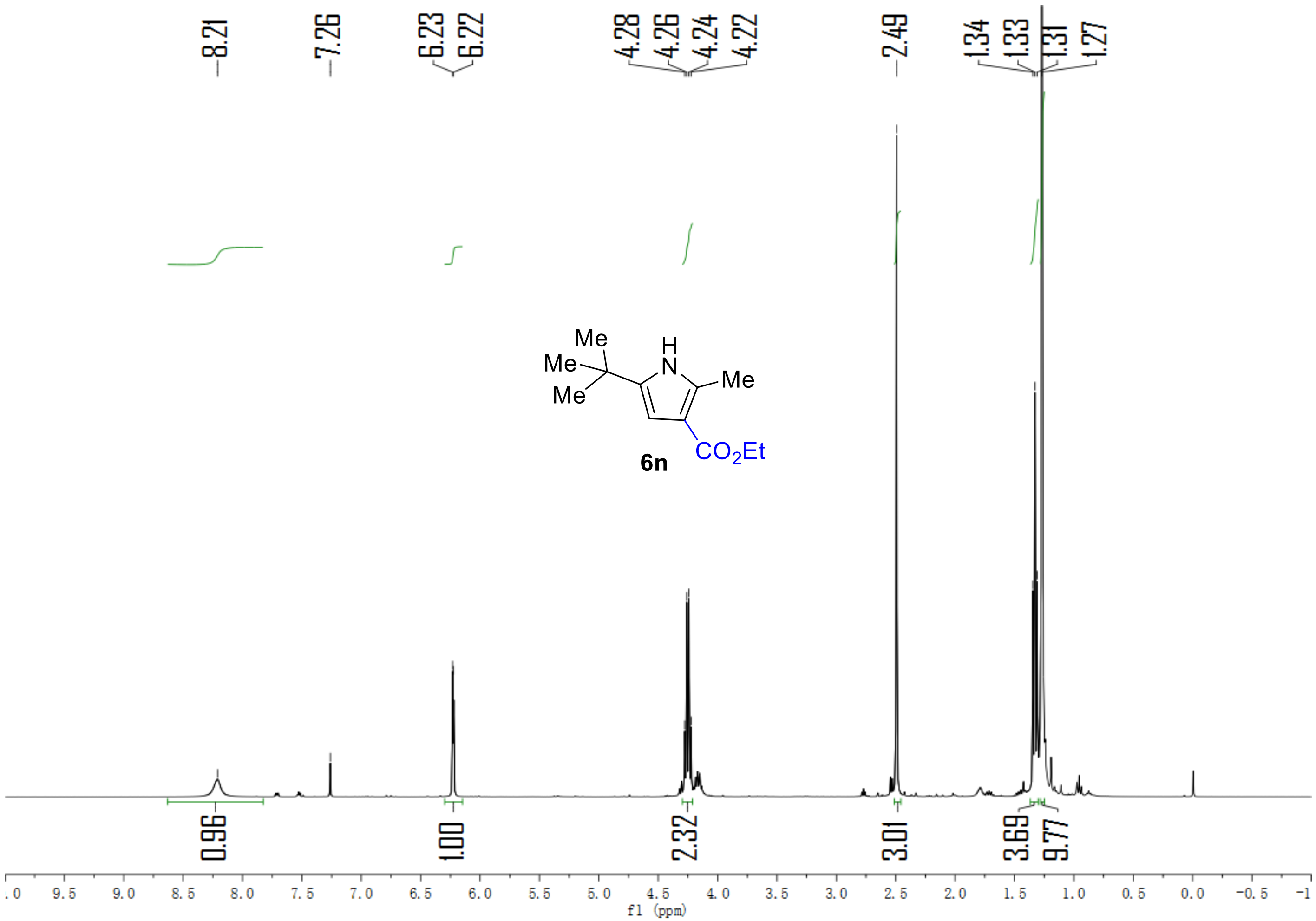

Supplementary Figure 113. ^{1}H NMR of **6n**

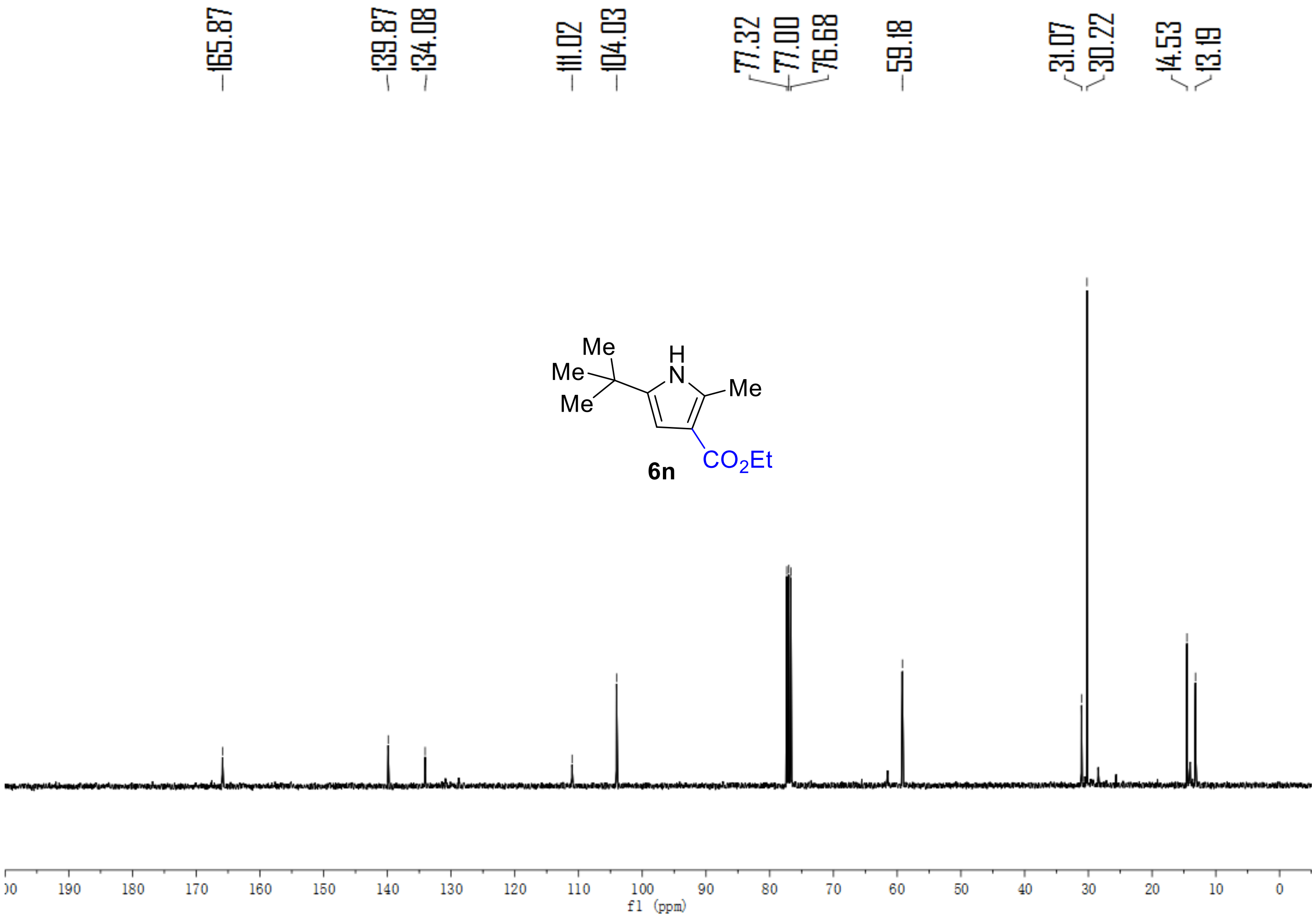

Supplementary Figure 114. ^{13}C NMR of **6n**

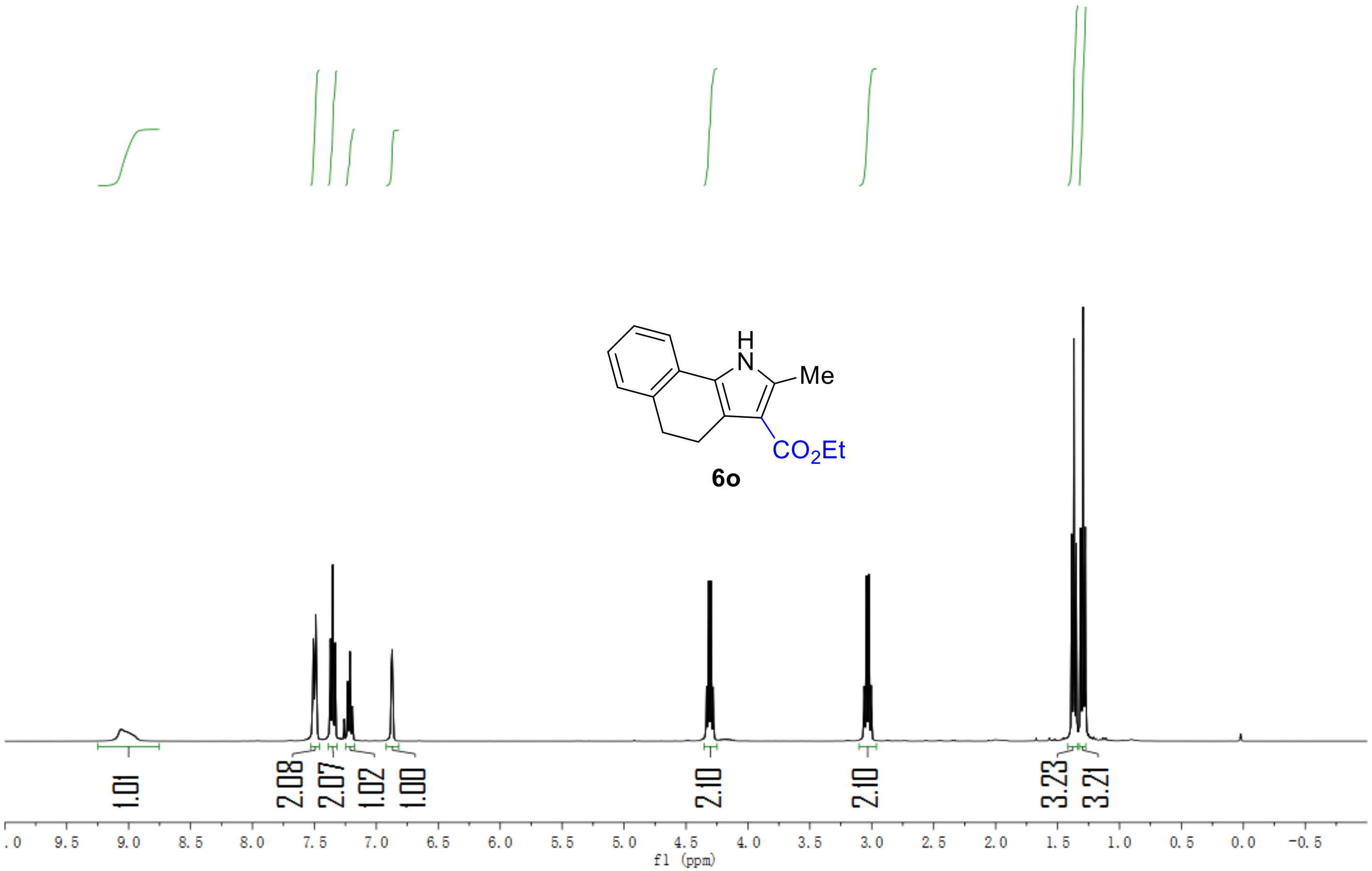

Supplementary Figure 115. ^{1}H NMR of **6o**

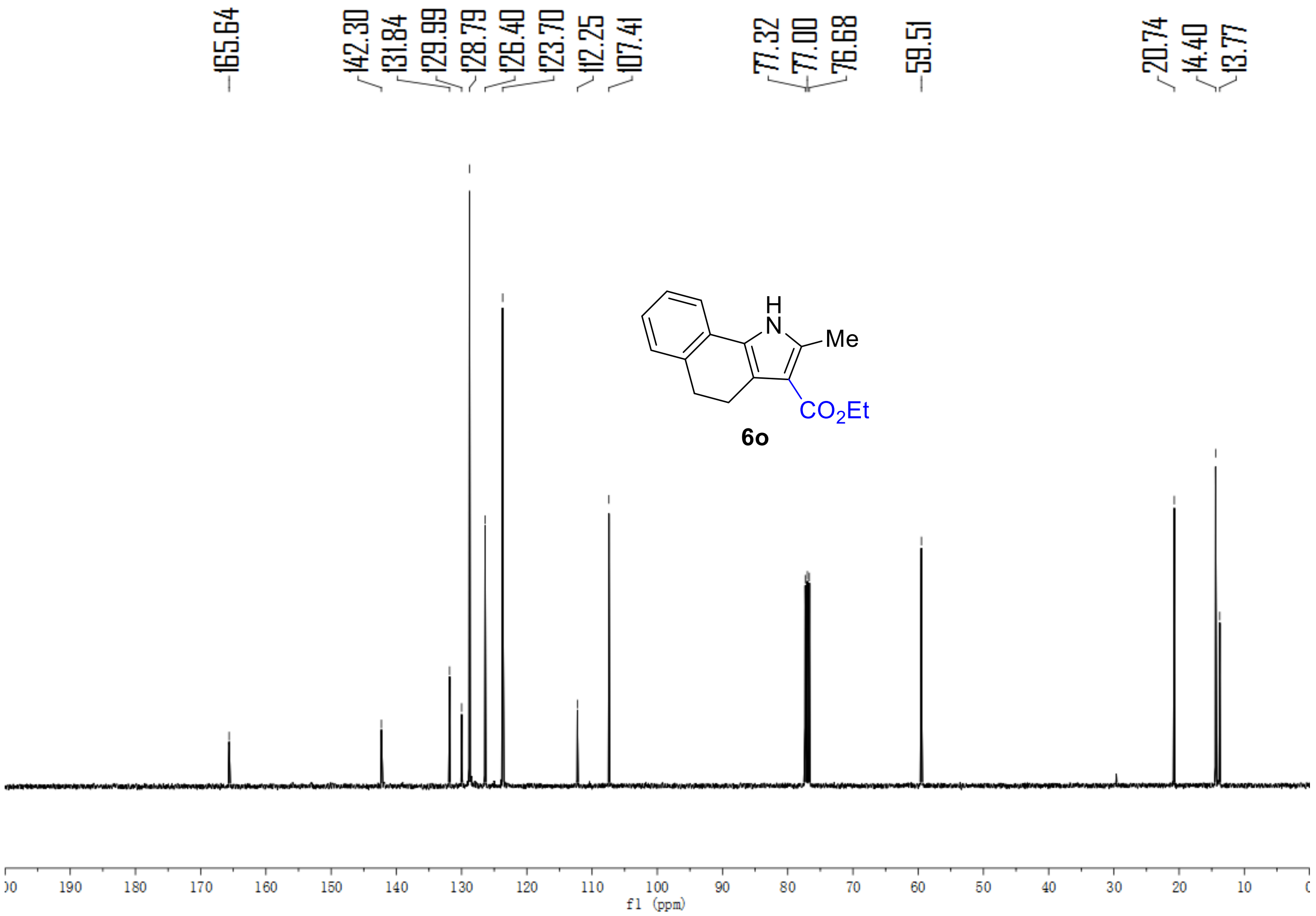

Supplementary Figure 116. ^{13}C NMR of **6o**

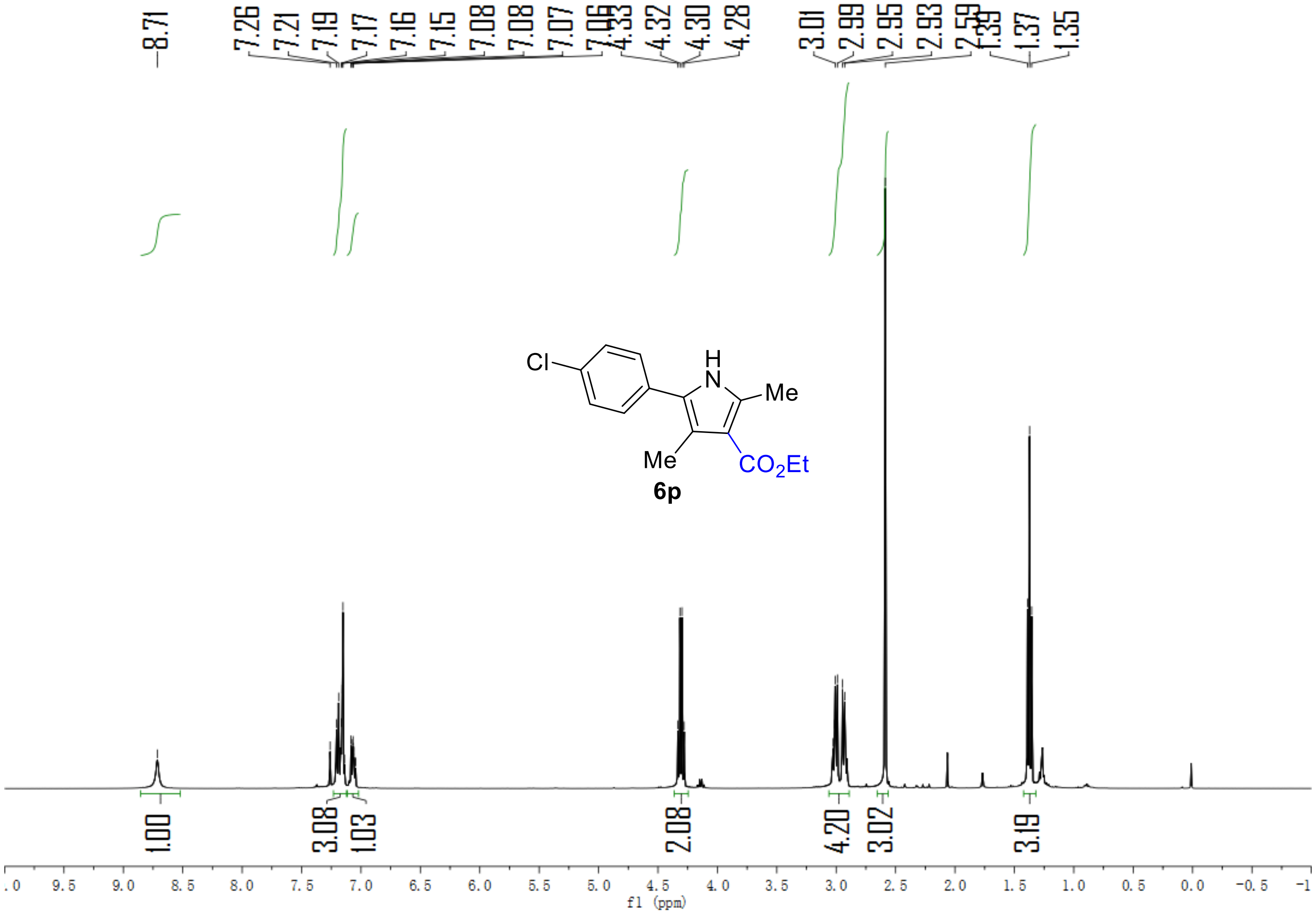

Supplementary Figure 117. ^{1}H NMR of **6p**

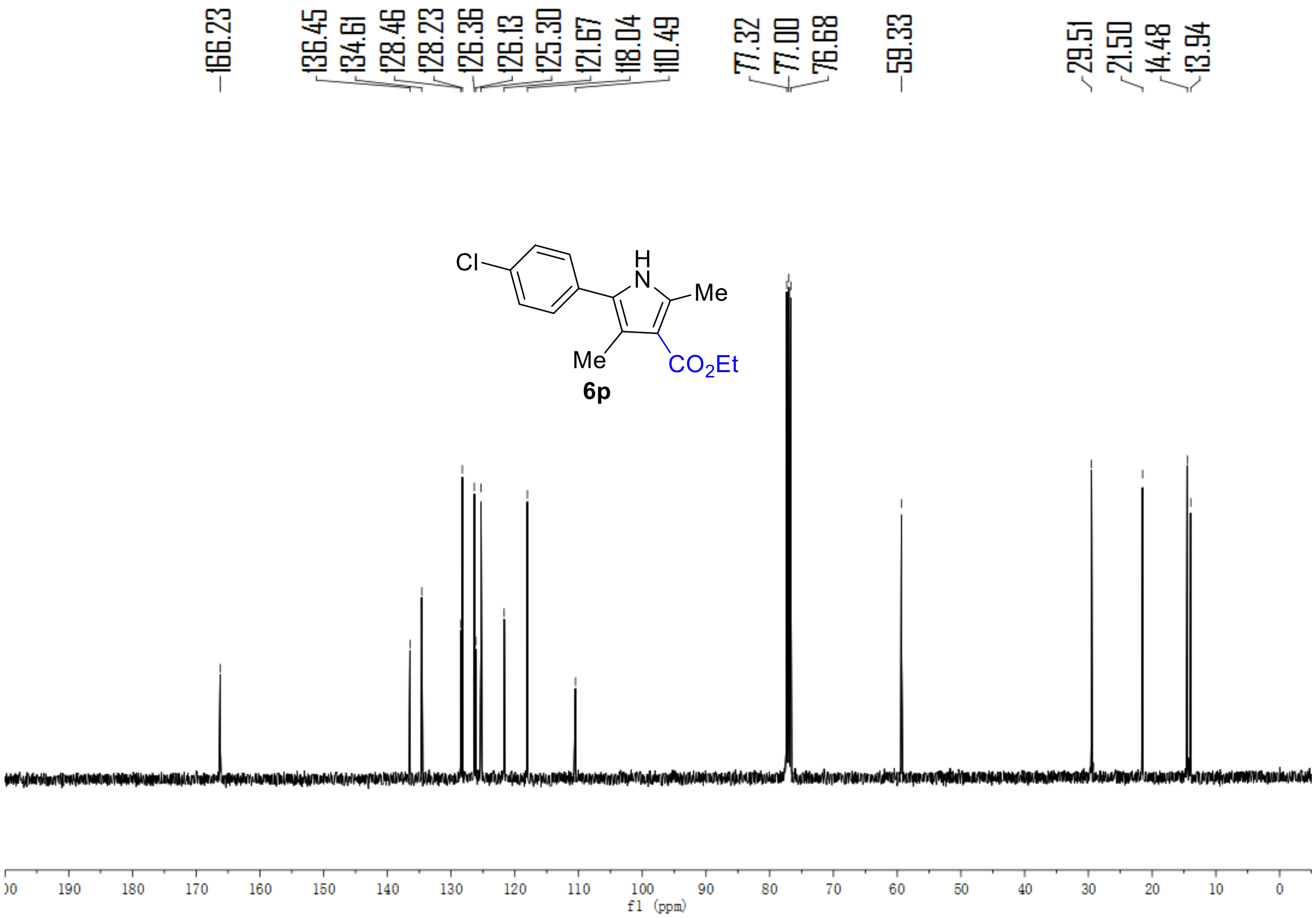

Supplementary Figure 118. ^{13}C NMR of **6p**

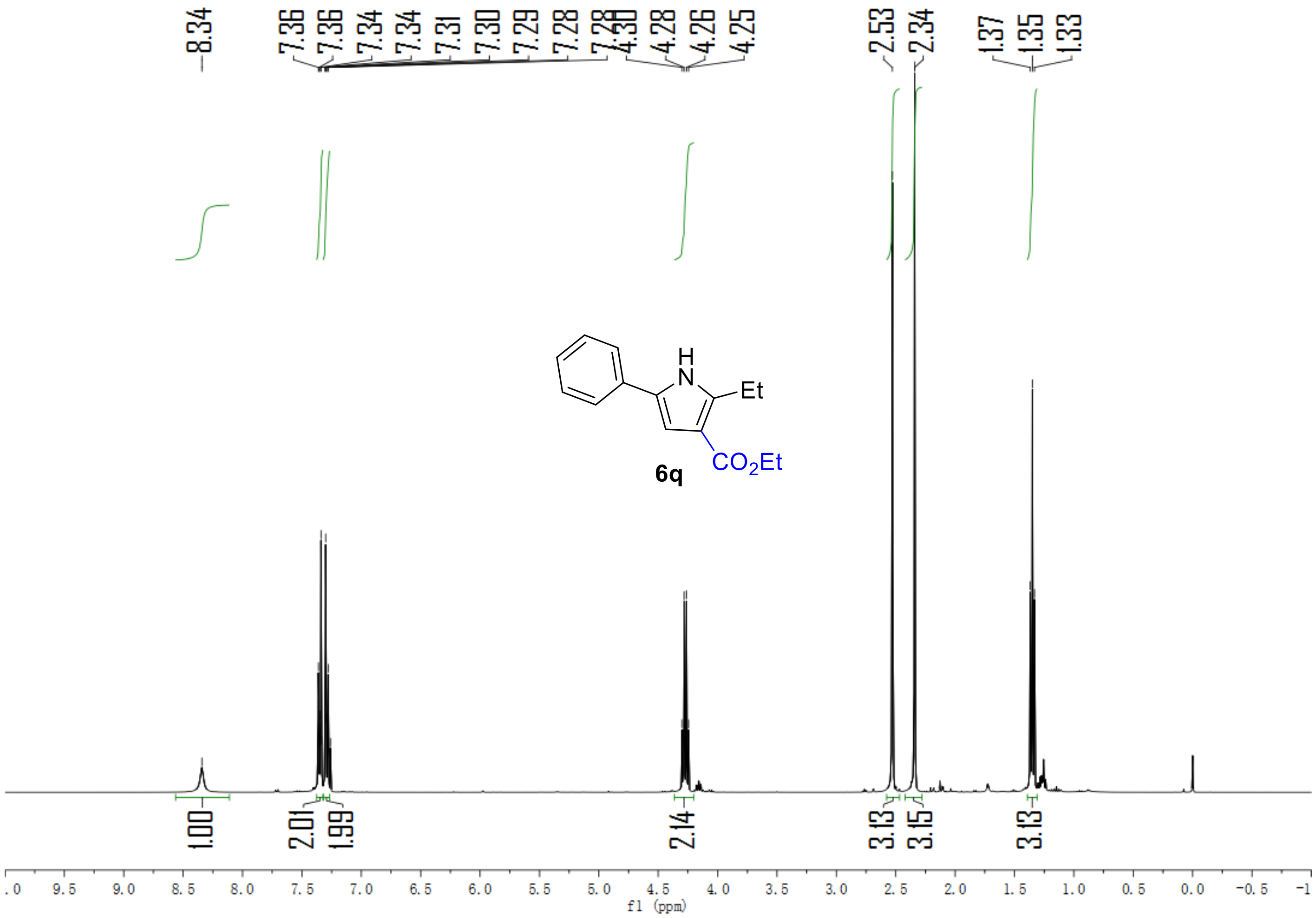

Supplementary Figure 119. ^{1}H NMR of **6q**

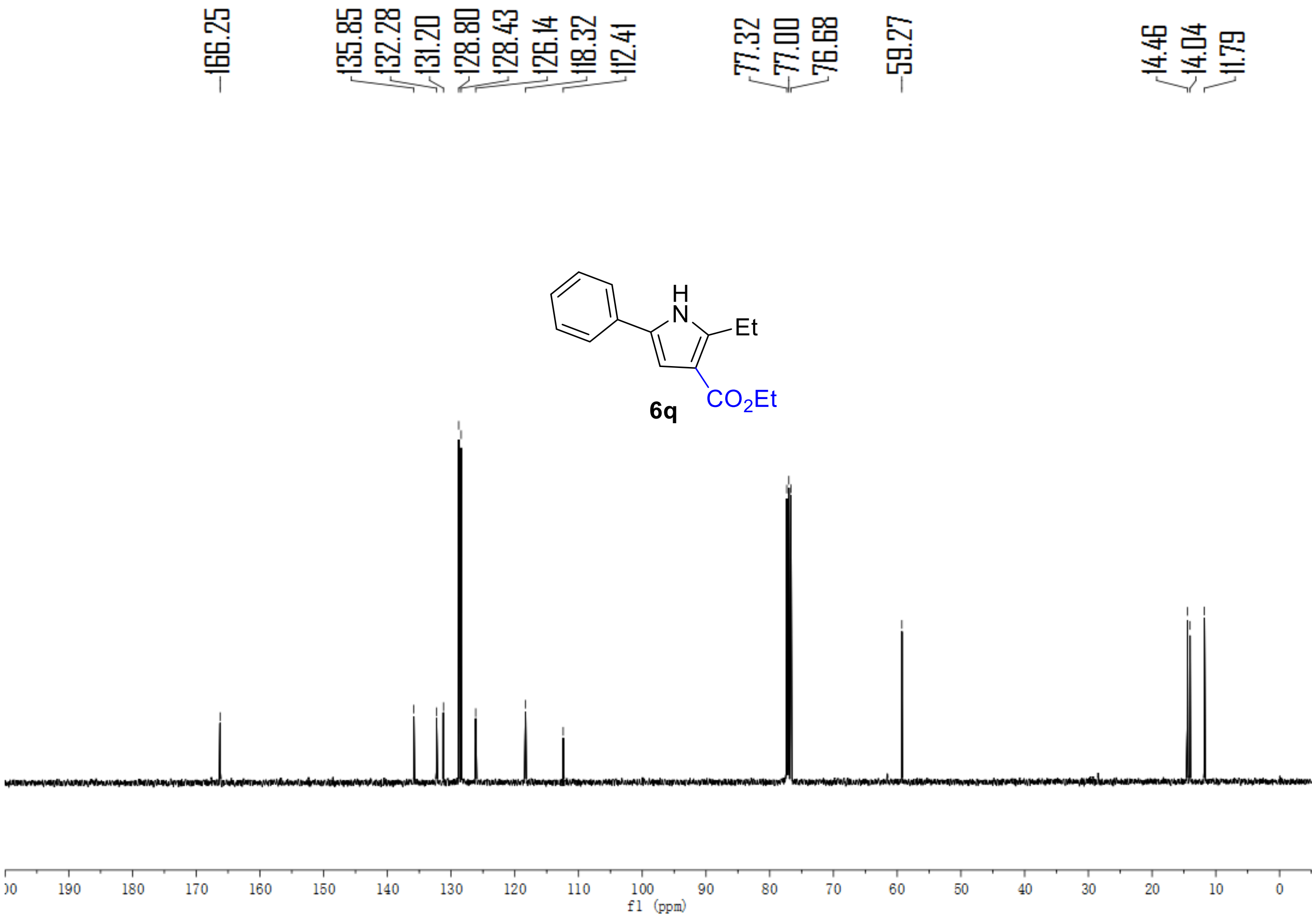

Supplementary Figure 120. ^{13}C NMR of **6q**

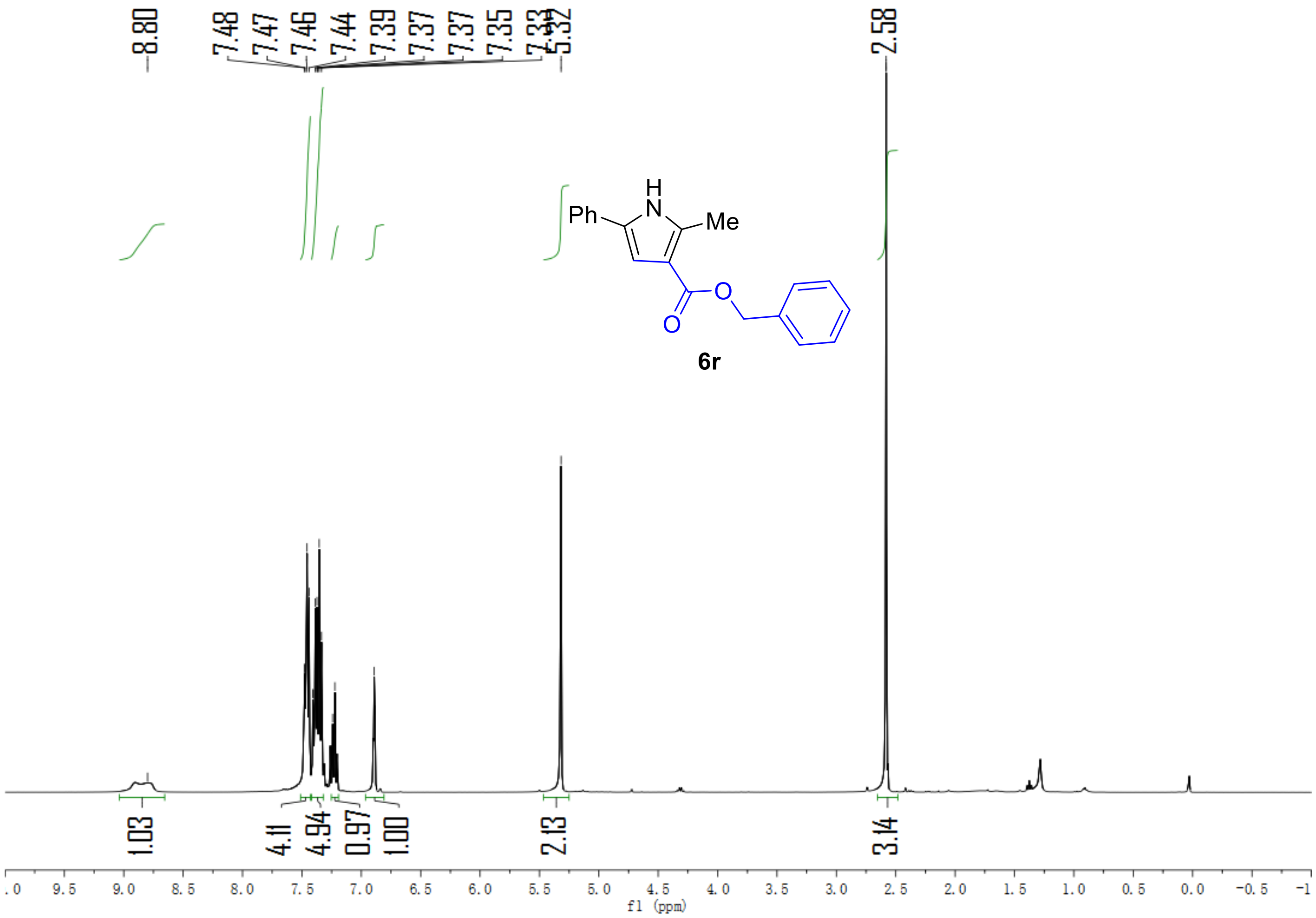

Supplementary Figure 121. ^{1}H NMR of **6r**

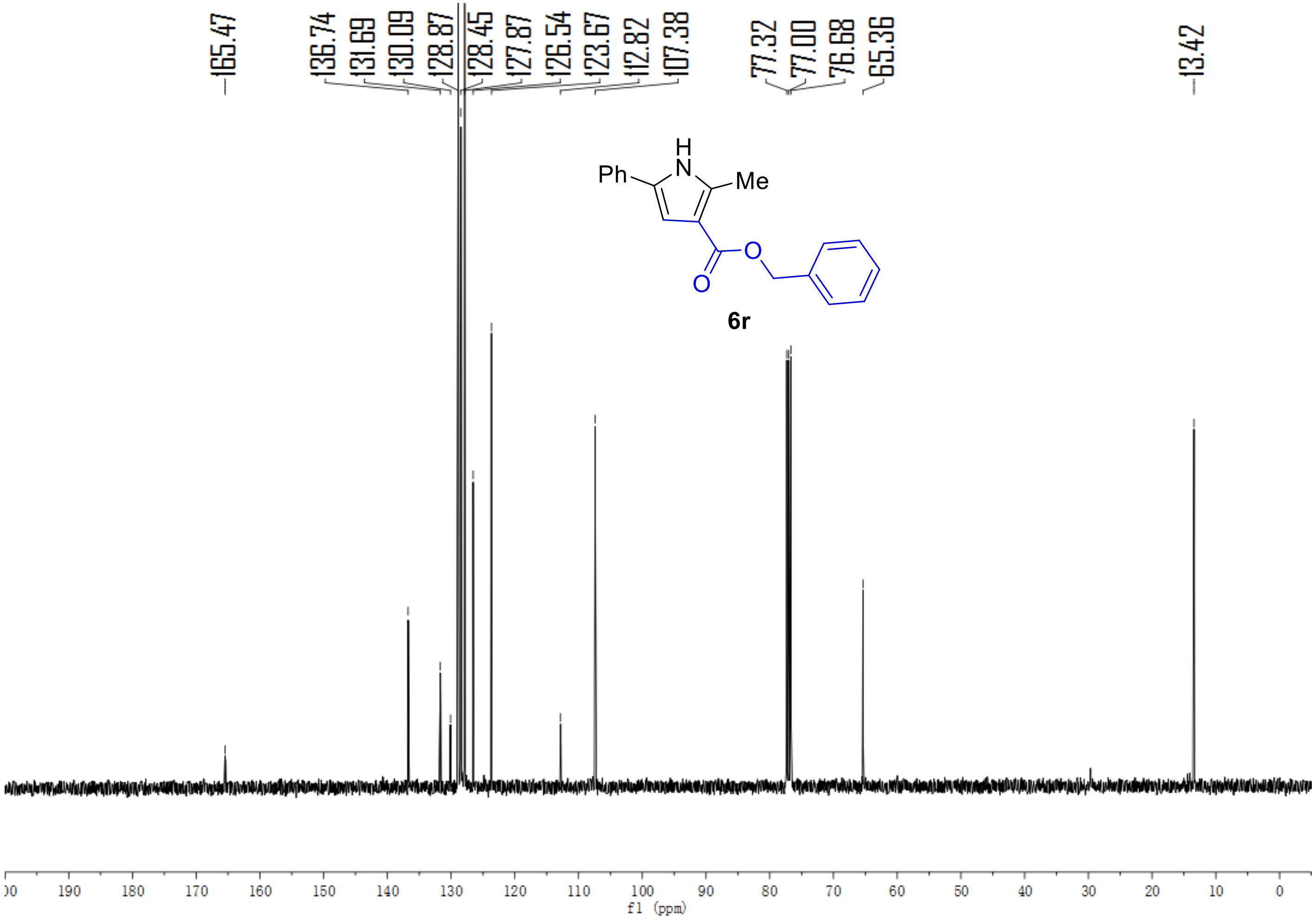

Supplementary Figure 122. ^{13}C NMR of **6r**

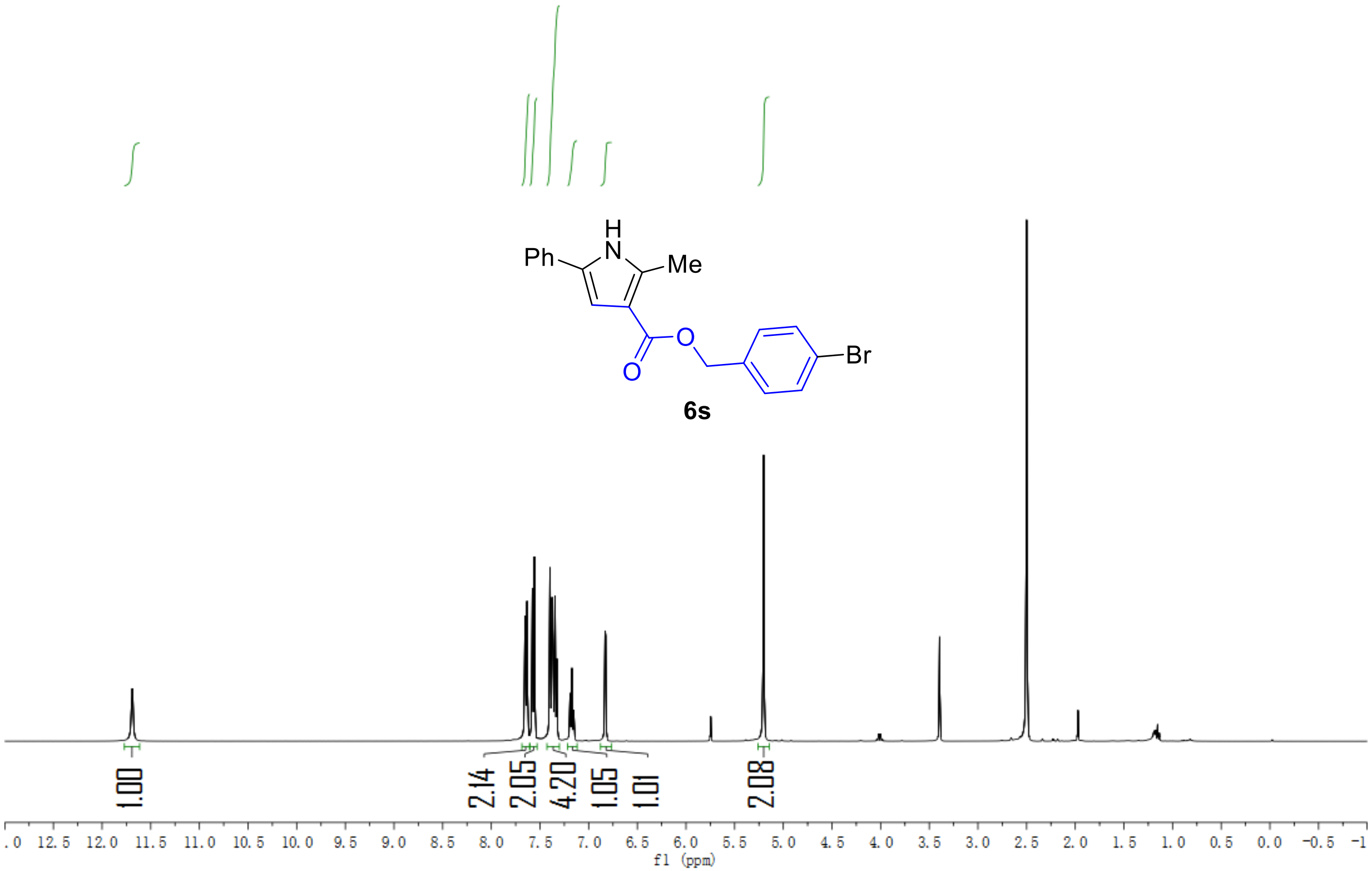

Supplementary Figure 123. ^{1}H NMR of **6s**

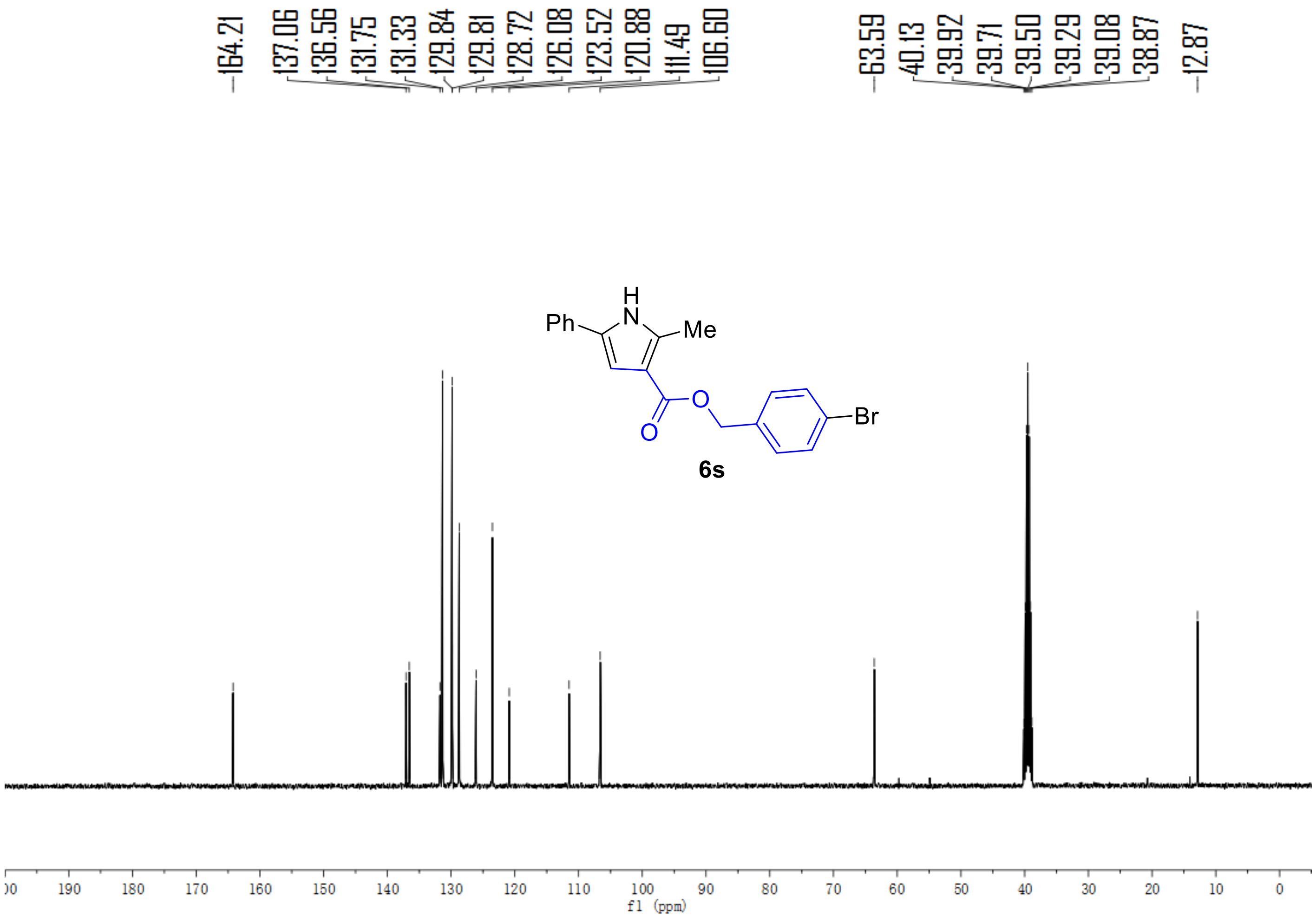

Supplementary Figure 124. ^{13}C NMR of **6s**

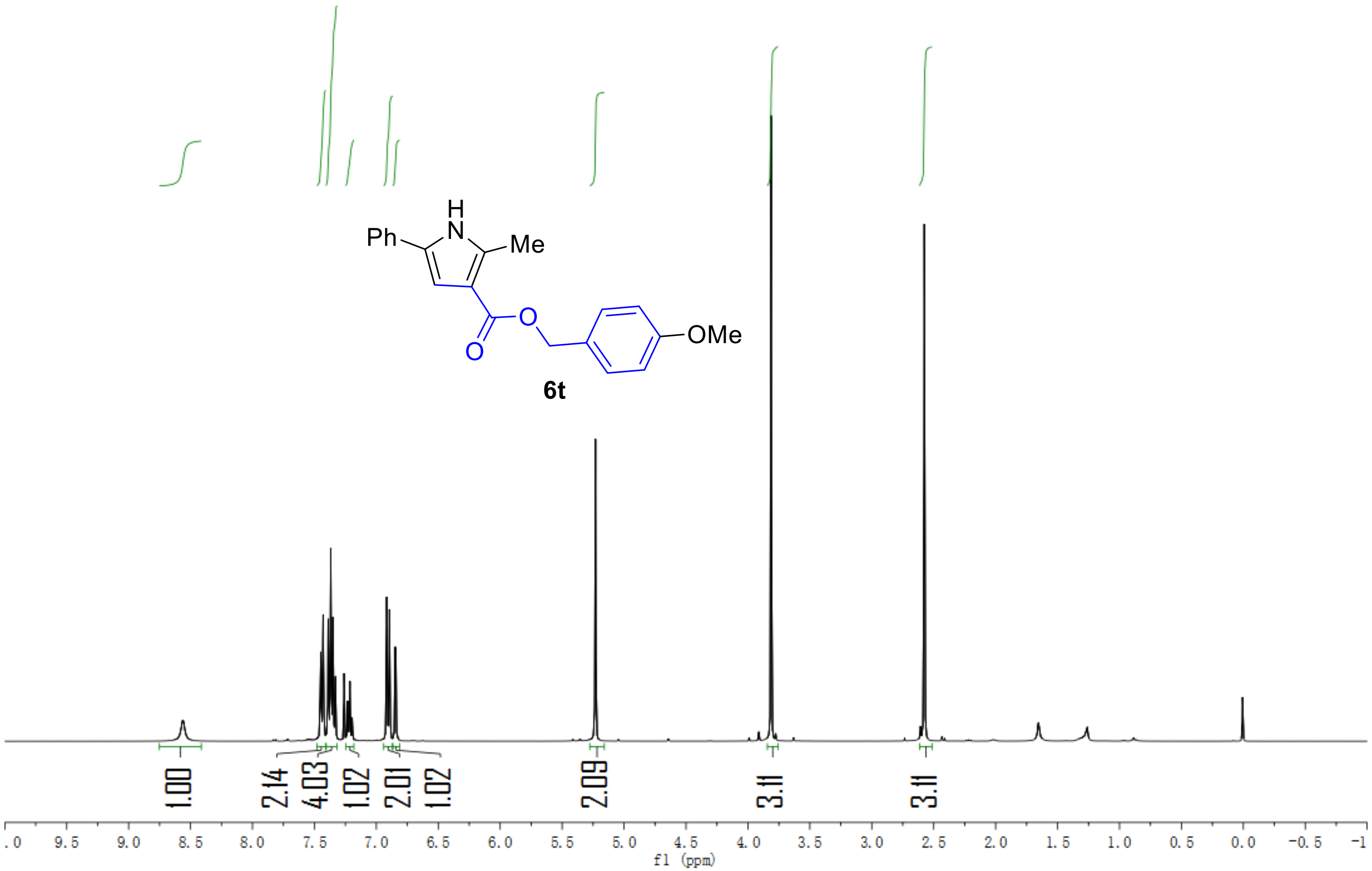

Supplementary Figure 125. ^{1}H NMR of **6t**

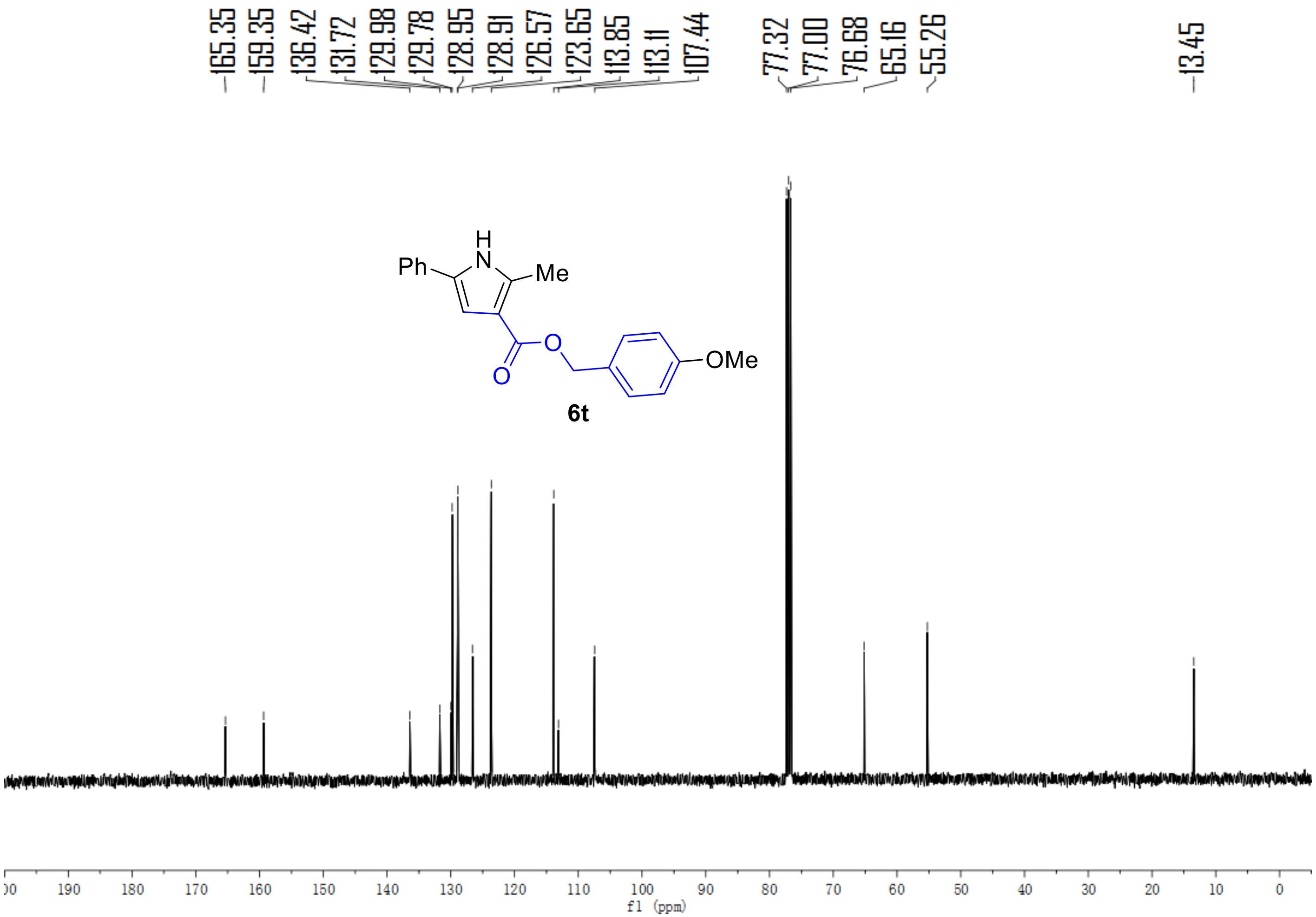

Supplementary Figure 126. ^{13}C NMR of **6t**

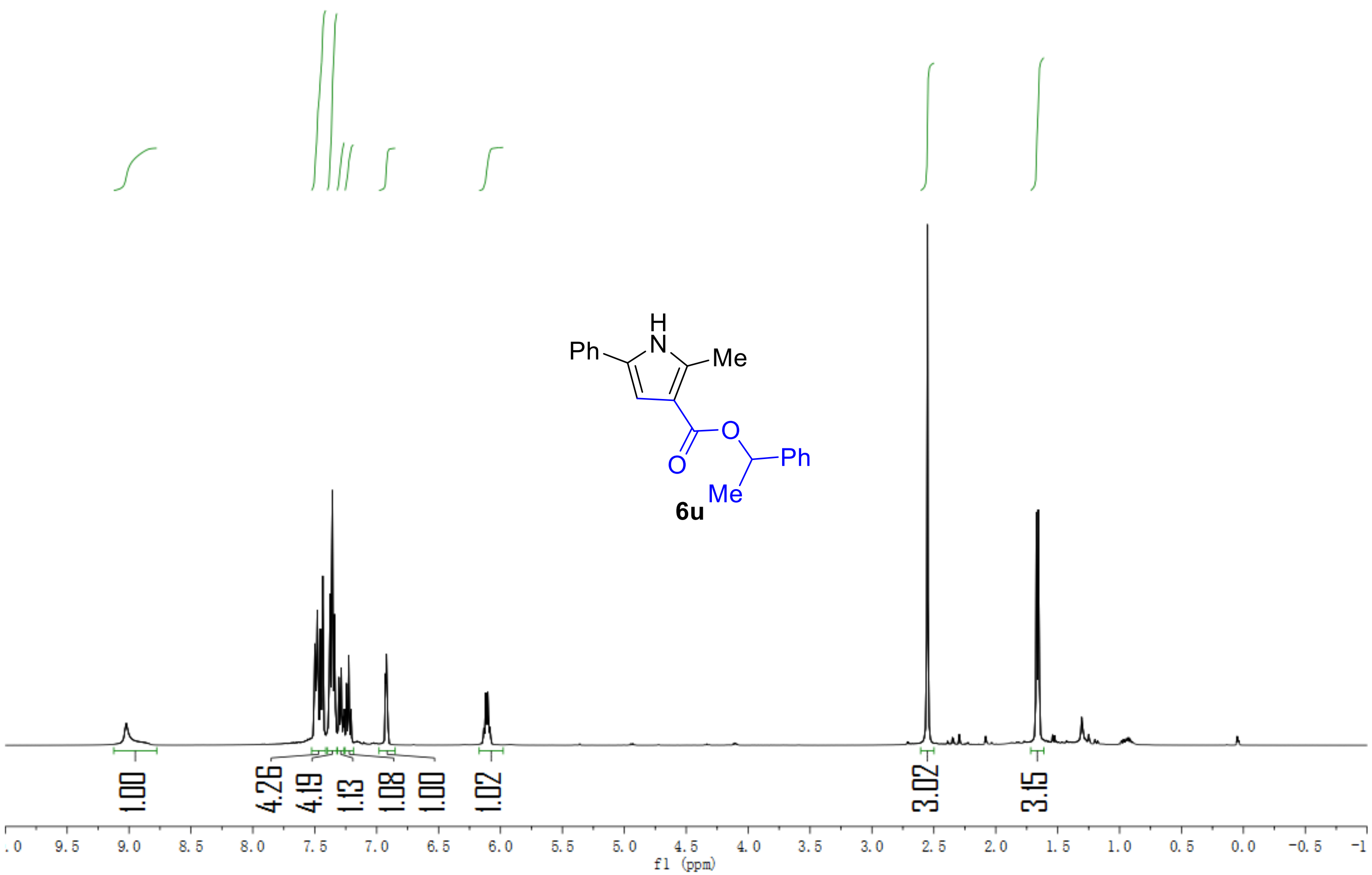

Supplementary Figure 127. ^{1}H NMR of **6u**

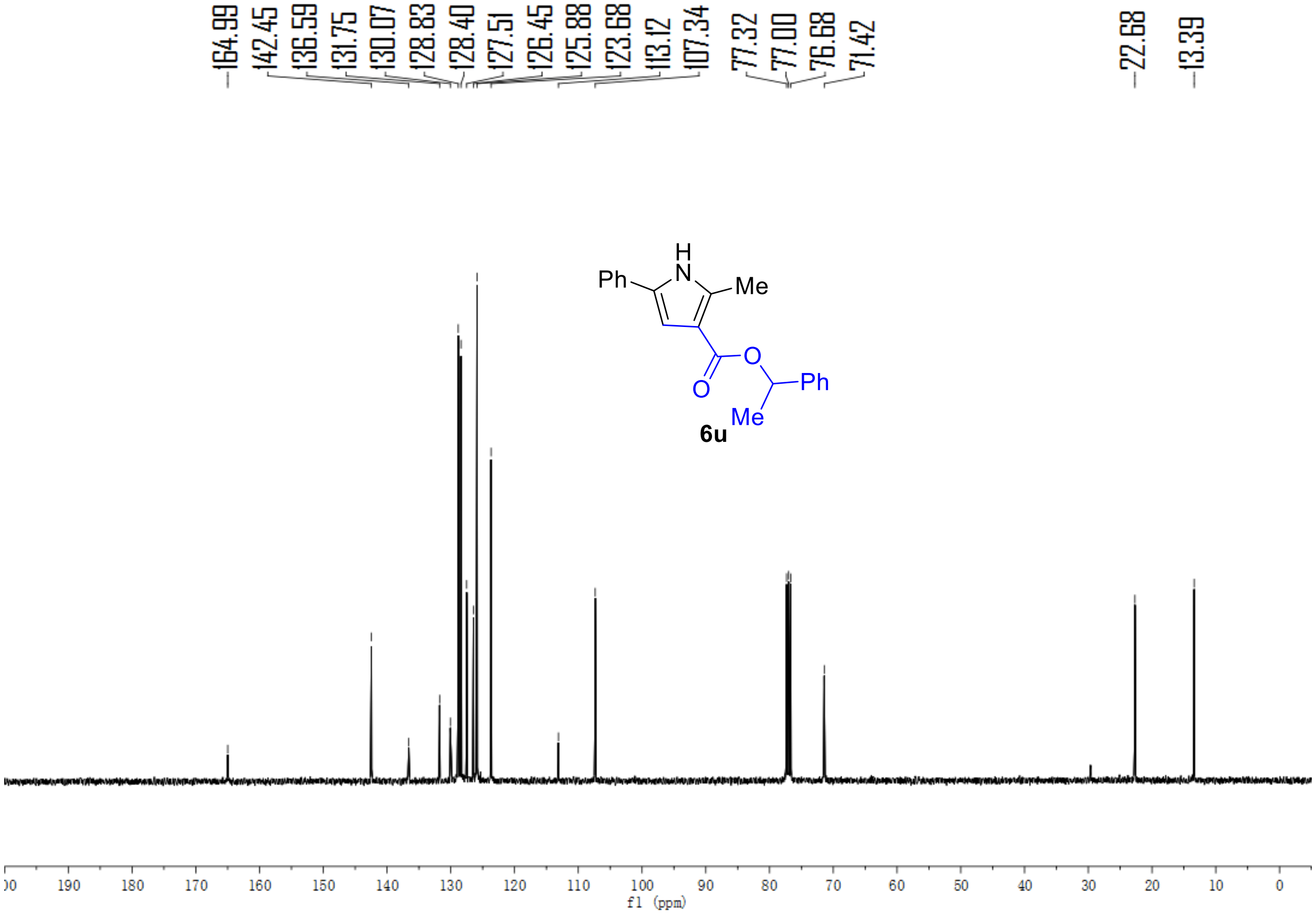

Supplementary Figure 128. ^{13}C NMR of **6u**

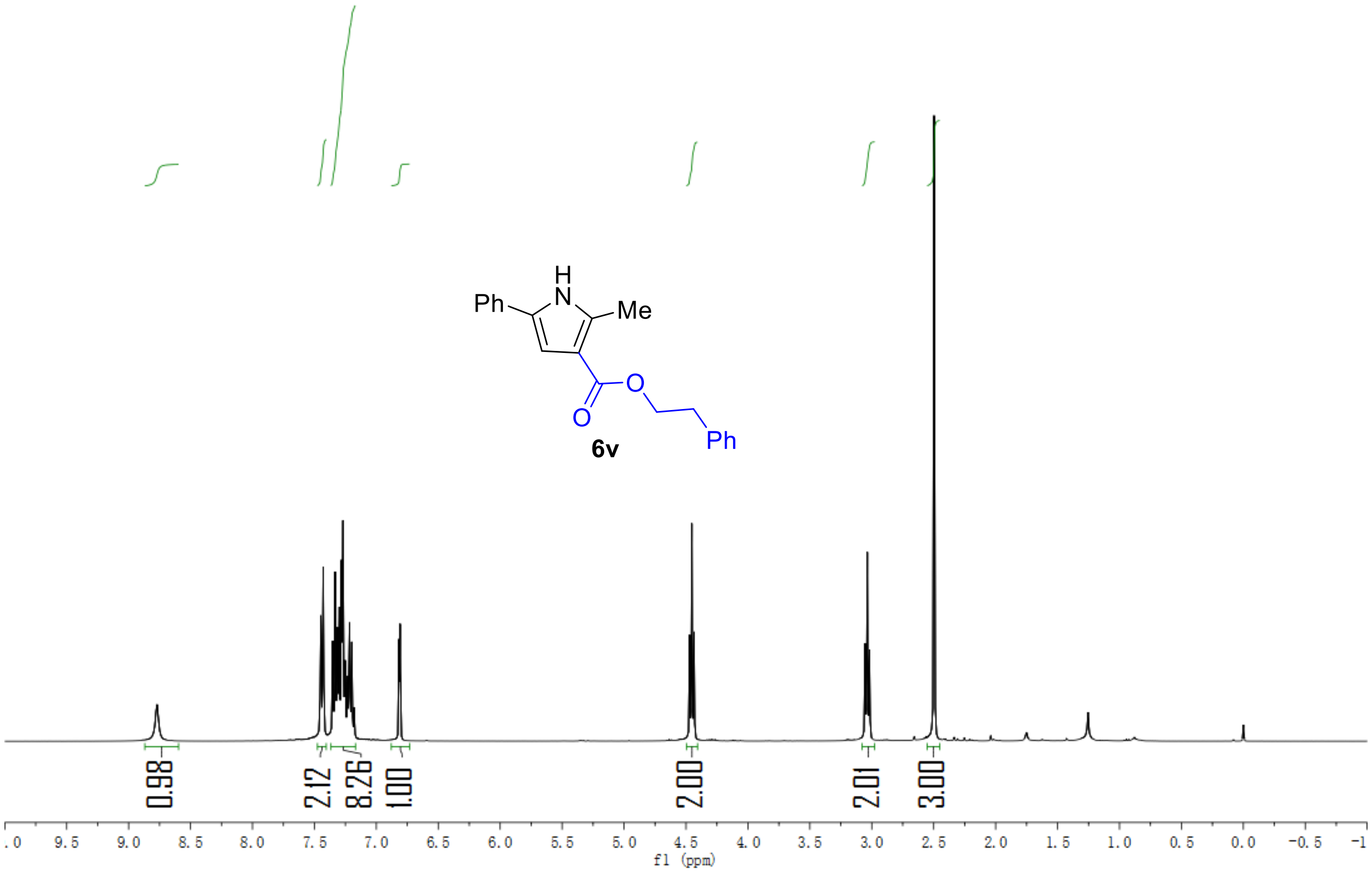

Supplementary Figure 129. ^{1}H NMR of **6v**

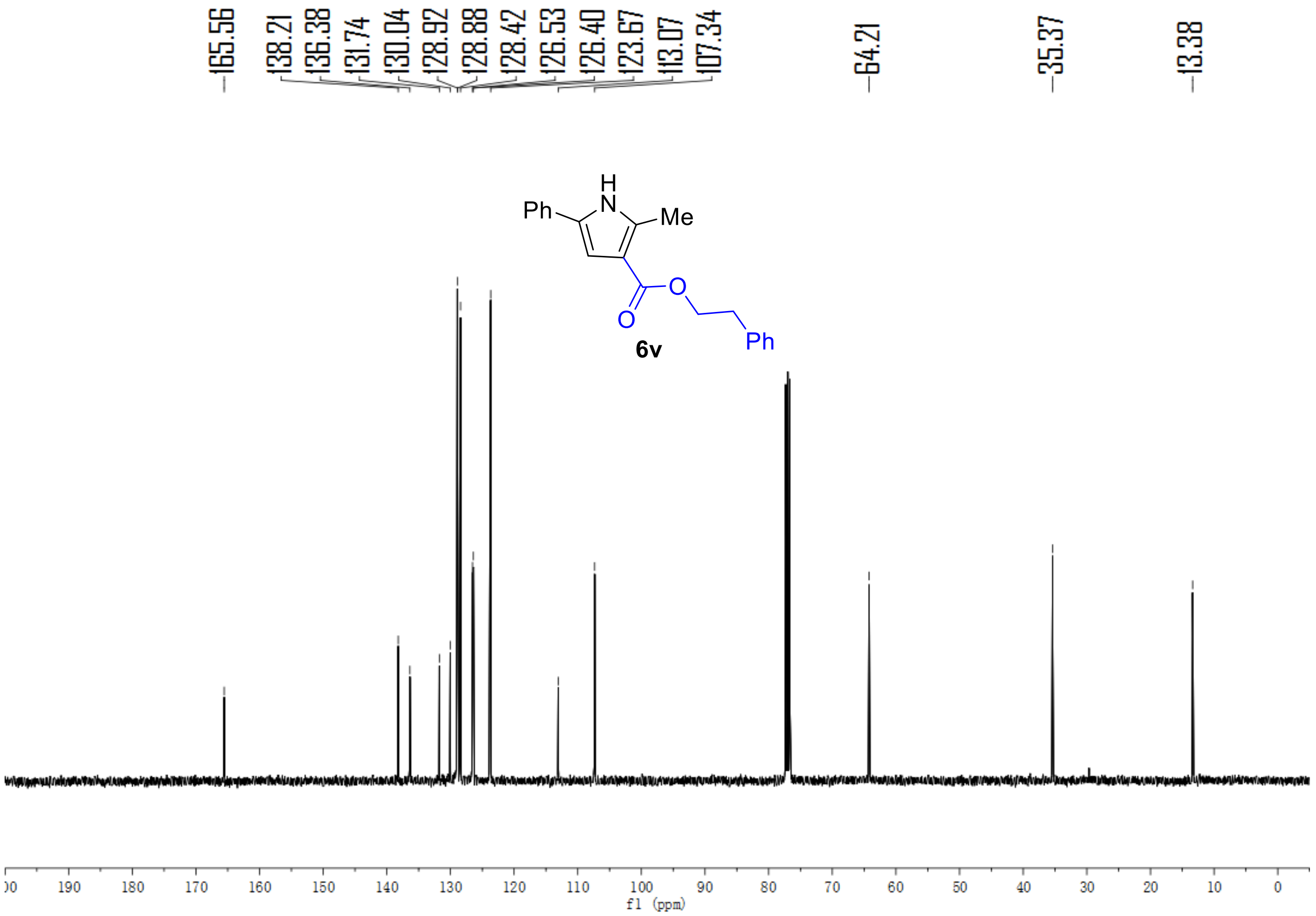

Supplementary Figure 130. ^{13}C NMR of **6v**

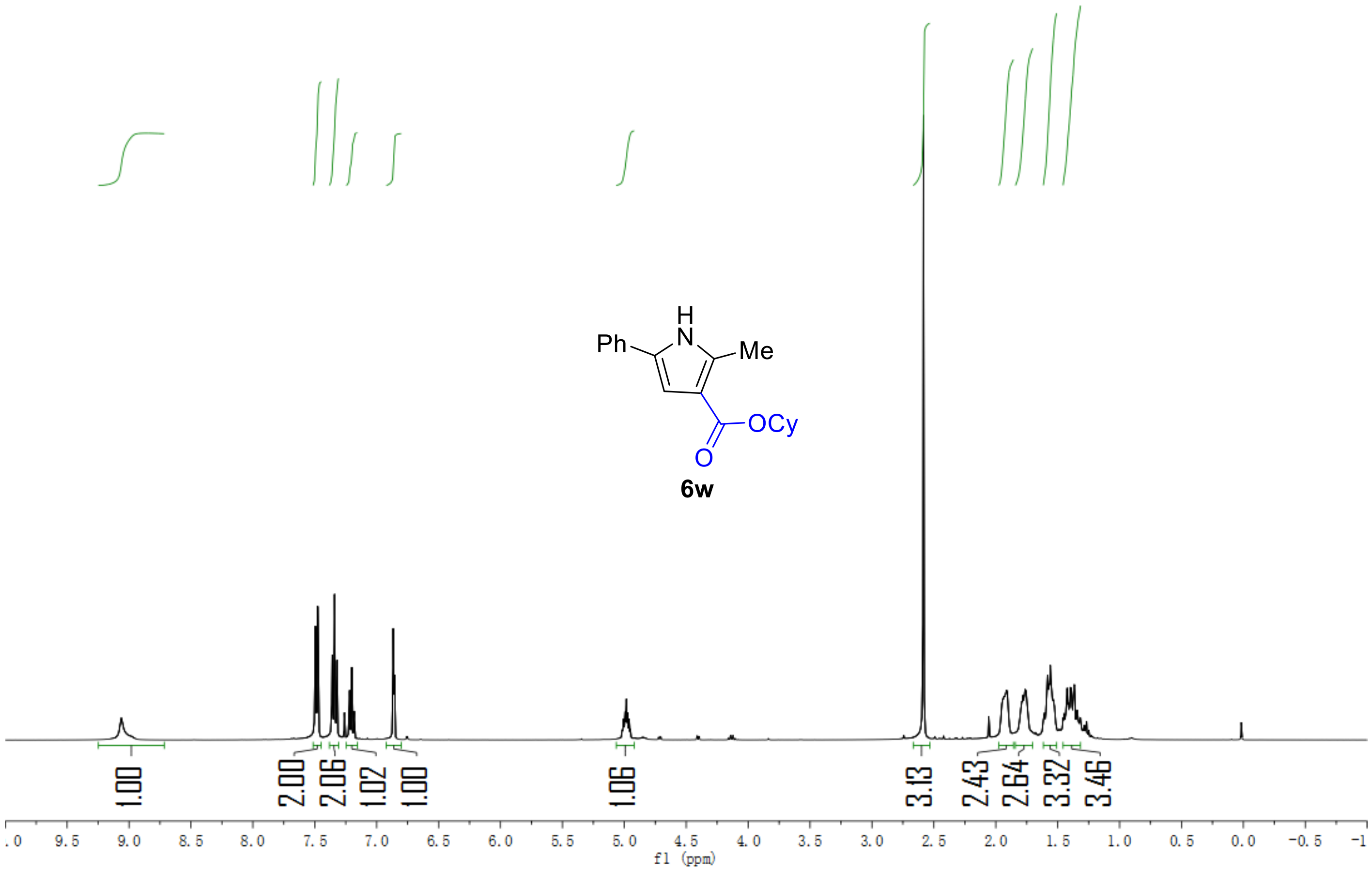

Supplementary Figure 131. ¹H NMR of **6w**

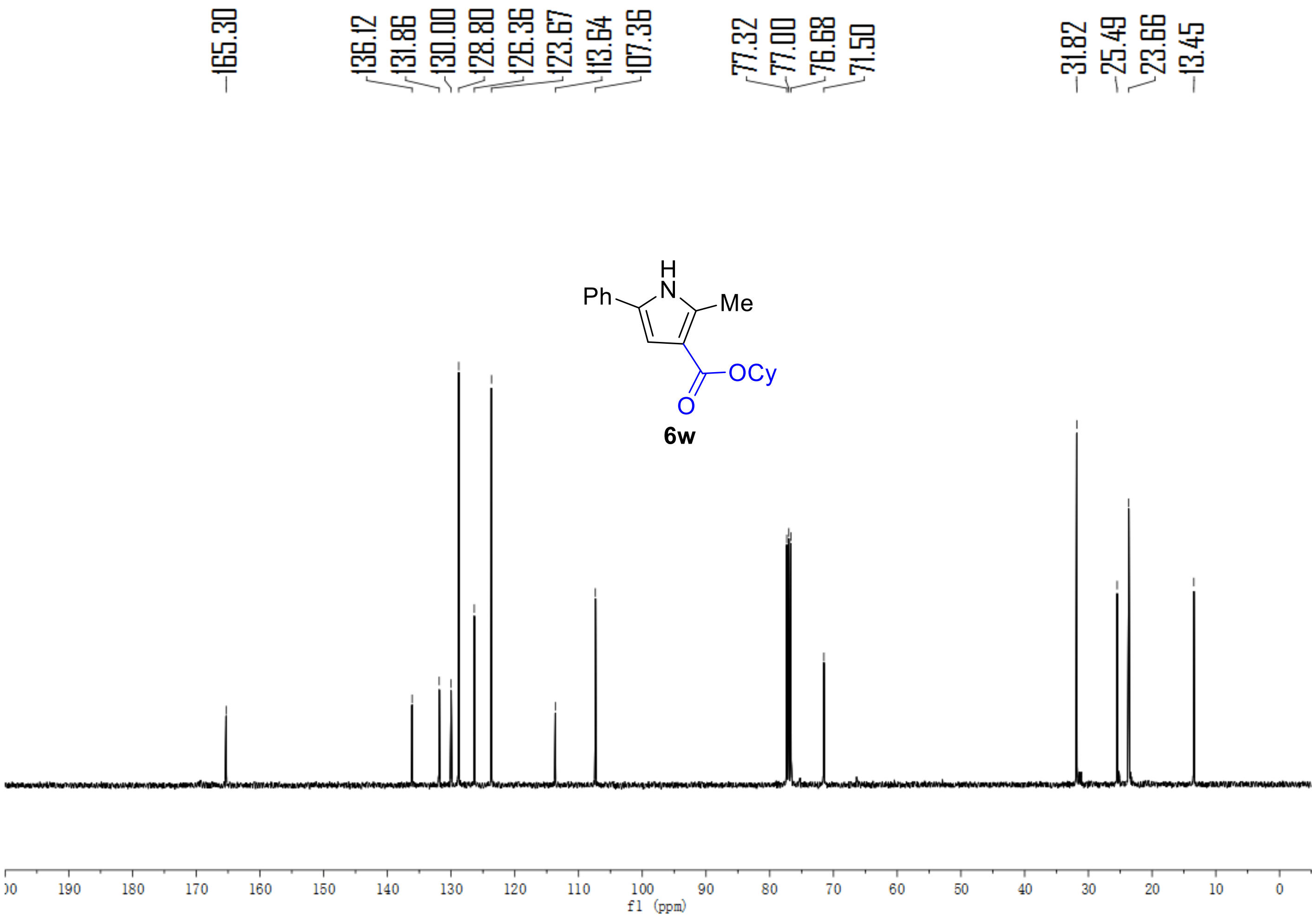

Supplementary Figure 132. ^{13}C NMR of **6w**

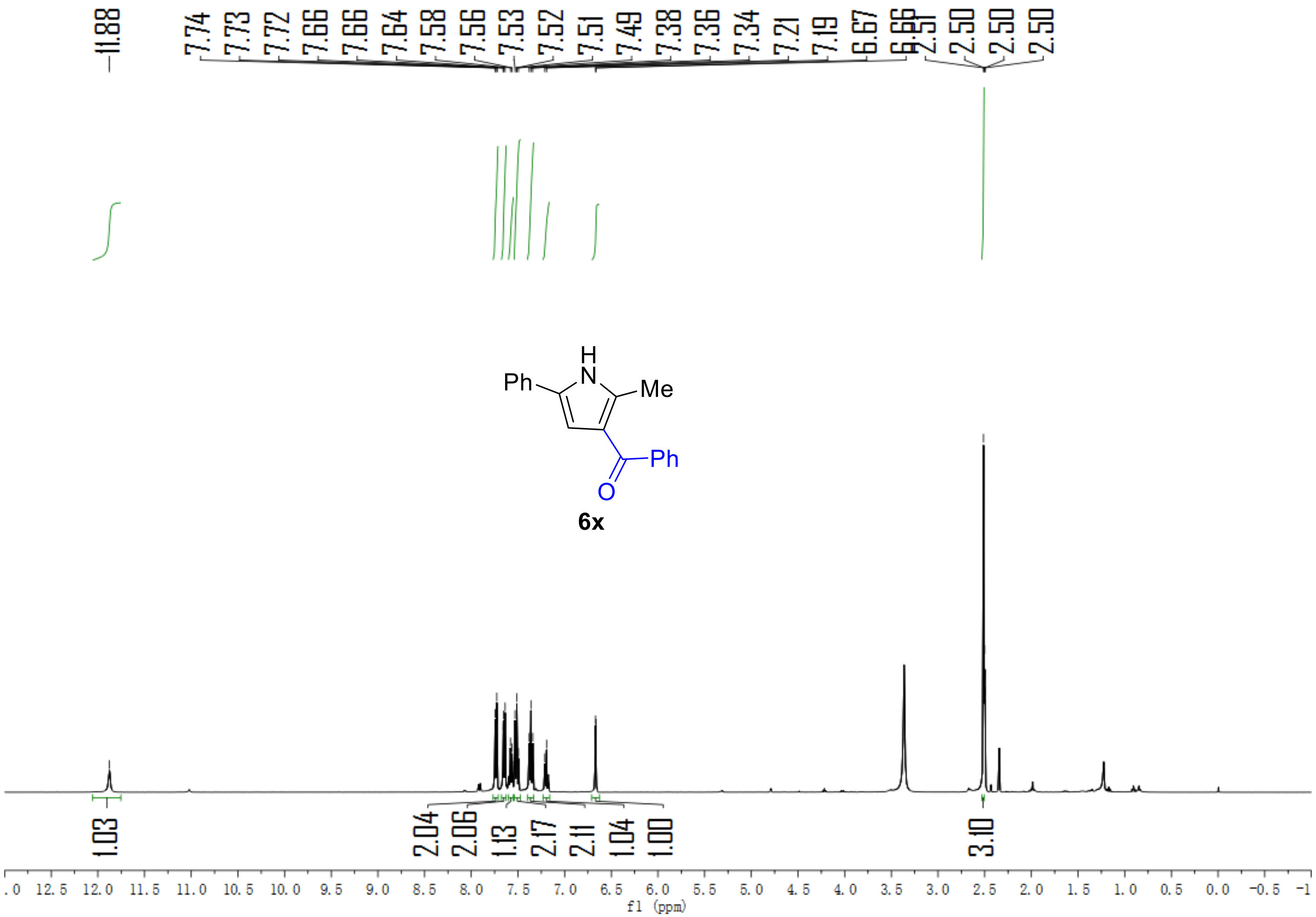

6x

Supplementary Figure 133. ^{1}H NMR of **6x**

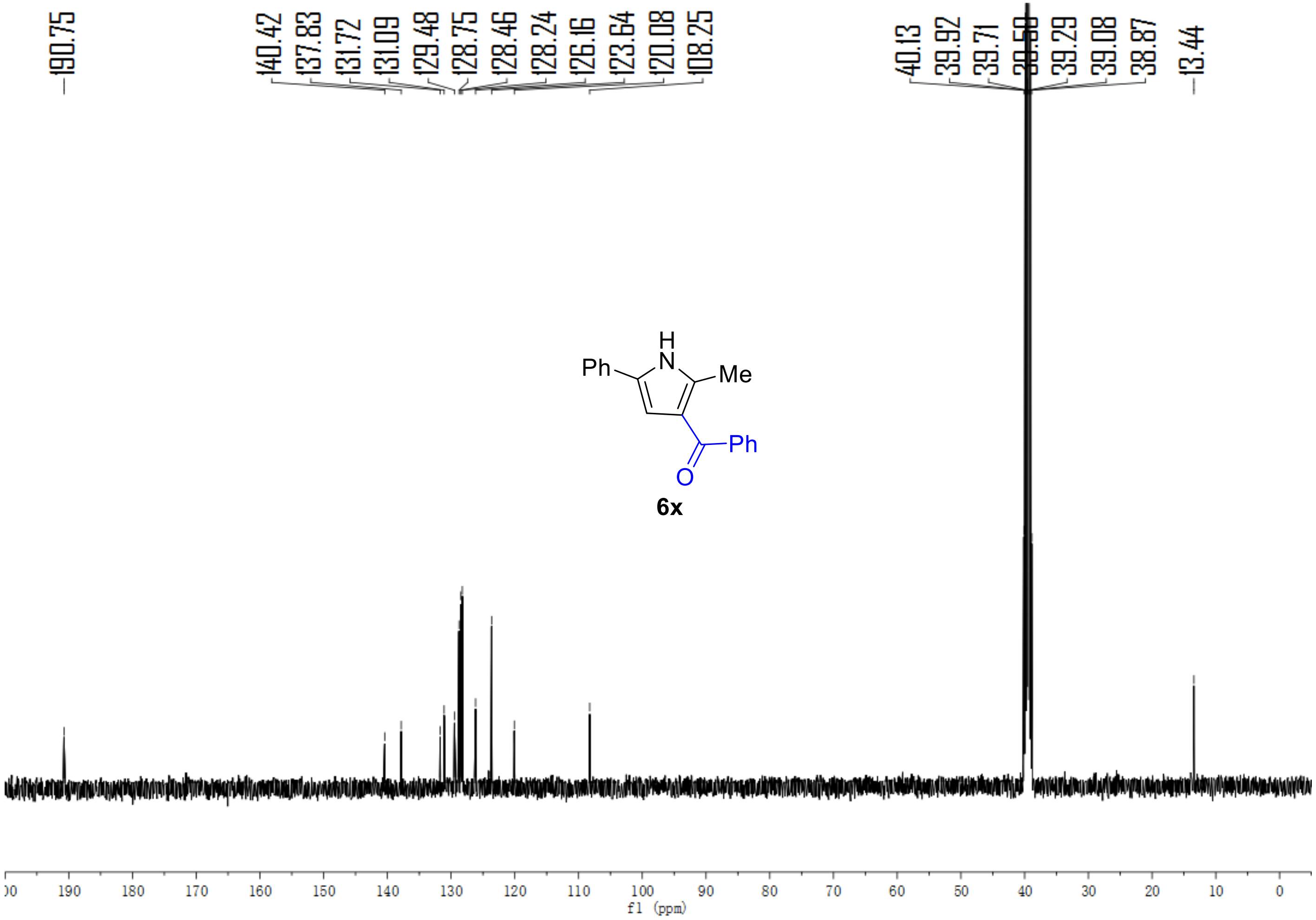

Supplementary Figure 134. ^{13}C NMR of **6x**

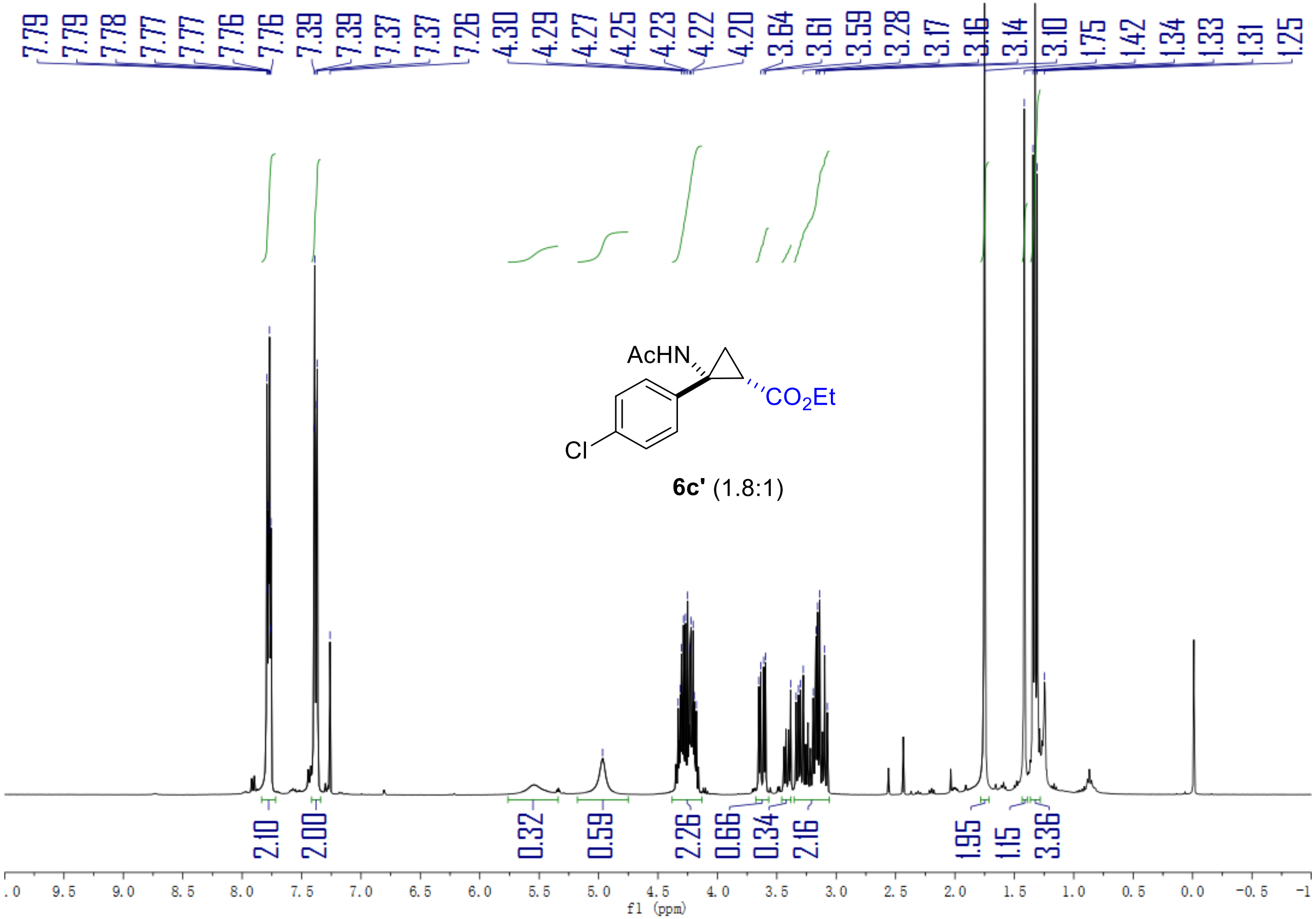

Supplementary Figure 135. ^{1}H NMR of **6c'**

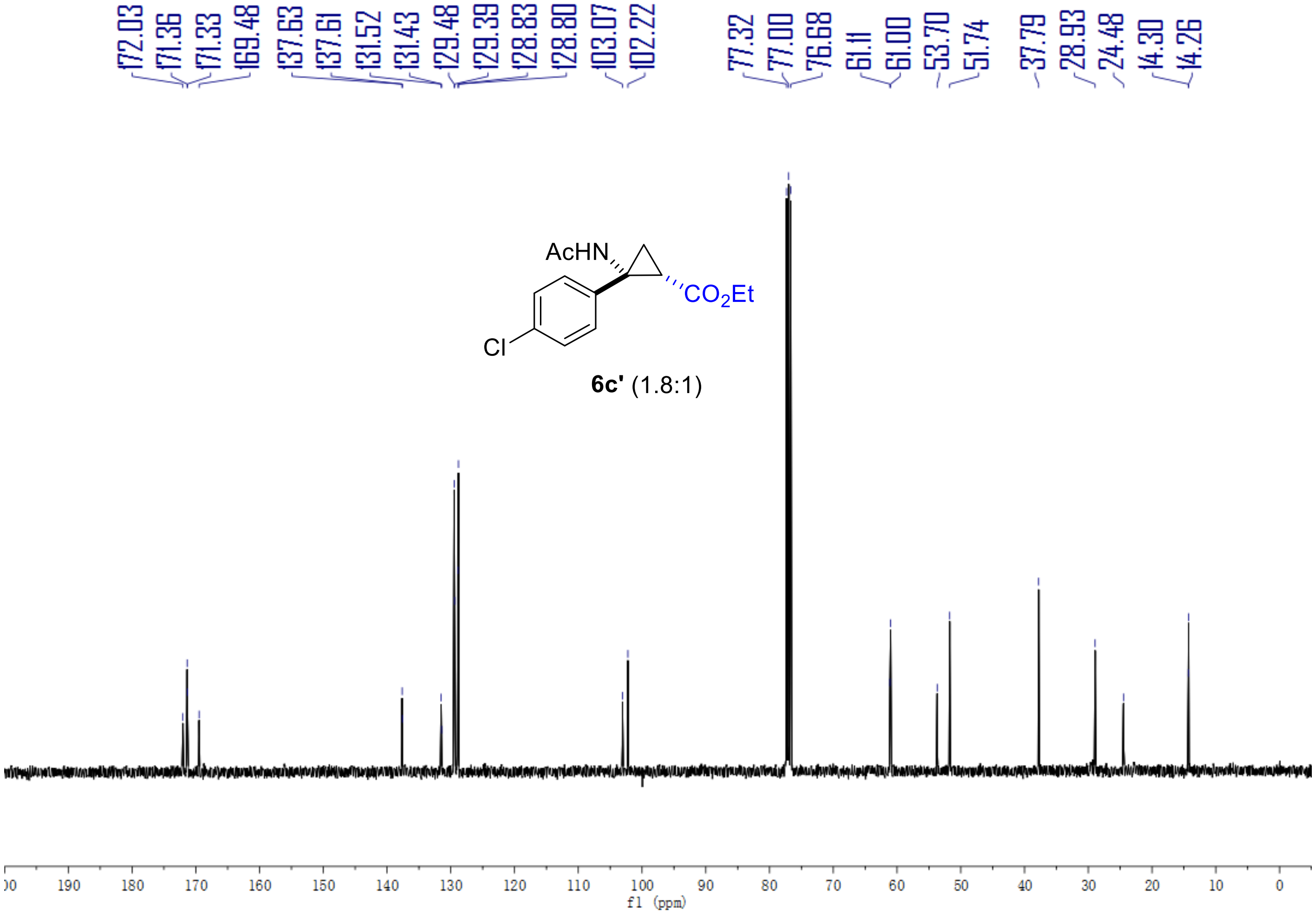

Supplementary Figure 136. ^{13}C NMR of **6c'**

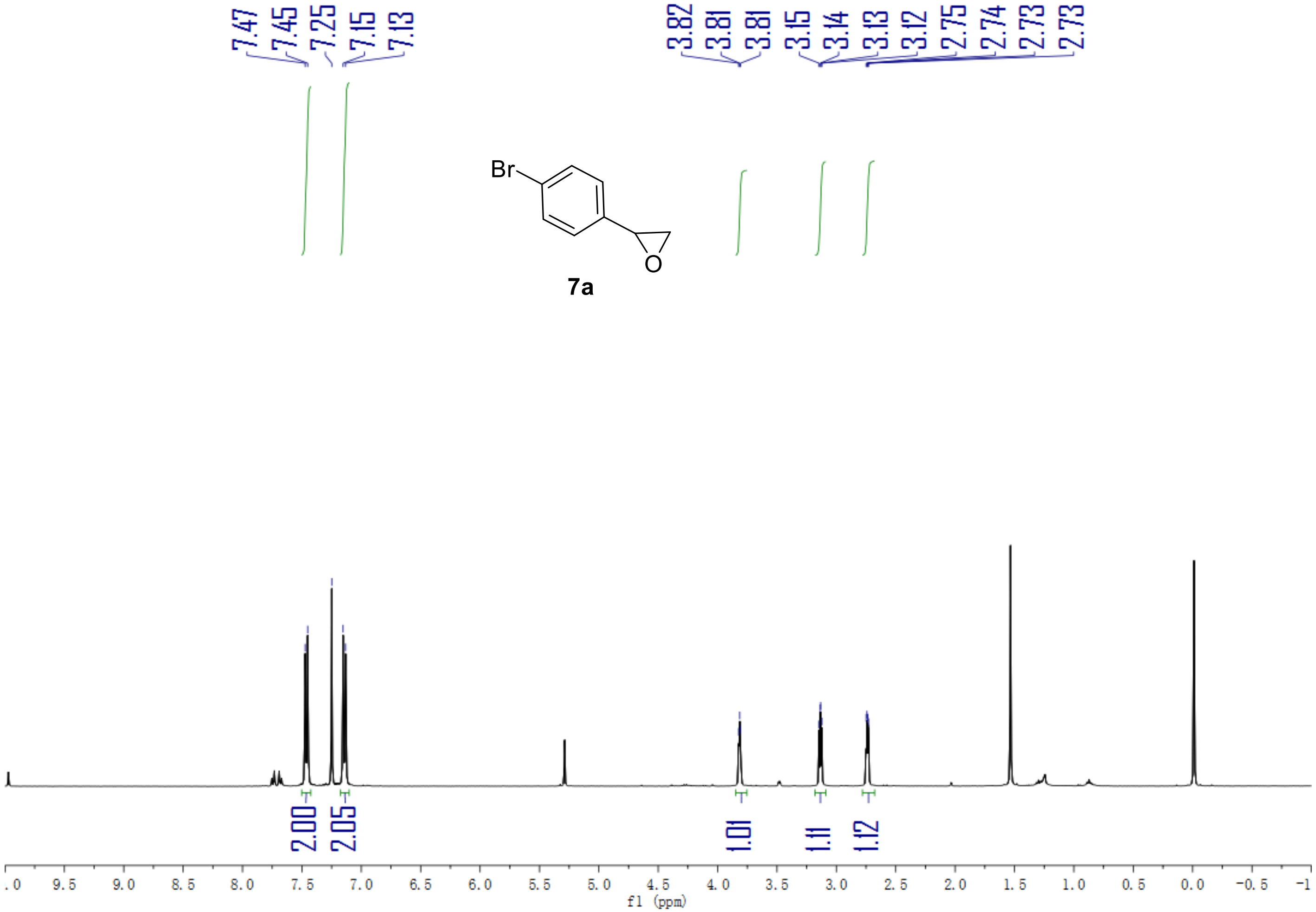

7a

Supplementary Figure 137. ^{1}H NMR of **7a**

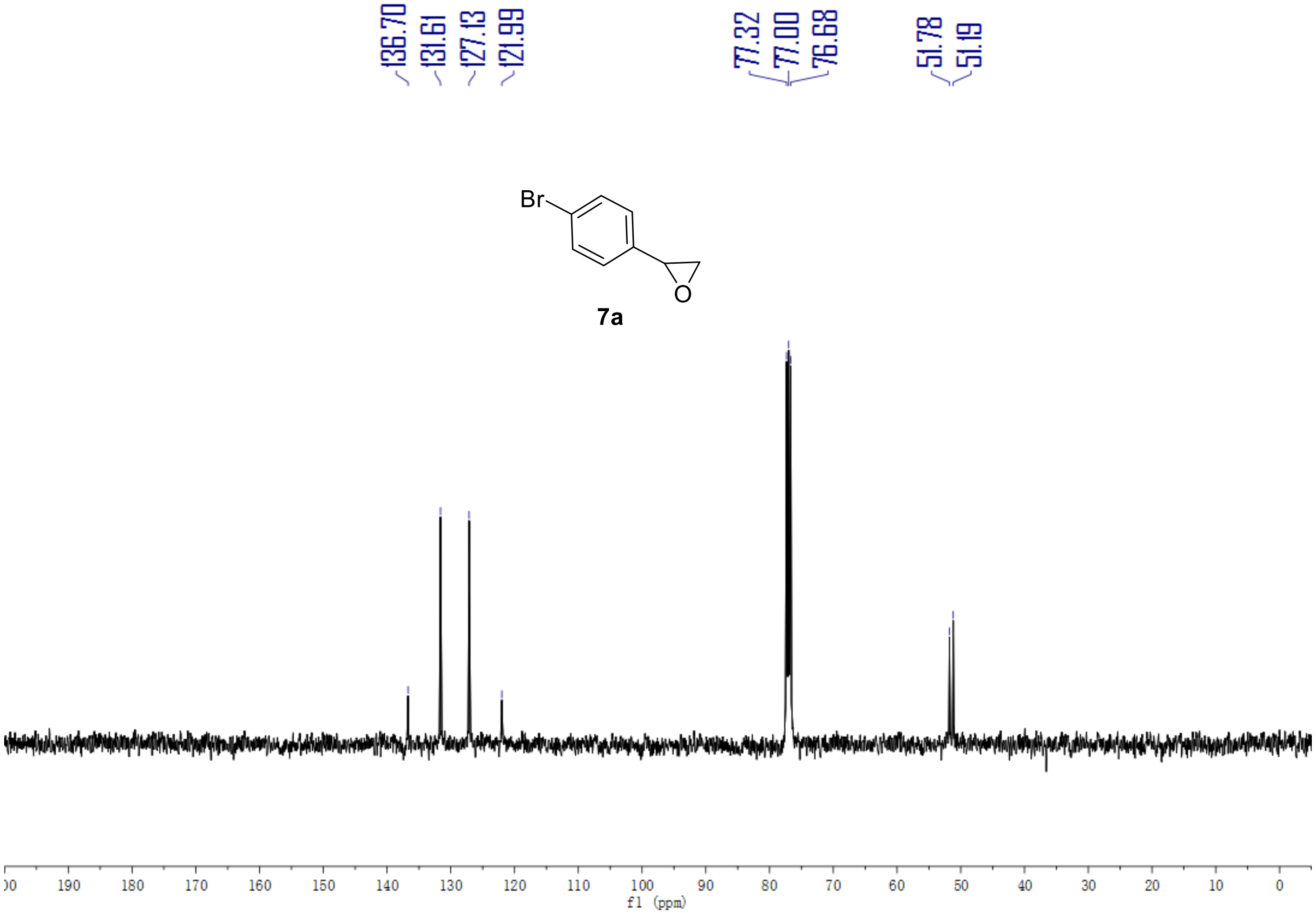

Supplementary Figure 138. ^{13}C NMR of **7a**

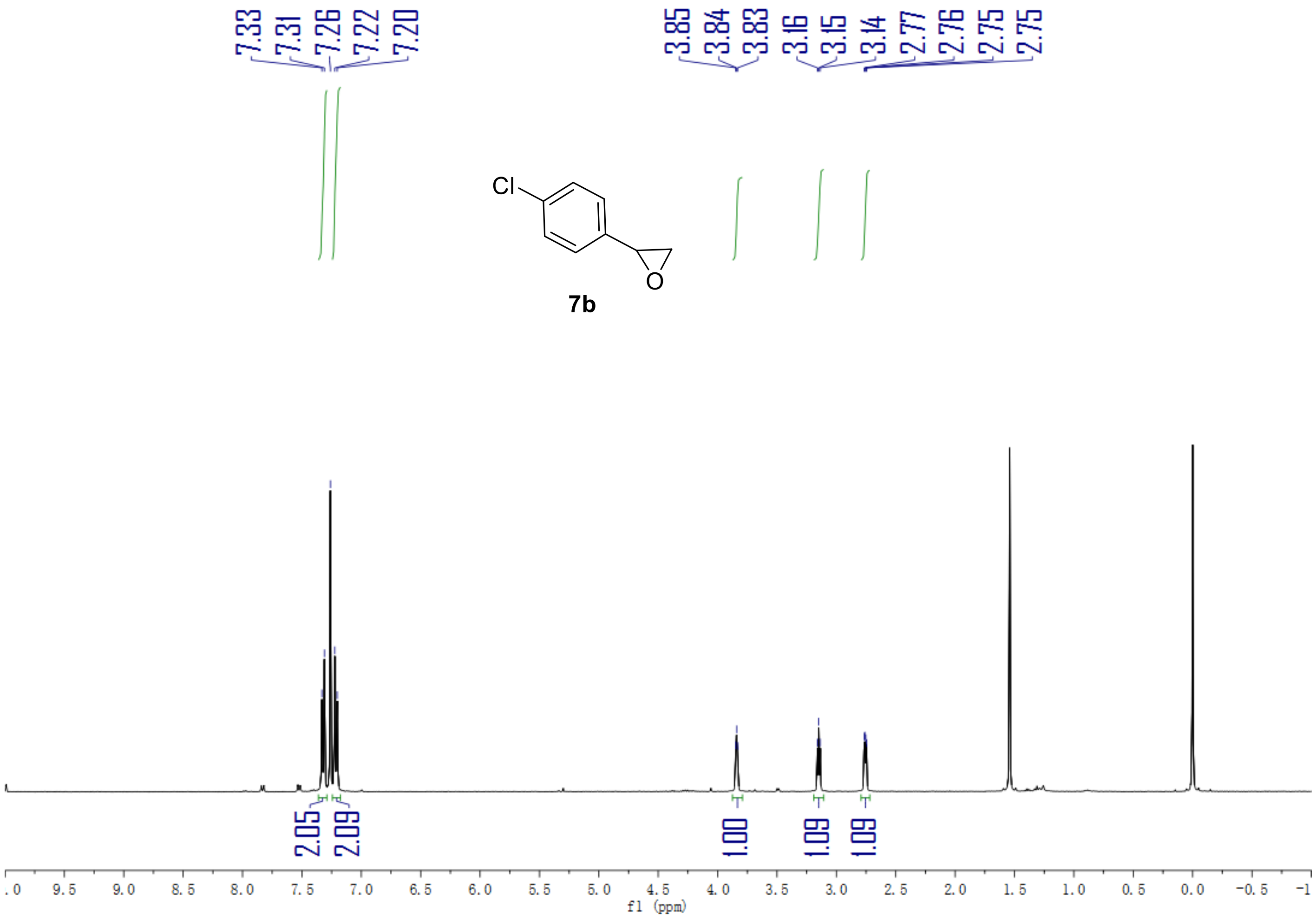

Supplementary Figure 139. ^{1}H NMR of **7b**

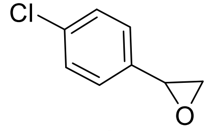

7b

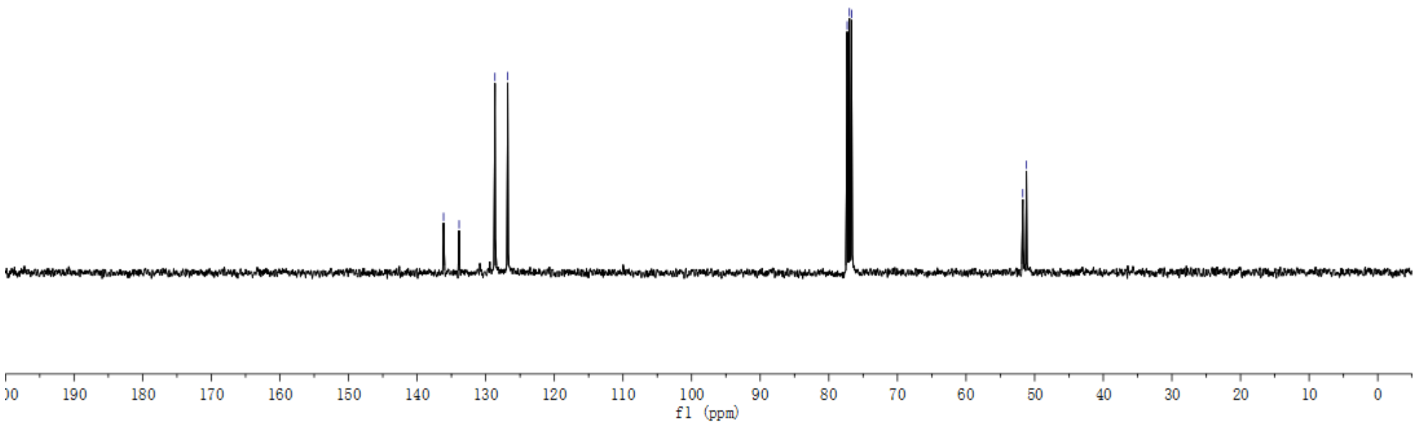

Supplementary Figure 140. ^{13}C NMR of **7b**

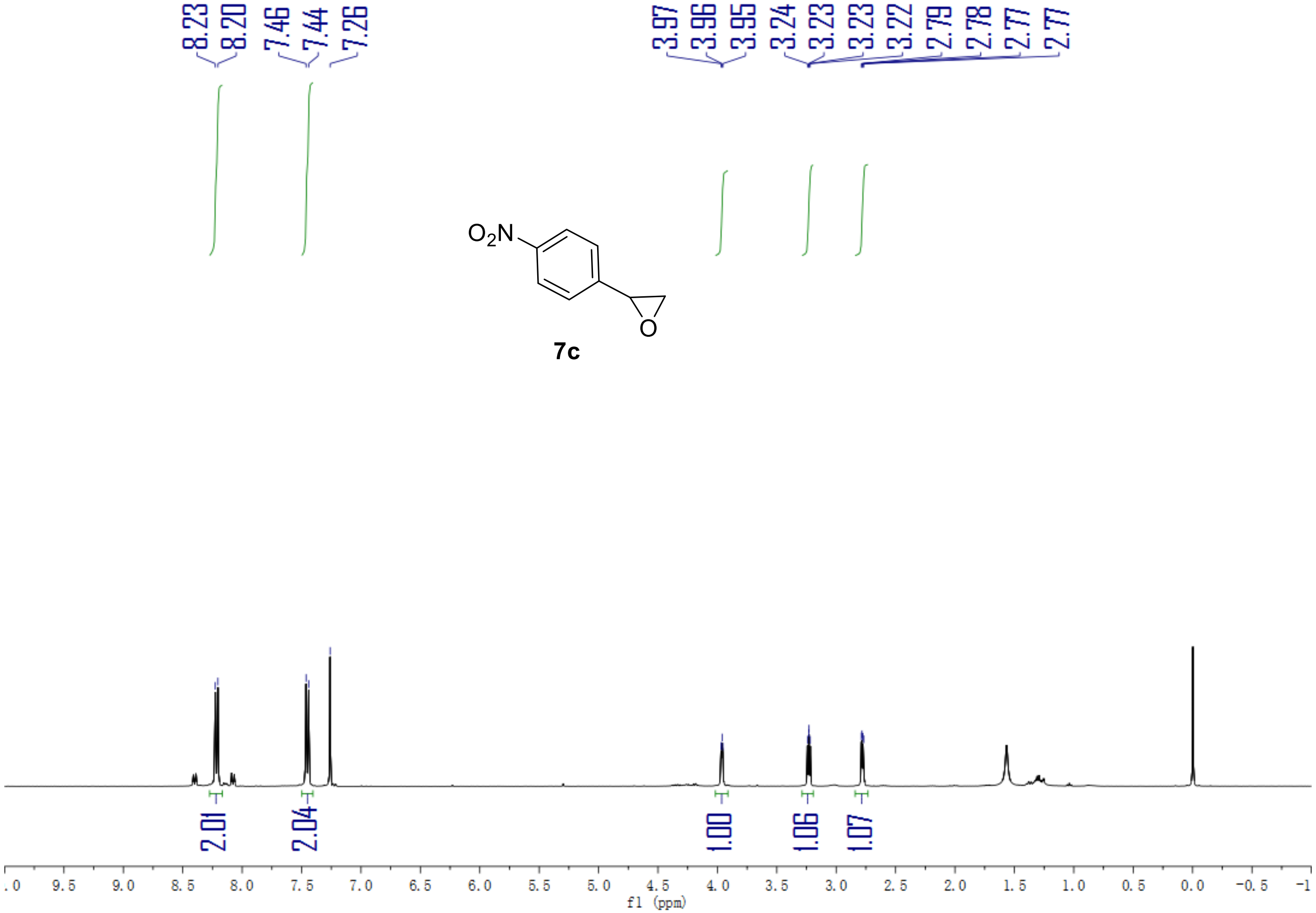

Supplementary Figure 141. ^{1}H NMR of **7c**

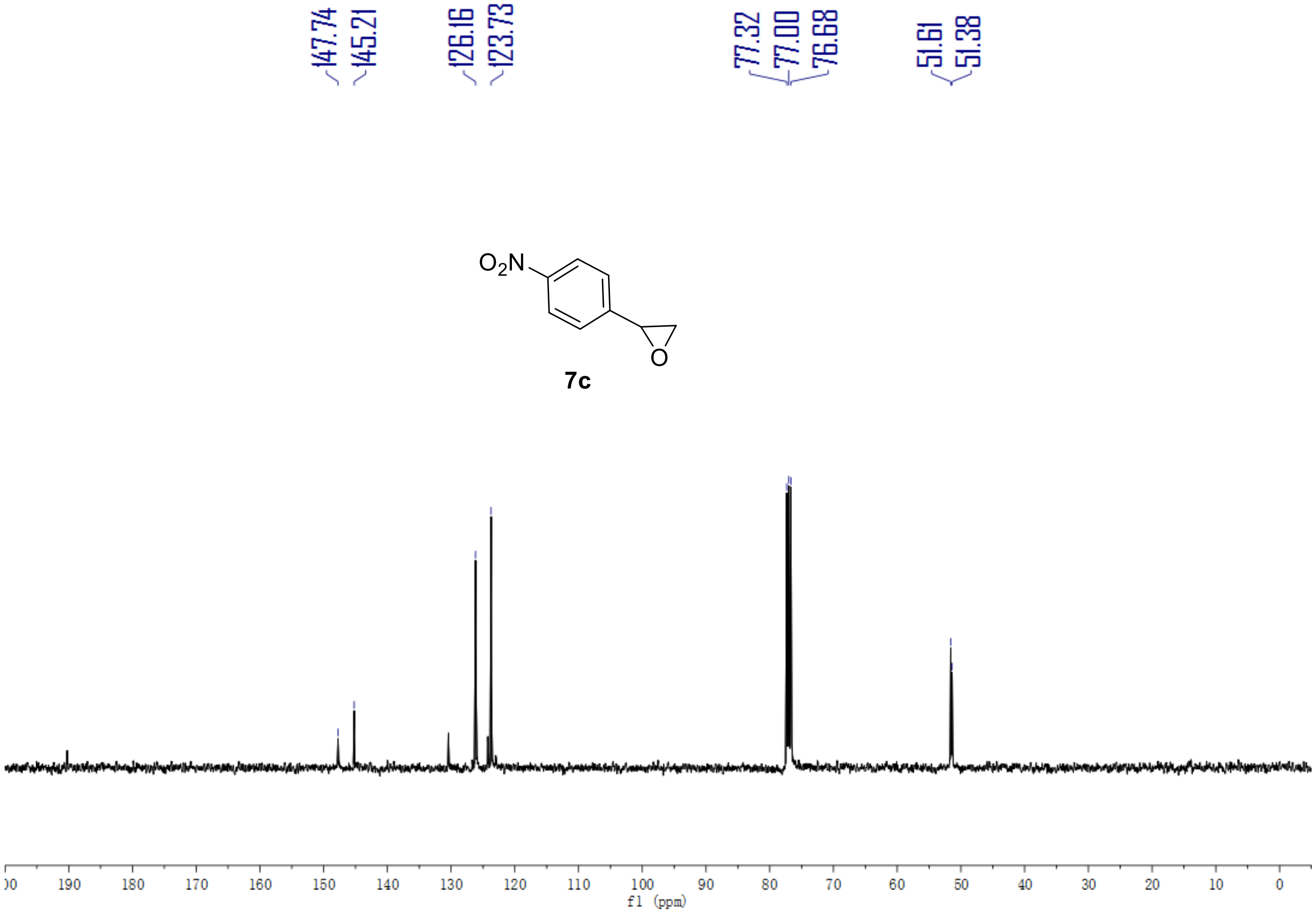

Supplementary Figure 142. ^{13}C NMR of **7c**

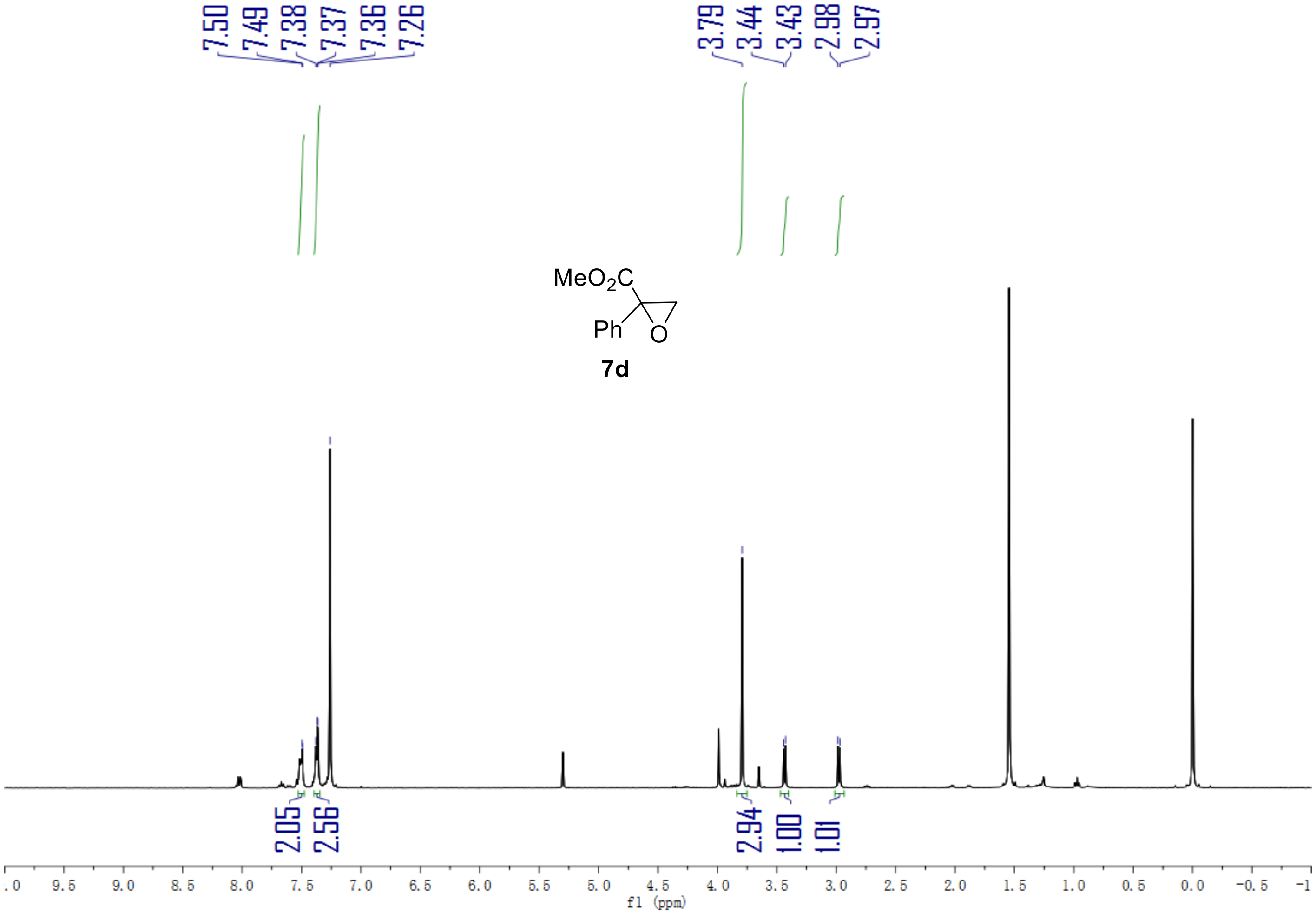

Supplementary Figure 143. ^{1}H NMR of **7d**

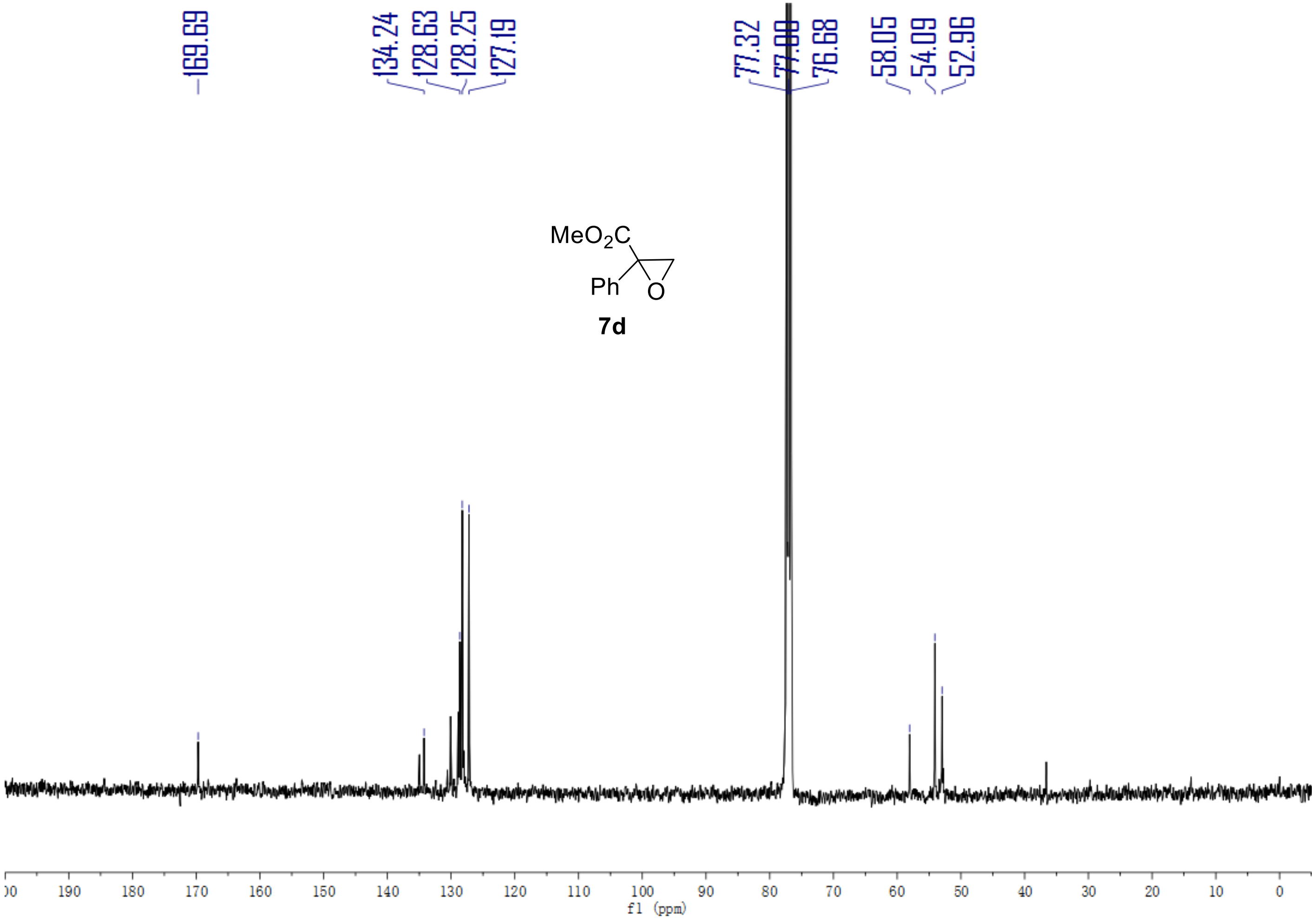

Supplementary Figure 144. ^{13}C NMR of **7d**

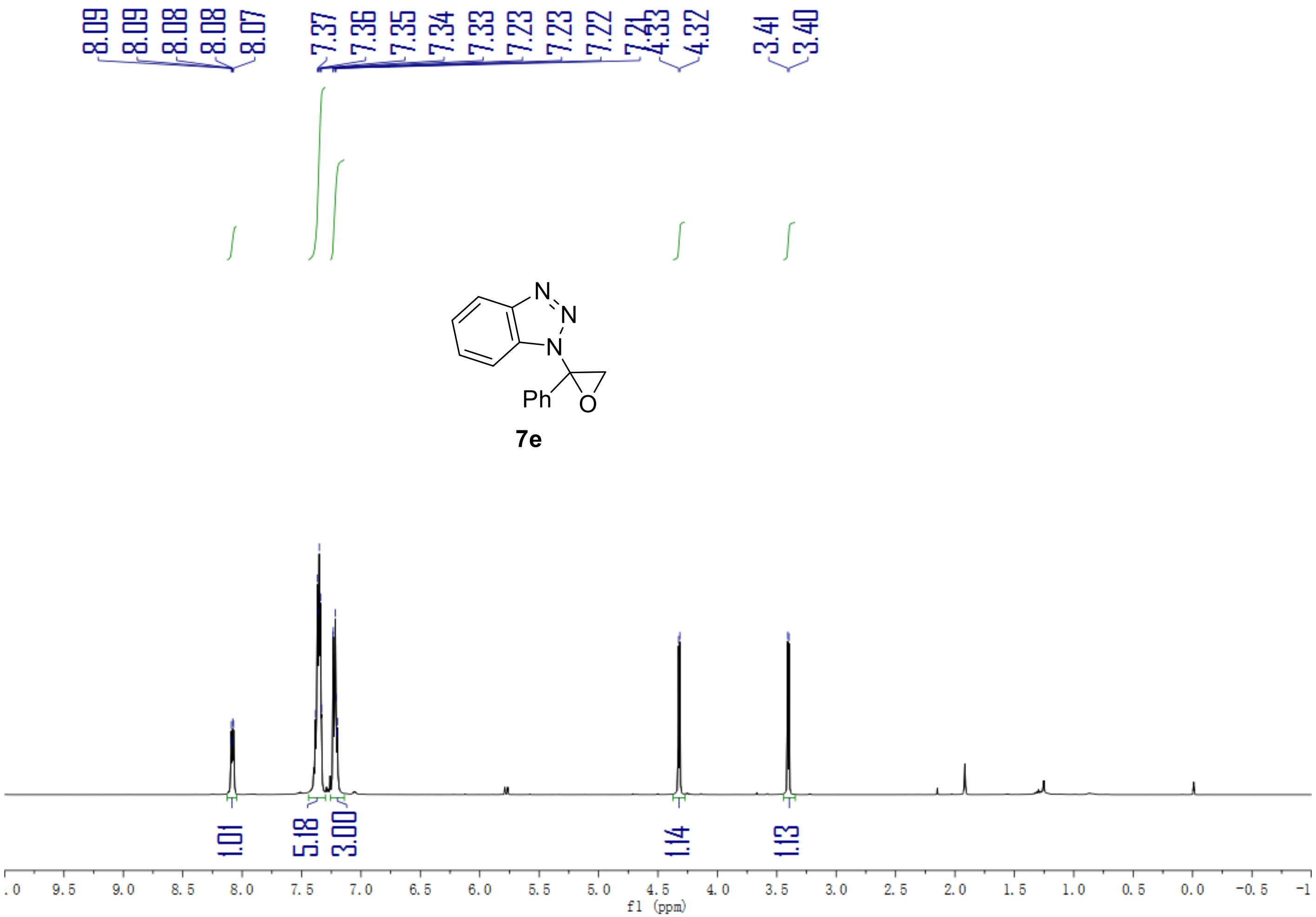

Supplementary Figure 145. ^{1}H NMR of **7e**

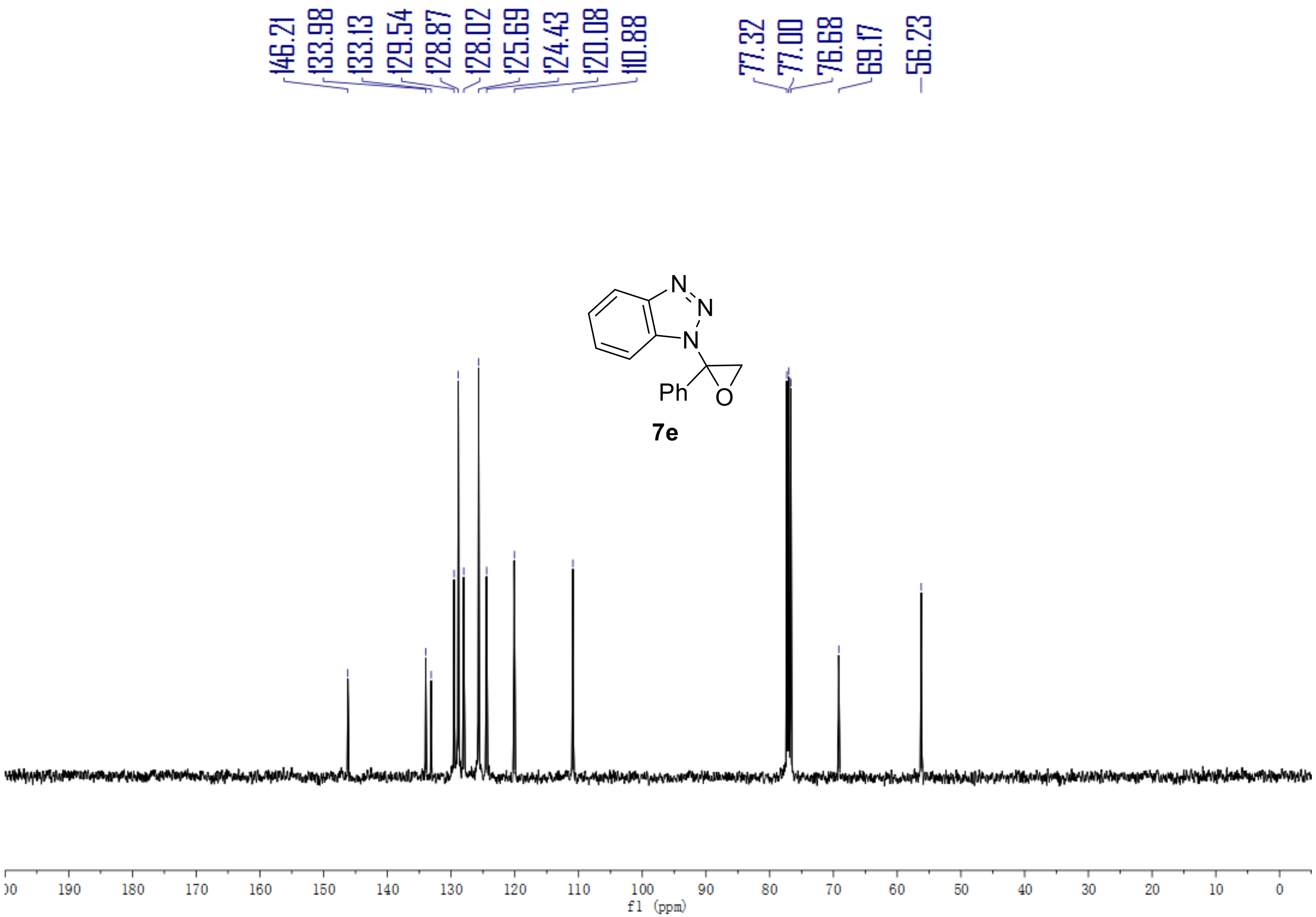

Supplementary Figure 146. ^{13}C NMR of **7e**
